# Distal C–H functionalization of alkoxyarenes through organic photoredox-catalyzed radical–radical coupling†

**DOI:** 10.1039/d4sc08407a

**Published:** 2025-01-29

**Authors:** Yamato Goto, Hirohisa Ohmiya

**Affiliations:** a Institute for Chemical Research, Kyoto University Gokasho, Uji Kyoto 611-0011 Japan ohmiya@scl.kyoto-u.ac.jp

## Abstract

We developed a distal C–H functionalization of electron-rich arenes such as alkoxyarenes using diverse coupling partners through organic photoredox catalysis. Our reaction features radical–radical coupling between a cyclohexadienyl radical and various persistent radicals. A range of functional groups, including sp^2^ carbon-based groups such as alkoxycarbonyl and pyridyl, as well as heteroatom-containing groups like sulfonyl, and alkyl groups were successfully introduced.

## Abstract 2

We developed a distal C–H functionalization of electron-rich arenes such as alkoxyarenes using diverse coupling partners through organic photoredox catalysis.

## Graphical abstract

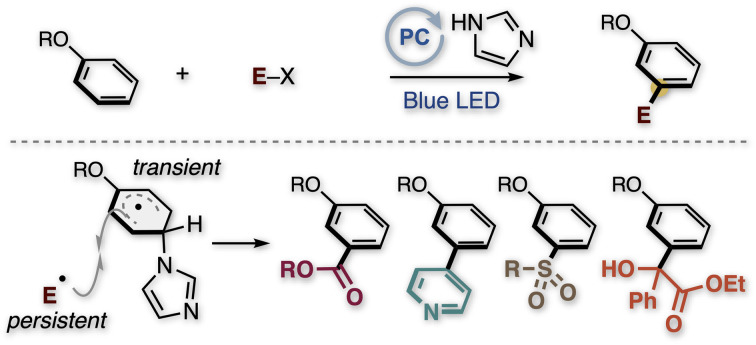

## Introduction

**Scheme 1:**
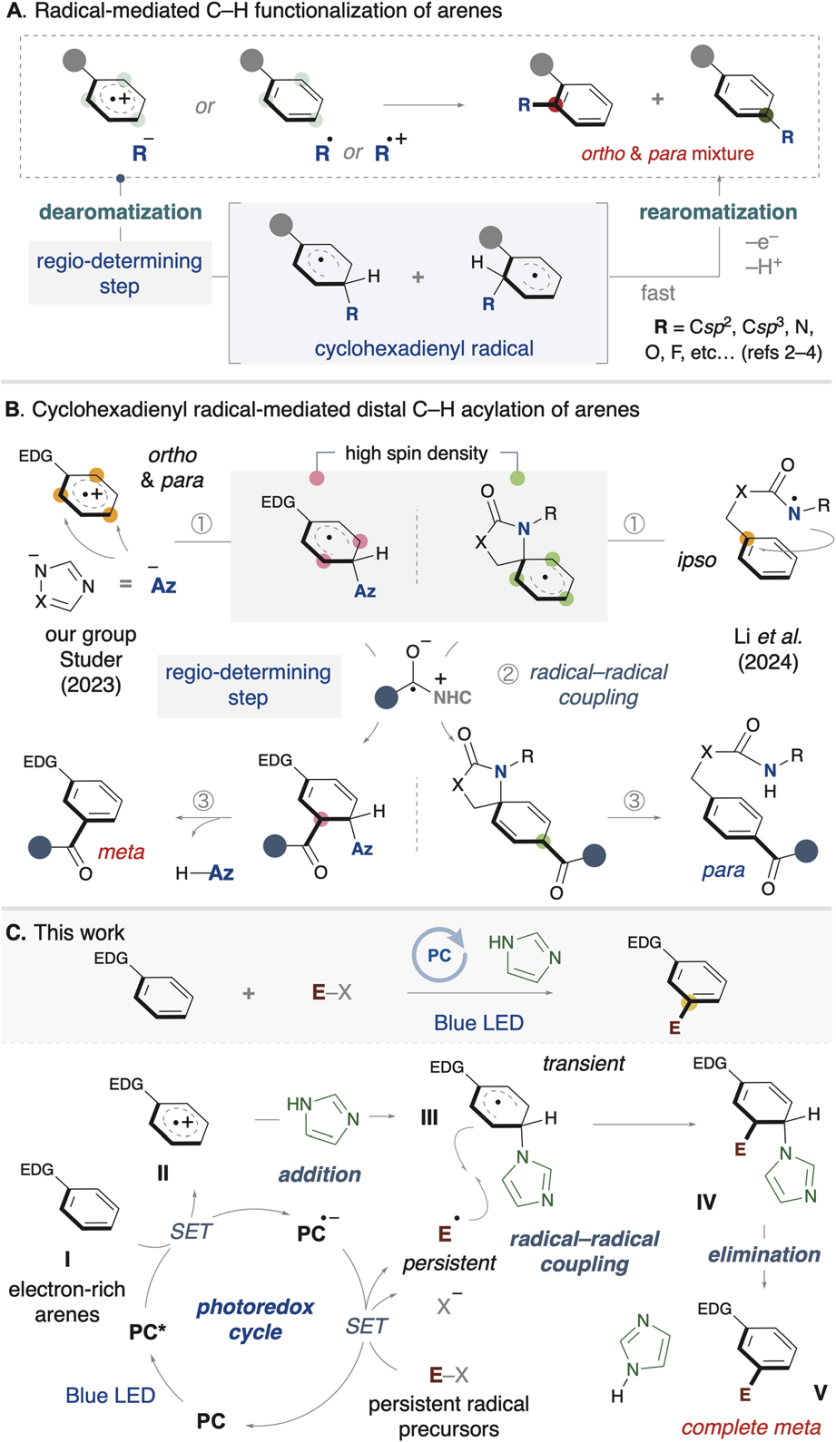
Regioselective radical functionalization of arenes.

Radical-mediated C–H functionalization has gained significant attention from many organic chemists in recent years.^1^ Among these studies, arene C–H functionalization has also been widely explored, and various functional groups have been successfully introduced into aromatic rings.^2–4^ This process is triggered by either addition of an anionic nucleophile to an arene radical cation or addition of an active radical to an inert arene (Scheme 1A). Typically, these reactions involve two key steps: (1) the temporary dearomatization of the arene to form a cyclohexadienyl radical, and (2) subsequent rearomatization *via* single-electron oxidation. Notably, a common feature of these reactions is their *ortho*- and *para*-selectivity. There are also a few examples of *meta*-C–H functionalization *via* radical addition to arenes accomplished through Ru-catalyzed C–H activation^2*e*,*f*^ or non-covalent interactions.^3*g*^ In most reported cases, the products are obtained as mixtures of regioisomers such as *ortho* and *para* isomers, highlighting the remaining challenge of achieving high regioselective control. Since the regio-determining step in these reactions is induced by the initial addition, the observed selectivity is typically influenced by the electronic properties of the arene.

On the other hand, the use of cyclohexadienyl radical intermediates in bond formation has recently emerged as a strategy for C–H functionalization of arenes. The system enables the activation of distal C–H bonds at the *meta* or *para* positions with complete regioselectivity. Earlier, our group and Studer developed a *meta*-selective acylation of arenes by means of NHC/photoredox catalysis (Scheme 1B).^5^ The following year, Li reported a *para*-selective acylation.^6^ The proposed reaction pathway involves three key steps: (1) dearomatization of the arene, (2) radical–radical coupling with a ketyl radical, and (3) rearomatization. In our group's system, an azole nucleophile adds to an arene radical cation, generating a cyclohexadienyl radical with high spin density at the *meta* position. In Li's system, a nitrogen radical on the directing group undergoes *ipso*-cyclization with the arene, generating a spiro-cyclohexadienyl radical. It is notable that the acylation proceeds with complete regioselectivity in both systems (either *meta* or *para*). In these strategies, the radical–radical coupling step is the regio-determining step, which depends on the π-radical distribution in the dearomatized arene, in contrast to previous examples of radical C–H functionalization shown in Scheme 1A. However, the functional groups that can be introduced are limited to acyl groups, indicating a lack of diversity at present.^7^

To introduce a diverse range of functional groups, we devised a synthetic strategy using cyclohexadienyl radical species (Scheme 1C). First, a single-electron transfer between the electron-rich arene (I) and the photoredox catalyst, excited by visible light, results in the generation of an arene radical cation (II). Next, a nucleophile, such as an azole (or azolide anion), temporarily adds to the *ortho* or *para* position of the arene radical cation, yielding a cyclohexadienyl radical (III). Meanwhile, the redox-active substrate (E–X) undergoes single-electron transfer with the photoredox catalyst, forming a radical anion species, or it undergoes mesolytic cleavage to generate a neutral radical species. Then, a radical–radical coupling between the cyclohexadienyl radical and the persistent radical species occurs, yielding a difunctionalized intermediate (IV). Finally, the product (V) is formed by rearomatization, and the azole is regenerated.

## Results and discussion

First, we designed the alkoxycarbonylation of electron-rich arenes using alkoxycarbonylation reagents^8^ and performed screening of the reaction conditions (Table 1). In the presence of 1,3-dimethoxy-2-methylbenzene 1a and an imidazolium ester 2a, and upon irradiation with 390 nm light, the use of an organic photocatalyst PC-1 with a high reduction potential in its excited state (+2.07 V *vs.* SCE),^9^ an imidazole nucleophile Az-1, and dichloromethane as the solvent resulted in the formation of the desired alkoxycarbonylated product 3aa as a single regioisomer in 78% yield (entry 1). Along with 3aa, an aryl imidazole 3aa′, formed through the overoxidation of the cyclohexadienyl radical intermediate, was identified as a side product (see Fig. 2A). However, when a less oxidizing photocatalyst PC-2 (+1.56 V *vs.* SCE) was employed, only a trace amount of 3aa was obtained (entry 2). No reaction occurred when PC-3 or PC-4 was employed and the arene was recovered quantitatively (entries 3 and 4). Similarly, the reaction did not proceed at all when bisphosphonium salt PC-5 and pyrylium salt PC-6, both possessing high oxidizing power, were used (entries 5 and 6). Next, azoles were screened. The use of a bulkier imidazole derivative (Az-2) led to a significant decrease in product yield (entry 7). A similar result was observed with Az-3, which possesses comparable nucleophilicity but produced an even lower yield (entry 8). The less nucleophilic Az-4 and the more nucleophilic Az-5 also failed to deliver the product (entries 9 and 10). When these azoles were tested, a complex mixture was obtained instead of the desired product. When the equivalents of the azole nucleophile were reduced, the product yield decreased significantly (entries 11 and 12). Because azole nucleophiles are consumed through the formation of byproducts (see Fig. 2A) or the retention of reaction intermediates (see Fig. 2B), their concentration decreases during the reaction. Maintaining a higher concentration of the azole nucleophile might have enhanced reaction efficiency. Control experiments were performed, showing that the reaction did not occur in the absence of a photocatalyst, and that the presence of an azole nucleophile was essential (entries 13 and 14). Furthermore, light irradiation, particularly at 390 nm, was crucial for the reaction to proceed (entries 15 and 16). Considering the unfavorable electron transfer between the imidazolium ester (−1.5 V *vs.* SCE)^8^ and PC-1 radical anion (−0.73 V), the involvement of a ConPET process was suspected (see Fig. 2D and E).^10^

**Table 1:** Screening of photoredox catalysts and azole nucleophiles for reaction between 1a and 2a^a^

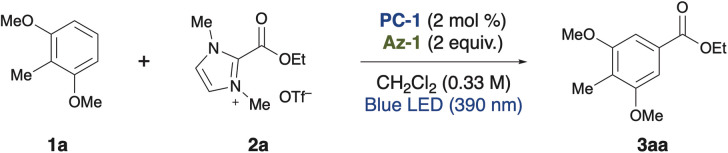			
Entry	Deviation from the standard conditions	Yield of 3aa^b^ (%)	Recovery of 1a^b^ (%)
1	None	78 (69)^c^	25
2	PC-2 instead of PC-1	2	Quant.
3	PC-3 instead of PC-1	0	Quant.
4	PC-4 instead of PC-1	0	Quant.
5	PC-5 instead of PC-1	1	Quant.
6	PC-6 instead of PC-1	1	Quant.
7	Az-2 instead of Az-1	11	93
8	Az-3 instead of Az-1	6	12
9	Az-4 instead of Az-1	0	17
10	Az-5 instead of Az-1	0	3
11	Az-1 (1 equiv.)	29	32
12	Az-1 (50 mol%)	14	32
13	W/o PC-1	2	Quant.
14	W/o Az-1	0	63
15	Under dark	0	124
16	440 nm light source	0	Quant.
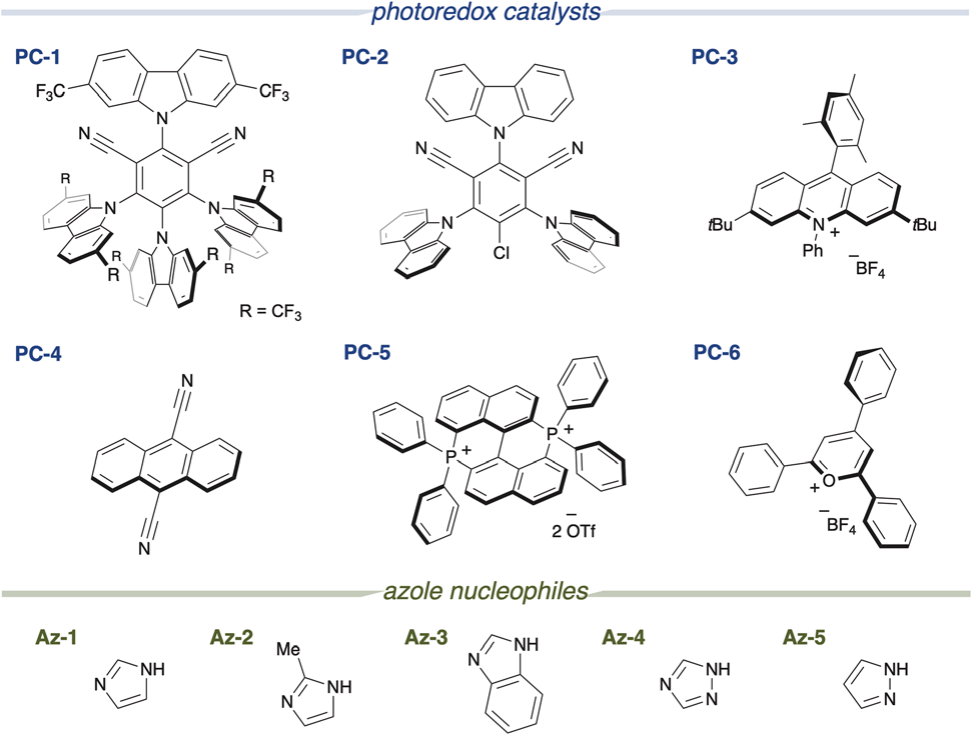			

a Reaction was carried out with 1a (0.15 mmol), 2a (0.1 mmol), a photoredox catalyst (0.002 mmol) and an azole nucleophile (0.2 mmol) in dichloromethane (0.3 mL) under blue LED (390 nm Kessil lamp, 100% intensity) irradiation for 16 h.

b ^1^H-NMR yield based on 2a.

c Isolated yield.

**Fig. 1:**
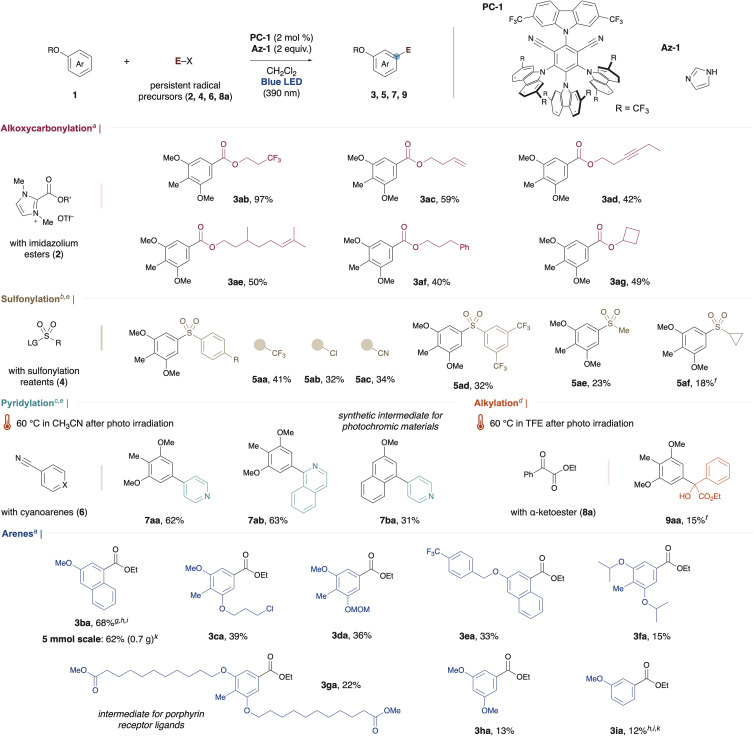
Substrate scope. Unless otherwise noted, the reaction was carried out with arene 1, persistent radical precursor, PC-1 (0.004 mmol), and Az-1 (0.4 mmol) in CH_2_Cl_2_ under blue LED (390 nm Kessil lamp, 100% intensity) irradiation for 16 h on a 0.2 mmol scale. Detailed reaction conditions were categorized into method A through D. ^*a*^Method A: 1 (0.3 mmol) and 2 (0.2 mmol) in CH_2_Cl_2_ (0.6 mL). ^*b*^Method B: 1 (0.2 mmol) and 4 (0.6 mmol) in CH_2_Cl_2_ (2.0 mL). ^*c*^Method C: 1 (0.2 mmol) and 6 (0.6 mmol) in CH_2_Cl_2_ (2.0 mL). ^*d*^Method D: 1a (0.3 mmol) and 8a (0.1 mmol) in CH_2_Cl_2_ (0.33 mL). ^*e*^Cs_2_CO_3_ (0.2 mmol) was used. ^*f*^0.1 mmol scale. ^*g*^1.0 mmol scale. ^*h*1^H-NMR yield. ^*i*^0.5 mmol scale. ^*j*^Photo irradiation for 24 h. ^*k*^Photo irradiation for 48 h.

Afterward, we commenced our investigation into the substrate scope of alkoxycarbonylation under optimized conditions (Fig. 1). Using imidazolium esters substituted with the trifluoromethyl group, we successfully obtained the desired ester in excellent yield (3ab). The reaction proceeded with moderate efficiency, even for substrates containing alkenes (3ac and 3ae), an alkyne (3ad), and an aromatic ring (3af). Notably, no decrease in yield was observed when esters derived from secondary alcohols were employed (3ag).

Next, we extended the scope to sulfonylation using sulfonylating reagents. In addition to the above conditions, the addition of cesium carbonate enabled the successful synthesis of diaryl sulfones from sulfonyl triazoles (5aa–5ad, see the ESI†). Aryl groups substituted with electron-withdrawing groups gave better yields than electron-donating groups, while no product was formed with alkyl-substituted groups. However, the use of alkyl sulfonylimines led to the desired alkyl sulfonylation, although yields remained low (5ae and 5af).^11^

**Fig. 2:**
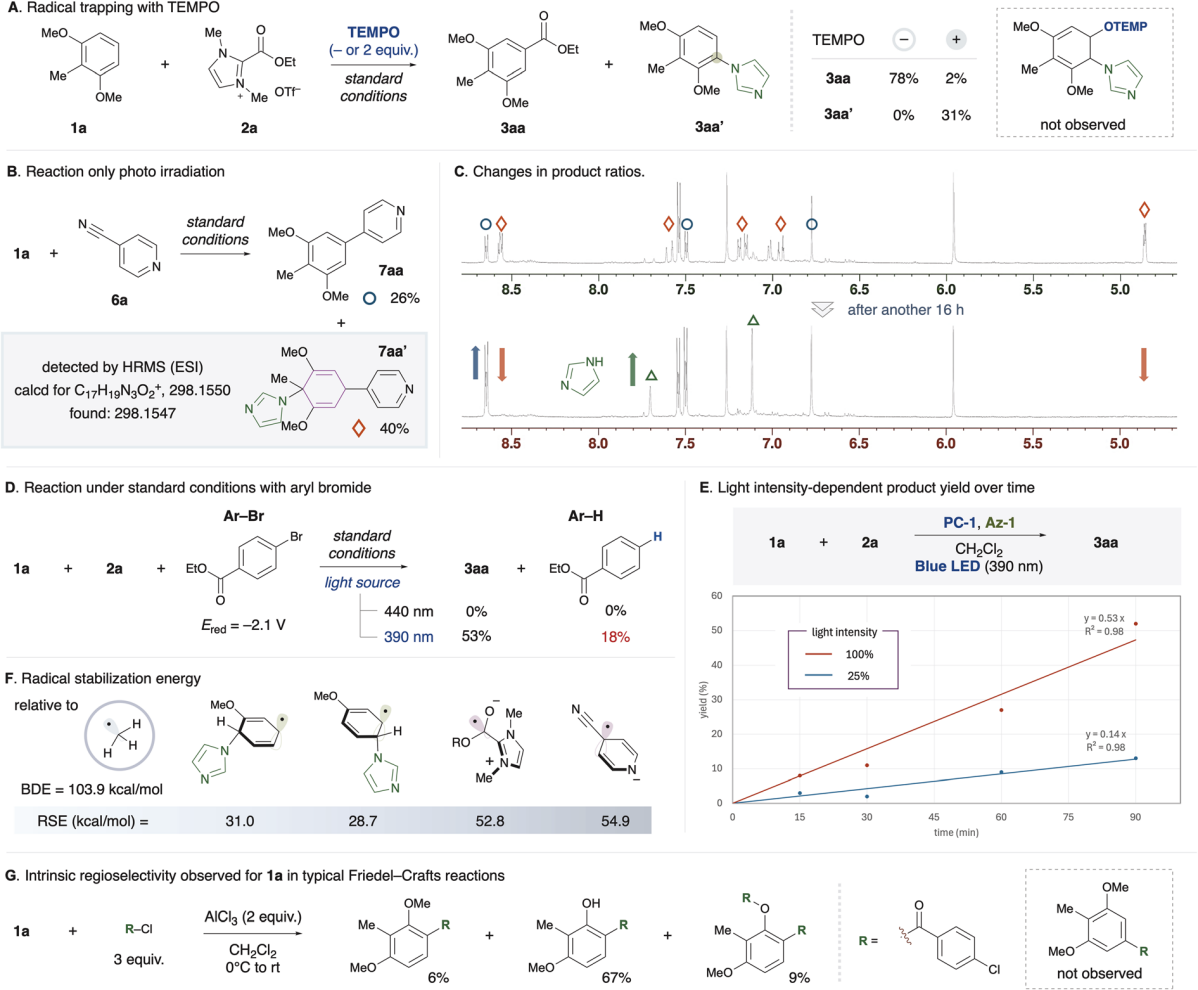
Mechanistic studies. (A) Radical trapping with TEMPO. (B) Reaction only photo irradiation. (C) The peaks of the target product (blue circle), byproducts (orange diamond shape), and imidazole (green triangle) were monitored by ^1^H-NMR (above: after photo irradiation, below: after another 16 h). (D) The value of the redox potential was reported *versus* SCE in MeCN. (E) The yield of 3aa under different light intensities (390 nm Kessil lamp, 100% or 25%) during the reaction between 1a and 2a over time was compared. (F) Each RSE value was calculated using the CBS-QB3//(U)ωB97X-D/def2-TZVPP level of theory. (G) Reaction was carried out with arene 1a (0.2 mmol), 4-chlorobenzoyl chloride (0.6 mmol), and AlCl_3_ (0.4 mmol) in CH_2_Cl_2_ (3.0 mL) with stirring, starting at 0 °C, warming to room temperature, and continuing for a total of 20 h.

We then developed a heteroarylation using cyano-substituted arenes. This reaction required heating after photo irradiation to complete. Without this step, reaction intermediates were observed (see Fig. 2B and the ESI†). Using 4-cyanopyridine as the pyridylating agent, we achieved a 62% yield of 7aa. Isoquinoline was also successfully incorporated (7ab). Additionally, 7ba is reported as a candidate for photochromic materials.^12^ Previously, it required a multi-step synthesis from 1,3-dihydroxynaphthalene, but our method allows for its production in a single step from commercially available 2-methoxynaphthalene. When ketoester was used as a coupling partner, *C4*-selective alkylation occurred, albeit with a low yield (9aa).

Notably, the alkoxycarbonylation with 2-methoxynaphthalene was scalable to 5 mmol without any compromise in yield (3ba). Even alkoxyarene substrates with a chloro group (3ca), a MOM-protecting group (3da), or a benzylic position (3ea) underwent the reaction with complete regioselectivity. Although the yield decreased for the bulky alkoxyarene (3fa), this method's ability to synthesize intermediates for porphyrin receptor ligands in a single step is a significant advantage (3ga).^13^ 1,3,5-Trisubstituted arene, known as precursors for dendrimers, was also synthesized from a cheaper simple arene (3ha).^14^ Even though the yield was low, simple anisole could be utilized, and the product was obtained with complete *meta*-selectivity (3ia). Notably, no *ortho* or *para* isomers were detected among the low-yielding products in the crude mixture. Instead, the aryl imidazole was predominantly formed. In the case of substrates with high oxidation potentials such as anisole or arenes substituted with electron-withdrawing groups, a significant amount of starting material remained, and the reaction proceeded at a slower rate (for other unsuccessful examples, see ESI†).

To gain deeper mechanistic insights, we conducted several experiments. First, to clarify the role of azole nucleophiles, we performed radical trapping experiments using TEMPO under standard conditions for the alkoxycarbonylation (Fig. 2A). As a result, the desired product 3aa was not formed. Instead, we observed an aryl imidazole 3aa′ as a byproduct. Given the well-known role of TEMPO as a persistent radical, we anticipated the trapping of the cyclohexadienyl radical, but no such product was detected. In contrast, when 4-cyanopyridine was used for radical trapping, we successfully detected reaction intermediates (Fig. 2B). The aminopyridylated product 7aa′ was characterized using ^1^H-NMR and HRMS. Along with 7aa′, a rearomatized product 7aa was also obtained in 26% yield. We monitored the changes in the product ratios using ^1^H-NMR (Fig. 2C). After 16 hours at room temperature, the peak corresponding to the intermediate had completely disappeared, while peaks for 7aa and an imidazole increased. It suggests that 7aa′ is a key intermediate.

Next, an aryl bromide (Ar-Br) with a redox potential of −2.1 V was added to the standard conditions of the alkoxycarbonylation (Fig. 2D). Under 440 nm light irradiation, the desired product was not obtained. On the other hand, under 390 nm light irradiation, the desired product was obtained along with the reduced form of the aryl bromide (Ar-H). The photoredox catalyst generates a radical anion species through reductive quenching in the excited state. It is widely known that 4CzIPN-type photoredox catalysts exhibit strong reducing power through a ConPET process,^15^ which might enable the single-electron transfer between the photocatalyst and redox-active substrates. In fact, the radical precursors used in Fig. 1 have a more positive *E*_red_ than Ar-Br.^16^

We investigated the dependence of the product (3aa) yield on light intensity during the reaction between 1a and 2a over time (Fig. 2E). When the light intensity was reduced to one-fourth of the normal level (100% to 25%), the yield decreased proportionally to approximately one-fourth, thereby ruling out the involvement of a nonlinear two-photon excitation (simultaneous two-photon absorption) process. These results are consistent with a mechanism in which two photons are sequentially involved (PC to PC* and PC˙^−^ to PC˙^−^*). Although these findings alone do not definitively confirm the involvement of ConPET, their combination with the results shown in Fig. 2D suggests the possibility of the presence of species such as the excited PC˙^−^, which possesses high reducing power and can be excited at 390 nm.

We evaluated the radical species used in this study from both thermodynamic aspects (stability) and kinetic aspects (persistence). To estimate the thermodynamic stability of each radical intermediate, the radical stabilization energy (RSE) was calculated (Fig. 2F). The RSE is defined as the difference between the bond dissociation energy (BDE) of the target C–H bond and the BDE of methane's C–H bond (103.9 kcal mol^−1^). In general, an increase in the RSE value (*i.e.*, a decrease in the BDE value) corresponds to greater stability of the free radical relative to the methyl radical (RSE = 0 kcal mol^−1^). A lower BDE value indicates that the hydrogen atom abstraction or dimerization of the free radical becomes thermodynamically reversible, rendering the radical less susceptible to deactivation and more persistent. The RSE of cyclohexadienyl radicals, generated from an anisole radical cation and an imidazole, were estimated to be 28.7–31.0 kcal mol^−1^. Meanwhile, the RSE of radical anion species derived from redox-active substrates demonstrated a higher value (for the evaluation of other radicals, see the ESI†). NHC-stabilized acyl radicals (RSE > 60 kcal mol^−1^)^16*a*^ exhibit notable stability, with their X-ray structures and EPR spectra previously reported.^17^ From a kinetic perspective, cyclohexadienyl radicals are prone to dimerization, making them transient.^18^ In contrast, radicals derived from imidazolium ester 2a suppress dimerization due to steric effects as well as electronic effects, making them persistent. The persistence of the cyanopyridine radical anion is also likely attributed to Coulomb repulsion, which prevents homocoupling. These insights support the plausibility of a radical–radical coupling mechanism in the reaction pathway, governed by the persistent radical effect.^19^

To further explore the intrinsic regioselectivity of 1a, a Friedel–Crafts (FC) reaction was conducted (Fig. 2G). Under standard FC reaction conditions, the acyl group was selectively introduced at the C4 position. Notably, a significant amount of decomposition products derived from the C4-substituted product was observed, while no C5-substituted products were detected. This revealed that this method demonstrates regioselectivity complementary to conventional FC reactions, even for substrate 1a. Similarly, for substrate 1b, the substitution at the C4 position using the current method is also complementary to the regioselectivity observed at the C1 position in FC reactions.^5*b*^

**Fig. 3:**
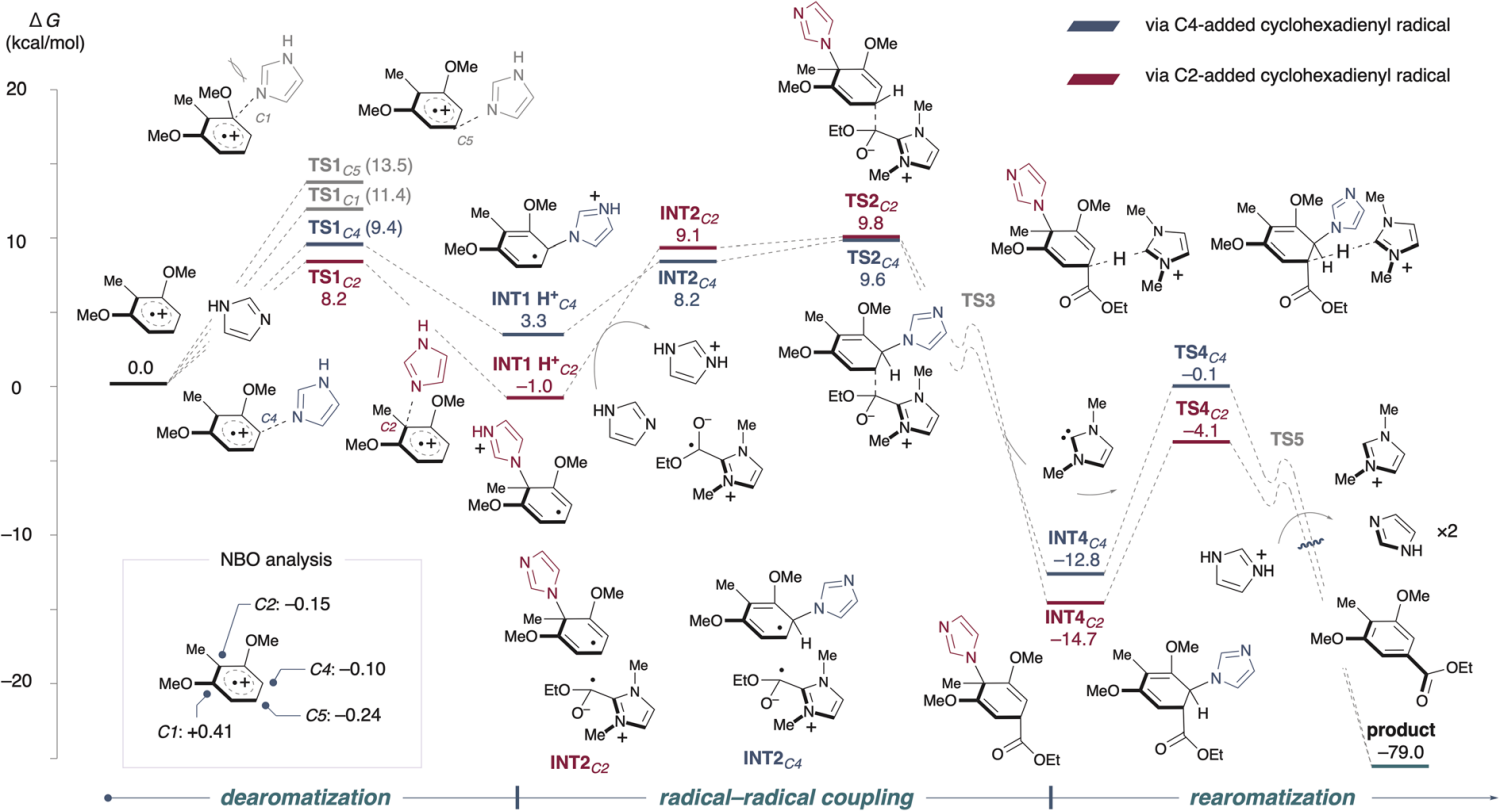
Energy profiles for the proposed pathway. The unit of Gibbs free energies (Δ*G*) is kcal mol^−1^. Density functional theory calculations were carried out at the (U)M06-2X/6-31+G(d,p)_SMD (dichloromethane) level of theory. The charge distribution of the radical cation was calculated at the UωB97X-D/def2-TZVPP_SMD (dichloromethane) level of theory. The reaction pathway *via* C4-added cyclohexadienyl radicals (blue line) and *via* C2-added cyclohexadienyl radicals (red line).

Using the alkoxycarbonylation as a model, we calculated the energy barriers for each step from the arene radical cation to the final product (see Fig. 3 and the ESI† for a detailed whole pathway). NBO analysis revealed that, in the radical cation state of dimethoxytoluene, the C2 and C4 positions exhibited a higher cationic character compared to the C5 position, making nucleophilic addition at C2 or C4 more favorable.^5,20^ Despite the C1 position of the methoxy group having the highest positive charge, the addition step was found to be significantly high (+11.4 kcal mol^−1^), rendering it kinetically unfavorable due to steric hindrance. For imidazole addition (TS1), the activation barriers were 8.2 kcal mol^−1^ at C2 and 9.4 kcal mol^−1^ at C4. Nucleophilic addition to C5, which has less positive charge, also showed a higher energy barrier. Deprotonation then occurred *via* the abundant imidazole base present in the reaction system, and the barrier for radical–radical coupling, starting from the neutral radical pair INT2, was calculated to be TS2. Following this, carbene elimination proceeded with relatively low activation energy (TS3), leading to the formation of the stable intermediate INT4. Subsequently, deprotonation by carbene occurred at TS4, generating an enolate intermediate. Finally, rearomatization was achieved through an azole elimination, resulting in the irreversible formation of the desired product.

## Conclusions

In summary, we have developed a distal C–H functionalization of alkoxyarenes through organic photoredox-catalyzed radical–radical coupling. Diverse functional groups including sp^2^ carbon-based groups such as alkoxycarbonyl and pyridyl, as well as heteroatom-containing groups like sulfonyl, and alkyl groups were introduced to the aromatic ring with complete regioselectivity.

## Data availability

The data supporting this article have been included as part of the ESI.†

## Author contributions

Y. G. and H. O. designed, performed and analysed the experiments. Y. G. and H. O. co-wrote the manuscript.

## Conflicts of interest

There are no conflicts to declare.

## Supplementary Material

SC-016-D4SC08407A-s001
