# Supplementary material for: Distal C–H functionalization of alkoxyarenes through organic photoredox-catalyzed radical–radical coupling
Source: Chem Sci. 2025 Jan 29;16(10):4320–7. doi: 10.1039/d4sc08407a (PMC11792067; doi:10.1039/d4sc08407a)

Supplementary Information (SI) for Chemical Science.
This journal is © The Royal Society of Chemistry 2025

Distal C–H Functionalization of Alkoxyarenes through Organic Photoredox-Catalyzed Radical–Radical Coupling

Yamato Goto and Hirohisa Ohmiya*

Institute for Chemical Research, Kyoto University, Gokasho, Uji, Kyoto 611-0011, Japan

*E-mail: Hirohisa Ohmiya: ohmiya@scl.kyoto-u.ac.jp

■ Supplementary Methods ■

1. Instrumentation and Chemicals S2–S4
2. Detailed Optimization Conditions for reaction conditions S5–S10
   2-1. Alkoxycarbonylation
   2-2. Heteroarylation
   2-3. Sulfonylation
   2-4. Alkylation
3. Preparation of Starting Materials S11–S23
4. General Procedures for Distal C–H Functionalization of Alkoxyarenes S24–S26
   4-1. Alkoxycarbonylation
   4-2. Sulfonylation
   4-3. Heteroarylation
   4-4. Alkylation
5. Characterization Data for Products S27–S39
6. Synthetic Procedures for the Larger Scale Reaction S40
7. Mechanistic Study S41–S46
   7-1. TEMPO Trapping
   7-2. Reaction Under Standard Conditions with Aryl Bromide
   7-3. Monitoring of the Reaction Intermediates
   7-4. Light Intensity-Dependent Product Yield Over Time
   7-5. Intrinsic Regioselectivity Observed for **1a** and **1b** in Ttypical Friedel–Crafts Reactions
8. Unsuccessful Examples S47
9. Computational Study S48–S135

■ Supplementary References ■ S136–S137

■ NMR Spectra ■ S138–S243

1. Instrumentation and Chemicals

NMR spectra were recorded on Bruker AVANCE NEO 400N spectrometer, operating at 400 MHz for ^{1}H NMR, 100.6 MHz for ^{13}C NMR, 376.5 MHz for ^{19}F NMR. Chloroform-d_1 ($CDCl_3$) containing 0.03% tetramethylsilane (TMS, δ 0.00 ppm in $CDCl_3$, 99.80%D, Eurisotop) and dimethyl sulfoxide-d_6 (DMSO-d_6, 99.9%D, Wako) were used as solvents for NMR measurements at ambient temperature. Chemical shifts (δ) for ^{1}H NMR are given in parts per million (ppm) relative to TMS (δ 0.00 ppm in $CDCl_3$) or residual dimethyl sulfoxide (δ 2.50 ppm). Chemical shifts (δ) for ^{13}C NMR are given in ppm relative to $CDCl_3$ (δ 77.0 ppm), or residual dimethyl sulfoxide (δ 40.0 ppm). The abbreviations s, d, t, q, br s, and m signify singlet, doublet, triplet, quartet, broad singlet, and multiplet, respectively.

ESI-MASS was measured with impact II (Bruker).

TLC analyses were performed on commercial glass plates bearing 0.25-mm layer of Merck Silica gel 60F254. Silica gel (Wakosil® 60, 64~210 µm) was used for column chromatography. PLC plate (Silicagel 70 F254-Wako) was used for preparative thin-layer chromatography.

LaboACE LC-5060 (for Gel Permeation Chromatography) were used for purification.

IR spectra were measured with a Thermo Scientific iD7 ATR Accessory for the Thermo Scientific Nicolet iS5 FT-IR Spectrometer.

Melting points were measured on a Stanford Research Systems MPA100.

Reaction set-up and materials: Kessil PR-160 440 nm and Kessil PR-160 390 nm (highest blue and intensity setting) was used as a light source. Unless otherwise noted, the reaction was conducted under photoirradiation at 100% intensity. TEKNOS MG9 was used as a fan.

All reactions unless otherwise noted were carried out under nitrogen atmosphere. Materials were obtained from commercial suppliers listed as below and stored under nitrogen, and used as received or prepared according to standard procedures unless otherwise noted. The photoredox catalysts, and substrates were listed in Supplementary Fig. 1. Some catalysts and substrates were prepared according to the literatures (**PC-1** [1], **PC-2** [2], **PC-3** [3], **PC-5** [4], **1g** [5], **4e** [6], **6b** [7]).

Fujifilm Wako Pure Chemical Co.

Cesium Carbonate, Sodium Hydroxide, Toluene anhydrous, N,N-Dimethylacetamide Anhydrous, N,N-Dimethylformamide, Acetonitrile Anhydrous, Diethyl Ether Anhydrous, Dichloromethane Anhydrous, Acetone Anhydrous, Triethylamine.

Tokyo Chemical Industry Co., Ltd.

3-Amino-4-chlorobenzotrifluoride, 3-Aminobenzotrifluoride, (±)-BINAP, Sodium *tert*-Butoxide, Tri-*tert*-butylphosphonium Tetrafluoroborate, 9,10-Dicyanoanthracene, Imidazole, Pyrazole, 2-Methylimidazole, Benzimidazole, 1,2,4-Triazole, 2-Methoxynaphthalene, 2-Naphthol, 2-Methylresorcinol, Iodomethane, 1-Bromo-3-chloropropane, Methyl 11-Bromoundecanoate, 1,3-Dimethoxybenzene, Anisole, 4-(Trifluoromethyl)benzyl Bromide, Sodium Hydride (60%, dispersion in Paraffin Liquid), 2-Iodopropane, Trichloroacetyl Chloride, 1-Methylimidazole, Methyl

Trifluoromethanesulfonate, 3,3,3-Trifluoro-1-propanol, 3-Buten-1-ol, 3-Hexyn-1-ol, β-Citronellol, 3-Phenyl-1-propanol, Cyclobutanol, Ethyl Chloroformate, 4-Chlorobenzenesulfonyl Chloride, 4-(Trifluoromethyl)benzenesulfonyl Chloride, 4-Cyanobenzenesulfonyl Chloride, *p*-Tolualdehyde, Methanesulfonamide, Cyclopropanesulfonamide, Tetraethyl Orthosilicate, 4-Cyanopyridine, Dimethylcarbamoyl Chloride, Ethyl Benzoylformate, 2,2,6,6-Tetramethylpiperidine 1-Oxyl Free Radical, Ethyl 4-Bromobenzoate, 2,4,6-Triphenylpyrylium Tetrafluoroborate, Aluminum(III) Chloride.

<u>Kanto Chemical Co., Inc.</u>

Trimethylsilyl Cyanide.

<u>Merk Sigma–Aldrich Japan Inc.</u>

Palladium(II) Acetate.

<u>Nacalai Tesque Inc.</u>

Potassium Carbonate, Benzaldehyde, Chloromethyl Methyl Ether, N-Methylmorpholine.

<u>Angene Int., Ltd.</u>

2,6-Dimethoxytoluene, 1,2,3-Triazole, 4-Cyanoimidazole, 4-Trifluoromethylimidazole, 3,5-Bis(trifluoromethyl)benzene-1-sulfonyl chloride.

<u>Oakwood Products Inc.</u>

Isoquinoline N-oxide.

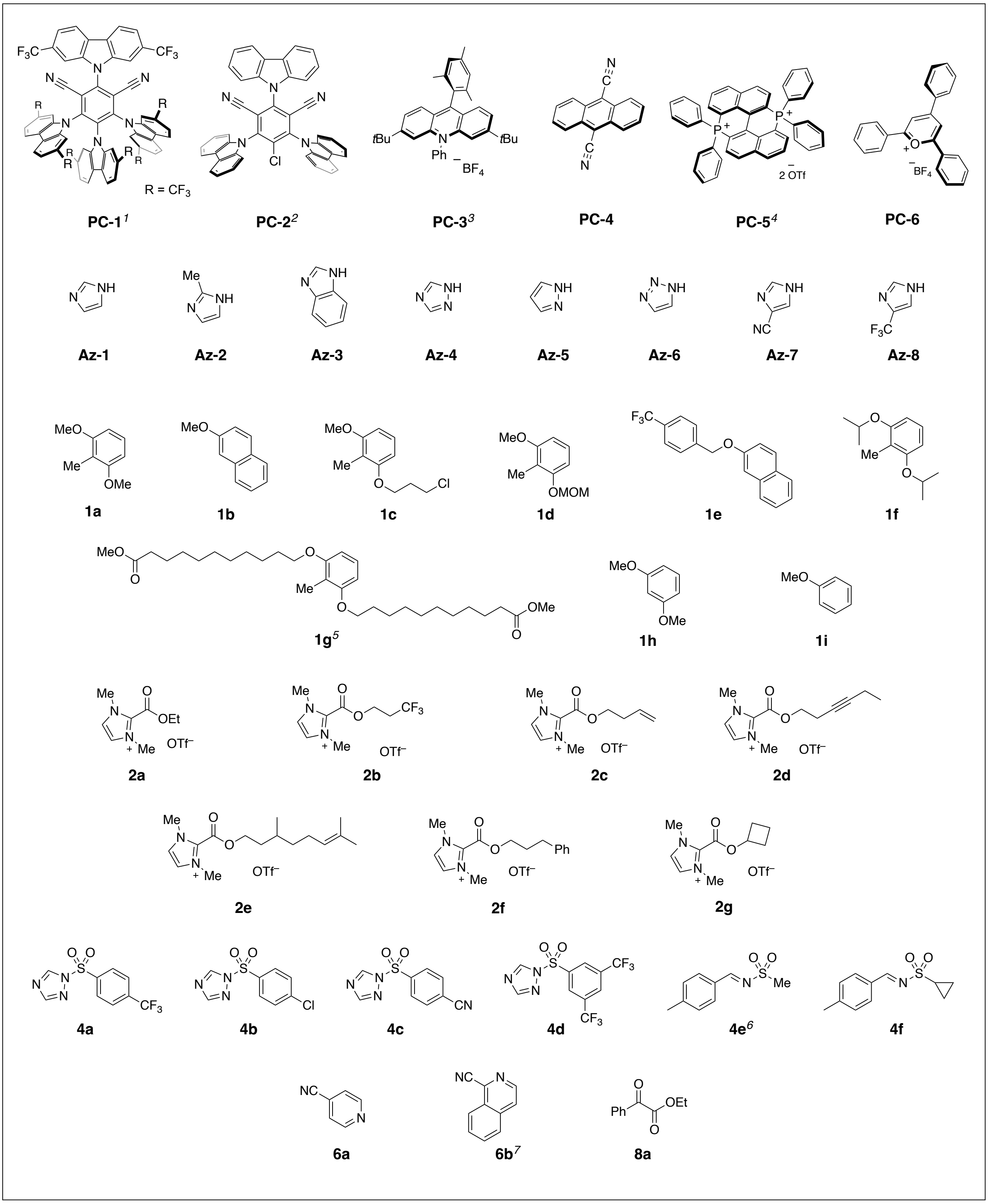

Supplementary Fig. 1. Photoredox catalysts, azoles, arenes and substrates.

2-1. Detailed Optimization Conditions for Alkoxycarbonylation Reaction

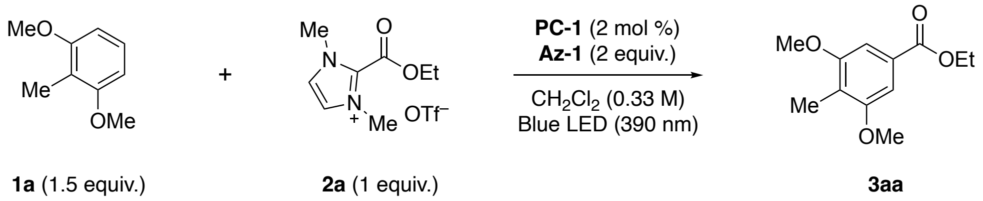

Entry	Deviation from the standard conditions	yield of **3aa** (%)[a]
1	none	78 (69)[b]
2	**PC-2** instead of **PC-1**	2
3	**PC-3** instead of **PC-1**	0
4	**PC-4** instead of **PC-1**	0
5	**Az-2** instead of **Az-1**	11
6	**Az-3** instead of **Az-1**	6
7	**Az-4** instead of **Az -1**	0
8	**Az-5** instead of **Az-1**	0
9	w/o **PC-1**	2
10	w/o **Az-1**	0
11	under dark	0
12	440 nm light source	0
13	**1a** (1 equiv.), **2a** (1.2 equiv.)	52
14	CH_2Cl_2 (0.2 M)	66
15	CH_2Cl_2 (0.1 M)	64
16	**PC-5** instead of **PC-1**	1
17	**PC-6** instead of **PC-1**	1
18	**Az-6** instead of **Az-1**	0
19	**Az-7** instead of **Az-1**	0
20	**Az-1** (1 equiv.)	29
21	**Az-1** (50 mol %)	14

Supplementary Table 1 | [a] Reaction was carried out with 2,6-dimethoxytoluene **1a** (0.15 mmol), imidazolium ester **2a** (0.10 mmol), **Az-1** (0.20 mmol) and **PC-1** (0.002 mmol) in CH_2Cl_2 (0.3 mL) under 390 nm (Kessil lamp) irradiation at ambient temperature for 16 h. The product yield was determined by ^{1}H-NMR. [b] Isolated yield.

2-2. Detailed Optimization Conditions for Heteroarylation Reaction

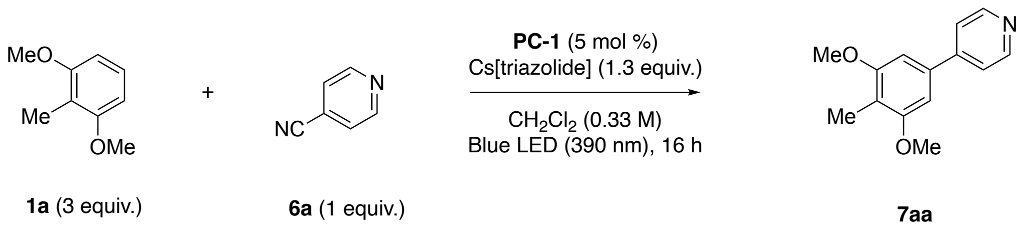

Entry	Deviation from the standard conditions	yield of **7aa** (%)[a]
1	none	6
2	**PC-3** instead of **PC-1**	0
3	440 nm light source	0

Supplementary Table 2 | [a] Reaction was carried out with 2,6-dimethoxytoluene **1a** (0.30 mmol), 4-cyanopyridine **6a** (0.10 mmol), Cs[triazolide] (0.13 mmol)and **PC-1** (0.005 mmol) in CH_2Cl_2 (0.30 mL) under 390 nm (Kessil lamp) irradiation at ambient temperature for 16 h. The product yield was determined by ^{1}H-NMR.

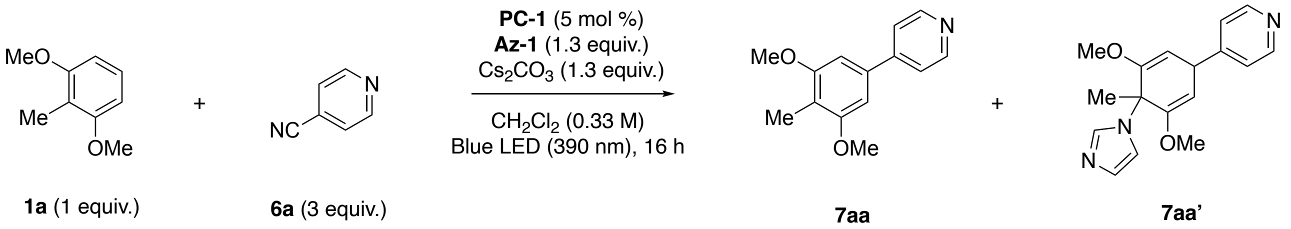

Entry	Deviation from the standard conditions	yield of **7aa** (%)[a]	yield of **7aa'** (%)[a]
1	none	34-43	
2	w/o **Az-1**	0	
3	**Az-2** instead of **Az-1**	18[b]	
4	**Az-3** instead of **Az-1**	31[b]	
5	**Az-5** instead of **Az-1**	2	
6	**Az-8** instead of **Az-1**	9[b]	
7	1,2-dichloroethane instead instead of CH_2Cl_2	35[b]	12
8	$CHCl_3$ instead of CH_2Cl_2	25[b]	13
9	toluene instead of CH_2Cl_2	13[b]	0
10	CH_3CN instead of CH_2Cl_2	0[b]	0

Supplementary Table 3 | [a] Reaction was carried out with 2,6-dimethoxytoluene **1a** (0.10 mmol), 4-cyanopyridine **6a** (0.30 mmol), **Az-1** (0.13 mmol)and **PC-1** (0.005 mmol) in CH_2Cl_2 (0.30 mL) under 390 nm (Kessil lamp) irradiation at ambient temperature for 16 h. The product yield was determined by ^{1}H-NMR. [b] 18 h.

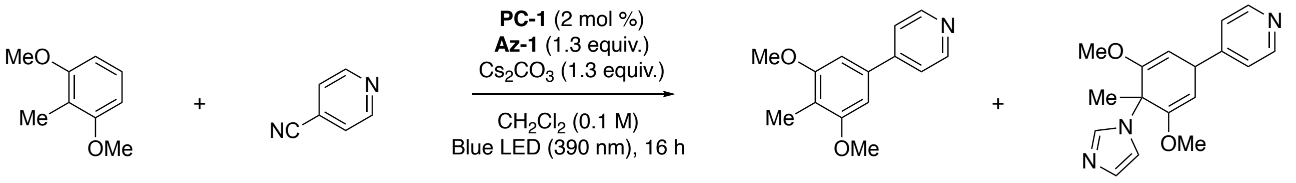

1a (1 equiv.) **6a** (3 equiv.) **7aa** **7aa'**

Entry	Deviation from the standard conditions	yield of **7aa** (%)[a]	yield of **7aa'** (%)[a]
1	none	21	35
2	**Az-1** (2 equiv.)	24	41
3	**Az-1** (2 equiv.), Cs_2CO_3 (1 equiv.)	29	42
4	**Az-1** (2 equiv.), Cs_2CO_3 (1 equiv.)	71 [b]	0

Supplementary Table 4 | [a] Reaction was carried out with 2,6-dimethoxytoluene **1a** (0.10 mmol), 4-cyanopyridine **6a** (0.30 mmol), **Az-1** (0.13 mmol), Cs_2CO_3 (0.13 mmol)and **PC-1** (0.002 mmol) in CH_2Cl_2 (1.0 mL) under 390 nm (Kessil lamp) irradiation at ambient temperature for 16 h. The product yield was determined by ^{1}H-NMR. [b] After photo irradiation, the reaction mixture was heated to 60°C for 4 h in MeCN.

2-3. Detailed Optimization Conditions for Sulfonylation Reaction

♦ Importance of the Imine Formation

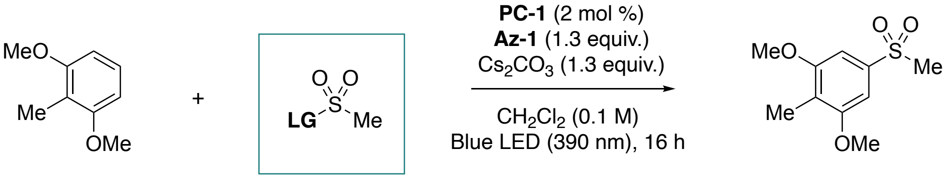

1a (1 equiv.) **5ae**

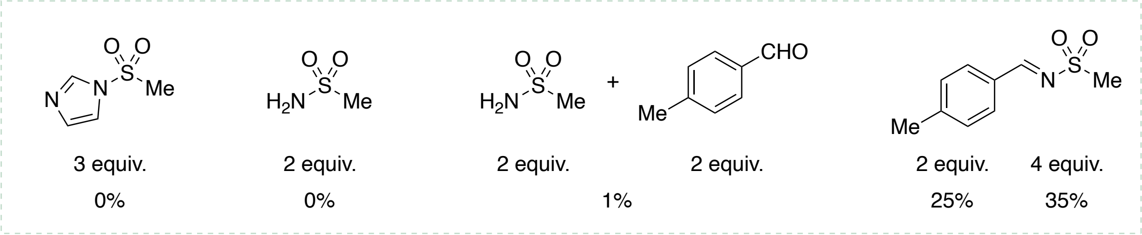

Supplementary Table 5 | [a] Reaction was carried out with 2,6-dimethoxytoluene **1a** (0.10 mmol), sulfonylation reagent (2–4 equiv.), **Az-1** (0.13 mmol), Cs_2CO_3 (0.13 mmol) and **PC-1** (0.002 mmol) in CH_2Cl_2 (1.0 mL) under 390 nm (Kessil lamp) irradiation at ambient temperature for 16 h. The product yield was determined by ^{1}H-NMR.

♦ Control Experiments

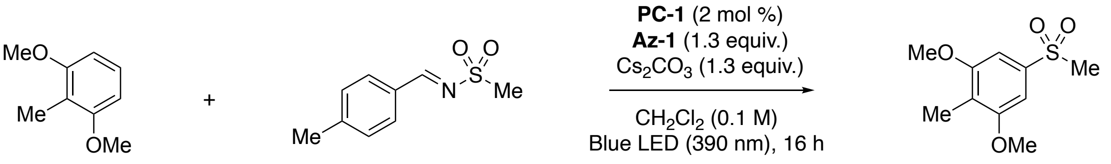

1a (1 equiv.) **4e** (2 equiv.) **5ae**

Entry	Deviation from the standard conditions	NMR yield of **5ae** (%)[a]
1	none	25
2	w/o **Az-1**	0
3	440 nm light source	2

Supplementary Table 6 | [a] Reaction was carried out with 2,6-dimethoxytoluene **1a** (0.10 mmol), sulfonylimine **4e** (0.2 mmol), **Az-1** (0.13 mmol), Cs_2CO_3 (0.13 mmol) and **PC-1** (0.002 mmol) in CH_2Cl_2 (1.0 mL) under 390 nm (Kessil lamp) irradiation at ambient temperature for 16 h. The product yield was determined by ^{1}H-NMR.

♦ Screening of Leaving Groups (Methanesulfonylation)

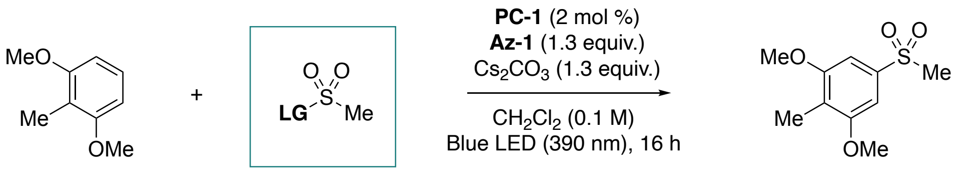

1a 2–4 equiv. **5ae**

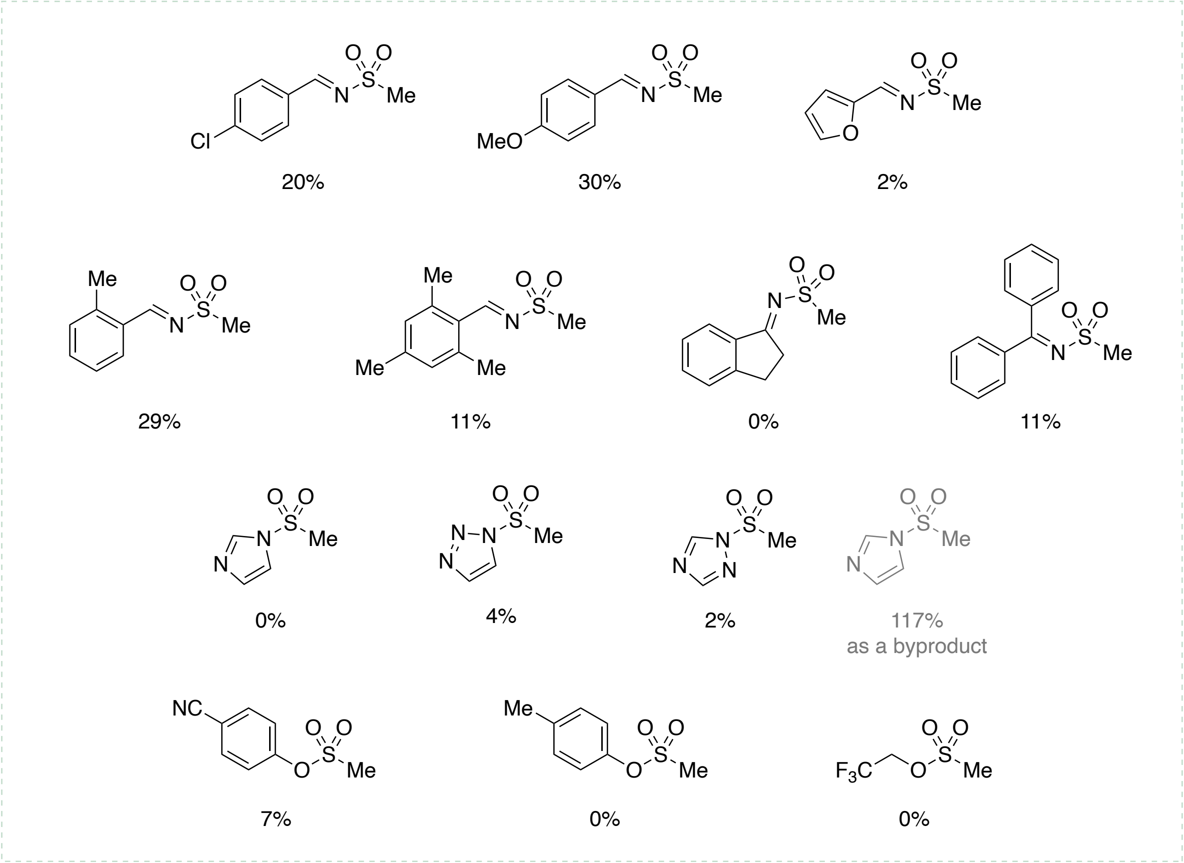

Supplementary Table 7 | [a] Reaction was carried out with 2,6-dimethoxytoluene **1a** (0.10 mmol), sulfonylation reagent (2–4 equiv.), **Az-1** (0.13 mmol), Cs_2CO_3 (0.13 mmol) and **PC-1** (0.002 mmol) in CH_2Cl_2 (1.0 mL) under 390 nm (Kessil lamp) irradiation at ambient temperature for 16 h. The product yield was determined by ^{1}H-NMR.

- Screening of Leaving Groups (*p*-Toluenesulfonylation)

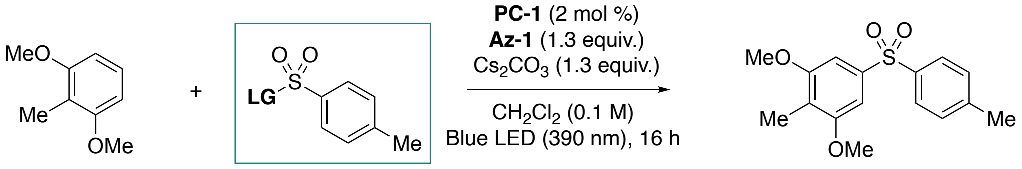

1a 2–4 equiv.

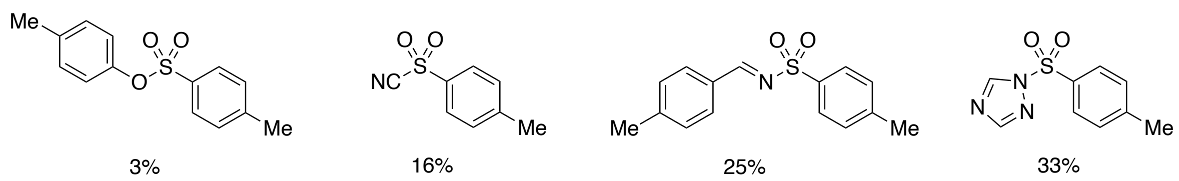

Supplementary Table 8 | [a] Reaction was carried out with 2,6-dimethoxytoluene **1a** (0.10 mmol), sulfonylation reagent (2–4 equiv.), **Az-1** (0.13 mmol), Cs_2CO_3 (0.13 mmol) and **PC-1** (0.002 mmol) in CH_2Cl_2 (1.0 mL) under 390 nm (Kessil lamp) irradiation at ambient temperature for 16 h. The product yield was determined by ^{1}H-NMR.

2-4. Detailed Optimization Conditions for Alkylation Reaction

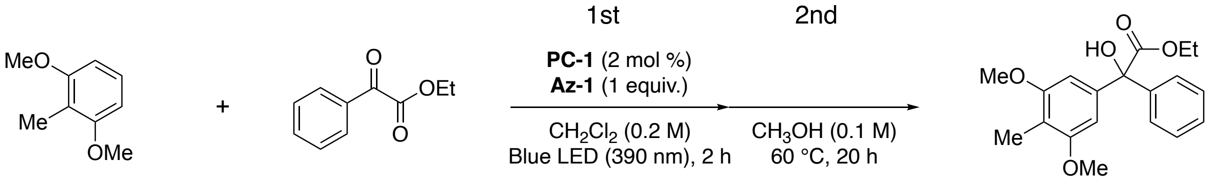

1a (1 equiv.) **8a** (1.5 equiv.) **9aa**

Entry	Deviation from the standard conditions	NMR yield of **9aa** (%)[a]
1	none	11
2	only 1st step	0
3	arene (3 equiv.), ketoester (1 equiv.)	18

Supplementary Table 9 | [a] Reaction was carried out with 2,6-dimethoxytoluene **1a** (0.10 mmol), ketoester **8a** (0.15 mmol), **Az-1** (0.10 mmol) and **PC-1** (0.002 mmol) in CH_2Cl_2 (0.5 mL) under 390 nm (Kessil lamp) irradiation at ambient temperature for 2 h. After photo irradiation, the reaction mixture was heated to 60 ℃ and stirred for 20 h in methanol. The product yield was determined by ^{1}H-NMR.

3. Preparation of Starting Materials

♦ Preparation of Arenes

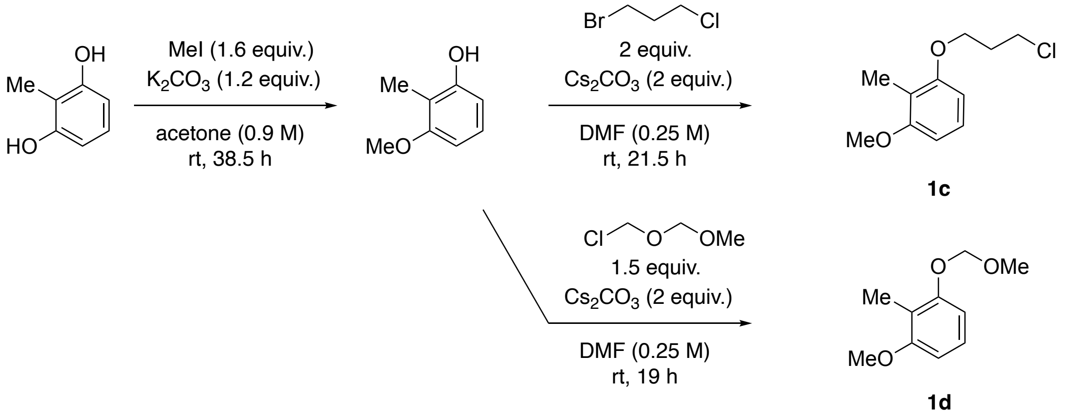

3-Methoxy-2-methylphenol

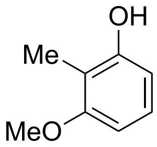

To an oven-dried 200 mL flask was charged with 2-methylresorcinol (5.6 g, 45.5 mmol, 1.0 equiv.), K_2CO_3 (7.5 g, 54 mmol, 1.2 equiv.) and iodomethane (4.4 mL, 71 mmol, 1.6 equiv.) in anhydrous acetone (50 mL) under nitrogen. The solution was stirred at room temperature for 38.5 h. AcOEt (100 mL) was added to the reaction mixture and the organic layer was washed with water (50 mL, 3 times). The organic layer was dried with Na_2SO_4 and then filtrated. The organic layer was concentrated in vacuo and the product was purified by flash chromatography on silica gel (100:0–90:10, hexane/AcOEt) to give 3-methoxy-2-methylphenol as off-white solid (2.2 g, 15.6 mmol, 34% isolated yield).
The ^{1}H NMR spectra data of the product was consistent with the literature.[8]

1-(3-Chloropropoxy)-3-methoxy-2-methylbenzene (1c)

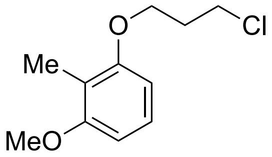

To an oven-dried screw-top 20 mL vial with a stirring bar was added 3-methoxy-2-methylphenol (276 mg, 2.0 mmol, 1.0 equiv.), Cs_2CO_3 (652 mg, 2 mmol, 1.0 equiv.) and 1-bromo-3-chloropropane (394 μL, 4.0 mmol, 2.0 equiv.) in anhydrous DMF (8 mL) under nitrogen. The solution was warmed to 80 °C and stirred for 21.5 h. Et_2O was added to the reaction mixture and washed with water. The organic layer was dried with Na_2SO_4 and filtrated. The organic layer was concentrated in vacuo and

the product was purified by flash chromatography on silica gel (100:0–98:2, hexane/AcOEt) to give 1-(3-chloropropoxy)-3-methoxy-2-methylbenzene **1c** as pale-yellow oil (184 mg, 0.86 mmol, 43% isolated yield, contains 12% inseparable impurity).

IR (neat) 1592.9, 1465.1, 1437.8, 1251.0, 1174.9, 1117.5, 765.6, 708.1 cm^{-1}.

^{1}H NMR (400 MHz, $CDCl_3$) *δ* 7.10 (t, *J* = 8.0 Hz, 1H), 6.54 (d, *J* = 8.0 Hz, 2H), 4.10 (t, *J* = 6.0 Hz, 2H), 3.82 (s, 3H), 3.76 (t, *J* = 6.4 Hz, 2H), 2.24 (tt, *J* = 6.4, 6.0 Hz, 2H), 2.10 (s, 3H).

^{13}C NMR (100.6 MHz, $CDCl_3$) *δ* 158.4, 157.3, 126.2, 114.7, 104.5, 103.6, 64.6, 55.7, 41.7, 32.5, 8.2.

HRMS–ESI (*m/z*): $[M+Na]^+$ calcd for $C_{11}H_{15}ClNaO_2^+$, 237.0653; found, 237.0637.

1-Methoxy-3-(methoxymethoxy)-2-methylbenzene (1d)

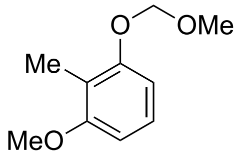

To an oven-dried 50 mL flask with a stirring bar was added 3-methoxy-2-methylphenol (276 mg, 2.0 mmol, 1.0 equiv.) and Cs_2CO_3 (1.3 g, 4.0 mmol, 2.0 equiv.) in anhydrous DMF (8 mL) under nitrogen. The reaction mixture was stirred at room temperature for 30 minutes. To the rection mixture was added chloromethyl methyl ether (226 µL, 3.0 mmol, 1.5 equiv.) slowly. The solution was kept stirring for another 19 h at room temperature. The reaction was quenched with water (20 mL). The mixture was dissolved in Et_2O (25 mL) and washed with water (25mL, 3 times). The organic layer was dried with Na_2SO_4 and filtrated. After concentration in vacuo, the product was purified by flash chromatography on silica gel (100:0–98:2, hexane/AcOEt) to give 1-methoxy-3-(methoxymethoxy)-2-methylbenzene **1d** as pale-yellow oil (246 mg, 1.3 mmol, 68% isolated yield).

IR (neat) 1594.3, 1472.2, 1251.0, 1153.4, 1111.7, 1068.6, 1019.8, 992.5 cm^{-1}.

^{1}H NMR (400 MHz, $CDCl_3$) *δ* 7.09 (dd, *J* = 8.4, 8.4 Hz, 1H), 6.72 (d, *J* = 8.4 Hz, 1H), 6.57 (d, *J* = 8.4 Hz, 1H), 5.19 (s, 2H), 3.82 (s, 3H), 3.48 (s, 3H), 2.13 (s, 3H).

^{13}C NMR (100.6 MHz, $CDCl_3$) *δ* 158.5, 155.9, 126.2, 115.7, 107.2, 104.3, 94.7, 56.0, 55.7, 8.4.

HRMS–ESI (*m/z*): $[M+K]^+$ calcd for $C_{10}H_{14}KO_3^+$, 221.0575; found, 221.0549.

2-((4-(Trifluoromethyl)benzyl)oxy)naphthalene (1e)

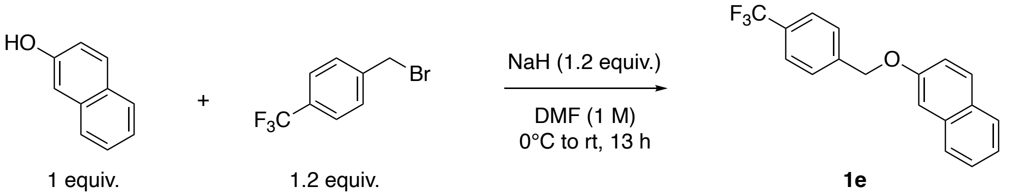

To an oven-dried 30 mL flask with a stirring bar was added 2-naphtol (721 mg, 5.0 mmol, 1.0 equiv.) in anhydrous DMF (5 mL) under nitrogen. NaH (240 mg, 6.0 mmol, 1.2 equiv., 60%, dispersion in paraffin liquid) was added. To the rection mixture was added 4-(trifluoromethyl)benzyl bromide (890 μL, 6.0 mmol, 1.2 equiv.) slowly at 0 °C. The solution was stirred for 13 h at room temperature. The reaction was quenched with water (20 mL). The mixture was dissolved in Et_2O (25 mL) and washed with water (25mL, 3 times). The organic layer was dried with Na_2SO_4 and filtrated. After concentration in vacuo, the product was purified by recrystallization (5:1, hexane/AcOEt, 25 mL) to give **1e** as white solid (269 mg, 0.89 mmol, 18% isolated yield).

M.p. 107–108 °C

IR (neat) 1341.5, 1156.2, 1110.3, 1072.9, 1018.4, 840.3, 818.7, 748.3 cm^{-1}.

^{1}H NMR (400 MHz, $CDCl_3$) *δ* 7.79–7.77 (m, 2H), 7.72 (d, *J* = 7.6 Hz, 1H), 7.66 (d, *J* = 8.0 Hz, 2H), 7.60 (d, *J* = 8.0 Hz, 2H), 7.45 (dd, *J* = 7.6, 7.6 Hz, 1H), 7.35 (dd, *J* = 7.6, 7.6 Hz, 1H), 7.23 (dd, *J* = 9.2, 2.4 Hz, 1H), 7.19 (d, *J* = 2.4 Hz, 1H), 5.24 (s, 2H).

^{13}C NMR (100.6 MHz, $CDCl_3$) *δ* 156.3, 140.9, 134.4, 130.1 (q, $J_{C\text{–}F}$ = 32.6 Hz), 129.6, 129.2, 127.7, 127.4, 126.8, 126.5, 125.6 (q, $J_{C\text{–}F}$ = 3.7 Hz), 124.1 (q, $J_{C\text{–}F}$ = 272.0 Hz), 123.9, 118.8, 107.2, 69.1.

^{19}F NMR (376.5 MHz, $CDCl_3$) *δ* –62.5 (s, 3F).

HRMS–ESI (*m/z*): $[M+H]^+$ calcd for $C_{18}H_{14}F_3O^+$, 303.0991; found, 303.1003.

1,3-Diisopropoxy-2-methylbenzene (1f)

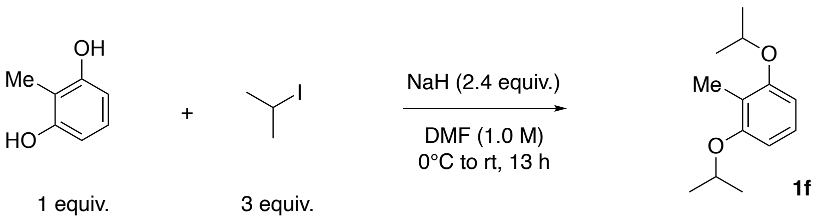

To an oven-dried 50 mL flask with a stirring bar was added 2-methyresorcinol (1.24 g, 10 mmol, 1.0 equiv.) in anhydrous DMF (10 mL) under nitrogen. To the rection mixture was added NaH (960 mg, 24 mmol, 2.4 equiv., 60%, dispersion in paraffin liquid) at 0 °C. The reaction mixture was stirred at room temperature for 10 minutes. To the rection mixture was added 2-iodopropane (2.98 mL, 30 mmol, 3.0 equiv.) slowly at 0 °C. The solution was allowed to warm to room temperature slowly and kept stirring for another 13 h. The reaction was quenched with water and extracted with Et_2O (10 mL, 3 times), then washed with water (10mL, 3 times). The organic layer was dried with Na_2SO_4 and then filtrated. After concentration in vacuo, the product was purified by flash chromatography on silica gel (100:0–98:2, hexane/AcOEt) to give 1,3-diisopropoxy-2-methylbenzene **1f** as colorless oil (483 mg, 2.3 mmol, 23% isolated yield).

IR (neat) 2976.1, 1591.5, 1465.1, 1383.2, 1371.7, 1249.6, 1116.0, 1088.7 cm^{-1}.

^{1}H NMR (400 MHz, $CDCl_3$) *δ* 7.03 (t, *J* = 8.4 Hz, 1H), 6.50 (d, *J* = 8.4 Hz, 2H), 4.47 (septet, *J* = 6.0 Hz, 2H), 2.08 (s, 3H), 1.32 (d, *J* = 6.0 Hz, 12H).

^{13}C NMR (100.6 MHz, $CDCl_3$) δ 157.0, 125.7, 117.6, 106.5, 70.6, 22.3, 8.8.
HRMS–ESI (*m/z*): $[M+Na]^+$ calcd for $C_{13}H_{20}NaO^+$, 215.1406; found, 215.1405.

♦ Preparation of Imidazolium Esters

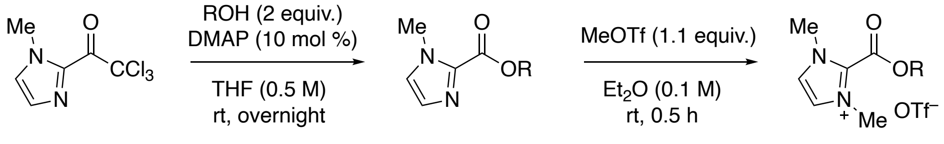

2

2,2,2-Trichloro-1-(1-methyl-1*H*-imidazol-2-yl)ethan-1-one was prepared by the reported procedure.[9]

Alkyl 1-methyl-1*H*-imidazole-2-carboxylate was prepared following a reported procedure.[10]
To an oven-dried, screw-top 20 mL vial containing a stirring bar, 2,2,2-trichloro-1-(1-methyl-1*H*-imidazol-2-yl)ethan-1-one (1.6–2.5 mmol, 1.0 equiv.) and 4-dimethylaminopyridine (10 mol %) were added in anhydrous THF (0.5 M). Alcohol (2.0 equiv.) was then added to the reaction mixture. The mixture was stirred at room temperature overnight. The reaction mixture was then concentrated in vacuo, and the product was purified by flash chromatography on silica gel (hexane/AcOEt) to yield alkyl 1-methyl-1H-imidazole-2-carboxylate.

2-(Alkoxycarbonyl)-1,3-dimethyl-1*H*-imidazol-3-ium trifluoromethanesulfonate **2** was also prepared according to a reported procedure.[10]
To an oven-dried, screw-top 20 mL vial with a stirring bar, alkyl 1-methyl-1*H*-imidazole-2-carboxylate (1.0–1.9 mmol, 1.0 equiv.) was added in anhydrous Et_2O (0.1 M). methyl trifluoromethanesulfonate (1.1 equiv.) was added portion-wise to the reaction mixture, which was then stirred vigorously at room temperature for more than 0.5 h. The resulting white precipitate was filtered and washed with Et_2O. If necessary, the product was purified by recrystallization (DCM/Et_2O).

3,3,3-Trifluoropropyl 1-Methyl-1*H*-imidazole-2-carboxylate

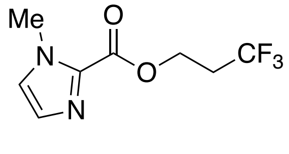

The product was synthesized using 2,2,2-trichloro-1-(1-methyl-1*H*-imidazol-2-yl)ethan-1-one (364 mg, 1.6 mmol) and 3,3,3-trifluoropropan-1-ol (281 μL, 3.2 mmol). The product was purified by flash chromatography on silica gel (100:0–50:50, hexane/AcOEt) to give 3,3,3-trifluoropropyl 1-methyl-1*H*-imidazole-2-carboxylate as colorless oil (225 mg, 1.0 mmol, 63% isolated yield).

IR (neat) 1716.4, 1423.4, 1397.6, 1294.1, 1248.2, 1156.2, 1126.1, 779.9 cm^{-1}.

^{1}**H NMR** (400 MHz, $CDCl_3$) δ 7.17 (d, J = 0.8 Hz, 1H), 7.06 (d, J = 0.8 Hz, 1H), 4.57 (t, J = 6.8 Hz, 2H), 4.02 (s, 3H), 2.67 (qt, J = 10.4, 6.8 Hz, 2H).

^{13}**C NMR** (100.6 MHz, $CDCl_3$) δ 158.6, 135.9, 129.8, 126.6, 125.6 (q, J_{C-F} = 276.3 Hz), 57.8 (q, J_{C-F} = 3.7 Hz), 35.9, 33.4 (q, J_{C-F} = 29.1 Hz).

^{19}**F NMR** (376.5 MHz, $CDCl_3$) δ –65.0 (t, J_{F-H} = 10.4 Hz, 3F).

HRMS–ESI (*m/z*): [M+H]$^+$ calcd for $C_8H_{10}F_3N_2O_2^+$, 223.0689; found, 223.0678.

3-Buten-1-yl 1-Methyl-1*H*-imidazole-2-carboxylate

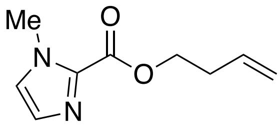

The product was synthesized using 2,2,2-trichloro-1-(1-methyl-1*H*-imidazol-2-yl)ethan-1-one (569 mg, 2.5 mmol) and 3-buten-1-ol (424 μL, 5.0 mmol). The product was purified by flash chromatography on silica gel (100:0–50:50, hexane/AcOEt) to give 3-buten-1-yl 1-methyl-1*H*-imidazole-2-carboxylate as colorless oil (407 mg, 2.3 mmol, 90% isolated yield).

IR (neat) 1709.2, 1417.7, 1255.4, 1156.2, 1123.2, 1051.4, 919.3, 781.4 cm^{-1}.

^{1}**H NMR** (400 MHz, $CDCl_3$) δ 7.15 (s, 1H), 7.04 (s, 1H), 5.86 (ddt, J = 17.2, 10.4, 6.8 Hz, 1H), 5.17 (dd, J = 17.2, 1.2 Hz, 1H), 5.11 (dd, J = 10.4, 1.2 Hz, 1H), 4.40 (t, J = 6.8 Hz, 2H), 4.01 (s, 3H), 2.57 (td, J = 6.8, 6.8 Hz, 2H).

^{13}**C NMR** (100.6 MHz, $CDCl_3$) δ 159.1, 136.5, 133.5, 129.4, 126.2, 117.5, 64.3, 35.8, 33.0.

HRMS–ESI (*m/z*): [M+H]$^+$ calcd for $C_9H_{13}N_2O_2^+$, 181.0972; found, 181.0965.

3-Hexyn-1-yl 1-Methyl-1*H*-imidazole-2-carboxylate

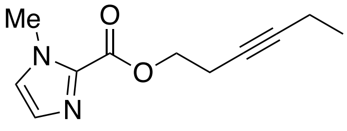

The product was synthesized using 2,2,2-trichloro-1-(1-methyl-1*H*-imidazol-2-yl)ethan-1-one (569 mg, 2.5 mmol) and 3-hexyn-1-ol (545 μL, 5.0 mmol). The product was purified by flash chromatography on silica gel (50:50, hexane/AcOEt) to give 3-hexyn-1-yl 1-methyl-1*H*-imidazole-2-carboxylate as white solid (412 mg, 2.0 mmol, 80% isolated yield).

M.p. 78–80 °C

IR (neat) 1712.1, 1420.5, 1411.9, 1256.8, 1144.8, 1131.8, 925.0, 802.9 cm^{-1}.

^{1}H NMR (400 MHz, $CDCl_3$) δ 7.15 (d, J = 0.8 Hz, 1H), 7.04 (d, J = 0.8 Hz, 1H), 4.41 (t, J = 7.6 Hz, 2H), 4.02 (s, 3H), 2.67 (tt, J = 7.6, 2.4 Hz, 2H), 2.15 (qt, J = 7.2, 2.4 Hz, 2H), 1.11 (t, J = 7.2 Hz, 3H).

^{13}C NMR (100.6 MHz, $CDCl_3$) δ 158.9, 136.5, 129.6, 126.3, 83.8, 74.3, 63.5, 35.9, 19.2, 14.0, 12.4.

HRMS–ESI (*m/z*): $[M+H]^+$ calcd for $C_{11}H_{15}N_2O_2^+$, 207.1128; found, 207.1122.

3,7-Dimethyloct-6-en-1-yl 1-Methyl-1*H*-imidazole-2-carboxylate

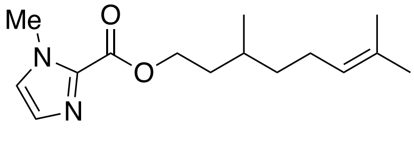

The product was synthesized using 2,2,2-trichloro-1-(1-methyl-1*H*-imidazol-2-yl)ethan-1-one (569 mg, 2.5 mmol) and 3,7-dimethyloct-6-en-1-ol (909 µL, 5.0 mmol). The product was purified by flash chromatography on silica gel (100:0–50:50, hexane/AcOEt) to give 3,7-dimethyloct-6-en-1-yl 1-methyl-1*H*-imidazole-2-carboxylate as colorless oil (566 mg, 2.1 mmol, 86% isolated yield).

IR (neat) 1709.2, 1420.5, 1377.4, 1291.3, 1258.2, 1126.1, 1050.0, 919.3 cm^{-1}.

^{1}H NMR (400 MHz, $CDCl_3$) δ 7.14 (s, 1H), 7.03 (s, 1H), 5.09 (m, 1H), 4.43–4.32 (m, 2H), 4.01 (s, 3H), 2.07–1.91 (m, 2H), 1.85 (m, 1H), 1.72–1.58 (m, 8H), 1.39 (m, 1H), 1.22 (m, 1H), 0.96 (d, J = 6.4 Hz, 3H).

^{13}C NMR (100.6 MHz, $CDCl_3$) δ 159.4, 136.8, 131.3, 129.4, 126.1, 124.5, 64.0, 37.0, 35.8, 35.4, 29.5, 25.7, 25.3, 19.4, 17.6.

HRMS–ESI (*m/z*): $[M+H]^+$ calcd for $C_{15}H_{25}N_2O_2^+$, 265.1911; found, 265.1910.

3-Phenylpropyl 1-Methyl-1*H*-imidazole-2-carboxylate

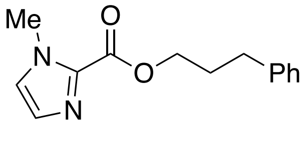

The product was synthesized using 2,2,2-trichloro-1-(1-methyl-1*H*-imidazol-2-yl)ethan-1-one (569 mg, 2.5 mmol) and 3-phenylpropan-1-ol (681 µL, 5.0 mmol). The product was purified by flash chromatography on silica gel (100:0–20:80, hexane/AcOEt) to give 3-phenylpropyl 1-methyl-1*H*-imidazole-2-carboxylate as pale-yellow oil (370 mg, 1.5 mmol, 61% isolated yield).

IR (neat) 1709.2, 1455.0, 1419.1, 1258.2, 1127.5, 781.4, 749.8, 700.9 cm^{-1}.

^{1}H NMR (400 MHz, $CDCl_3$) δ 7.28 (dd, J = 7.2, 7.2 Hz, 2H), 7.22–7.17 (m, 3H), 7.16 (d, J = 0.8 Hz, 1H), 7.04 (d, J = 0.8 Hz, 1H), 4.36 (t, J = 6.8 Hz, 2H), 4.01 (s, 3H), 2.78 (t, J = 8.0 Hz, 2H), 2.15 (tt, J = 8.0, 6.8 Hz, 2H).

^{13}C NMR (100.6 MHz, $CDCl_3$) δ 159.3, 141.0, 136.6, 129.4, 128.4 (×2C), 126.3, 125.9, 64.7, 35.8, 32.1, 30.0.

HRMS–ESI (*m/z*): $[M+H]^+$ calcd for $C_{14}H_{17}N_2O_2^+$, 245.1285; found, 245.1281.

Cyclobutyl 1-Methyl-1*H*-imidazole-2-carboxylate

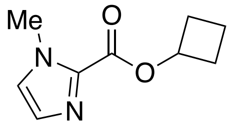

The product was synthesized using 2,2,2-trichloro-1-(1-methyl-1*H*-imidazol-2-yl)ethan-1-one (364 mg, 1.6 mmol) and cyclobutanol (251 μL, 3.2 mmol). The product was purified by flash chromatography on silica gel (100:0–50:50, hexane/AcOEt) to give cyclobutyl 1-methyl-1*H*-imidazole-2-carboxylate as colorless oil (175 mg, 0.97 mmol, 61% isolated yield).

IR (neat) 1704.9, 1478.0, 1413.4, 1291.3, 1258.2, 1154.8, 1124.6, 1057.1 cm^{-1}.

^{1}H NMR (400 MHz, $CDCl_3$) δ 7.14 (s, 1H), 7.03 (s, 1H), 5.20 (tt, J = 8.0, 8.0 Hz, 1H), 4.00 (s, 3H), 2.48–2.41 (m, 2H), 2.38–2.28 (m, 2H), 1.87 (m, 1H), 1.68 (m, 1H).

^{13}C NMR (100.6 MHz, $CDCl_3$) δ 158.7, 136.6, 129.4, 126.3, 69.8, 35.8, 30.3, 13.6.

HRMS–ESI (*m/z*): $[M+H]^+$ calcd for $C_9H_{13}N_2O_2^+$, 181.0972; found, 181.0957.

1,3-Dimethyl-2-((3,3,3-trifluoropropoxy)carbonyl)-1*H*-imidazol-3-ium Trifluoromethanesulfonate (2b)

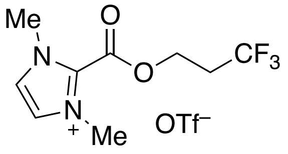

The product **2b** was synthesized from 3,3,3-trifluoropropyl 1-methyl-1*H*-imidazole-2-carboxylate (222 mg, 1.0 mmol). The product was purified by recrystallization (DCM/Et_2O) to give **2b** as white solid (259 mg, 0.67 mmol, 67% isolated yield).

M.p. 140–142 °C

IR (neat) 1742.3, 1449.3, 1304.2, 1253.9, 1160.6, 1139.0, 1031.3 cm^{-1}.

^{1}H NMR (400 MHz, DMSO-d_6) δ 7.96 (s, 2H), 4.61 (t, J = 6.0 Hz, 2H), 4.05 (s, 6H), 2.92 (qt, J = 11.6, 6.0 Hz, 2H).

^{13}C NMR (100.6 MHz, DMSO-d_6) δ 154.0, 132.9, 127.0 (q, J_{C-F} = 276.7 Hz), 126.6, 121.2 (q, J_{C-F} = 322.1 Hz), 60.6 (q, J_{C-F} = 3.7 Hz), 39.0, 32.4 (q, J_{C-F} = 28.7 Hz).

^{19}F NMR (376.5 MHz, DMSO-d_6) δ –63.5 (t, J_{F-H} = 11.6 Hz, 3F), –77.8 (s, 3F).

HRMS–ESI (*m/z*): $[M–OTf^-]^+$ calcd for $C_9H_{12}F_3N_2O_2^+$, 237.0845; found, 237.0843.

2-((3-Buten-1-yloxy)carbonyl)-1,3-dimethyl-1*H*-imidazol-3-ium Trifluoromethanesulfonate (2c)

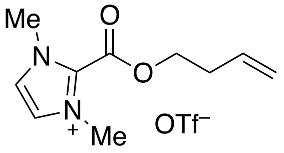

The product **2c** was synthesized from 3-buten-1-yl 1-methyl-1*H*-imidazole-2-carboxylate (335 mg, 1.9 mmol). The product was purified by recrystallization (DCM/Et_2O) to give **2c** as white solid (520 mg, 1.5 mmol, 81% isolated yield).

M.p. 107–109 °C

IR (neat) 1738.0, 1446.4, 1298.4, 1256.8, 1223.8, 1159.1, 1029.9, 756.9 cm^{-1}.

^{1}H NMR (400 MHz, $CDCl_3$) δ 7.74 (s, 2H), 5.83 (ddt, J = 17.2, 10.4, 6.8 Hz, 1H), 5.22–5.15 (m, 2H), 4.57 (t, J = 6.4 Hz, 2H), 4.17 (s, 6H), 2.59 (dt, J = 6.8, 6.4 Hz, 2H).

^{13}C NMR (100.6 MHz, $CDCl_3$) δ 153.6, 133.2, 132.1, 126.7, 120.5 (q, $J_{C–F}$ = 320.3 Hz), 118.4, 67.1, 39.4, 32.5.

^{19}F NMR (376.5 MHz, $CDCl_3$) δ –78.6 (s, 3F).

HRMS–ESI (*m/z*): [M–OTf$^-$]$^+$ calcd for $C_{10}H_{15}N_2O_2^+$, 195.1128; found, 195.1125.

2-((3-Hexyn-1-yloxy)carbonyl)-1,3-dimethyl-1*H*-imidazol-3-ium Trifluoromethanesulfonate (2d)

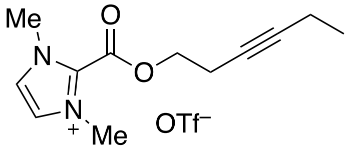

The product **2d** was synthesized from 3-hexyn-1-yl 1-methyl-1*H*-imidazole-2-carboxylate (383 mg, 1.9 mmol). The product was purified by recrystallization (DCM/Et_2O) to give **2d** as white solid (602 mg, 1.6 mmol, 86% isolated yield).

M.p. 104–106 °C

IR (neat) 1742.3, 1443.5, 1304.2, 1291.3, 1275.5, 1252.5, 1223.8, 1160.6 cm^{-1}.

^{1}H NMR (400 MHz, $CDCl_3$) δ 7.77 (s, 2H), 4.55 (t, J = 6.4 Hz, 2H), 4.22 (s, 6H), 2.69 (tt, J = 6.4, 2.4 Hz, 2H), 2.15 (qt, J = 7.6, 2.4 Hz, 2H), 1.10 (t, J = 7.6 Hz, 3H).

^{13}C NMR (100.6 MHz, $CDCl_3$) δ 153.4, 132.0, 126.8, 120.5, (q, $J_{C–F}$ = 320.1 Hz), 84.3, 74.1, 66.5, 39.5, 18.9, 13.9, 12.2.

^{19}F NMR (376.5 MHz, $CDCl_3$) δ –78.6 (s, 3F).

HRMS–ESI (*m/z*): [M–OTf$^-$]$^+$ calcd for $C_{12}H_{17}N_2O_2^+$, 221.1285; found, 221.1283.

2-(((3,7-Dimethyloct-6-en-1-yl)oxy)carbonyl)-1,3-dimethyl-1*H*-imidazol-3-ium Trifluoromethanesulfonate (2e)

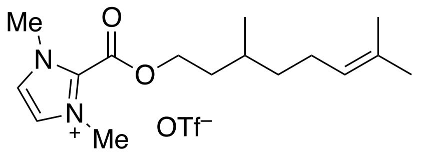

The product **2e** was synthesized from 3,7-dimethyloct-6-en-1-yl 1-methyl-1*H*-imidazole-2-carboxylate (384 mg, 1.5 mmol). The product **2e** was obtained without further purification as white solid (378 mg, 0.88 mmol, 59% isolated yield).

M.p. 107–109 °C

IR (neat) 1739.4, 1446.4, 1298.4, 1258.2, 1226.6, 1159.1, 1032.7, 640.6 cm^{-1}.

^{1}H NMR (400 MHz, $CDCl_3$) δ 7.77 (s, 2H), 5.07 (t, J = 7.2 Hz, 1H), 4.59–4.48 (m, 2H), 4.19 (s, 6H), 2.06–1.94 (m, 2H), 1.90–1.76 (m, 2H), 1.68–1.55 (m, 7H), 1.43–1.34 (m, 1H), 1.28–1.19 (m, 1H), 0.97 (d, J = 6.4 Hz, 3H).

^{13}C NMR (100.6 MHz, $CDCl_3$) δ 153.7, 132.1, 131.7, 126.8, 124.1, 120.5 (q, $J_{C–F}$ = 320.0 Hz), 67.0, 39.4, 36.8, 35.0, 29.4, 25.7, 25.2, 19.2, 17.6.

^{19}F NMR (376.5 MHz, $CDCl_3$) δ –78.6 (s, 3F).

HRMS–ESI (*m/z*): [M–OTf$^-$]$^+$ calcd for $C_{16}H_{27}N_2O_2^+$, 279.2067; found, 279.2065.

1,3-Dimethyl-2-((3-phenylpropoxy)carbonyl)-1*H*-imidazol-3-ium Trifluoromethanesulfonate (2f)

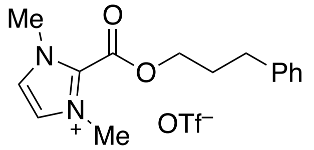

The product **2f** was synthesized from 3-phenylpropyl 1-methyl-1*H*-imidazole-2-carboxylate (301 mg, 1.2 mmol). The product was purified by recrystallization (DCM/Et_2O) to give **2f** as white solid (424 mg, 1.0 mmol, 86% isolated yield).

M.p. 86–88 °C

IR (neat) 1739.4, 1529.7, 1443.5, 1261.1, 1225.2, 1157.7, 1031.3, 755.5 cm^{-1}.

^{1}H NMR (400 MHz, $CDCl_3$) δ 7.70 (s, 2H), 7.28 (t, J = 8.0 Hz, 2H), 7.20–7.16 (m, 3H), 4.51 (t, J = 6.4 Hz, 2H), 4.13 (s, 6H), 2.77 (t, J = 7.6 Hz, 2H), 2.17 (tt, J = 7.6, 6.4 Hz, 2H).

^{13}C NMR (100.6 MHz, $CDCl_3$) δ 153.6, 140.3, 132.1, 128.5, 128.3, 126.6, 126.3, 120.6 (q, $J_{C–F}$ = 319.9 Hz), 67.8, 39.4, 32.1, 29.6.

^{19}F NMR (376.5 MHz, $CDCl_3$) δ –78.5 (s, 3F).

HRMS–ESI (*m/z*): [M–OTf$^-$]$^+$ calcd for $C_{15}H_{19}N_2O_2^+$, 259.1441; found, 259.1440.

2-(Cyclobutoxycarbonyl)-1,3-dimethyl-1*H*-imidazol-3-ium Trifluoromethanesulfonate (2g)

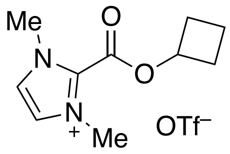

The product **2g** was synthesized from cyclobutyl 1-methyl-1*H*-imidazole-2-carboxylate (175 mg, 1.0 mmol). The product **2g** was obtained without further purification as white solid (272 mg, 0.79 mmol, 81% isolated yield).

M.p. 143–145 °C

IR (neat) 1740.8, 1447.8, 1291.3, 1258.2, 1223.8, 1163.4, 1031.3, 640.6 cm^{-1}.

^{1}**H NMR** (400 MHz, DMSO-*d*$_6$) δ 7.92 (s, 2H), 5.25 (tt, J = 10.4, 7.2 Hz, 1H), 4.05 (s, 6H), 2.44–2.37 (m, 2H), 2.34–2.24 (m, 2H), 1.86 (m, 1H), 1.70 (m, 1H).

^{13}**C NMR** (100.6 MHz, DMSO-*d*$_6$) δ 153.6, 133.2, 126.3, 121.2 (q, $J_{C–F}$ = 322.1 Hz), 72.1, 39.1, 30.0, 13.8.

^{19}**F NMR** (376.5 MHz, DMSO-*d*$_6$) δ –77.8 (s, 3F).

HRMS–ESI (*m/z*): [M–OTf$^-$]$^+$ calcd for $C_{10}H_{15}N_2O_2^+$, 195.1128; found, 195.1128.

♦ **Preparation of Sulfonyl Triazoles**

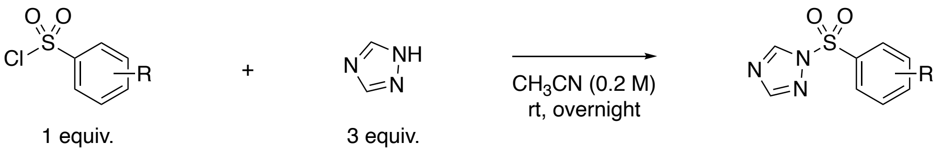

In an oven-dried 200 mL flask equipped with a stirring bar, sulfonyl chloride (10 mmol, 1.0 equiv.) was dissolved in anhydrous CH_3CN (50 mL) under a nitrogen atmosphere. Triazole (2.1 g, 30 mmol, 3.0 equiv.) was then added to the reaction mixture in a single portion, and the solution was stirred at room temperature overnight (typically 1–14 hours). After completion of the reaction, indicated by sufficient formation of a white precipitate, the reaction mixture was concentrated under reduced pressure and dissolved in DCM (30 mL). The organic layer was washed with water (20 mL) three to four times, then dried over Na_2SO_4. After filtration, the organic phase was concentrated in vacuo, and the product was used as a substrate without further purification.

1-((4-(Trifluoromethyl)phenyl)sulfonyl)-1*H*-1,2,4-triazole (4a)

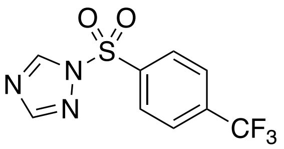

The product **4a** was synthesized using 4-(trifluoromethyl)benzenesulfonyl chloride (1.2 g, 5.0 mmol). The product **4a** was obtained as white solid (0.78 g, 2.8 mmol, 56% isolated yield).

M.p. 115–117 °C

IR (neat) 1394.7, 1320.0, 1182.1, 1172.0, 1129.0, 1103.1, 716.7, 634.9 cm^{-1}.

^{1}H NMR (400 MHz, $CDCl_3$) δ 8.79 (s, 1H), 8.25 (d, J = 8.0 Hz, 2H), 8.07 (s, 1H), 7.89 (d, J = 8.0 Hz, 2H).

^{13}C NMR (100.6 MHz, $CDCl_3$) δ 154.7, 144.8, 139.2, 137.0 (q, $J_{C–F}$ = 33.4 Hz), 129.3, 126.9 (q, $J_{C–F}$ =3.5 Hz), 122.7 (q, $J_{C–F}$ = 273.4 Hz).

^{19}F NMR (376.5 MHz, $CDCl_3$) δ –63.5 (s, 3F).

HRMS–ESI (*m/z*): $[M+H]^+$ calcd for $C_9H_7F_3N_3O_2S^+$, 278.0206; found, 278.0206.

1-((4-Chlorophenyl)sulfonyl)-1*H*-1,2,4-triazole (4b)

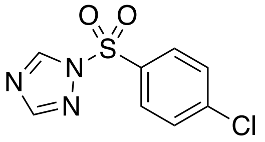

The product **4b** was synthesized using 4-chlorobenzenesulfonyl chloride (2.1 g, 10 mmol). The product **4b** was obtained as white solid (1.7 g, 7.0 mmol, 71% isolated yield).

M.p. 126–127 °C

IR (neat) 1390.4, 1190.7, 1177.8, 1137.6, 1103.1, 1095.9, 758.4, 640.6 cm^{-1}.

^{1}H NMR (400 MHz, $CDCl_3$) δ 8.76 (s, 1H), 8.05–8.03 (m, 3H), 7.59 (d, J = 8.8 Hz, 2H).

^{13}C NMR (100.6 MHz, $CDCl_3$) δ 154.5, 144.6, 142.7, 134.0, 130.14, 130.07.

HRMS–ESI (*m/z*): $[M+H]^+$ calcd for $C_8H_7ClN_3O_2S^+$, 243.9942; found, 243.9942.

4-((1*H*-1,2,4-Triazol-1-yl)sulfonyl)benzonitrile (4c)

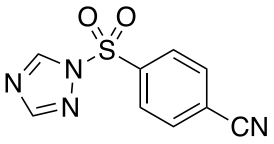

The product **4c** was synthesized using 4-cyanobenzenesulfonyl chloride (0.94 g, 4.6 mmol). The product **4c** was obtained as white solid (0.81 mg, 3.4 mmol, 74% isolated yield).

M.p. 146–148 °C

IR (neat) 1399.0, 1269.7, 1205.1, 1190.7, 1177.8, 1136.1, 1104.5, 647.8 cm^{-1}.

^{1}H NMR (400 MHz, $CDCl_3$) δ 8.79 (s, 1H), 8.24 (d, J = 8.4 Hz, 2H), 8.08 (s, 1H), 7.92 (d, J = 8.4 Hz, 2H).

^{13}C NMR (100.6 MHz, $CDCl_3$) δ 154.8, 144.9, 139.6, 133.4, 129.3, 119.2, 116.4.

HRMS–ESI (*m/z*): $[M+H]^+$ calcd for $C_9H_7N_4O_2S^+$, 235.0284; found, 235.0273.

1-((3,5-Bis(trifluoromethyl)phenyl)sulfonyl)-1*H*-1,2,4-triazole (4d)

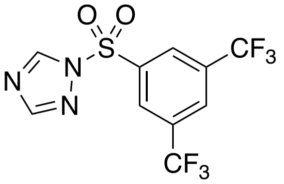

The product **4d** was synthesized using 3,5-bis(trifluoromethyl)benzenesulfonyl chloride (3.1 g, 10 mmol). The product **4d** was obtained as off-white solid (3.2 g, 9.2 mmol, 92% isolated yield).

M.p. 85–87 °C

IR (neat) 1406.2, 1279.8, 1185.0, 1137.6, 1111.7, 1100.2, 682.3, 636.3 cm^{-1}.

^{1}H NMR (400 MHz, $CDCl_3$) δ 8.83 (s, 1H), 8.57 (s, 2H), 8.24 (s, 1H), 8.12 (s, 1H).

^{13}C NMR (100.6 MHz, $CDCl_3$) δ 155.1, 145.0, 138.5, 133.8 (q, $J_{C–F}$ =35.3 Hz), 129.2–129.0 (m, ×2C), 121.9 (q, $J_{C–F}$ = 273.6 Hz).

^{19}F NMR (376.5 MHz, $CDCl_3$) δ –63.0 (s, 6F).

HRMS–ESI (*m/z*): $[M+H]^+$ calcd for $C_{10}H_6F_6N_3O_2S^+$, 346.0079; found, 346.0077.

♦ Preparation of Sulfonyl Imines

(*E*)-*N*-(4-Methylbenzylidene)cyclopropanesulfonamide (4f)

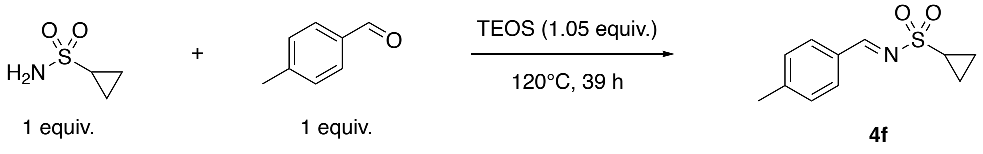

In an oven-dried 50 mL flask equipped with a stirring bar, cyclopropyl sulfonamide (607 mg, 5.0 mmol, 1.0 equiv.), *p*-tolualdehyde (589 µL, 5.0 mmol, 1.0 equiv.), and tetraethyl orthosilicate (TEOS, 1.2 mL, 5.25 mmol, 1.05 equiv.) were added under a nitrogen atmosphere. The reaction mixture was stirred at 120 °C for 39 hours. After cooling to room temperature, the mixture was dissolved in DCM,

and the organic layer was washed several times with water to remove residual sulfonamide. The organic layer was dried over Na_2SO_4. After filtration, it was concentrated under reduced pressure. The product was purified by recrystallization (hexane/AcOEt) to give **4f** as off-white solid (572 mg, 2.6 mmol, 51% isolated yield).

M.p. 98–100 °C (decomp.)

IR (neat) 1597.2, 1562.7, 1322.9, 1297.0, 1140.4, 887.7, 818.7, 801.5 cm^{-1}.

^{1}H NMR (400 MHz, $CDCl_3$) δ 8.92 (s, 1H), 7.87 (d, J = 8.4 Hz, 2H), 7.34 (d, J = 8.4 Hz, 2H), 2.61 (tt, J = 8.0, 4.8 Hz, 1H), 2.46 (s, 3H), 1.32 (ddd, J = 6.8, 4.8, 4.4 Hz, 2H), 1.11 (ddd, J = 8.0, 6.8, 4.4 Hz, 2H).

^{13}C NMR (100.6 MHz, $CDCl_3$) δ 170.8, 146.4, 131.3, 129.9, 129.7, 29.5, 22.0, 5.9.

HRMS–ESI (*m/z*): $[M+H]^+$ calcd for $C_{11}H_{14}NO_2S^+$, 224.0740; found, 224.0738.

4. General Procedures for Distal C–H Functionalization of Alkoxyarenes

♦ Alkoxycarbonylation (**Method A**)

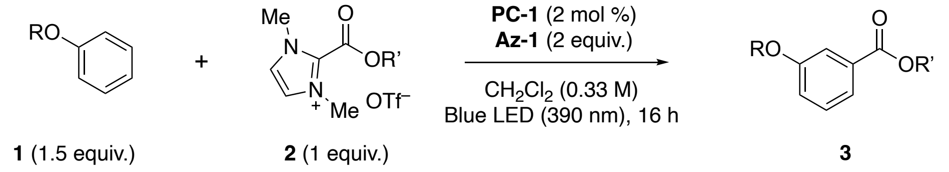

PC-1 (5.3 mg, 4.0 μmol) and imidazolium ester **2** (0.20 mmol) were placed in an oven-dried screw-top 4 mL vial containing a magnetic stirring bar. The vial was then introduced in the nitrogen-filled glovebox and to this was added imidazole **Az-1** (27.2 mg, 0.40 mmol), arene **1** (0.30 mmol), and 600 μL anhydrous dichloromethane. The vial was then brought outside the glovebox. After 16 h stirring at ambient temperature under photoirradiation (390 nm), the reaction mixture was evaporated under reduced pressure. The crude was purified by flash column chromatography on silica gel.

♦ Sulfonylation (**Method B**)

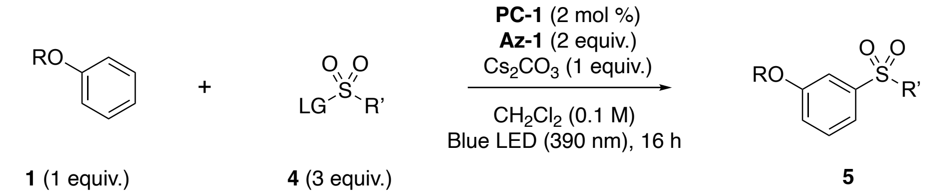

PC-1 (5.3 mg, 4.0 μmol) and sulfonylation reagent **4** (0.60 mmol) were placed in an oven-dried screw-top 4 mL vial containing a magnetic stirring bar. The vial was then introduced in the nitrogen-filled glovebox and to this was added imidazole **Az-1** (27.2 mg, 0.40 mmol), cesium carbonate (0.2 mmol), arene **1** (0.30 mmol), and 600 μL anhydrous dichloromethane. The vial was then brought outside the glovebox. After 16 h stirring at ambient temperature under photoirradiation (390 nm), the reaction mixture was passed through silica short pad using AcOEt as eluent, and then evaporated under reduced pressure. The crude was purified by flash column chromatography on silica gel.

♦ Heteroarylation (**Method C**)

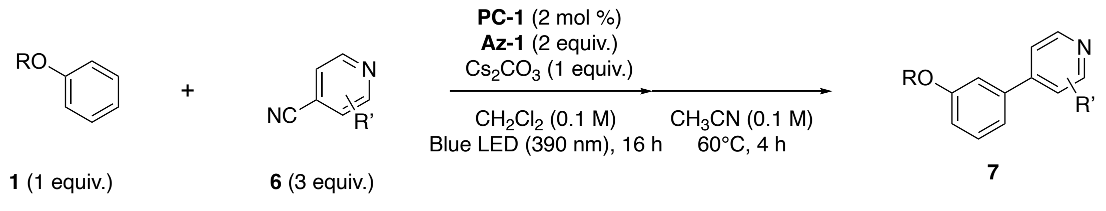

Arene **1** (0.20 mmol) and **PC-1** (5.3 mg, 4.0 μmol) were placed in an oven-dried screw-top 4 mL vial containing a magnetic stirring bar. The vial was then introduced in the nitrogen-filled glovebox and to this was added imidazole **Az-1** (27.2 mg, 0.40 mmol), cyanoarene **6** (0.60 mmol), cesium carbonate (65.2 mg, 0.20 mmol), and 2.0 mL anhydrous dichloromethane. The vial was then brought outside the glovebox. After 16 h stirring at ambient temperature under photoirradiation (390 nm), the reaction mixture was evaporated under reduced pressure and dissolved in acetonitrile (2.0 mL). After 4 h stirring at 60°C, the reaction mixture was passed through silica short pad using AcOEt as eluent. The crude was purified by flash column chromatography on silica gel.

♦ Alkylation (**Method D**)

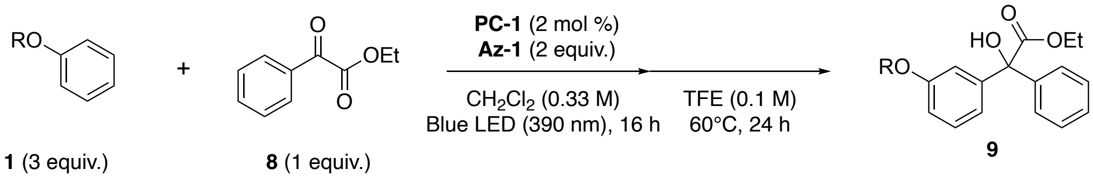

Arene **1** (0.30 mmol) and **PC-1** (2.7 mg, 2.0 μmol) were placed in an oven-dried screw-top 4 mL vial containing a magnetic stirring bar. The vial was then introduced in the nitrogen-filled glovebox and to this was added imidazole **Az-1** (13.6 mg, 0.20 mmol), ketoester **8** (0.10 mmol, 1.0 equiv.) and 300 μL anhydrous dichloromethane. The vial was then brought outside the glovebox. After 16 h stirring at ambient temperature under photoirradiation (390 nm), the reaction mixture was evaporated under reduced pressure and dissolved in 2,2,2-trifluoroethanol (1.0 mL). After 24 h stirring at 60°C, the reaction mixture was evaporated under reduced pressure. The crude was purified by preparative thin-layer chromatography.

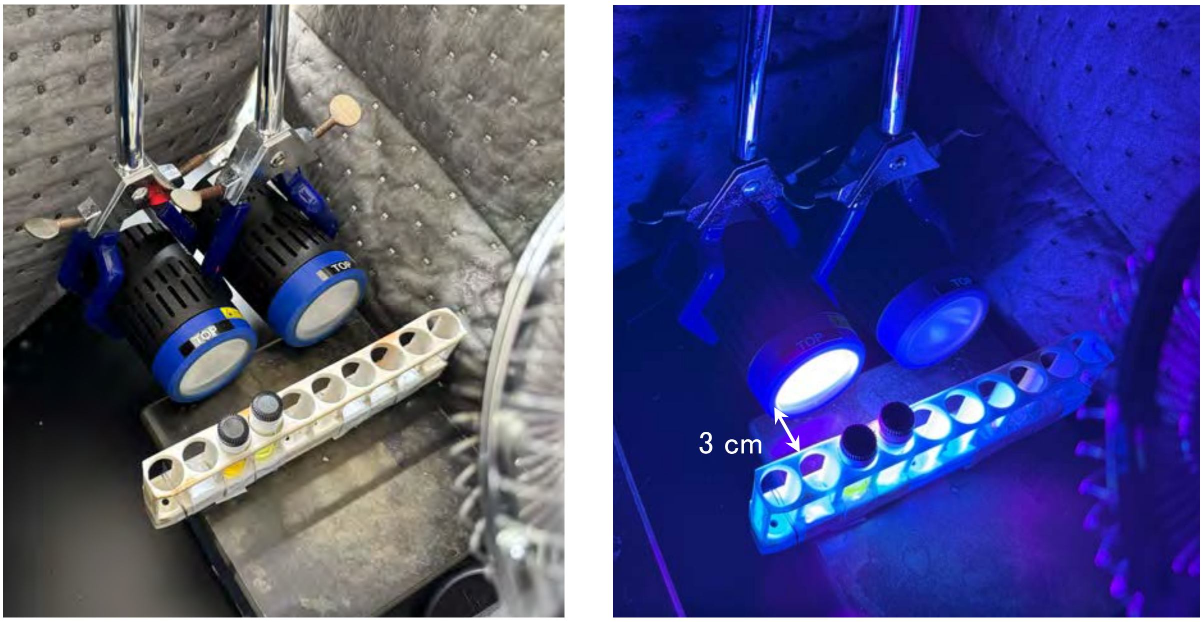

Supplementary Fig. 2. Light set-up.

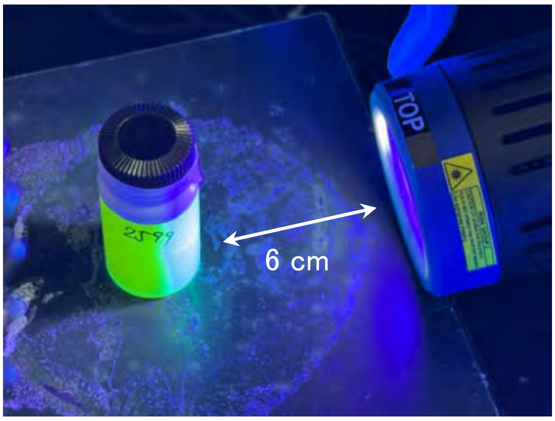

Supplementary Fig. 3. Light set-up (1 or 5 mmol scale).

5. Characterization Data for Products

Ethyl 3,5-Dimethoxy-4-methylbenzoate (3aa)

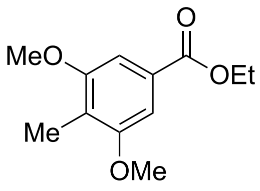

The product **3aa** was synthesized according to **Method A** on a 0.1 mmol scale, using 2,6-dimethoxytoluene **1a** (22.8 mg, 0.15 mmol) and imidazolium ester **2a** (31.8 mg, 0.1 mmol). The product was purified by flash chromatography on silica gel (100:0–95:5, hexane/AcOEt) to give **3aa** as pale-yellow solid (15.4 mg, 0.069 mmol, 69% isolated yield).

^{1}H NMR (400 MHz, $CDCl_3$) δ 7.23 (s, 2H), 4.38 (q, J = 7.2 Hz, 2H), 3.88 (s, 6H), 2.13 (s, 3H), 1.41 (t, J = 7.2 Hz, 3H).

^{13}C NMR (100.6 MHz, $CDCl_3$) δ 166.8, 158.0, 128.5, 120.1, 104.6, 61.0, 55.8, 14.4, 8.6.

The ^{1}H and ^{13}C NMR spectra data of product **3aa** were consistent with the literature.[11]

3,3,3-Trifluoropropyl 3,5-Dimethoxy-4-methylbenzoate (3ab)

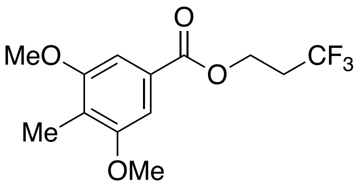

The product **3ab** was synthesized according to **Method A** using 2,6-dimethoxytoluene **1a** (45.6 mg, 0.3 mmol) and imidazolium ester **2b** (77.3 mg, 0.2 mmol). The product was purified by flash chromatography on silica gel (100:0–96:4, hexane/AcOEt) to give **3ab** as white solid (43.2 mg, 0.19 mmol, 97% isolated yield).

M.p. 62–64 °C

IR (neat) 1707.8, 1590.0, 1413.4, 1318.6, 1264.0, 1243.9, 1228.1, 1137.6 cm^{-1}.

^{1}H NMR (400 MHz, $CDCl_3$) δ 7.22 (s, 2H), 4.54 (t, J = 6.4 Hz, 2H), 3.86 (s, 6H), 2.61 (qt, J = 10.4, 6.4 Hz, 2H), 2.13 (s, 3H).

^{13}C NMR (100.6 MHz, $CDCl_3$) δ 166.2, 158.1, 127.5, 125.9 (q, $J_{C–F}$ = 276.8 Hz), 120.8, 104.6, 57.6 (q, $J_{C–F}$ = 3.6 Hz), 55.7, 33.4 (q, $J_{C–F}$ = 29.2 Hz), 8.6.

^{19}F NMR (376.5 MHz, $CDCl_3$) δ –64.9 (t, $J_{F–H}$ = 10.4 Hz, 3F).

HRMS–ESI (*m/z*): $[M+H]^+$ calcd for $C_{13}H_{16}F_3O_4^+$, 293.0995; found, 293.1010.

3-Butenyl 3,5-Dimethoxy-4-methylbenzoate (3ac)

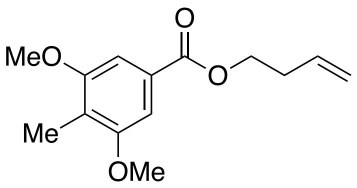

The product **3ac** was synthesized according to **Method A** using 2,6-dimethoxytoluene **1a** (45.6 mg, 0.3 mmol) and imidazolium ester **2c** (68.9 mg, 0.2 mmol). The product was purified by preparative thin-layer chromatography (95:5, hexane/AcOEt) to give **3ac** as colorless oil (29.4 mg, 0.12 mmol, 59% isolated yield).

IR (neat) 1713.5, 1588.6, 1410.5, 1315.7, 1230.9, 1180.7, 1136.1, 1113.2 cm^{-1}.

^{1}H NMR (400 MHz, $CDCl_3$) δ 7.22 (s, 2H), 5.88 (ddt, J = 17.2, 10.4, 6.8 Hz, 1H), 5.18 (ddt, J = 17.2, 1.6, 1.2 Hz, 1H), 5.11 (dd, J = 10.4, 1.6 Hz, 1H), 4.37 (t, J = 6.8 Hz, 2H), 3.87 (s, 6H), 2.54 (tdd, J = 6.8, 6.8, 1.2 Hz, 2H), 2.13 (s, 3H).

^{13}C NMR (100.6 MHz, $CDCl_3$) δ 166.6, 158.0, 134.1, 128.3, 120.2, 117.3, 104.5, 63.9, 55.8, 33.2, 8.6.

HRMS–ESI (*m/z*): $[M+Na]^+$ calcd for $C_{14}H_{18}NaO_4^+$, 273.1097; found, 273.1090.

3-Hexyn-1-yl 3,5-Dimethoxy-4-methylbenzoate (3ad)

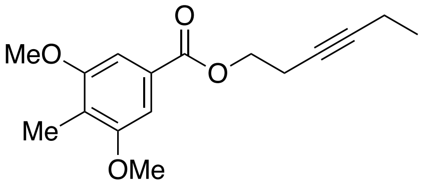

The product **3ad** was synthesized according to **Method A** using 2,6-dimethoxytoluene **1a** (45.6 mg, 0.3 mmol) and imidazolium ester **2d** (74.1 mg, 0.2 mmol). The product was purified by flash chromatography on silica gel (100:0–96:4, hexane/AcOEt) to give **3ad** as white solid (23.3 mg, 0.084 mmol, 42% isolated yield).

M.p. 72–74 °C

IR (neat) 1709.2, 1588.6, 1407.6, 1315.7, 1229.5, 1183.5, 1139.0, 1114.6 cm^{-1}.

^{1}H NMR (400 MHz, $CDCl_3$) δ 7.24 (s, 2H), 4.38 (t, J = 7.2 H, 2H), 3.87 (s, 6H), 2.63 (tt, J = 7.2, 2.4 Hz, 2H), 2.17 (qt, J = 7.6, 2.4 Hz, 2H), 2.13 (s, 3H), 1.12 (t, J = 7.6 Hz, 3H).

^{13}C NMR (100.6 MHz, $CDCl_3$) δ 166.5, 158.0, 128.1, 120.4, 104.6, 83.5, 74.8, 63.2, 55.8, 19.3, 14.1, 12.3, 8.6.

HRMS–ESI (*m/z*): $[M+Na]^+$ calcd for $C_{16}H_{20}NaO_4^+$, 299.1254; found, 299.1250.

3,7-Dimethyloct-6-enyl 3,5-Dimethoxy-4-methylbenzoate (3ae)

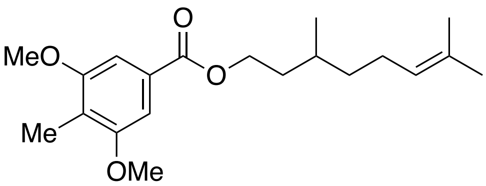

The product **3ae** was synthesized according to **Method A** using 2,6-dimethoxytoluene **1a** (45.6 mg, 0.3 mmol) and imidazolium ester **2e** (85.7 mg, 0.2 mmol). The product was purified by flash chromatography on silica gel (100:0–97:3, hexane/AcOEt) to give **3ae** as colorless oil (33.6 mg, 0.10 mmol, 50% isolated yield).

IR (neat) 1716.4, 1590.0, 1455.0, 1410.5, 1317.1, 1232.4, 1182.1, 1140.4 cm^{-1}.

^{1}H NMR (400 MHz, $CDCl_3$) δ 7.22 (s, 2H), 5.10 (m, 1H), 4.38–4.34 (m, 2H), 3.87 (s, 6H), 2.13 (s, 3H), 2.09–1.93 (m, 2H), 1.83 (m, 1H), 1.67–1.55 (m, 8H), 1.42 (m, 1H), 1.25 (m, 1H), 0.98 (d, J = 6.4 Hz, 3H).

^{13}C NMR (100.6 MHz, $CDCl_3$) δ 166.8, 158.0, 131.4, 128.5, 124.5, 120.2, 104.6, 63.6, 55.8, 37.0, 35.5, 29.8, 25.7, 25.4, 19.5, 17.6, 8.6.

HRMS–ESI (*m/z*): $[M+H]^+$ calcd for $C_{20}H_{31}O_4^+$, 335.2217; found, 335.2201.

3-Phenylpropyl 3,5-Dimethoxy-4-methylbenzoate (3af)

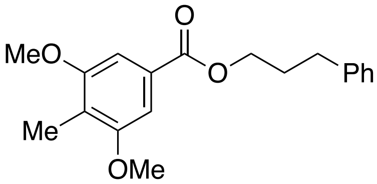

The product **3af** was synthesized according to **Method A** using 2,6-dimethoxytoluene **1a** (45.6 mg, 0.3 mmol) and imidazolium ester **2f** (81.7 mg, 0.2 mmol). The product was purified by flash chromatography on silica gel (100:0–97:3, hexane/AcOEt) to give **3af** as colorless oil (24.9 mg, 0.079 mmol, 40% isolated yield).

IR (neat) 1713.5, 1588.6, 1455.0, 1410.5, 1315.7, 1232.4, 1180.7, 1137.6 cm^{-1}.

^{1}H NMR (400 MHz, $CDCl_3$) δ 7.30 (t, J = 7.6 Hz, 2H), 7.23 (s, 2H), 7.22–7.18 (m, 3H), 4.34 (t, J = 6.8 Hz, 2H), 3.88 (s, 6H), 2.78 (t, J = 8.0 Hz, 2H), 2.14 (s, 3H), 2.12 (tt, J = 8.0, 6.8 Hz, 2H).

^{13}C NMR (100.6 MHz, $CDCl_3$) δ 166.7, 158.0, 141.1, 128.45, 128.38 (×2C), 126.0, 120.3, 104.6, 64.2, 55.8, 32.2, 30.3, 8.6.

HRMS–ESI (*m/z*): $[M+H]^+$ calcd for $C_{19}H_{23}O_4^+$, 315.1591; found, 315.1587.

Cyclobutyl 3,5-Dimethoxy-4-methylbenzoate (3ag)

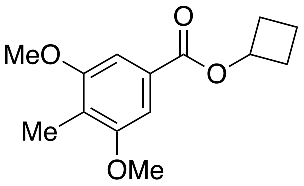

The product **3ag** was synthesized according to **Method A** using 2,6-dimethoxytoluene **1a** (45.6 mg, 0.3 mmol) and imidazolium ester **2g** (68.9 mg, 0.2 mmol). The product was purified by flash chromatography on silica gel (100:0–96:4, hexane/AcOEt) to give **3ag** as colorless oil (24.6 mg, 0.098 mmol, 49% isolated yield).

IR (neat) 1713.5, 1588.6, 1410.5, 1315.7, 1232.4, 1182.1, 1139.0, 1114.6 cm^{-1}.

^{1}H NMR (400 MHz, $CDCl_3$) δ 7.21 (s, 2H), 5.20 (tt, J = 7.2, 7.2 Hz, 1H), 3.88 (s, 6H), 2.51–2.43 (m, 2H), 2.29–2.18 (m, 2H), 2.13 (s, 3H), 1.86 (m, 1H), 1.72 (m, 1H).

^{13}C NMR (100.6 MHz, $CDCl_3$) δ 166.1, 158.0, 128.4, 120.2, 104.5, 69.4, 55.8, 30.4, 13.6, 8.6.

HRMS–ESI (*m/z*): $[M+Na]^+$ calcd for $C_{14}H_{18}NaO_4^+$, 273.1097; found, 273.1093.

1,3-Dimethoxy-2-methyl-5-((4-(trifluoromethyl)phenyl)sulfonyl)benzene (5aa)

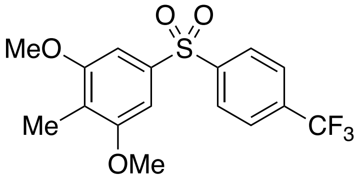

The product **5aa** was synthesized according to **Method B** using 2,6-dimethoxytoluene (30.4 mg, 0.2 mmol) and 1-((4-(trifluoromethyl)phenyl)sulfonyl)-1*H*-1,2,4-triazole **4a** (166 mg, 0.6 mmol). The product was purified by flash chromatography on silica gel (100:0–90:10, hexane/AcOEt) to give **5aa** as white solid (29.6 mg, 0.082 mmol, 41% isolated yield).

M.p. 142–143 °C

IR (neat) 1404.7, 1320.0, 1297.0, 1134.7, 1106.0, 1061.5, 711.0, 636.3 cm^{-1}.

^{1}H NMR (400 MHz, $CDCl_3$) δ 8.06 (d, J = 8.4 Hz, 2H), 7.76 (d, J = 8.4 Hz, 2H), 7.09 (s, 2H), 3.87 (s, 6H), 2.10 (s, 3H).

^{13}C NMR (100.6 MHz, $CDCl_3$) δ 158.7, 145.6, 138.0, 134.6 (q, J_{C-F} = 33.0 Hz), 127.8, 126.4 (q, J_{C-F} = 3.7 Hz), 123.1 (q, J_{C-F} = 273.0 Hz), 121.5, 102.9, 56.1, 8.7.

^{19}F NMR (376.5 MHz, $CDCl_3$) δ –63.2 (s, 3F).

HRMS–ESI (*m/z*): $[M+H]^+$ calcd for $C_{16}H_{16}F_3O_4S^+$, 361.0716; found, 361.0712.

5-((4-Chlorophenyl)sulfonyl)-1,3-dimethoxy-2-methylbenzene (5ab)

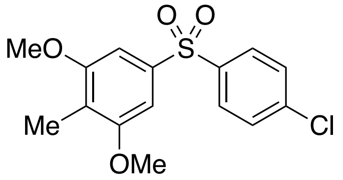

The product **5ab** was synthesized according to **Method B** using 2,6-dimethoxytoluene **1a** (30.4 mg, 0.2 mmol) and 1-((4-chlorophenyl)sulfonyl)-1*H*-1,2,4-triazole **4b** (146 mg, 0.6 mmol). The product was purified by flash chromatography on silica gel (100:0–90:10, hexane/AcOEt) to give **5ab** as white solid (20.9 mg, 0.064 mmol, 32% isolated yield).

M.p. 139–142 °C

IR (neat) 1590.0, 1404.7, 1317.1, 1297.0, 1141.9, 1088.7, 759.8, 646.4 cm^{-1}.

^{1}H NMR (400 MHz, $CDCl_3$) δ 7.87 (d, J = 8.8 Hz, 2H), 7.47 (d, J = 8.8 Hz, 2H), 7.06 (s, 2H), 3.86 (s, 6H), 2.09 (s, 3H).

^{13}C NMR (100.6 MHz, $CDCl_3$) δ 158.6, 140.5, 139.6, 138.7, 129.5, 128.8, 121.0, 102.6, 56.0, 8.7.

HRMS–ESI (*m/z*): $[M+H]^+$ calcd for $C_{15}H_{16}ClO_4S^+$, 327.0452; found, 327.0443.

4-((3,5-Dimethoxy-4-methylphenyl)sulfonyl)benzonitrile (5ac)

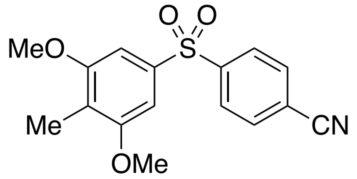

The product **5ac** was synthesized according to **Method B** using 2,6-dimethoxytoluene **1a** (30.4 mg, 0.2 mmol) and 4-((1*H*-1,2,4-triazol-1-yl)sulfonyl)benzonitrile **4c** (141 mg, 0.6 mmol). The product was purified by flash chromatography on silica gel (100:0–80:20, hexane/AcOEt) to give **5ac** as white solid (21.6 mg, 0.068 mmol, 34% isolated yield).

M.p. 197–199 °C

IR (neat) 1590.0, 1406.2, 1321.4, 1297.0, 1141.9, 1107.4, 655.0, 594.6 cm^{-1}.

^{1}H NMR (400 MHz, $CDCl_3$) δ 8.04 (d, J = 8.4 Hz, 2H), 7.79 (d, J = 8.4 Hz, 2H), 7.07 (s, 2H), 3.87 (s, 6H), 2.10 (s, 3H).

^{13}C NMR (100.6 MHz, $CDCl_3$) δ 158.7, 146.2, 137.5, 133.0, 127.9, 121.8, 117.2, 116.6, 102.9, 56.1, 8.7.

HRMS–ESI (*m/z*): $[M+H]^+$ calcd for $C_{16}H_{16}NO_4S^+$, 318.0795; found, 318.0783.

5-((3,5-Bis(trifluoromethyl)phenyl)sulfonyl)-1,3-dimethoxy-2-methylbenzene (5ad)

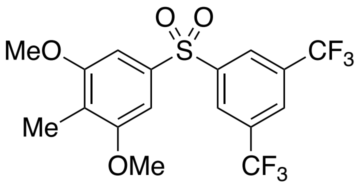

The product **5ad** was synthesized according to **Method B** using 2,6-dimethoxytoluene **1a** (30.4 mg, 0.2 mmol) and 1-((3,5-bis(trifluoromethyl)phenyl)sulfonyl)-1*H*-1,2,4-triazole **4d** (207 mg, 0.6 mmol). The product was purified by flash chromatography on silica gel (100:0–97:3, hexane/AcOEt) to give **5ad** as white solid (27.0 mg, 0.063 mmol, 32% isolated yield).

M.p. 164–167 °C

IR (neat) 1590.0, 1409.0, 1360.2, 1311.4, 1278.3, 1241.0, 1187.8, 1143.3 cm^{-1}.

^{1}H NMR (400 MHz, $CDCl_3$) δ 8.38 (s, 2H), 8.05 (s, 1H), 7.10 (s, 2H). 3.89 (s, 6H), 2.11 (s, 3H).

^{13}C NMR (100.6 MHz, $CDCl_3$) δ 158.9, 145.0, 137.1, 133.1 (q, $J_{C–F}$ = 34.7 Hz), 127.6 (q, $J_{C–F}$ = 3.4 Hz), 126.6 (m, $J_{C–F}$ = 3.5 Hz), 122.3 (q, $J_{C–F}$ = 273.3 Hz), 122.2, 102.9, 56.2, 8.8.

^{19}F NMR (376.5 MHz, $CDCl_3$) δ –62.9 (s, 6F).

HRMS–ESI (*m/z*): $[M+Na]^+$ calcd for $C_{17}H_{14}F_6NaO_4S^+$, 451.0409; found, 451.0399.

1,3-Dimethoxy-2-methyl-5-(methylsulfonyl)benzene (5ae)

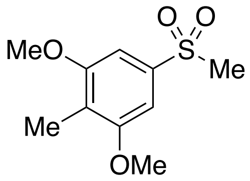

The product **5ae** was synthesized according to **Method B** using 2,6-dimethoxytoluene **1a** (30.4 mg, 0.2 mmol) and (*E*)-*N*-(4-methylbenzylidene)methanesulfonamide **4e** (118 mg, 0.6 mmol). The product was purified by flash chromatography on silica gel (100:0–70:30, hexane/AcOEt) to give **5ae** as pale-yellow solid (10.6 mg, 0.046 mmol, 23% isolated yield).

M.p. 126–129 °C

IR (neat) 1587.1, 1403.3, 1292.7, 1130.4, 1107.4, 762.7, 745.5, 555.9 cm^{-1}.

^{1}H NMR (400 MHz, $CDCl_3$) δ 7.08 (s, 2H), 3.89 (s, 6H), 3.06 (s, 3H), 2.14 (s, 3H).

^{13}C NMR (100.6 MHz, $CDCl_3$) δ 158.5, 138.3, 120.9, 102.2, 56.0, 44.6, 8.6.

HRMS–ESI (*m/z*): $[M+H]^+$ calcd for $C_{10}H_{15}O_4S^+$, 231.0686; found, 231.0665.

5-(Cyclopropylsulfonyl)-1,3-dimethoxy-2-methylbenzene (5af)

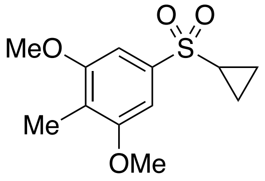

The product **5af** was synthesized according to **Method B** on a 0.1 mmol scale, using 2,6-dimethoxytoluene **1a** (15.2 mg, 0.1 mmol) and (*E*)-*N*-(4-methylbenzylidene)cyclopropanesulfonamide **4f** (89.3 mg, 0.4 mmol). The product was purified by flash chromatography on silica gel (100:0–85:15, hexane/AcOEt) to give **5af** as white solid (9.3 mg, 0.036 mmol, 18% isolated yield).

M.p. 127–129 °C

IR (neat) 1588.6, 1403.3, 1295.6, 1136.1, 899.1, 703.8, 604.7, 593.2 cm^{-1}.

^{1}H NMR (400 MHz, $CDCl_3$) δ 7.03 (s, 2H), 3.89 (s, 6H), 2.48 (tt, *J* = 8.0, 4.8 Hz, 1H), 2.14 (s, 3H), 1.34 (ddd, *J* = 4.8, 2.0, 1.6 Hz, 2H), 1.03 (ddd, *J* = 8.0, 2.0, 1.6 Hz, 2H).

^{13}C NMR (100.6 MHz, $CDCl_3$) δ 158.5, 138.5, 120.6, 102.5, 56.0, 33.1, 8.7, 6.0.

HRMS–ESI (*m/z*): $[M+H]^+$ calcd for $C_{12}H_{17}O_4S^+$, 257.0842; found, 257.0827.

4-(3,5-Dimethoxy-4-methylphenyl)pyridine (7aa)

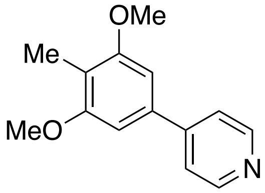

The product **7aa** was synthesized according to **Method C** using 2,6-dimethoxytoluene **1a** (30.4 mg, 0.2 mmol) and 4-cyanopyridine **6a** (62.5 mg, 0.2 mmol). The product was purified by flash chromatography on silica gel (100:0–70:30, hexane/AcOEt) to give **7aa** as pale-yellow solid (28.4 mg, 0.12 mmol, 62% isolated yield).

M.p. 100–103 °C

IR (neat) 1585.7, 1541.2, 1455.0, 1401.9, 1331.5, 1242.4, 1183.5, 1140.4 cm^{-1}.

^{1}H NMR (400 MHz, $CDCl_3$) δ 8.64 (dd, *J* = 4.4, 1.6 Hz, 2H), 7.49 (dd, *J* = 4.4, 1.6 Hz, 2H), 6.77 (s, 2H), 3.90 (s, 6H), 2.15 (s, 3H).

^{13}C NMR (100.6 MHz, $CDCl_3$) δ 158.8, 150.1, 148.9, 136.6, 121.6, 115.8, 102.4, 55.8, 8.2.

HRMS–ESI (*m/z*): $[M+H]^+$ calcd for $C_{14}H_{16}NO_2^+$, 230.1176; found, 230.1170.

1-(3,5-Dimethoxy-4-methylphenyl)isoquinoline (7ab)

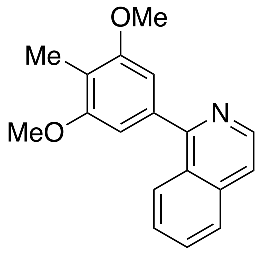

The product **7ab** was synthesized according to **Method C** using 2,6-dimethoxytoluene **1a** (30.4 mg, 0.2 mmol) and isoquinoline-1-carbonitrile **6b** (92.5 mg, 0.6 mmol). The product was purified by preparative thin-layer chromatography (85:15, hexane/AcOEt) to give **7ab** as white solid (35.2 mg, 0.13 mmol, 63% isolated yield).

M.p. 210–213 °C

IR (neat) 1581.4, 1554.1, 1456.4, 1407.6, 1386.1, 1357.3, 1236.7, 1136.1 cm^{-1}.

^{1}H NMR (400 MHz, $CDCl_3$) δ 8.61 (d, J = 6.0 Hz, 1H), 8.19 (d, J = 8.8 Hz, 1H), 7.90 (d, J = 8.0 Hz, 1H), 7.72 (ddd, J = 8.0, 6.8, 1.2 Hz, 1H), 7.67 (d, J = 6.0 Hz, 1H), 7.56 (ddd, J = 8.8, 6.8, 1.2 Hz, 1H), 6.87 (s, 2H), 3.87 (s, 6H), 2.20 (s, 3H).

^{13}C NMR (100.6 MHz, $CDCl_3$) δ 160.9, 158.1, 141.4, 137.04, 136.96, 130.3, 127.8, 127.3, 127.0, 126.7, 120.0, 115.2, 105.5, 55.9, 8.3.

HRMS–ESI (*m/z*): $[M+H]^+$ calcd for $C_{18}H_{18}NO_2^+$, 280.1332; found, 280.1326.

4-(3-Methoxynaphthalen-1-yl)pyridine (7ba)

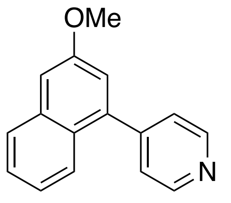

The product **7ba** was synthesized according to **Method C** using 2-methoxynaphthalene **1b** (31.6 mg, 0.2 mmol) and 4-cyanopyridine **6a** (62.5 mg, 0.6 mmol). The product was purified by flash chromatography on silica gel (100:0–80:20, hexane/AcOEt) to give **7ba** as pale-yellow solid (14.7 mg, 0.062 mmol, 31% isolated yield).

^{1}H NMR (400 MHz, $CDCl_3$) δ 8.73 (d, J = 6.0 Hz, 2H), 7.82 (d, J = 8.4 Hz, 1H), 7.72 (d, J = 8.4 Hz, 1H), 7.48 (ddd, J = 8.4, 8.4, 1.2 Hz, 1H), 7.43 (d, J =6.0 Hz, 2H), 7.32 (ddd, J = 8.4, 8.4, 1.2 Hz, 1H), 7.21 (d, J = 2.8 Hz, 1H), 7.10 (d, J = 2.8 Hz, 1H), 3.96 (s, 3H).

^{13}C NMR (100.6 MHz, $CDCl_3$) δ 156.8, 149.7, 148.2, 138.9, 135.2, 127.4, 126.7, 126.4, 125.1, 124.9, 124.2, 119.6, 106.5, 55.4.

The ^{1}H and ^{13}C NMR spectra data of product **7ba** were consistent with the literature.[12]

Ethyl 2-(3,5-Dimethoxy-4-methylphenyl)-2-hydroxy-2-phenylacetate (9aa)

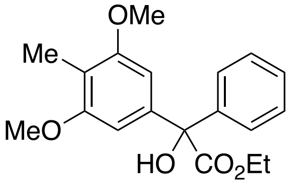

The product **9aa** was synthesized according to **Method D** using 2,6-dimethoxytoluene **1a** (45.6 mg, 0.3 mmol) and ethyl benzoylformate **8a** (15.9 μL, 0.1 mmol). The product was purified by preparative thin-layer chromatography (70:30, hexane/AcOEt) to give **9aa** as colorless oil (5.1 mg, 0.015 mmol, 15% isolated yield).

IR (neat) 3494.6, 2956.0, 1725.0, 1590.0, 1452.1, 1411.9, 1301.3, 1249.6 cm^{-1}.

^{1}H NMR (400 MHz, $CDCl_3$) δ 7.42–7.39 (m, 2H), 7.36–7.31 (m, 3H), 6.69 (s, 2H), 4.41–4.26 (m, 2H), 4.25 (s, 1H), 3.76 (s, 6H), 2.09 (s, 3H), 1.30 (t, J = 7.2 Hz, 3H).

^{13}C NMR (100.6 MHz, $CDCl_3$) δ 174.5, 157.8, 142.0, 139.9, 128.03, 128.01, 127.4, 114.3, 103.0, 81.0, 62.9, 55.7, 14.1, 8.1.

HRMS–ESI (*m/z*): $[M+Na]^+$ calcd for $C_{19}H_{22}NaO_5^+$, 353.1359; found, 353.1362.

Ethyl 3-Methoxy-1-naphthoate (3ba)

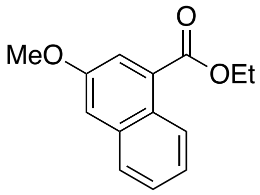

The product **3ba** was synthesized according to **Method A** using 2-methoxynaphthalene **1b** (47.5 mg, 0.3 mmol) and 2-(ethoxycarbonyl)-1,3-dimethyl-1*H*-imidazol-3-ium trifluoromethanesulfonate **2a** (63.7 mg, 0.2 mmol). The product was purified by flash chromatography on silica gel (98:2, hexane/AcOEt) to give **3ba** as white solid (19.3 mg, 0.084 mmol, 42% isolated yield).

M.p. 33–35 °C

IR (neat) 1712.1, 1601.5, 1292.7, 1219.4, 1202.2, 1149.1, 1127.5, 1050.0 cm^{-1}.

^{1}H NMR (400 MHz, $CDCl_3$) δ 8.79 (dd, J = 8.0, 1.6 Hz, 1H), 7.84 (d, J = 2.8 Hz, 1H), 7.77 (dd, J = 7.6, 2.4 Hz, 1H), 7.51–7.43 (m, 2H), 7.31 (d, J = 2.8 Hz, 1H), 4.46 (q, J = 6.8 Hz, 2H), 3.95 (s, 3H), 1.45 (t, J = 6.8 Hz, 3H).

^{13}C NMR (100.6 MHz, $CDCl_3$) δ 167.1, 156.0, 135.2, 129.0, 127.3, 126.71, 126.65, 125.7, 125.2, 122.6, 110.9, 61.2, 55.5, 14.3.

HRMS–ESI (*m/z*): $[M+H]^+$ calcd for $C_{14}H_{15}O_3^+$, 231.1016; found, 231.1008.

Ethyl 3-(3-Chloropropoxy)-5-methoxy-4-methylbenzoate (3ca)

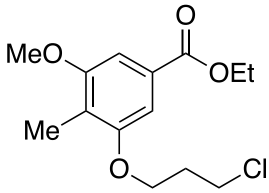

The product **3ca** was synthesized according to **Method A** using 1-(3-chloropropoxy)-3-methoxy-2-methylbenzene **1c** (64.4 mg, 0.3 mmol) and 2-(ethoxycarbonyl)-1,3-dimethyl-1*H*-imidazol-3-ium trifluoromethanesulfonate **2a** (63.7 mg, 0.2 mmol). The product was purified by flash chromatography on silica gel (100:0–95:5, hexane/AcOEt) to give **3ca** as colorless oil (22.6 mg, 0.079 mmol, 39% isolated yield, contains 6% inseparable impurity).

IR (neat) 1713.5, 1588.6, 1417.7, 1367.4, 1317.1, 1233.8, 1182.1, 1136.1 cm^{-1}.

^{1}H NMR (400 MHz, $CDCl_3$) δ 7.23 (s, 2H), 4.38 (q, J = 7.2 Hz, 2H), 4.17 (t, J = 6.0 Hz, 2H), 3.88 (s, 3H), 3.77 (t, J = 6.4 Hz, 2H), 2.28 (tt, J = 6.4, 6.0 Hz, 2H), 2.13 (s, 3H), 1.40 (t, J = 7.2 Hz, 3H).

^{13}C NMR (100.6 MHz, $CDCl_3$) δ 166.7, 158.1, 157.0, 128.6, 120.3, 105.5, 104.7, 64.8, 61.0, 55.8, 41.5, 32.3, 14.4, 8.7.

HRMS–ESI (*m/z*): $[M+H]^+$ calcd for $C_{14}H_{20}ClO_4^+$, 287.1045; found, 287.1037.

Ethyl 3-Methoxy-5-(methoxymethoxy)-4-methylbenzoate (3da)

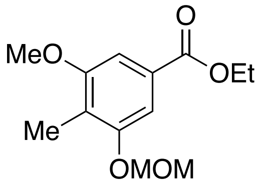

The product **3da** was synthesized according to **Method A** using 1-methoxy-3-(methoxymethoxy)-2-methylbenzene **1d** (54.7 mg, 0.3 mmol) and 2-(ethoxycarbonyl)-1,3-dimethyl-1*H*-imidazol-3-ium trifluoromethanesulfonate **2a** (63.7 mg, 0.2 mmol). The product was purified by flash chromatography on silica gel (100:0–95:5, hexane/AcOEt) to give **3da** as colorless oil (18.1 mg, 0.071 mmol, 36% isolated yield).

IR (neat) 1716.4, 1590.0, 1399.0, 1315.7, 1235.2, 1156.2, 1075.8, 1028.4 cm^{-1}.

^{1}H NMR (400 MHz, $CDCl_3$) δ 7.39 (d, J = 0.8 Hz, 1H), 7.26 (d, J = 0.8 Hz, 1H), 5.24 (s, 2H), 4.37 (q, J = 7.2 Hz, 2H), 3.88 (s, 3H), 3.50 (s, 3H), 2.17 (s, 3H), 1.39 (t, J = 7.2 Hz, 3H).

^{13}C NMR (100.6 MHz, $CDCl_3$) δ 166.6, 158.2, 155.5, 128.7, 121.3, 108.1, 105.2, 94.6, 61.0, 56.2, 55.8, 14.4, 8.9.

HRMS–ESI (*m/z*): $[M+H]^+$ calcd for $C_{13}H_{19}O_5^+$, 225.1227; found, 225.1222.

Ethyl 3-((4-(Trifluoromethyl)benzyl)oxy)-1-naphthoate (3ea)

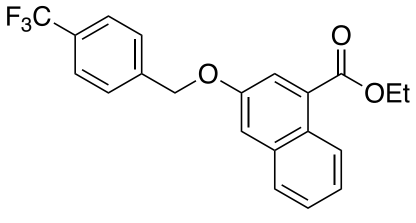

The product **3ea** was synthesized according to **Method A** using 2-((4-(trifluoromethyl)benzyl)oxy)naphthalene **1e** (90.7 mg, 0.3 mmol) amd 2-(ethoxycarbonyl)-1,3-dimethyl-1*H*-imidazol-3-ium trifluoromethanesulfonate **2a** (63.7 mg, 0.2 mmol). The product was purified by flash chromatography on silica gel (100:0–96:4, hexane/AcOEt) to give **3ea** as white solid (25.0 mg, 0.067 mmol, 33% isolated yield).

M.p. 63–65 °C

IR (neat) 1716.4, 1325.7, 1239.6, 1218.0, 1196.5, 1166.3, 1127.5, 1067.2 cm^{-1}.

^{1}**H NMR** (400 MHz, $CDCl_3$) δ 8.80 (dd, J = 7.6, 2.8 Hz, 1H), 7.94 (d, J = 2.8 Hz, 1H), 7.75 (dd, J = 7.2, 2.4 Hz, 1H), 7.67 (d, J = 8.4 Hz, 2H), 7.61 (d, J = 8.4 Hz, 2H), 7.51–7.45 (m, 2H), 7.36 (d, J = 2.8 Hz, 1H), 5.25 (s, 2H), 4.47 (q, J = 7.2 Hz, 2H), 1.45 (t, J = 7.2 Hz, 3H).

^{13}**C NMR** (100.6 MHz, $CDCl_3$) δ 167.0, 154.7, 140.49, 140.48, 135.1, 130.3 (q, $J_{C–F}$ = 32.5 Hz), 129.2, 127.44, 127.38, 127.0, 126.8, 125.7, 125.6 (q, $J_{C–F}$ = 3.5 Hz), 124.0 (q, $J_{C–F}$ = 271.9 Hz), 122.8, 112.3, 69.3, 61.3, 14.3.

^{19}**F NMR** (376.5 MHz, $CDCl_3$) δ –62.5 (s, 3F).

HRMS–ESI (*m/z*): $[M+H]^+$ calcd for $C_{21}H_{18}F_3O_3^+$, 375.1203; found, 375.1209.

Ethyl 3,5-Diisopropoxy-4-methylbenzoate (3fa)

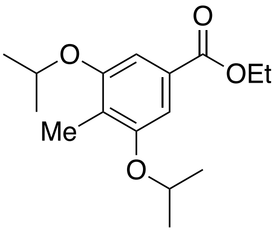

The product **3fa** was synthesized according to **Method A** using 1,3-diisopropoxy-2-methylbenzene (**1f**) (44.8 mg, 0.3 mmol) and 2-(ethoxycarbonyl)-1,3-dimethyl-1*H*-imidazol-3-ium trifluoromethanesulfonate **2a** (63.7 mg, 0.2 mmol). The product was purified by flash chromatography on silica gel (100:0–95:5, hexane/AcOEt) to give **3fa** as colorless oil (7.9 mg, 0.030 mmol, 15% isolated yield, contains trace amount of inseparable impurity).

IR (neat) 2977.5, 1716.4, 1587.1, 1422.0, 1311.4, 1233.8, 1123.2, 1031.3 cm^{-1}.

^{1}**H NMR** (400 MHz, $CDCl_3$) δ 7.19 (s, 2H), 4.59 (septet, J = 6.0 Hz, 2H), 4.36 (q, J = 7.2 Hz, 2H),

2.11 (s, 3H), 1.39 (t, J = 7.2 Hz, 3H), 1.34 (d, J = 6.0 Hz, 12H).

^{13}C NMR (100.6 MHz, $CDCl_3$) δ 166.9, 156.7, 128.2, 123.3, 107.4, 70.8, 60.9, 22.2, 14.4, 9.3.

HRMS–ESI (*m/z*): $[M+Na]^+$ calcd for $C_{16}H_{24}NaO_3^+$, 287.1618; found, 287.1617.

Dimethyl 11,11'-((5-(Ethoxycarbonyl)-2-methyl-1,3-phenylene)bis(oxy))diundecanoate (3ga)

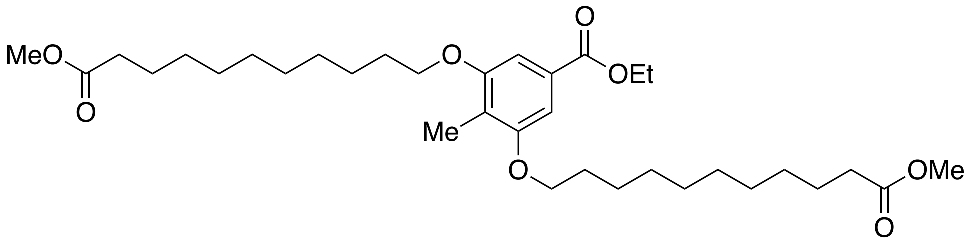

The product **3ga** was synthesized according to **Method A** using dimethyl 11,11'-((2-methyl-1,3-phenylene)bis(oxy))diundecanoate **1g** (156.2 mg, 0.3 mmol) amd 2-(ethoxycarbonyl)-1,3-dimethyl-1*H*-imidazol-3-ium trifluoromethanesulfonate **2a** (63.7 mg, 0.2 mmol). The product was purified by preparative thin-layer chromatography (85:15, hexane/AcOEt) to give **3ga** as white solid (25.7 mg, 0.043 mmol, 22% isolated yield).

M.p. 42–43 °C

IR (neat) 2924.4, 1739.4, 1716.4, 1422.0, 1315.7, 1235.2, 1190.7, 1170.6 cm^{-1}.

^{1}H NMR (400 MHz, $CDCl_3$) δ 7.18 (s, 2H), 4.37 (q, J = 7.2 Hz, 2H), 4.00 (t, J = 6.4 Hz, 4H), 3.67 (s, 6H), 2.31 (t, J = 8.0 Hz, 4H), 2.14 (s, 3H), 1.80 (tt, J = 7.6, 6.4 Hz, 4H), 1.62 (tt, J = 8.0, 7.2 Hz, 4H), 1.47 (tt, J = 9.0, 7.6 Hz, 4H), 1.40 (t, J = 7.2 Hz, 3H), 1.37–1.25 (m, 20H).

^{13}C NMR (100.6 MHz, $CDCl_3$) δ 174.3, 166.9, 157.5, 128.3, 120.6, 105.3, 68.4, 60.9, 51.4, 34.1, 29.5, 29.34, 29.30, 29.26, 29.2, 29.1, 26.1, 24.9, 14.4, 8.8.

HRMS–ESI (*m/z*): $[M+H]^+$ calcd for $C_{34}H_{57}O_8^+$, 593.4048; found, 593.4038.

Ethyl 3,5-Dimethoxybenzoate (3ha)

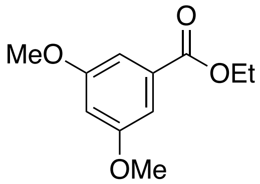

The product **3ha** was synthesized according to **Method A** using 1,3-dimethoxybenzene (**1h**) (38.7 μL, 0.3 mmol) and 2-(ethoxycarbonyl)-1,3-dimethyl-1*H*-imidazol-3-ium trifluoromethanesulfonate **2a** (63.7 mg, 0.2 mmol). The product was purified by flash chromatography on silica gel (100:0–97:3, hexane/AcOEt) to give **3ha** as colorless oil (5.5 mg, 0.026 mmol, 13% isolated yield).

^{1}H NMR (400 MHz, $CDCl_3$) δ 7.20 (d, J = 2.4 Hz, 2H), 6.65 (t, J = 2.4 Hz, 1H), 4.37 (q, J = 7.2

Hz, 2H), 3.83 (s, 6H), 1.39 (t, J = 7.2 Hz, 3H).

^{13}C NMR (100.6 MHz, $CDCl_3$) δ 166.4, 160.6, 132.4, 107.1, 105.5, 61.2, 55.6, 14.3.
The ^{1}H and ^{13}C NMR spectra data of product **3ha** were consistent with the literature.[13]

Ethyl 3-Methoxybenzoate (3ia)

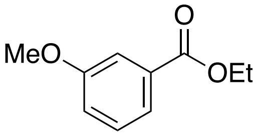

The product **3ia** was synthesized according to **Method A** on a 0.5 mmol scale (photo irradiation for 63 h), using anisole **1i** (81.1 μL, 0.75 mmol) and 2-(ethoxycarbonyl)-1,3-dimethyl-1*H*-imidazol-3-ium trifluoromethanesulfonate **2a** (159 mg, 0.5 mmol). The product was purified by preparative thin-layer chromatography (85:15, hexane/AcOEt) to give **3ia** as colorless oil (1.2 mg, 0.007 mmol, 1% isolated yield, 12% NMR yield).

^{1}H NMR (400 MHz, $CDCl_3$) δ 7.64 (ddd, J = 8.0, 1.6, 0.8 Hz, 1H), 7.57 (dd, J = 2.8, 1.6 Hz, 1H), 7.35 (dd, J = 8.0, 8.0 Hz, 1H), 7.10 (ddd, J = 8.0, 2.8, 0.8 Hz, 1H), 4.38 (q, J = 7.2 Hz, 2H), 3.86 (s, 3H), 1.40 (t, J = 7.2 Hz, 3H).

^{13}C NMR (100.6 MHz, $CDCl_3$) δ 166.5, 159.5, 131.8, 129.3, 121.9, 119.3, 114.0, 61.1, 55.4, 14.3.
The ^{1}H and ^{13}C NMR spectra data of product **3ia** were consistent with the literature.[14]

6. Synthetic Procedures for the Larger Scale Reaction

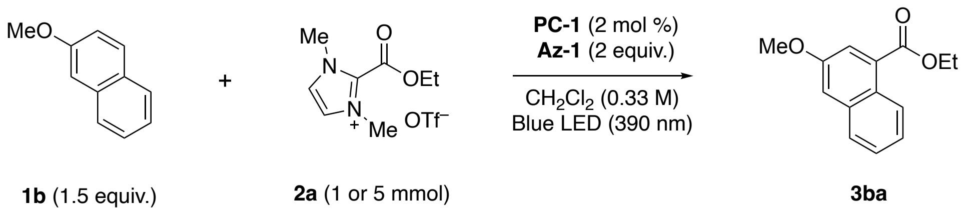

5 mmol scale

2-methoxynaphthalene **1b** (1.19 g, 7.5 mmol), **PC-1** (133 mg, 0.1 mmol) and imidazolium ester **2a** (1.59 g, 5.0 mmol) were placed in an oven-dried screw-top 20 mL vial containing a magnetic stirring bar. The vial was then introduced in the nitrogen-filled glovebox and to this was added imidazole **Az-1** (681 mg, 10 mmol) and 15 mL anhydrous dichloromethane. The vial was then brought outside the glovebox. After 48 h stirring at ambient temperature under photoirradiation (390 nm), the reaction mixture was evaporated under reduced pressure. The product was purified by flash column chromatography on silica gel (100:0–95:5, hexane/AcOEt). The product **3ba** was obtained as white solid (716 mg, 3.1 mmol, 62% isolated yield, contains 4% inpurity).

1 mmol scale

2-methoxynaphthalene **1b** (0.24 g, 1.5 mmol), **PC-1** (27 mg, 20 μmol) and imidazolium ester **2a** (0.32 g, 1.0 mmol) were placed in an oven-dried screw-top 10 mL vial containing a magnetic stirring bar. The vial was then introduced in the nitrogen-filled glovebox and to this was added imidazole **Az-1** (0.14 g, 2.0 mmol) and 3 mL anhydrous dichloromethane. The vial was then brought outside the glovebox. After 24 h stirring at ambient temperature under photoirradiation (390 nm), the reaction mixture was evaporated under reduced pressure (68% ^{1}H-NMR yield).

7. Mechanistic Study

7-1. TEMPO Trapping

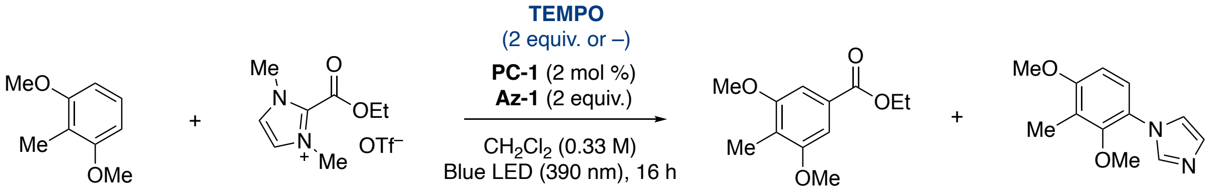

1a 2a 3aa 3aa’

	3aa	3aa’
TEMPO (–)	78%	2%
TEMPO (+)	0%	31%

Arene **1a** (22.8 mg, 0.15 mmol), **PC-1** (2.7 mg, 2 μmol), and imidazolium ester **2a** (31.8 mg, 0.1 mmol) were placed in an oven-dried screw-top 4 mL vial containing a magnetic stirring bar. The vial was then introduced in the nitrogen-filled glovebox and to this was added imidazole **Az-1** (13.6 mg, 0.2 mmol), TEMPO (31.2 mg, 0.2 mmol), and 300 μL anhydrous dichloromethane. The vial was then brought outside the glovebox. After 16 h stirring at ambient temperature under photoirradiation (390 nm), the reaction mixture was evaporated under reduced pressure. The yields of **3aa** and **3aa’**were determined by ^{1}H-NMR.

7-2. Reaction Under Standard Conditions with Aryl Bromide

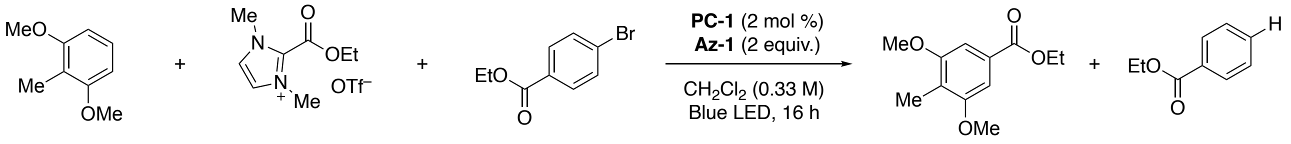

1a 2a Ar–Br 3aa Ar–H

	3aa	Ar–H
440 nm	0%	0%
390 nm	53%	18%

Arene **1a** (22.8 mg, 0.15 mmol), **PC-1** (2.7 mg, 2 μmol), and imidazolium ester **2a** (31.8 mg, 0.1 mmol) were placed in an oven-dried screw-top 4 mL vial containing a magnetic stirring bar. The vial was then introduced in the nitrogen-filled glovebox and to this was added imidazole **Az-1** (13.6 mg, 0.2 mmol), ethyl 4-bromobenzoate **Ar–Br** (16.0 μL, 0.1 mmol), and 300 μL anhydrous dichloromethane. The vial was then brought outside the glovebox. After 16 h stirring at ambient temperature under photoirradiation (440 nm or 390 nm), the reaction mixture was evaporated under reduced pressure. The yields of **3aa** and **Ar–H** were determined by ^{1}H-NMR.

7-3. Monitoring of the Reaction Intermediates

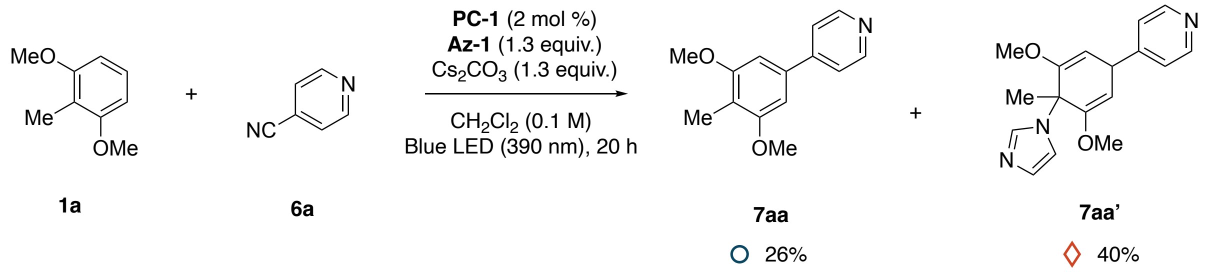

Arene **1a** (30.4 mg, 0.2 mmol) and **PC-1** (5.7 mg, 4 µmol) were placed in an oven-dried screw-top 4 mL vial containing a magnetic stirring bar. The vial was then introduced in the nitrogen-filled glovebox and to this was added imidazole **Az-1** (17.8 mg, 0.26 mmol), cyanoarene **6a** (41.6 mg, 0.4 mmol), cesium carbonate (84.8 mg, 0.26 mmol), and 2 mL anhydrous dichloromethane. The vial was then brought outside the glovebox. After 20 h stirring at ambient temperature under photoirradiation (390 nm), water added to the reaction mixture (1.5 mL). The organic layer was extracted with AcOEt (1.5 mL, 3 times). The combined organic layer was dried by Na_2SO_4 and filtrated. After concentration of organic layer, the crude mixture was monitored by ^{1}H-NMR and the yields of **7aa** and **7aa'** were determined.

HRMS–ESI (*m/z*): $[M+H]^+$ calcd for $C_{17}H_{19}N_3O_2^+$, 298.1550; found, 298.1547.

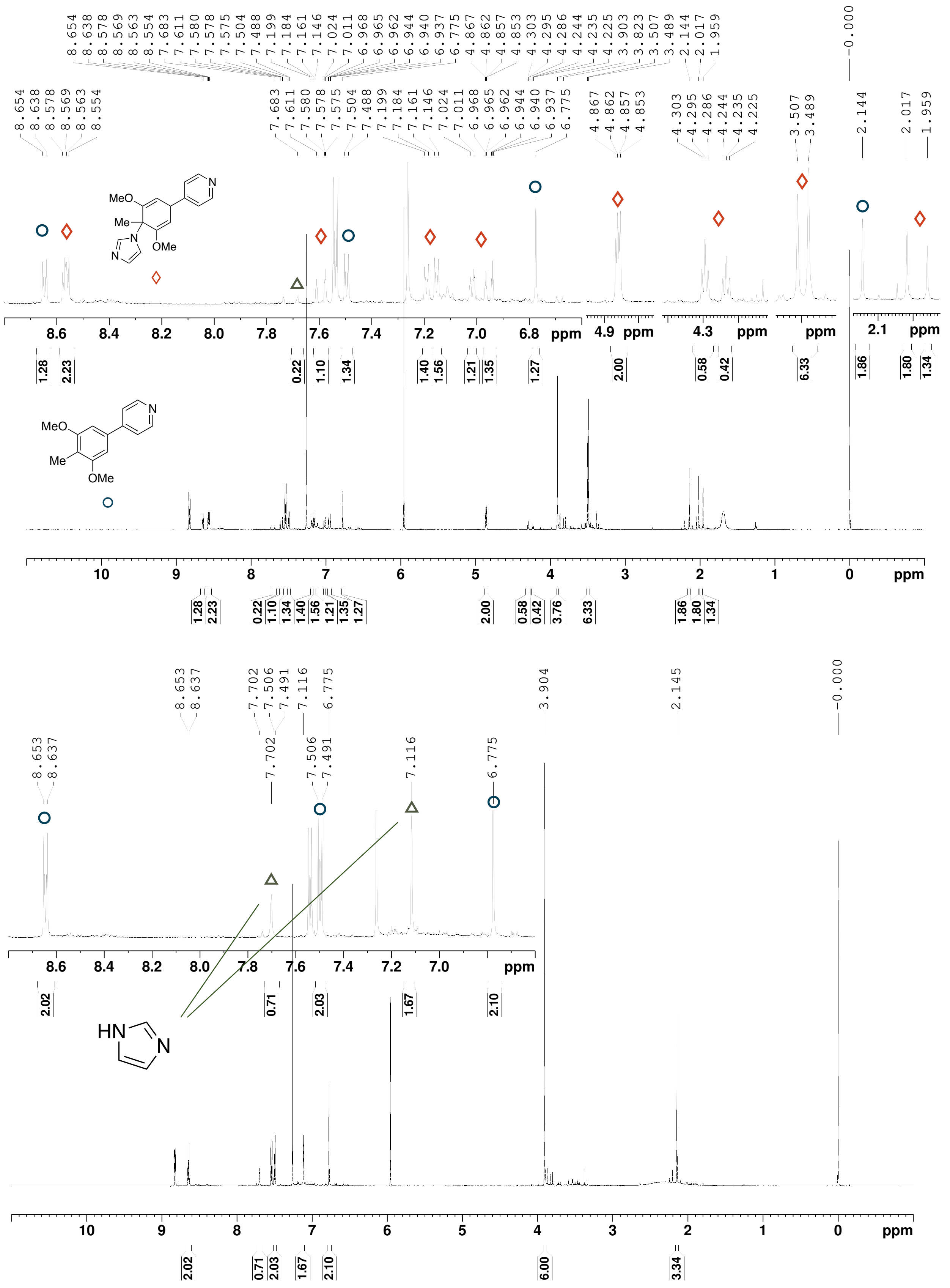

Supplementary Fig. 4. ^{1}H NMR spectra ($CDCl_3$) of the reaction mixture.
After photo irradiation (above) and after another 16 h (below)

7-4. Light Intensity-Dependent Product Yield Over Time

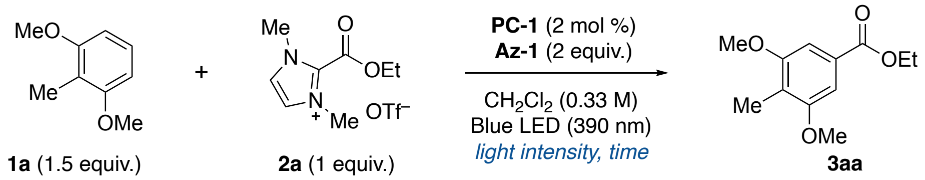

PC-1 (2.7 mg, 2.0 μmol) and imidazolium ester **2a** (0.10 mmol), arene **1a** (0.15 mmol) were placed in an oven-dried screw-top 4 mL vial containing a magnetic stirring bar. The vial was then introduced in the nitrogen-filled glovebox and to this was added imidazole **Az-1** (13.6 mg, 0.20 mmol), and 300 μL anhydrous dichloromethane. The vial was then brought outside the glovebox. After stirring at ambient temperature under photoirradiation (390 nm, 25% or 100% intensity), the reaction mixture was passed through a short silica pad using Et_2O as the eluent and then evaporated under reduced pressure. The yield of **3aa** was determined by ^{1}H-NMR.

light intensity/ reaction time (min)	15	30	60	90
100%	8	11	27	52
25%	3	2	9	13

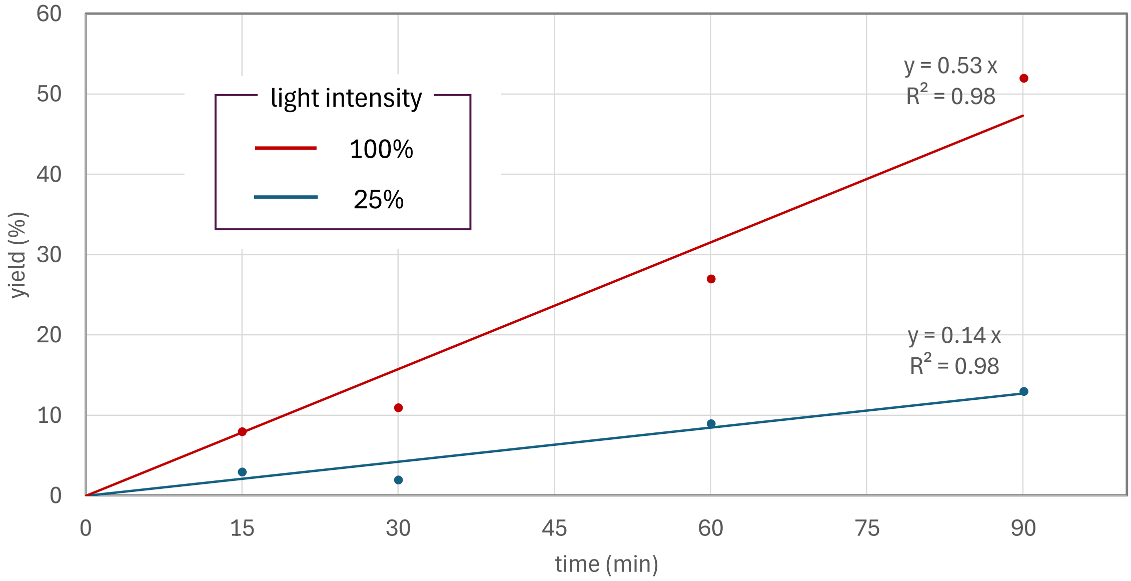

Supplementary Fig. 5. Light intensity-dependent product yield over time.

7-5. Intrinsic Regioselectivity Observed for 1a and 1b in Typical Friedel–Crafts Reactions

♦ Acylation

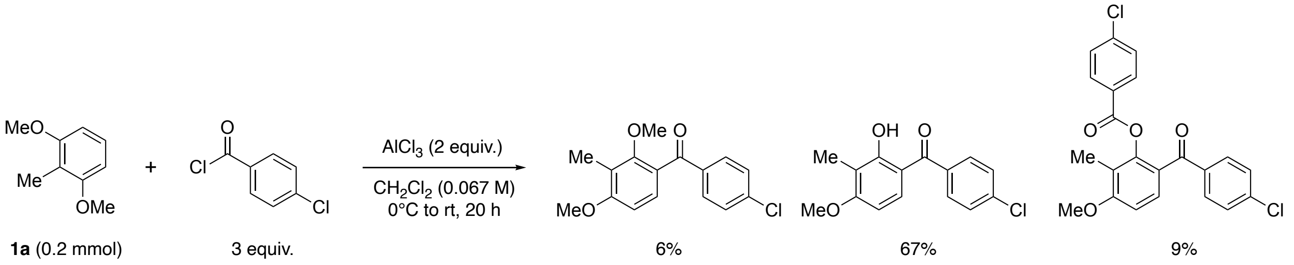

Arene **1a** (30.4 mg, 0.2 mmol) and $AlCl_3$ (53.3 mg, 0.4 mmol), anhydrous dichloromethane (3.0 mL) were placed in an oven-dried screw-top 10 mL vial containing a magnetic stirring bar. The reaction mixture was cooled to 0°C, and 4-chlorobenzoyl chloride (76.6 μL, 0.6 mmol) was added in one portion. The mixture was then stirred at room temperature for a total of 20 h. Subsequently, the reaction mixture was poured into 1 M HCl (4 mL). The organic layer was extracted with dichloromethane (3 mL, 3 times). The combined organic layers were dried over Na_2SO_4 and filtered. After concentrating the organic layer, the crude mixture was analyzed by ^{1}H-NMR, and the yields of the products were determined.

Comment: The results demonstrated that the acyl group was selectively introduced at the C4 position, with no other isomers detected. Additionally, significant amounts of byproducts derived from the C4-substituted product were observed. These findings confirm that under Friedel–Crafts acylation conditions using **1a**, the acyl group is introduced at the C4 position with high regioselectivity.

The control experiments described below were conducted in the same manner.

♦ Ethoxycarbonylation

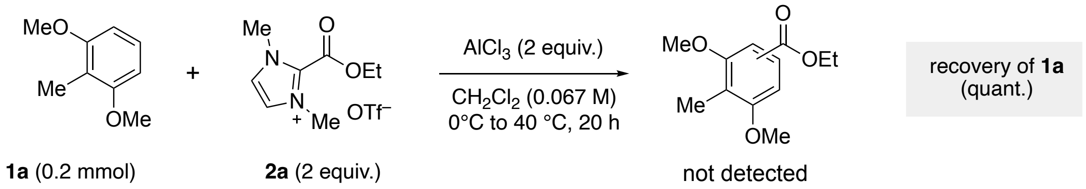

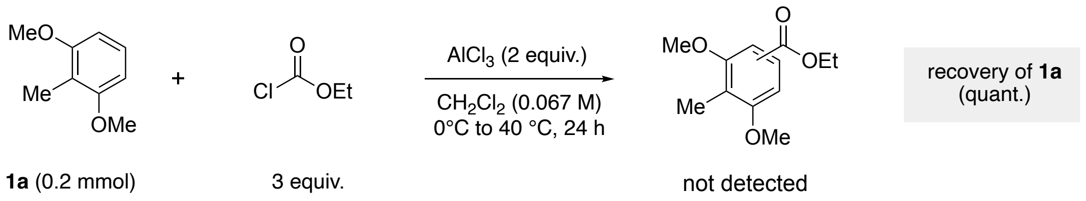

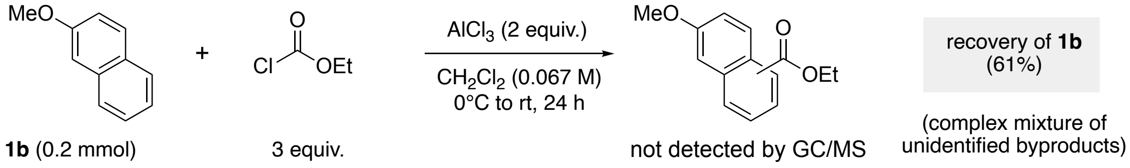

Comment: In the reaction using **1a**, despite prolonged heating at 40°C, no substituted product was formed. Even when a more reactive chloroformic acid ester was employed, the desired product was not obtained. In both cases, the starting arene was quantitatively recovered. In the reaction using **1b,** some of **1b** was consumed, resulting in a complex mixture that was difficult to analyze. Despite this, no product corresponding to the C–H ethoxycarbonylation product was detected in the GC/MS analysis.

♦ **Pyridylation**

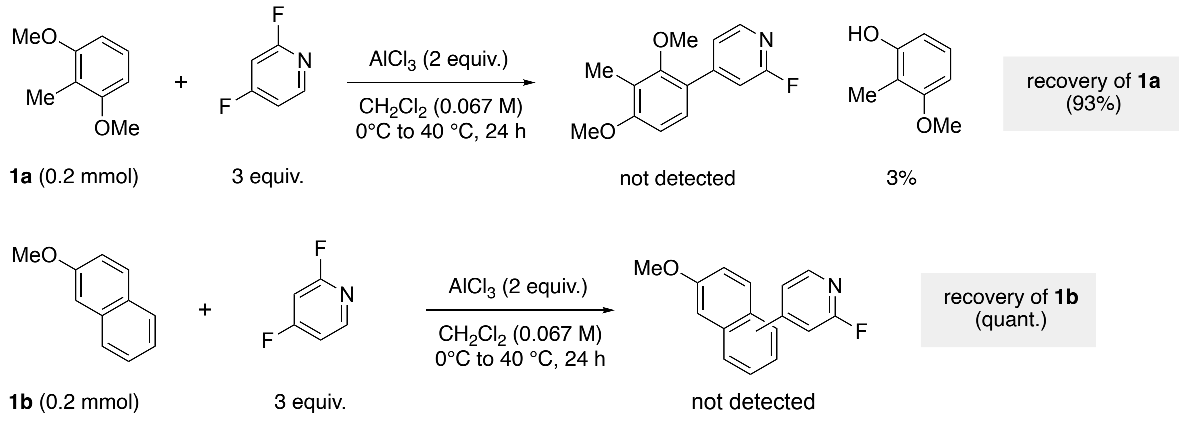

Comment: The desired product was not obtained. A small amount of demethylated **1a** was observed, while the majority of the starting material was recovered.

8. Unsuccessful Examples

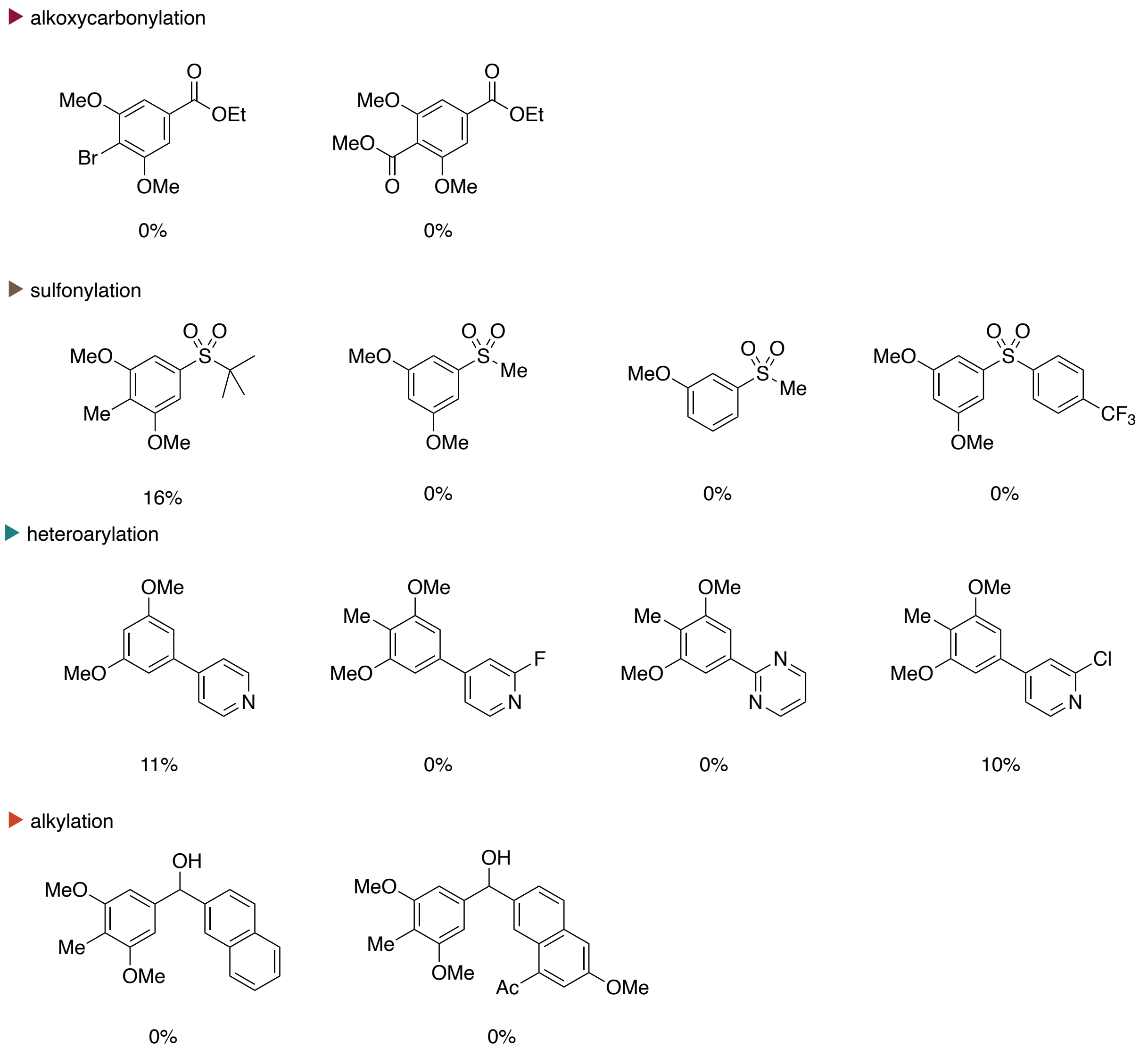

Supplementary Fig. 6. Unsuccessful examples.

9. Computational Study

Computational Method

The calculations were performed with Gaussian 16 packages (revision C.01).[15]

- NBO calculation

The DFT method was employed using the UωB97X-D functional using the def2-TZVPP basis set for NBO calculation. Natural population analyses (NPA) were performed using the NBO formalism and the values of positive charges were calculated in dichloromethane.

- RSE calculation

The DFT method was employed using the (U)ωB97X-D functional using the def2-TZVPP basis set for geometry optimization. The single-point energy calculations were then carried out with CBS-QB3.[16] Energies were adjusted to 0 K by adding a scaled (by 0.9779)[17] (U)ωB97X-D/def2-TZVPP zero-point vibrational energy (ZPVE).

The BDE values of C–H bonds in substrates were calculated according to the following equation.

$$\text{BDE (C–H)} = \Delta H_{\text{C radical}} + \Delta H_{\text{H radical}} - \Delta H_{\text{C–H}}$$

The RSEs of each radical were calculated according to the following equation.[18]

$$\text{RSE} = \text{BDE (H–CH}_3) - \text{BDE (H–CX}_3)$$

- TS calculation

(U)M06-2X level[19] with the 6-31+G(d,p) with SMD[20] model (dichloromethane) was employed for TS calculations. The IRC calculations were performed for each transitions state to confirm the transition state connecting the reaction pathway between the starting materials and the products.

♦ **NBO calculation**

Cartesian coordinates of radical cation

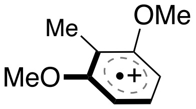

Center Number	Atomic Number	Atomic Type	Coordinates (Angstroms) X	Y	Z
1	6	0	1.188975	-0.018994	-0.022836
2	6	0	0.014614	2.127980	-0.032474
3	6	0	-1.179155	1.443382	-0.025252
4	6	0	-1.181179	0.000706	-0.020457
5	6	0	-0.001315	-0.738493	-0.041376
6	1	0	0.023071	3.207902	-0.039628
7	1	0	-2.114351	1.982415	-0.026258
8	8	0	-2.297112	-0.685237	0.005959
9	8	0	2.305737	-0.705644	0.003913
10	6	0	-3.578379	-0.043147	0.077677
11	1	0	-3.749613	0.569009	-0.806144
12	1	0	-4.298920	-0.853908	0.107535
13	1	0	-3.653879	0.552797	0.986045
14	6	0	3.586761	-0.065219	0.080567
15	1	0	4.306004	-0.877051	0.114617
16	1	0	3.764037	0.545587	-0.803064
17	1	0	3.659989	0.531699	0.988574
18	6	0	1.198692	1.425669	-0.031263
19	1	0	2.140007	1.953857	-0.039297
20	6	0	-0.042539	-2.235199	-0.051495
21	1	0	-0.457433	-2.617571	0.882565
22	1	0	-0.677937	-2.594440	-0.861198
23	1	0	0.951181	-2.653360	-0.181271

Natural Population Analysis

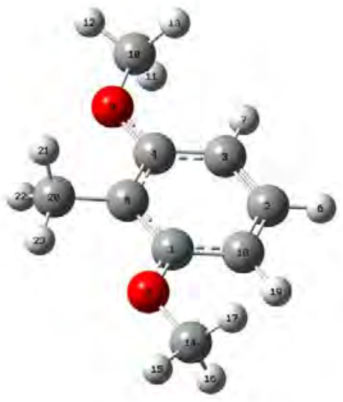

Atom	No	Natural Charge
C	1	0.40934
C	2	-0.24163
C	3	-0.10127
C	4	0.41068
C	5	-0.14772
H	6	0.25900
H	7	0.25510
O	8	-0.39331
O	9	-0.39793
C	10	-0.27865
H	11	0.20609
H	12	0.22229
H	13	0.20621
C	14	-0.27839
H	15	0.22204
H	16	0.20547
H	17	0.20564
C	18	-0.09846
H	19	0.25439
C	20	-0.63501
H	21	0.23783
H	22	0.23791
H	23	0.24039

♦ Radical stabilization energy

CBS-QB3//(U)ωB97X-D/def2-TZVPP						
radicals		ΔH $_{\text{C radical}}$ (hartree)	ΔH $_{\text{H radical}}$ (hartree)	ΔH $_{\text{C–H radical}}$ (hartree)	BDE (C–H) (kcal/mol)	RSE (kcal/mol)
(a)	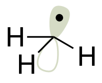	-39.744928	-0.499818	-40.410269	103.9	0
(b)	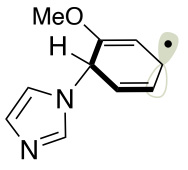	-571.352638	-0.499818	-571.968564	72.9	31.0
(c)	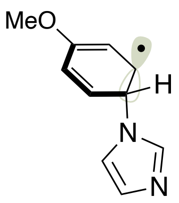	-571.348646	-0.499818	-571.968179	75.1	28.7
(d)	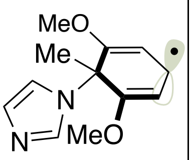	-724.948040	-0.499818	-725.562989	72.2	31.6
(e)	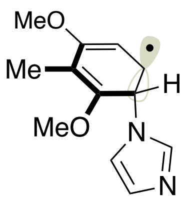	-724.939883	-0.499818	-725.559630	75.3	28.6
(f)	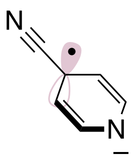	-339.975310	-0.499818	-340.553223	49.0	54.9
(g)	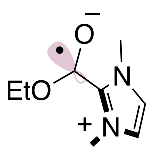	-571.630405	-0.499818	-572.211604	51.1	52.8
(h)	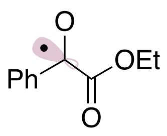	-611.810339	-0.499818	-612.390863	50.6	53.2

(i)	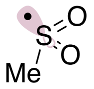	-587.808235	-0.499818	-588.421861	71.4	32.5

Supplementary Fig. 7. BDEs and RSEs of radicals.

Cartesian coordinates of (a) C radical

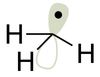

Center Number	Atomic Number	Atomic Type	Coordinates (Angstroms) X	Y	Z
1	6	0	0.000000	0.000000	0.000025
2	1	0	0.000000	1.078666	-0.000050
3	1	0	-0.934152	-0.539333	-0.000050
4	1	0	0.934152	-0.539333	-0.000050

Cartesian coordinates of (a) C–H

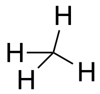

Center Number	Atomic Number	Atomic Type	Coordinates (Angstroms) X	Y	Z
1	6	0	0.000000	0.000000	0.000000
2	1	0	0.628159	0.628159	0.628159
3	1	0	-0.628159	-0.628159	0.628159
4	1	0	-0.628159	0.628159	-0.628159
5	1	0	0.628159	-0.628159	-0.628159

Cartesian coordinates of (b) C radical

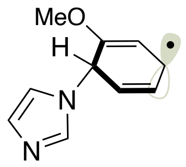

Center Number	Atomic Number	Atomic Type	Coordinates (Angstroms) X	Y	Z
1	6	0	-0.647068	-1.838849	-0.760241
2	6	0	-1.762524	-2.180023	-0.072018
3	6	0	-2.555693	-1.206954	0.570074
4	6	0	-2.215526	0.158449	0.483891
5	6	0	-1.108095	0.553037	-0.202819
6	1	0	-0.033575	-2.586499	-1.244438
7	1	0	-2.053808	-3.220658	-0.013969
8	1	0	-3.435637	-1.502156	1.122662
9	1	0	-2.852579	0.889231	0.960718
10	8	0	-0.698462	1.814427	-0.390120
11	6	0	-1.421196	2.855977	0.229232
12	1	0	-0.905608	3.776876	-0.026492
13	1	0	-2.448448	2.896366	-0.141652
14	1	0	-1.429969	2.731509	1.314556
15	6	0	-0.164423	-0.424381	-0.851056
16	1	0	-0.047470	-0.133562	-1.902068
17	7	0	1.178443	-0.294255	-0.280042
18	6	0	1.564622	-0.681880	0.977350
19	6	0	2.253566	0.302634	-0.850780
20	6	0	2.868684	-0.301573	1.091066
21	1	0	0.889534	-1.185205	1.646785
22	1	0	2.211623	0.713483	-1.847304
23	1	0	3.527675	-0.439419	1.932037
24	7	0	3.291825	0.312255	-0.058254

Cartesian coordinates of (b) C–H

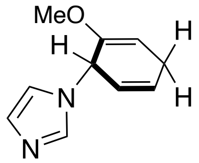

Center Number	Atomic Number	Atomic Type	Coordinates (Angstroms) X	Y	Z
1	6	0	-0.661355	-1.801296	-0.769754
2	6	0	-1.768762	-2.133896	-0.126686
3	6	0	-2.177516	0.267289	0.443566
4	6	0	-1.065239	0.603893	-0.203768
5	1	0	-0.051804	-2.555390	-1.252113
6	1	0	-2.072965	-3.173392	-0.093590
7	1	0	-2.800294	1.028171	0.892827
8	8	0	-0.587540	1.852589	-0.384512
9	6	0	-1.275585	2.916825	0.226633
10	1	0	-0.716518	3.818187	-0.008181
11	1	0	-2.293385	3.006778	-0.163990
12	1	0	-1.316658	2.785076	1.311295
13	6	0	-0.143201	-0.395948	-0.853082
14	1	0	-0.022632	-0.109024	-1.901575
15	7	0	1.194515	-0.311685	-0.276940
16	6	0	1.565244	-0.705458	0.983024
17	6	0	2.292818	0.238514	-0.849954
18	6	0	2.883255	-0.375918	1.095361
19	1	0	0.871978	-1.176372	1.656926
20	1	0	2.266845	0.645245	-1.848658
21	1	0	3.535316	-0.533755	1.938243
22	7	0	3.330851	0.212997	-0.057331
23	6	0	-2.646367	-1.147937	0.568969
24	1	0	-2.732501	-1.426992	1.625408
25	1	0	-3.664386	-1.234836	0.173553

Cartesian coordinates of (c) C radical

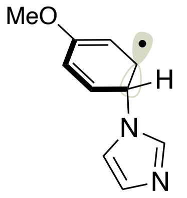

Center Number	Atomic Number	Atomic Type	Coordinates (Angstroms) X	Y	Z
1	6	0	0.123240	1.525727	-0.302609
2	6	0	0.301551	-0.382401	1.290885
3	6	0	1.575918	-0.592199	0.864842
4	6	0	2.151162	0.225525	-0.125889
5	6	0	1.389068	1.282661	-0.691848
6	1	0	-0.133812	-1.023152	2.045731
7	1	0	2.142908	-1.402108	1.301659
8	8	0	3.403766	0.092441	-0.605733
9	6	0	4.218672	-0.937565	-0.093332
10	1	0	3.783295	-1.921809	-0.284040
11	1	0	5.168770	-0.859383	-0.614288
12	1	0	4.385472	-0.816234	0.980050
13	6	0	-0.576143	0.696296	0.733001
14	1	0	-0.915957	1.347592	1.549037
15	7	0	-1.818814	0.130536	0.192691
16	6	0	-1.922824	-0.741713	-0.859053
17	6	0	-3.080094	0.356820	0.635372
18	6	0	-3.256266	-0.994858	-0.993772
19	1	0	-1.057025	-1.086453	-1.396305
20	1	0	-3.281611	1.015067	1.466369
21	1	0	-3.739543	-1.634406	-1.713453
22	7	0	-3.972863	-0.303214	-0.054322
23	1	0	-0.446654	2.328794	-0.750606
24	1	0	1.860067	1.891536	-1.452460

Cartesian coordinates of (c) C–H

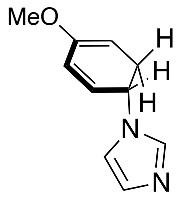

Center Number	Atomic Number	Atomic Type	Coordinates (Angstroms) X	Y	Z
1	6	0	-0.117300	-1.457517	-1.103535
2	6	0	-1.425481	-0.169165	1.079683
3	6	0	-1.946477	-0.158965	-0.150525
4	6	0	-1.327385	-0.924962	-1.233687
5	1	0	-1.864667	0.387400	1.893731
6	8	0	-3.038782	0.521570	-0.565879
7	6	0	-3.711824	1.301299	0.392879
8	1	0	-3.057298	2.077905	0.799065
9	1	0	-4.550124	1.765145	-0.119540
10	1	0	-4.083059	0.681815	1.214455
11	6	0	0.676275	-1.284246	0.165524
12	1	0	1.250498	-2.191456	0.354103
13	7	0	1.675899	-0.234764	-0.007088
14	6	0	1.474976	1.048477	-0.447597
15	6	0	2.987568	-0.322872	0.332794
16	6	0	2.687984	1.663743	-0.352222
17	1	0	0.511909	1.389418	-0.781841
18	1	0	3.413692	-1.237428	0.715790
19	1	0	2.936564	2.679213	-0.611939
20	7	0	3.629306	0.797199	0.135941
21	6	0	-0.236717	-1.038215	1.373531
22	1	0	0.361566	-0.612897	2.179816
23	1	0	-0.587547	-2.014082	1.727873
24	1	0	0.355222	-1.973974	-1.928600
25	1	0	-1.882649	-1.006130	-2.158921

Cartesian coordinates of (d) C radical

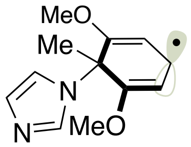

Center Number	Atomic Number	Atomic Type	Coordinates (Angstroms) X	Y	Z
1	6	0	-1.247583	-0.889729	0.107693
2	6	0	-1.222968	-1.996900	-0.681266
3	6	0	0.000230	-2.563681	-1.087710
4	6	0	1.223332	-1.996685	-0.681277
5	6	0	1.247759	-0.889509	0.107682
6	1	0	-2.145790	-2.455189	-1.005314
7	1	0	0.000304	-3.441083	-1.716882
8	1	0	2.146232	-2.454810	-1.005334
9	8	0	2.351424	-0.262952	0.551771
10	6	0	3.606325	-0.726577	0.112729
11	1	0	4.346449	-0.062828	0.550488
12	1	0	3.790065	-1.750314	0.449829
13	1	0	3.678624	-0.685275	-0.977054
14	6	0	0.000028	-0.178852	0.587975
15	7	0	-0.000102	1.181496	0.013750
16	6	0	-0.000154	1.446151	-1.332681
17	6	0	-0.000294	2.384762	0.641083
18	6	0	-0.000219	2.803346	-1.443258
19	1	0	-0.000078	0.655273	-2.062388
20	1	0	-0.000347	2.480376	1.712856
21	1	0	-0.000228	3.395167	-2.343508
22	7	0	-0.000342	3.382957	-0.203151
23	8	0	-2.351355	-0.263363	0.551788
24	6	0	-3.606175	-0.727172	0.112711
25	1	0	-4.346409	-0.063530	0.550446
26	1	0	-3.678447	-0.685885	-0.977074
27	1	0	-3.789776	-1.750935	0.449809
28	6	0	0.000029	-0.102280	2.121973
29	1	0	-0.890391	0.408822	2.480107
30	1	0	0.000097	-1.116619	2.517331
31	1	0	0.890384	0.408939	2.480100

Cartesian coordinates of (d) C–H

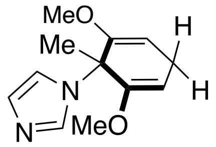

Center Number	Atomic Number	Atomic Type	Coordinates (Angstroms) X	Y	Z
1	6	0	-1.251838	-0.867468	0.119090
2	6	0	-1.250786	-1.979599	-0.608423
3	6	0	1.250666	-1.979671	-0.608413
4	6	0	1.251778	-0.867540	0.119100
5	1	0	-2.184615	-2.434523	-0.907248
6	1	0	2.184471	-2.434650	-0.907230
7	8	0	2.344792	-0.198769	0.544876
8	6	0	3.605355	-0.656445	0.124248
9	1	0	4.334840	0.036615	0.534028
10	1	0	3.808538	-1.663387	0.500510
11	1	0	3.677539	-0.658780	-0.966913
12	6	0	-0.000011	-0.153078	0.591377
13	7	0	0.000041	1.194626	0.004777
14	6	0	0.000111	1.453749	-1.342537
15	6	0	0.000126	2.400707	0.626065
16	6	0	0.000034	2.810354	-1.459148
17	1	0	0.000132	0.660941	-2.069401
18	1	0	0.000155	2.500614	1.697397
19	1	0	0.000000	3.397736	-2.362321
20	7	0	0.000079	3.395621	-0.221860
21	8	0	-2.344815	-0.198636	0.544865
22	6	0	-3.605405	-0.656254	0.124255
23	1	0	-4.334852	0.036839	0.534046
24	1	0	-3.677606	-0.658584	-0.966905
25	1	0	-3.808629	-1.663187	0.500518
26	6	0	-0.000016	-0.074073	2.121828
27	1	0	-0.889960	0.437035	2.480745
28	1	0	0.000015	-1.088476	2.517253
29	1	0	0.889894	0.437094	2.480746
30	6	0	-0.000078	-2.664561	-1.048493
31	1	0	-0.000108	-3.695413	-0.676795
32	1	0	-0.000077	-2.759072	-2.140470

Cartesian coordinates of (e) C radical

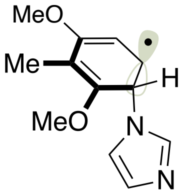

Center Number	Atomic Number	Atomic Type	X	Y	Z
1	6	0	-0.110526	1.016124	-0.200375
2	6	0	-0.244913	-1.218690	-1.296197
3	6	0	-1.526031	-1.328386	-0.855553
4	6	0	-2.112504	-0.308574	-0.091319
5	6	0	-1.397248	0.887832	0.216555
6	1	0	0.208651	-2.007863	-1.879557
7	1	0	-2.089973	-2.213874	-1.110296
8	8	0	-3.375212	-0.353007	0.387825
9	8	0	0.578855	2.126739	0.163448
10	6	0	-4.163361	-1.488977	0.121716
11	1	0	-3.712548	-2.393818	0.537653
12	1	0	-5.121137	-1.312401	0.603621
13	1	0	-4.320578	-1.623624	-0.951705
14	6	0	1.558160	2.642198	-0.715924
15	1	0	1.852114	3.604825	-0.305991
16	1	0	2.439156	1.999796	-0.766883
17	1	0	1.149776	2.794059	-1.718873
18	6	0	0.634353	-0.066129	-0.934887
19	1	0	1.085252	0.347191	-1.845126
20	7	0	1.790578	-0.521526	-0.141867
21	6	0	1.771470	-0.941522	1.161090
22	6	0	3.061421	-0.689222	-0.585184
23	6	0	3.047908	-1.333391	1.438282
24	1	0	0.872814	-0.911767	1.751486
25	1	0	3.349088	-0.439723	-1.595233
26	1	0	3.435755	-1.717503	2.366914
27	7	0	3.848853	-1.172293	0.338958
28	6	0	-2.090064	1.967516	0.995957
29	1	0	-3.039657	2.228302	0.529045
30	1	0	-2.317534	1.625989	2.006870
31	1	0	-1.468327	2.854625	1.063289

Cartesian coordinates of (e) C–H

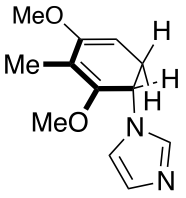

Center Number	Atomic Number	Atomic Type	Coordinates (Angstroms) X	Y	Z
1	6	0	-0.044262	1.232983	-0.166465
2	6	0	-1.410889	-1.071914	-1.141847
3	6	0	-1.939535	-0.276399	-0.206688
4	6	0	-1.313704	0.994154	0.193953
5	1	0	-1.863434	-2.007344	-1.430003
6	8	0	-3.072120	-0.524155	0.491180
7	8	0	0.586091	2.322626	0.339229
8	6	0	-3.767183	-1.710440	0.197843
9	1	0	-3.148939	-2.590207	0.398399
10	1	0	-4.638979	-1.724712	0.846485
11	1	0	-4.088963	-1.731507	-0.847351
12	6	0	1.783260	2.784239	-0.247340
13	1	0	2.048939	3.688198	0.294241
14	1	0	2.595178	2.061493	-0.141538
15	1	0	1.644836	3.035726	-1.302686
16	6	0	0.743647	0.247722	-0.994242
17	1	0	1.404075	0.785638	-1.673088
18	7	0	1.631444	-0.533056	-0.137174
19	6	0	1.328100	-1.147939	1.049726
20	6	0	2.917727	-0.862518	-0.423707
21	6	0	2.459945	-1.813215	1.417897
22	1	0	0.360468	-1.048314	1.507053
23	1	0	3.408555	-0.507817	-1.317367
24	1	0	2.618278	-2.410327	2.300288
25	7	0	3.450267	-1.628047	0.490051
26	6	0	-2.090860	1.934366	1.065611
27	1	0	-3.083659	2.110179	0.651911
28	1	0	-2.233653	1.513244	2.062111
29	1	0	-1.572156	2.883275	1.165431
30	6	0	-0.178481	-0.616840	-1.856196
31	1	0	0.397017	-1.465101	-2.227487

32	1	0	-0.457909	-0.017671	-2.731091

Cartesian coordinates of (f) C radical

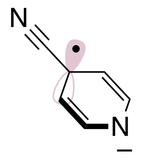

Center Number	Atomic Number	Atomic Type	Coordinates (Angstroms) X	Y	Z
1	6	0	-1.494636	-1.138361	-0.000010
2	6	0	-0.130739	-1.207854	0.000000
3	6	0	0.646933	-0.000012	0.000005
4	6	0	-0.130718	1.207843	0.000001
5	6	0	-1.494616	1.138376	-0.000009
6	7	0	-2.236601	0.000014	-0.000016
7	1	0	-2.065335	-2.064854	-0.000014
8	1	0	0.362148	-2.172059	0.000005
9	1	0	0.362185	2.172040	0.000007
10	1	0	-2.065299	2.064879	-0.000011
11	6	0	2.039172	-0.000027	0.000012
12	7	0	3.207161	0.000015	0.000017

Cartesian coordinates of (f) C–H

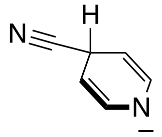

Center Number	Atomic Number	Atomic Type	Coordinates (Angstroms) X	Y	Z
1	6	0	-1.406312	-1.122461	-0.169992
2	6	0	-0.179294	-1.224089	0.404193
3	6	0	-0.179117	1.223962	0.404292
4	6	0	-1.406050	1.122714	-0.169857
5	7	0	-2.079405	0.000141	-0.511400
6	1	0	-1.937592	-2.054748	-0.367398
7	1	0	0.232493	-2.198126	0.639371
8	1	0	0.232962	2.197781	0.639931
9	1	0	-1.937278	2.055048	-0.367078
10	6	0	1.927599	-0.000114	-0.112288
11	7	0	2.915371	0.000130	-0.704081
12	6	0	0.663839	-0.000281	0.683601
13	1	0	1.033661	-0.000236	1.723840

Cartesian coordinates of (g) C radical

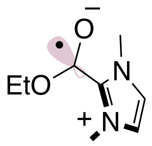

Center Number	Atomic Number	Atomic Type	Coordinates (Angstroms) X	Y	Z
1	8	0	0.610494	-1.991210	-0.047858
2	6	0	-0.691349	-0.040652	-0.003610
3	6	0	-2.267914	1.561977	0.017112
4	1	0	-2.670478	2.558350	0.024696
5	7	0	-0.908184	1.332188	0.014538
6	6	0	0.087116	2.374955	-0.039613
7	1	0	0.771479	2.307109	0.803359
8	1	0	0.670850	2.314643	-0.957571
9	1	0	-0.429153	3.332062	-0.007243
10	6	0	0.520060	-0.764501	-0.013896
11	7	0	-1.951208	-0.621402	-0.007099
12	6	0	-2.242013	-2.035697	0.023216
13	1	0	-1.845321	-2.496013	0.926673
14	1	0	-3.323630	-2.154229	-0.001008
15	1	0	-1.797841	-2.541415	-0.830872
16	6	0	-2.899374	0.375249	0.009155
17	1	0	-3.950826	0.153058	0.014660
18	6	0	2.877852	-0.627885	-0.005285
19	1	0	2.947464	-1.238770	-0.907334
20	1	0	2.946894	-1.304247	0.848657
21	8	0	1.626388	0.045127	0.019395
22	6	0	3.962116	0.423606	0.034351
23	1	0	4.942544	-0.052947	0.016819
24	1	0	3.890143	1.089823	-0.825690
25	1	0	3.889591	1.023419	0.941905

Cartesian coordinates of (g) C–H

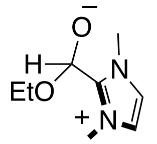

Center Number	Atomic Number	Atomic Type	Coordinates (Angstroms) X	Y	Z
1	8	0	0.708671	-1.737731	-1.044344
2	6	0	-0.732158	-0.012765	-0.218943
3	6	0	-2.428284	1.299300	0.345795
4	1	0	-2.960763	2.222665	0.481676
5	7	0	-1.149911	1.257080	-0.157901
6	6	0	-0.355371	2.406874	-0.556647
7	1	0	0.667145	2.250992	-0.223404
8	1	0	-0.375445	2.522784	-1.639096
9	1	0	-0.771456	3.294257	-0.087015
10	7	0	-1.725100	-0.774026	0.239128
11	6	0	-1.689798	-2.225937	0.391266
12	1	0	-1.635699	-2.471612	1.450199
13	1	0	-2.596476	-2.644205	-0.041302
14	1	0	-0.793935	-2.568030	-0.132172
15	6	0	-2.787397	0.023603	0.597594
16	1	0	-3.695068	-0.387921	0.999201
17	6	0	2.877588	-0.190616	-0.081433
18	1	0	3.140651	0.279575	-1.041706
19	1	0	3.027310	-1.266801	-0.198455
20	8	0	1.528824	0.070058	0.223202
21	6	0	3.743721	0.361026	1.029446
22	1	0	4.797500	0.170464	0.820837
23	1	0	3.605053	1.438065	1.137378
24	1	0	3.490158	-0.111676	1.978669
25	6	0	0.627441	-0.486995	-0.808202
26	1	0	0.791702	0.184520	-1.697507

Cartesian coordinates of (h) C radical

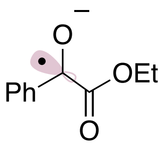

Center Number	Atomic Number	Atomic Type	Coordinates (Angstroms) X	Y	Z
1	6	0	-4.467575	-0.261421	0.786320
2	1	0	-5.095396	0.026986	-0.058351
3	1	0	-4.438944	0.573993	1.487373
4	1	0	-4.929075	-1.118798	1.281951
5	6	0	-3.070976	-0.618557	0.313836
6	1	0	-2.436722	-0.892086	1.159636
7	1	0	-3.084896	-1.474087	-0.360024
8	8	0	-2.547634	0.519055	-0.346345
9	6	0	-1.196067	0.783426	-0.385859
10	8	0	-0.898365	1.964656	-0.487827
11	8	0	-0.700889	-1.532645	-0.609727
12	6	0	1.157925	-0.141008	-0.122063
13	6	0	1.777466	1.094692	0.146290
14	6	0	1.985006	-1.280912	-0.124061
15	6	0	3.136517	1.170874	0.399086
16	1	0	1.174449	1.989130	0.143478
17	6	0	3.341263	-1.194811	0.127544
18	1	0	1.513963	-2.232523	-0.329344
19	6	0	3.936790	0.033607	0.393138
20	1	0	3.580414	2.139536	0.601830
21	1	0	3.944487	-2.096351	0.116783
22	1	0	5.000151	0.103612	0.588454
23	6	0	-0.275904	-0.357215	-0.380997

Cartesian coordinates of (h) C–H

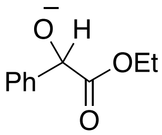

Center Number	Atomic Number	Atomic Type	Coordinates (Angstroms) X	Y	Z
1	6	0	-4.688969	-0.458567	-0.366849
2	1	0	-4.590221	-1.178236	-1.180649
3	1	0	-4.864225	0.525109	-0.802807
4	1	0	-5.557826	-0.733634	0.234538
5	6	0	-3.441260	-0.443631	0.488864
6	1	0	-3.255865	-1.423357	0.937859
7	1	0	-3.533005	0.271204	1.310297
8	8	0	-2.356304	-0.075670	-0.338835
9	6	0	-1.147233	0.036372	0.279090
10	8	0	-1.009631	-0.294651	1.432041
11	8	0	-0.301548	2.056436	-0.409176
12	6	0	1.277112	0.209114	-0.334546
13	6	0	2.230177	1.068873	0.187965
14	6	0	1.639216	-1.105823	-0.603888
15	6	0	3.521753	0.625978	0.439496
16	1	0	1.901568	2.084542	0.376436
17	6	0	2.926251	-1.557633	-0.353941
18	1	0	0.898113	-1.784462	-1.015945
19	6	0	3.876300	-0.689276	0.170596
20	1	0	4.258531	1.308192	0.849487
21	1	0	3.192519	-2.586999	-0.566881
22	1	0	4.884118	-1.036908	0.366263
23	6	0	-0.132615	0.757060	-0.625141
24	1	0	-0.358230	0.410835	-1.670709

Cartesian coordinates of (i) C radical

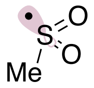

Center Number	Atomic Number	Atomic Type	Coordinates (Angstroms) X	Y	Z
1	16	0	0.204505	-0.000006	-0.268128
2	8	0	0.734273	-1.261571	0.201360
3	8	0	0.734478	1.261469	0.201369
4	6	0	-1.563375	0.000103	0.093190
5	1	0	-1.993732	0.900890	-0.334016
6	1	0	-1.994096	-0.899942	-0.335220
7	1	0	-1.654013	-0.000655	1.178319

Cartesian coordinates of (i) C–H

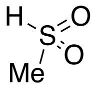

Center Number	Atomic Number	Atomic Type	Coordinates (Angstroms) X	Y	Z
1	8	0	-0.736578	-1.246536	-0.226547
2	8	0	-0.736882	1.246385	-0.226556
3	6	0	1.552356	0.000151	-0.109672
4	1	0	1.990011	0.900625	0.315026
5	1	0	1.990364	-0.899489	0.316444
6	1	0	1.666961	-0.000732	-1.192574
7	16	0	-0.186074	-0.000005	0.205018
8	1	0	-0.196599	-0.000014	1.563673

♦ TS calculation (nucleophilic addition)

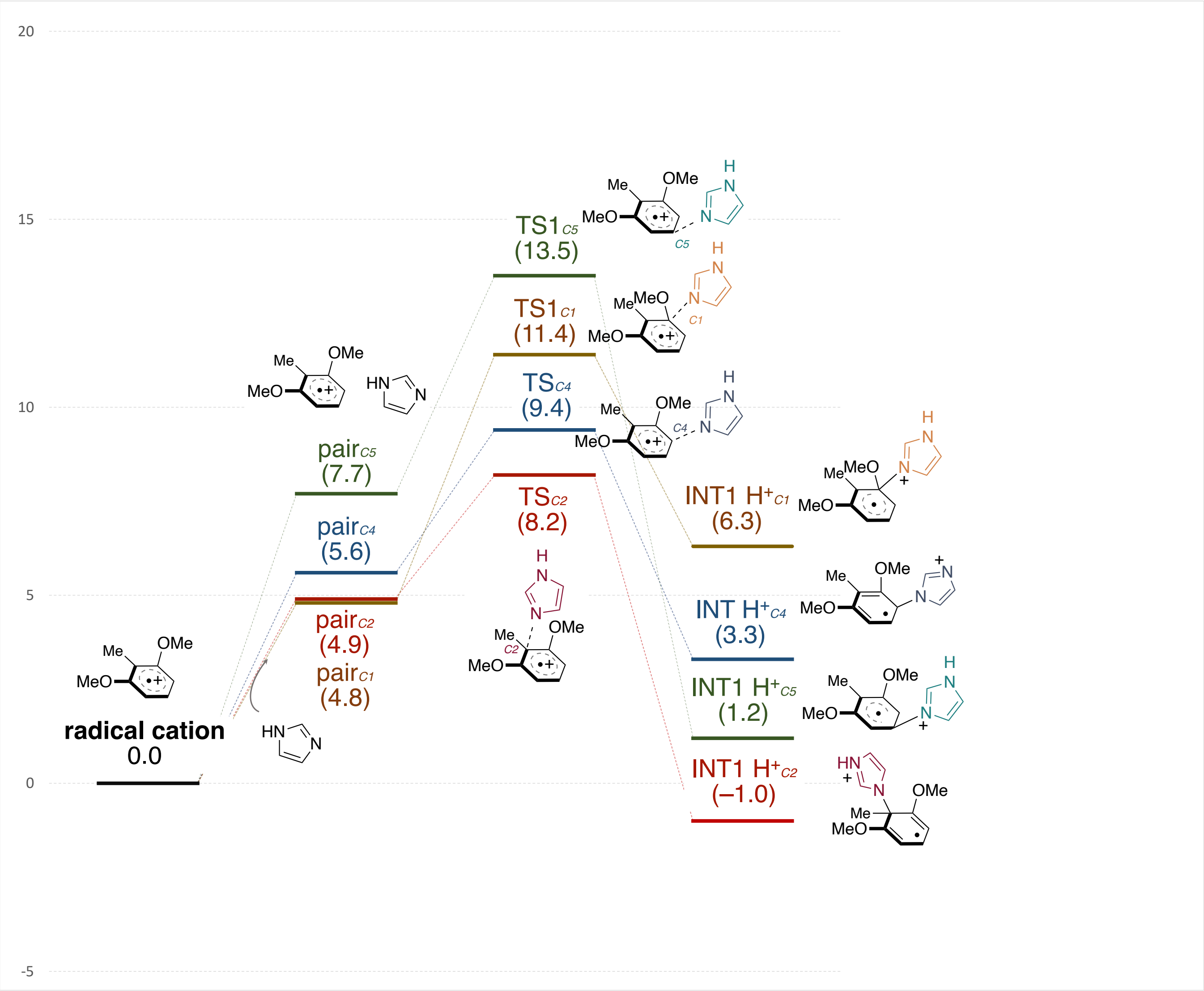

(U)M06-2X/6-31+G(d,p)/SMD (dichloromethane)			
	Free Energies (hartree)	Thermal Enthalpies (hartree)	Energies (hartree)
radical cation	-500.049290	-499.998900	-500.205320
imidazole	-226.110160	-226.079340	-226.156290
imidazole–H^+	-226.5469	-226.51593	-226.60661
pair_C2	-726.151600	-726.087010	-726.372950
TS1_C2	-726.146390	-726.087160	-726.372800

INT1 H^+_C2	-726.161000	-726.102390	-726.389570
pair_C4	-726.150580	-726.085640	-726.371400
TS1_C4	-726.144480	-726.083100	-726.368820
INT1 H^+_C4	-726.15425	-726.094320000	-726.38154
pair_C1	-726.15176	-726.08567	-726.37139
TS1_C1	-726.141350	-726.080050	-726.364190
INT1 H^+_C1	-726.149420	-726.089440	-726.375100
pair_C5	-726.147210	-726.080900	-726.366310
TS1_C5	-726.138020	-726.075830	-726.360910
INT1 H^+_C5	-726.157510000	-726.0968	-726.383780000

Supplementary Fig. 8. Total electronic energies, enthalpies and free energies.

Cartesian coordinates of radical cation

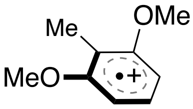

Center Number	Atomic Number	Atomic Type	Coordinates (Angstroms) X	Y	Z
1	6	0	1.203323	-0.022909	-0.022534
2	6	0	-0.018984	2.130692	-0.032977
3	6	0	-1.210713	1.464522	-0.033198
4	6	0	-1.191234	0.019362	-0.021756
5	6	0	0.015679	-0.734731	-0.037920
6	1	0	0.009172	3.214073	-0.039510
7	1	0	-2.152076	1.999340	-0.041436
8	8	0	-2.286080	-0.689759	0.009826
9	8	0	2.339589	-0.723254	-0.010238
10	6	0	-3.590546	-0.072486	0.080029
11	1	0	-3.764046	0.526216	-0.815329
12	1	0	-4.289187	-0.904568	0.120772
13	1	0	-3.662306	0.532348	0.985559
14	6	0	3.596934	-0.044074	0.085599
15	1	0	4.346407	-0.833105	0.108695
16	1	0	3.761667	0.593564	-0.786782
17	1	0	3.648287	0.541595	1.007791
18	6	0	1.194855	1.410421	-0.024757
19	1	0	2.129422	1.958021	-0.023871
20	6	0	-0.042319	-2.231259	-0.050680
21	1	0	-0.560966	-2.603683	0.838204
22	1	0	-0.597636	-2.583865	-0.925343
23	1	0	0.961216	-2.653057	-0.076290

Cartesian coordinates of imidazole

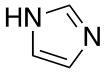

Center Number	Atomic Number	Atomic Type	Coordinates (Angstroms) X	Y	Z
1	6	0	0.599971	0.986823	-0.000074
2	6	0	1.141146	-0.273687	-0.000071
3	6	0	-0.986585	-0.543079	0.000049
4	7	0	-0.758084	0.793084	0.000045
5	1	0	-1.465162	1.517156	0.000130
6	1	0	1.042612	1.970851	-0.000147
7	1	0	2.186019	-0.549668	-0.000139
8	1	0	-1.982909	-0.960655	0.000108
9	7	0	0.142691	-1.221373	0.000045

Cartesian coordinates of imidazole–H$^+$

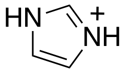

Center Number	Atomic Number	Atomic Type	Coordinates (Angstroms) X	Y	Z
1	6	0	0.675073	0.977651	-0.000262
2	6	0	-0.685887	0.970200	-0.000023
3	6	0	0.006193	-1.136486	-0.000312
4	7	0	1.070807	-0.341155	0.000307
5	1	0	2.032317	-0.669248	0.000304
6	1	0	1.383368	1.790587	-0.000246
7	1	0	-1.403469	1.774969	-0.000064
8	1	0	0.012669	-2.215533	-0.000709
9	7	0	-1.066863	-0.352795	0.000399
10	1	0	-2.024773	-0.691316	-0.000643

Cartesian coordinates of pair_C2

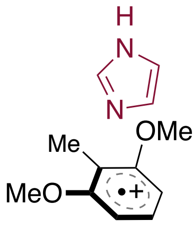

Center Number	Atomic Number	Atomic Type	Coordinates (Angstroms) X	Y	Z
1	6	0	-1.010713	1.346838	0.055293
2	6	0	-1.388561	1.373498	-1.277367
3	6	0	-1.692514	0.162716	-1.910290
4	6	0	-1.640681	-1.079472	-1.267220
5	6	0	-1.265078	-1.118341	0.065733
6	1	0	-1.454939	2.302533	-1.827975
7	1	0	-1.988356	0.189038	-2.954719
8	1	0	-1.896103	-1.980121	-1.810006
9	8	0	-1.184452	-2.214610	0.808140
10	8	0	-0.706350	2.410147	0.787850
11	6	0	-1.474420	-3.474571	0.193474
12	1	0	-0.777760	-3.660116	-0.628861
13	1	0	-1.339386	-4.219075	0.975501
14	1	0	-2.506186	-3.490623	-0.168228
15	6	0	-0.742609	3.699061	0.165502
16	1	0	-0.455497	4.405786	0.941399
17	1	0	-0.028995	3.736842	-0.662490
18	1	0	-1.753047	3.919513	-0.190367
19	6	0	-0.907041	0.093480	0.770259
20	7	0	1.433472	-0.158400	0.093350
21	6	0	2.480302	-0.214179	0.891961
22	6	0	1.911927	-0.239191	-1.191848
23	1	0	2.462090	-0.176343	1.971801
24	6	0	3.278005	-0.346374	-1.160977
25	1	0	1.252966	-0.216581	-2.049082
26	1	0	4.017426	-0.431920	-1.942067
27	6	0	-0.636551	0.071603	2.229252
28	1	0	-0.006439	0.911795	2.520361
29	1	0	-1.591468	0.166544	2.764681
30	1	0	-0.180719	-0.872973	2.525449
31	7	0	3.616020	-0.328766	0.169049

32	1	0	4.553989	-0.388842	0.547272

Cartesian coordinates of TS1_C2

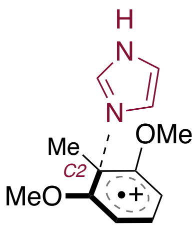

Center Number	Atomic Number	Atomic Type	Coordinates (Angstroms) X	Y	Z
1	6	0	-1.235370	-1.056555	0.106696
2	6	0	-1.228911	-1.686155	-1.126861
3	6	0	0.000872	-1.985628	-1.725746
4	6	0	1.230422	-1.684979	-1.126974
5	6	0	1.236383	-1.055421	0.106605
6	1	0	-2.150921	-1.962058	-1.621943
7	1	0	0.001065	-2.481876	-2.691064
8	1	0	2.152649	-1.959990	-1.622149
9	8	0	2.329717	-0.733035	0.797806
10	8	0	-2.328961	-0.735161	0.797955
11	6	0	3.603416	-1.057020	0.235672
12	1	0	3.740039	-0.541782	-0.719743
13	1	0	4.340079	-0.706567	0.956249
14	1	0	3.696892	-2.138568	0.100647
15	6	0	-3.602393	-1.060381	0.235931
16	1	0	-4.339337	-0.710434	0.956467
17	1	0	-3.739517	-0.545455	-0.719581
18	1	0	-3.694902	-2.142043	0.101133
19	6	0	0.000341	-0.646935	0.746538
20	7	0	-0.000548	1.351385	0.058500
21	6	0	-0.000890	2.461038	0.772375
22	6	0	-0.001078	1.703085	-1.267956
23	1	0	-0.000641	2.529398	1.850223
24	6	0	-0.001741	3.069007	-1.352294
25	1	0	-0.000906	0.963716	-2.056548
26	1	0	-0.002264	3.740526	-2.196660
27	6	0	0.000271	-0.341065	2.212104
28	1	0	-0.895344	0.212552	2.493430
29	1	0	0.001070	-1.289762	2.762316
30	1	0	0.895123	0.213887	2.493232

31	7	0	-0.001600	3.522360	-0.056073
32	1	0	-0.002033	4.493877	0.234382

Cartesian coordinates of INT1 H$^+$_C2

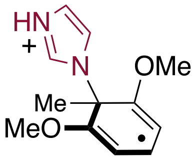

Center Number	Atomic Number	Atomic Type	Coordinates (Angstroms) X	Y	Z
1	6	0	-1.251394	-0.913014	0.083831
2	6	0	-1.230012	-2.051845	-0.673097
3	6	0	0.000259	-2.637072	-1.054326
4	6	0	1.230415	-2.051610	-0.673089
5	6	0	1.251573	-0.912775	0.083839
6	1	0	-2.158602	-2.515613	-0.985863
7	1	0	0.000346	-3.539728	-1.653147
8	1	0	2.159096	-2.515198	-0.985850
9	8	0	2.349951	-0.250704	0.500176
10	8	0	-2.349901	-0.251150	0.500158
11	6	0	3.618284	-0.776970	0.123041
12	1	0	3.720815	-0.790551	-0.967058
13	1	0	4.361302	-0.108608	0.555976
14	1	0	3.749690	-1.787959	0.522444
15	6	0	-3.618132	-0.777638	0.122988
16	1	0	-4.361278	-0.109406	0.555905
17	1	0	-3.720630	-0.791235	-0.967114
18	1	0	-3.749372	-1.788650	0.522387
19	6	0	0.000022	-0.229990	0.586638
20	7	0	-0.000115	1.170197	0.024791
21	6	0	-0.000313	2.319257	0.695628
22	6	0	-0.000057	1.439883	-1.330069
23	1	0	-0.000406	2.437974	1.767394
24	6	0	-0.000225	2.792677	-1.468438
25	1	0	0.000095	0.646571	-2.063436
26	1	0	-0.000246	3.420417	-2.345063
27	6	0	0.000012	-0.154900	2.120492
28	1	0	-0.896168	0.351741	2.485381
29	1	0	0.000082	-1.178028	2.502850
30	1	0	0.896119	0.351864	2.485387
31	7	0	-0.000362	3.309667	-0.194097
32	1	0	-0.000494	4.296174	0.047637

Cartesian coordinates of pair_C4

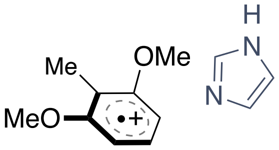

Center Number	Atomic Number	Atomic Type	Coordinates (Angstroms) X	Y	Z
1	6	0	0.114006	1.463205	0.124459
2	6	0	-1.066094	1.132770	-1.967372
3	6	0	-1.997136	0.335938	-1.275331
4	6	0	-1.858028	0.106626	0.129642
5	6	0	-0.827216	0.683265	0.851314
6	1	0	-1.188814	1.290054	-3.032626
7	1	0	-2.822139	-0.108634	-1.819024
8	8	0	-2.701105	-0.670679	0.817290
9	8	0	1.088762	1.978349	0.821464
10	6	0	-3.784549	-1.315189	0.139796
11	1	0	-3.408414	-1.995023	-0.630105
12	1	0	-4.306027	-1.883320	0.908087
13	1	0	-4.463311	-0.576799	-0.296270
14	6	0	2.178003	2.671209	0.182262
15	1	0	2.869069	2.910311	0.987244
16	1	0	2.645082	2.006429	-0.547824
17	1	0	1.813497	3.587329	-0.286928
18	6	0	-0.012688	1.692451	-1.299307
19	1	0	0.721403	2.296403	-1.816867
20	7	0	1.917482	-0.568253	-0.677925
21	6	0	1.130609	-1.616046	-1.098669
22	6	0	2.718492	-1.071827	0.240752
23	6	0	1.465428	-2.760202	-0.420538
24	1	0	0.371662	-1.487130	-1.858903
25	1	0	3.472609	-0.530415	0.794507
26	1	0	1.091513	-3.771345	-0.465938
27	6	0	-0.677694	0.424778	2.318658
28	1	0	-0.320469	-0.598677	2.484784
29	1	0	0.029952	1.116874	2.773332
30	1	0	-1.644533	0.517617	2.819235
31	7	0	2.478844	-2.391463	0.427528
32	1	0	2.964587	-2.998892	1.076062

Cartesian coordinates of TS1_C4

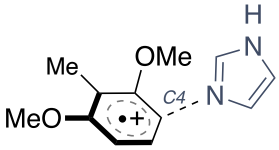

Center Number	Atomic Number	Atomic Type	X	Y	Z
1	6	0	0.245421	1.235967	0.290277
2	6	0	0.351802	-0.725623	1.782648
3	6	0	1.518252	-1.116068	1.186789
4	6	0	2.045813	-0.336936	0.118245
5	6	0	1.425281	0.853696	-0.326350
6	1	0	-0.060705	-1.288251	2.612933
7	1	0	2.047507	-1.991719	1.542081
8	8	0	3.167328	-0.656513	-0.524654
9	8	0	-0.354168	2.350753	-0.154645
10	6	0	3.895092	-1.827969	-0.141767
11	1	0	3.278263	-2.721634	-0.268746
12	1	0	4.749828	-1.869430	-0.814571
13	1	0	4.240251	-1.743794	0.891993
14	6	0	-1.479492	2.877778	0.552302
15	1	0	-1.749239	3.791075	0.024094
16	1	0	-2.320008	2.178584	0.529084
17	1	0	-1.207778	3.117195	1.584631
18	6	0	-0.376220	0.407186	1.297432
19	1	0	-1.120890	0.847847	1.950201
20	7	0	-1.848602	-0.413856	0.160466
21	6	0	-2.980180	-1.011580	0.656151
22	6	0	-1.786968	-0.657596	-1.135114
23	6	0	-3.625479	-1.626738	-0.382223
24	1	0	-3.247845	-0.957905	1.701379
25	1	0	-1.012499	-0.323038	-1.810992
26	1	0	-4.540902	-2.195703	-0.425870
27	6	0	2.058639	1.656465	-1.426886
28	1	0	3.100270	1.885941	-1.184932
29	1	0	2.061916	1.089946	-2.364084
30	1	0	1.518920	2.589480	-1.585196
31	7	0	-2.856542	-1.389688	-1.494826
32	1	0	-3.058136	-1.709212	-2.436121

Cartesian coordinates of INT1 H$^+$_C4

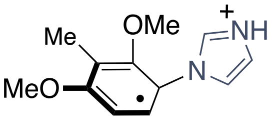

Center Number	Atomic Number	Atomic Type	Coordinates (Angstroms) X	Y	Z
1	6	0	-0.113018	1.030002	-0.149190
2	6	0	-0.270958	-1.113185	-1.432570
3	6	0	-1.545726	-1.266646	-0.963836
4	6	0	-2.115236	-0.311785	-0.092591
5	6	0	-1.390740	0.865825	0.295305
6	1	0	0.160218	-1.833643	-2.119715
7	1	0	-2.125431	-2.124191	-1.287150
8	8	0	-3.361104	-0.399868	0.413659
9	8	0	0.623347	2.091321	0.288852
10	6	0	-4.159100	-1.526604	0.064167
11	1	0	-3.684620	-2.456348	0.393956
12	1	0	-5.105466	-1.392789	0.586612
13	1	0	-4.336461	-1.558409	-1.015483
14	6	0	1.311579	2.852253	-0.706755
15	1	0	1.626859	3.773786	-0.217011
16	1	0	2.198518	2.327353	-1.077769
17	1	0	0.643102	3.090550	-1.540888
18	6	0	0.604899	0.015774	-0.996380
19	1	0	1.091687	0.489114	-1.859526
20	7	0	1.768581	-0.521962	-0.198861
21	6	0	1.659516	-1.172377	1.012474
22	6	0	3.047765	-0.475254	-0.557005
23	6	0	2.919591	-1.521718	1.389675
24	1	0	0.703608	-1.323894	1.491513
25	1	0	3.433342	-0.023451	-1.458898
26	1	0	3.285669	-2.038150	2.262497
27	6	0	-2.067139	1.877185	1.178393
28	1	0	-3.024420	2.182305	0.746390
29	1	0	-2.280373	1.447458	2.162529
30	1	0	-1.436441	2.755957	1.309637
31	7	0	3.759246	-1.075745	0.395217
32	1	0	4.768876	-1.184142	0.378603

Cartesian coordinates of pair_C1

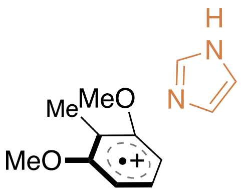

Center Number	Atomic Number	Atomic Type	Coordinates (Angstroms) X	Y	Z
1	6	0	0.008923	-1.698014	-1.287978
2	6	0	2.002382	-0.354976	-1.271571
3	6	0	1.861160	-0.111354	0.131224
4	6	0	0.824431	-0.674117	0.855361
5	1	0	-0.728716	-2.301081	-1.801519
6	1	0	2.831846	0.078974	-1.817040
7	8	0	2.707307	0.667165	0.813294
8	8	0	-1.099186	-1.956923	0.833113
9	6	0	0.669285	-0.399938	2.319325
10	1	0	0.285561	0.616205	2.471612
11	1	0	-0.021498	-1.103029	2.783219
12	1	0	1.637960	-0.461930	2.820542
13	6	0	3.795940	1.300113	0.133151
14	1	0	4.317828	1.872607	0.897923
15	1	0	4.472419	0.554639	-0.294253
16	1	0	3.425391	1.974802	-0.643915
17	6	0	-2.188865	-2.653566	0.198965
18	1	0	-2.883494	-2.879982	1.004579
19	1	0	-2.651393	-1.996655	-0.541039
20	1	0	-1.826091	-3.576922	-0.257212
21	6	0	1.067761	-1.151394	-1.958657
22	1	0	1.191730	-1.318868	-3.022202
23	6	0	-0.119397	-1.454838	0.133443
24	7	0	-1.924347	0.568853	-0.703574
25	6	0	-1.119577	1.611734	-1.102183
26	6	0	-2.729446	1.071152	0.212241
27	6	0	-1.447561	2.751501	-0.413379
28	1	0	-0.353433	1.482477	-1.855148
29	1	0	-3.496435	0.532383	0.750670
30	1	0	-1.060466	3.758304	-0.441680
31	7	0	-2.475020	2.385086	0.418679

32	1	0	-2.960821	2.990751	1.068818

Cartesian coordinates of TS1_C1

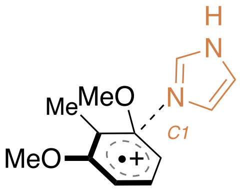

Center Number	Atomic Number	Atomic Type	Coordinates (Angstroms) X	Y	Z
1	6	0	-0.005621	1.420785	1.392376
2	6	0	2.081826	0.184018	1.295938
3	6	0	1.933658	-0.038153	-0.080851
4	6	0	0.831947	0.453693	-0.774240
5	1	0	-0.751483	1.982961	1.940003
6	1	0	2.946698	-0.191013	1.828874
7	8	0	2.833965	-0.714046	-0.832114
8	8	0	-1.024085	1.874537	-0.769023
9	6	0	0.684173	0.216572	-2.240434
10	1	0	0.535264	-0.853187	-2.435110
11	1	0	-0.156040	0.772673	-2.654510
12	1	0	1.600335	0.505918	-2.763823
13	6	0	4.031693	-1.164024	-0.206028
14	1	0	4.610734	-1.647650	-0.991793
15	1	0	4.599726	-0.322078	0.202976
16	1	0	3.813733	-1.888477	0.585124
17	6	0	-2.061228	2.629833	-0.128979
18	1	0	-2.684380	3.009341	-0.937241
19	1	0	-2.651834	1.987155	0.530479
20	1	0	-1.633498	3.464087	0.431995
21	6	0	1.105742	0.930413	2.007029
22	1	0	1.265344	1.119365	3.063567
23	6	0	-0.224859	1.117165	-0.015337
24	7	0	-1.438048	-0.445452	0.201235
25	6	0	-1.360841	-1.479224	1.099225
26	6	0	-2.376301	-0.735133	-0.681923
27	6	0	-2.293444	-2.417251	0.748757
28	1	0	-0.661248	-1.472136	1.923068
29	1	0	-2.668540	-0.120556	-1.521580
30	1	0	-2.564194	-3.363797	1.189652
31	7	0	-2.917736	-1.926034	-0.371940

32	1	0	-3.669639	-2.378298	-0.880843

Cartesian coordinates of INT1 H$^+$_C1

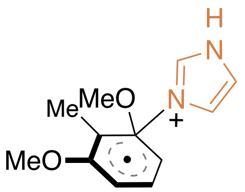

Center Number	Atomic Number	Atomic Type	Coordinates (Angstroms) X	Y	Z
1	6	0	-0.006337	1.441071	1.147781
2	6	0	2.272486	0.592625	1.112682
3	6	0	2.012385	-0.167340	-0.046167
4	6	0	0.778076	-0.148924	-0.664781
5	1	0	-0.798635	2.020083	1.610772
6	1	0	3.246619	0.577675	1.583365
7	8	0	2.949092	-0.967872	-0.624689
8	8	0	-0.810541	1.498097	-1.159506
9	6	0	0.485547	-0.968478	-1.881023
10	1	0	0.107368	-1.962459	-1.602435
11	1	0	-0.268355	-0.484200	-2.507029
12	1	0	1.390969	-1.118674	-2.471260
13	6	0	4.262746	-0.972562	-0.079953
14	1	0	4.843236	-1.640831	-0.715577
15	1	0	4.705121	0.029080	-0.105055
16	1	0	4.264153	-1.355313	0.946174
17	6	0	-1.628391	2.620314	-0.819139
18	1	0	-2.045065	2.974278	-1.762499
19	1	0	-2.446341	2.341680	-0.145187
20	1	0	-1.031705	3.415877	-0.365003
21	6	0	1.236189	1.383242	1.684399
22	1	0	1.455453	1.946118	2.586647
23	6	0	-0.342180	0.693909	-0.108042
24	7	0	-1.497410	-0.253146	0.218970
25	6	0	-1.549299	-1.099873	1.307188
26	6	0	-2.553102	-0.476070	-0.560425
27	6	0	-2.680156	-1.846079	1.175991
28	1	0	-0.783618	-1.097685	2.068662
29	1	0	-2.770725	0.031784	-1.487325
30	1	0	-3.102085	-2.617264	1.800303
31	7	0	-3.281768	-1.434215	0.009713

32	1	0	-4.148333	-1.801434	-0.372840

Cartesian coordinates of pair_C5

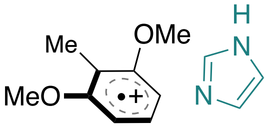

Center Number	Atomic Number	Atomic Type	Coordinates (Angstroms) X	Y	Z
1	6	0	-1.394669	1.251729	-0.184024
2	6	0	-0.597763	1.232302	-1.307544
3	6	0	-0.634180	-1.242200	-1.325630
4	6	0	-1.434743	-1.236728	-0.194013
5	6	0	-1.844986	0.016443	0.421160
6	1	0	-0.240442	2.143102	-1.770543
7	1	0	-0.315215	-2.164998	-1.791953
8	8	0	-1.896781	-2.307982	0.422273
9	8	0	-1.824910	2.337567	0.448783
10	6	0	-2.699255	0.055802	1.624666
11	1	0	-2.926851	-0.939221	1.999037
12	1	0	-2.208790	0.650488	2.404612
13	1	0	-3.631923	0.584933	1.388170
14	6	0	-1.569061	-3.602482	-0.104873
15	1	0	-2.069826	-4.314901	0.546769
16	1	0	-1.942554	-3.692467	-1.128332
17	1	0	-0.486851	-3.753627	-0.074851
18	6	0	-1.440487	3.615268	-0.071505
19	1	0	-1.907987	4.349395	0.581327
20	1	0	-0.352310	3.721609	-0.042147
21	1	0	-1.807812	3.730526	-1.095379
22	6	0	-0.230540	-0.015106	-1.854763
23	1	0	0.398389	-0.017134	-2.738367
24	7	0	2.607574	0.089396	-0.664491
25	6	0	2.146370	-0.027719	0.626389
26	6	0	3.921930	0.049765	-0.573442
27	6	0	3.198300	-0.138932	1.499578
28	1	0	1.088657	-0.024780	0.854587
29	1	0	4.621690	0.114657	-1.394032
30	1	0	3.248865	-0.246646	2.572006
31	7	0	4.321218	-0.087489	0.714130
32	1	0	5.279447	-0.139819	1.037174

Cartesian coordinates of TS1_C5

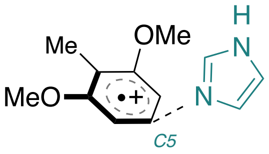

Center Number	Atomic Number	Atomic Type	Coordinates (Angstroms) X	Y	Z
1	6	0	1.177137	-1.302655	-0.202535
2	6	0	0.191460	-1.227097	-1.155853
3	6	0	0.399940	1.247885	-1.153265
4	6	0	1.387423	1.158656	-0.200393
5	6	0	1.793490	-0.116818	0.323528
6	1	0	-0.284611	-2.110466	-1.560821
7	1	0	0.077049	2.198038	-1.558192
8	8	0	2.055890	2.202998	0.308619
9	8	0	1.665949	-2.439649	0.314020
10	6	0	2.847095	-0.237472	1.363187
11	1	0	3.130150	0.733926	1.763164
12	1	0	2.505952	-0.892689	2.170875
13	1	0	3.734190	-0.718471	0.930468
14	6	0	1.710587	3.506642	-0.157379
15	1	0	2.357505	4.194227	0.384850
16	1	0	1.895747	3.590562	-1.232810
17	1	0	0.661455	3.727508	0.062551
18	6	0	1.109848	-3.671301	-0.144511
19	1	0	1.637141	-4.453605	0.399044
20	1	0	0.039563	-3.716972	0.080392
21	1	0	1.274357	-3.789715	-1.220156
22	6	0	-0.248020	0.057777	-1.586913
23	1	0	-0.881386	0.112317	-2.464157
24	7	0	-2.093717	0.198558	-0.385521
25	6	0	-1.970430	0.314715	0.973937
26	6	0	-3.376266	0.062783	-0.658960
27	6	0	-3.217724	0.252424	1.536293
28	1	0	-1.007172	0.437761	1.451926
29	1	0	-3.815960	-0.056641	-1.637944
30	1	0	-3.554469	0.307590	2.559669
31	7	0	-4.088447	0.093478	0.486878
32	1	0	-5.096319	0.012355	0.557716

Cartesian coordinates of INT1 H^+_C5

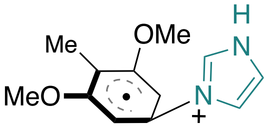

Center Number	Atomic Number	Atomic Type	Coordinates (Angstroms) X	Y	Z
1	6	0	-1.278335	1.216068	-0.154134
2	6	0	-0.054207	1.258158	-0.749648
3	6	0	-0.038493	-1.262693	-0.742721
4	6	0	-1.264805	-1.241101	-0.147316
5	6	0	-1.927328	-0.015704	0.198687
6	1	0	0.426302	2.191304	-1.017071
7	1	0	0.456281	-2.189379	-1.006336
8	8	0	-1.972658	-2.355726	0.160734
9	8	0	-2.012034	2.312858	0.155562
10	6	0	-3.264234	0.007876	0.862263
11	1	0	-3.554874	-0.987570	1.195435
12	1	0	-3.257487	0.689230	1.718924
13	1	0	-4.032015	0.381624	0.172621
14	6	0	-1.389925	-3.615239	-0.143038
15	1	0	-2.107538	-4.364277	0.190081
16	1	0	-1.222913	-3.719393	-1.220760
17	1	0	-0.443742	-3.748492	0.392972
18	6	0	-1.458894	3.585474	-0.150283
19	1	0	-2.193028	4.318568	0.182144
20	1	0	-0.515625	3.740949	0.384953
21	1	0	-1.294897	3.691547	-1.228274
22	6	0	0.697796	0.001630	-1.046008
23	1	0	1.035849	0.000451	-2.090624
24	7	0	1.989505	0.011686	-0.270797
25	6	0	2.086053	0.017354	1.105125
26	6	0	3.210297	0.017288	-0.794939
27	6	0	3.412408	0.026800	1.409559
28	1	0	1.209537	0.014317	1.735625
29	1	0	3.443819	0.014982	-1.849160
30	1	0	3.926889	0.033438	2.356862
31	7	0	4.085437	0.026480	0.209682
32	1	0	5.094394	0.033015	0.094754

◆ TS calculation (whole pathway)

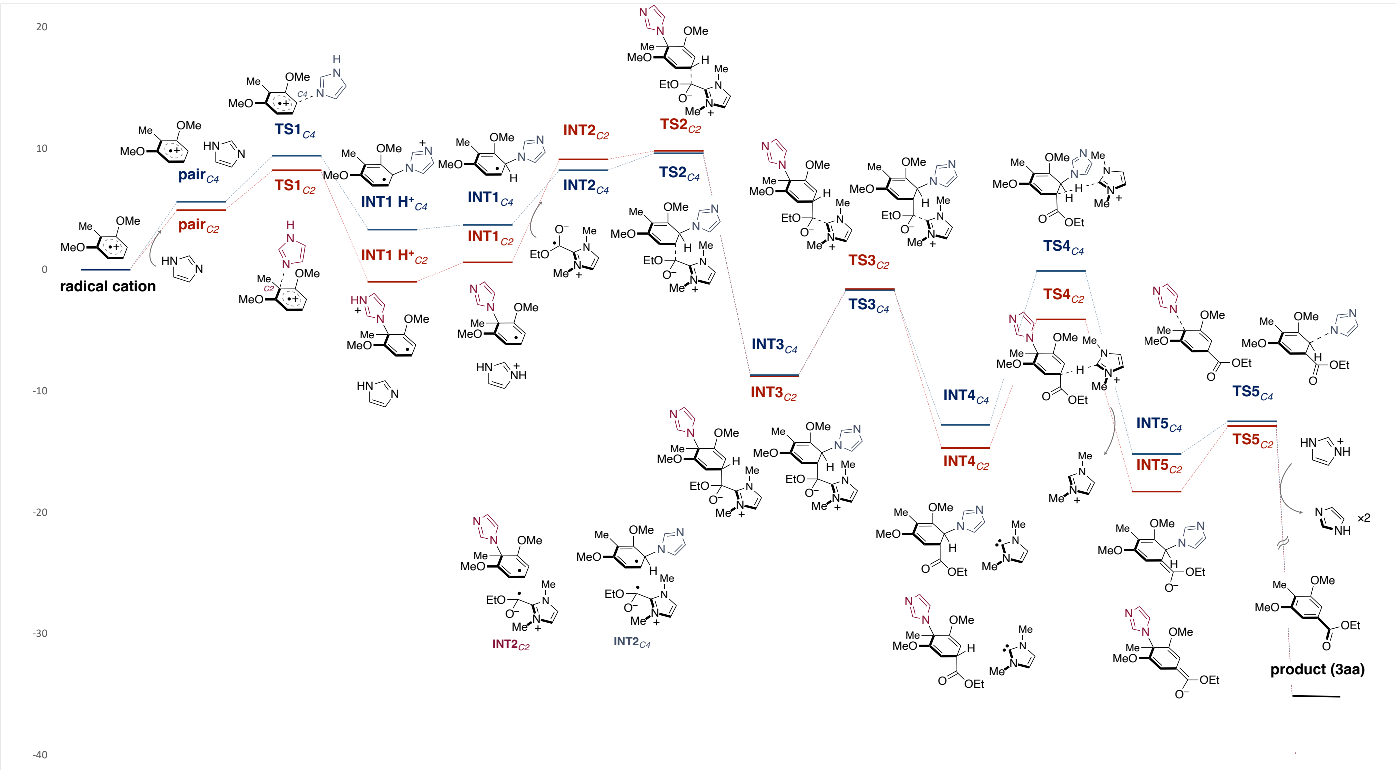

(U)M06-2X/6-31+G(d,p)/SMD (dichloromethane)			
	Free Energies (hartree)	Thermal Enthalpies (hartree)	Energies (hartree)
INT1_C2	-725.721680	-725.662720	-725.936180
INT1_C4	-725.716780	-725.655700	-725.929110
NHC–alkoxycarbonyl radical	-572.220655	-572.164405	-572.388747
INT2_C2	-1297.928882	-1297.840120	-1298.340901
TS2_C2	-1297.927815	-1297.841047	-1298.340825
INT3_C2	-1297.957399	-1297.866551	-1298.368803
INT2_C4	-1297.930309	-1297.840416	-1298.340819
TS2_C4	-1297.928120	-1297.839528	-1298.339027
INT3_C4	-1297.957187	-1297.866305	-1298.368815

TS3_C2	-1297.945981	-1297.855708	-1298.356605
INT4_C2	-1297.966782	-1297.872036	-1298.374451
TS3_C4	-1297.946148	-1297.852895	-1298.353896
INT4_C4	-1297.963721	-1297.868704	-1298.371369
NHC–H^+	-305.078450	-305.040402	-305.190720
TS4_C2	-1297.949963	-1297.859474	-1298.356045
INT5_C2	-992.894107	-992.821657	-993.171666
TS4_C4	-1297.943462	-1297.849645	-1298.346320
INT5_C4	-992.889089	-992.815685	-993.165685
product (3aa)	-767.317350	-767.254025	-767.538847
imidazolide anion	-225.631431	-225.600919	-225.664196
TS5_C2	-992.885424	-992.812818	-993.161082
TS5_C4	-992.884870	-992.810124	-993.158114

Supplementary Fig. 9. Total electronic energies, enthalpies and free energies.

Cartesian coordinates of INT1_C2

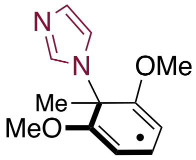

Center Number	Atomic Number	Atomic Type	Coordinates (Angstroms) X	Y	Z
1	6	0	-1.249760	-0.884102	0.092045
2	6	0	-1.229769	-2.018447	-0.672491
3	6	0	0.000561	-2.599153	-1.063346
4	6	0	1.230645	-2.017928	-0.672489
5	6	0	1.250157	-0.883580	0.092053
6	1	0	-2.158172	-2.482027	-0.986461
7	1	0	0.000749	-3.495692	-1.671783
8	1	0	2.159248	-2.481107	-0.986463
9	8	0	2.355320	-0.238641	0.520025
10	8	0	-2.355193	-0.239615	0.520003
11	6	0	3.617873	-0.756888	0.123418
12	1	0	3.711547	-0.754133	-0.967919
13	1	0	4.365349	-0.094437	0.558579
14	1	0	3.757491	-1.774070	0.505259
15	6	0	-3.617532	-0.758361	0.123365
16	1	0	-4.365280	-0.096203	0.558505
17	1	0	-3.711179	-0.755643	-0.967975
18	1	0	-3.756758	-1.775597	0.505206
19	6	0	0.000051	-0.177977	0.577911
20	7	0	-0.000226	1.197939	0.010619
21	6	0	-0.000629	2.392326	0.657810
22	6	0	-0.000160	1.471552	-1.337161
23	1	0	-0.000746	2.469912	1.734660
24	6	0	-0.000593	2.838381	-1.438416
25	1	0	0.000180	0.683077	-2.076578
26	1	0	-0.000691	3.440587	-2.336137
27	7	0	-0.000939	3.405968	-0.185925
28	6	0	0.000037	-0.102791	2.114239
29	1	0	-0.893885	0.409317	2.475711
30	1	0	0.000369	-1.123376	2.505002
31	1	0	0.893635	0.409901	2.475689

Cartesian coordinates of INT1_C4

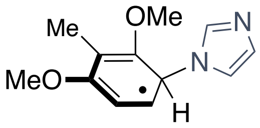

Center Number	Atomic Number	Atomic Type	Coordinates (Angstroms) X	Y	Z
1	6	0	-0.093724	1.026211	-0.178282
2	6	0	-0.241288	-1.173001	-1.357035
3	6	0	-1.525912	-1.300502	-0.907233
4	6	0	-2.105863	-0.300958	-0.095804
5	6	0	-1.383375	0.889462	0.246563
6	1	0	0.204125	-1.935654	-1.987964
7	1	0	-2.101587	-2.173691	-1.193999
8	8	0	-3.366102	-0.359725	0.393218
9	8	0	0.619257	2.115464	0.225783
10	6	0	-4.153327	-1.504745	0.091751
11	1	0	-3.683066	-2.415468	0.477307
12	1	0	-5.109585	-1.348623	0.590295
13	1	0	-4.313929	-1.597203	-0.987571
14	6	0	1.506246	2.708237	-0.721333
15	1	0	1.830890	3.649866	-0.277926
16	1	0	2.383952	2.079880	-0.901619
17	1	0	0.988330	2.910110	-1.665582
18	6	0	0.644800	-0.032413	-0.957284
19	1	0	1.119143	0.405476	-1.846980
20	7	0	1.792970	-0.526454	-0.152148
21	6	0	1.726675	-1.044517	1.118035
22	6	0	3.082914	-0.630496	-0.560715
23	6	0	3.006567	-1.433945	1.422390
24	1	0	0.799397	-1.082183	1.671531
25	1	0	3.398456	-0.289373	-1.538034
26	1	0	3.366724	-1.882345	2.337641
27	7	0	3.848517	-1.171516	0.366023
28	6	0	-2.070496	1.945197	1.069499
29	1	0	-3.037227	2.206450	0.629749
30	1	0	-2.266167	1.579933	2.083362
31	1	0	-1.454397	2.841524	1.137341

Cartesian coordinates of NHC–alkoxycarbonyl radical

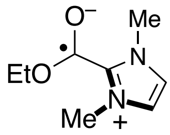

Center Number	Atomic Number	Atomic Type	Coordinates (Angstroms) X	Y	Z
1	6	0	-0.512204	-0.777121	-0.029642
2	8	0	-1.621968	0.021119	0.117596
3	6	0	-2.881466	-0.632069	-0.047730
4	1	0	-2.921720	-1.099743	-1.037942
5	1	0	-2.986088	-1.422736	0.702439
6	6	0	-3.955947	0.422323	0.108872
7	1	0	-4.943148	-0.033009	-0.011908
8	1	0	-3.906359	0.881352	1.100774
9	1	0	-3.842453	1.207258	-0.644952
10	8	0	-0.603009	-2.014937	-0.157423
11	6	0	0.696201	-0.044227	-0.015117
12	7	0	1.963563	-0.608162	-0.015673
13	6	0	2.246664	1.588320	0.046825
14	6	0	2.903002	0.403573	0.030677
15	1	0	2.625002	2.598821	0.066208
16	1	0	3.960120	0.187065	0.050146
17	7	0	0.886903	1.331644	0.029652
18	6	0	2.275114	-2.023772	0.072294
19	1	0	1.867191	-2.451439	0.991708
20	1	0	1.860038	-2.563712	-0.779194
21	1	0	3.361421	-2.121222	0.077142
22	6	0	-0.123884	2.363702	-0.129114
23	1	0	-0.840739	2.338713	0.692317
24	1	0	0.389799	3.325580	-0.135863
25	1	0	-0.661385	2.234855	-1.072508

Cartesian coordinates of INT2_C2

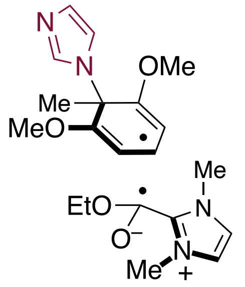

Center Number	Atomic Number	Atomic Type	Coordinates (Angstroms) X	Y	Z
1	6	0	1.249471	1.198094	-0.099511
2	6	0	0.249828	1.218346	-1.025301
3	6	0	0.051765	-1.202724	-0.898051
4	6	0	1.041588	-1.247342	0.035599
5	1	0	-0.081768	2.171910	-1.430444
6	1	0	-0.430394	-2.127257	-1.207895
7	8	0	1.477682	-2.398826	0.665812
8	8	0	1.900000	2.316218	0.386806
9	6	0	1.108401	-3.625937	0.075517
10	1	0	1.427174	-3.665974	-0.974607
11	1	0	1.614314	-4.409021	0.642924
12	1	0	0.024068	-3.796034	0.121560
13	6	0	1.717204	3.522232	-0.322076
14	1	0	2.381272	4.256212	0.137791
15	1	0	1.982380	3.398820	-1.380731
16	1	0	0.683614	3.886989	-0.255307
17	6	0	1.849315	-0.057050	0.475078
18	7	0	3.260105	-0.211660	-0.106607
19	6	0	4.459428	-0.291112	0.520128
20	6	0	3.512604	-0.300946	-1.452192
21	1	0	4.558343	-0.246943	1.594282
22	6	0	4.873805	-0.431848	-1.575562
23	1	0	2.715172	-0.263089	-2.180532
24	1	0	5.457151	-0.529089	-2.481012
25	7	0	5.461920	-0.424866	-0.333250
26	6	0	2.007976	0.014444	2.001287
27	1	0	2.616526	0.874636	2.289834
28	1	0	1.012166	0.126265	2.433008
29	1	0	2.463179	-0.899197	2.390382
30	6	0	-0.431940	0.030931	-1.418917

31	1	0	-1.097526	0.037594	-2.275264
32	6	0	-2.023139	0.540811	0.753023
33	8	0	-2.485326	1.745667	0.381175
34	6	0	-1.872341	2.875415	1.035444
35	1	0	-2.263289	2.933041	2.057177
36	1	0	-0.793473	2.709520	1.085349
37	6	0	-2.227137	4.104077	0.230513
38	1	0	-1.788383	4.988782	0.700830
39	1	0	-1.838371	4.024146	-0.789595
40	1	0	-3.311438	4.240818	0.181776
41	8	0	-1.213255	0.354729	1.642815
42	6	0	-2.751499	-0.570563	0.117854
43	7	0	-2.678906	-1.846835	0.549813
44	6	0	-4.068388	-1.810002	-1.162078
45	6	0	-3.479549	-2.629914	-0.251133
46	1	0	-4.762880	-2.019232	-1.960752
47	1	0	-3.567488	-3.693459	-0.092623
48	7	0	-3.629710	-0.530768	-0.907427
49	6	0	-1.938757	-2.355788	1.709282
50	1	0	-0.879363	-2.123212	1.609992
51	1	0	-2.330127	-1.902844	2.621034
52	1	0	-2.096063	-3.433543	1.737200
53	6	0	-3.971603	0.632782	-1.720787
54	1	0	-4.548399	0.276226	-2.573048
55	1	0	-4.560907	1.340863	-1.138955
56	1	0	-3.054366	1.116222	-2.061640

Cartesian coordinates of TS2_C2

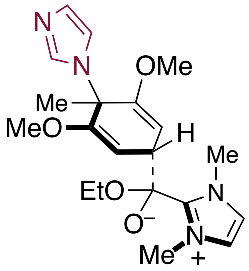

Center Number	Atomic Number	Atomic Type	Coordinates (Angstroms) X	Y	Z
1	6	0	1.281961	1.199203	-0.102528
2	6	0	0.243039	1.225800	-0.977032
3	6	0	0.030008	-1.201569	-0.836725
4	6	0	1.059062	-1.254892	0.046601
5	1	0	-0.094459	2.178554	-1.378041
6	1	0	-0.465340	-2.122230	-1.136284
7	8	0	1.537758	-2.405073	0.635501
8	8	0	1.979543	2.302554	0.336917
9	6	0	1.127220	-3.631373	0.068563
10	1	0	1.376376	-3.669759	-1.000021
11	1	0	1.668616	-4.415382	0.600306
12	1	0	0.048506	-3.797125	0.187747
13	6	0	1.751625	3.518408	-0.343697
14	1	0	2.446928	4.244842	0.079855
15	1	0	1.945345	3.407207	-1.418755
16	1	0	0.725069	3.880365	-0.201676
17	6	0	1.876451	-0.061797	0.473927
18	7	0	3.268655	-0.227218	-0.114141
19	6	0	4.469366	-0.309355	0.510357
20	6	0	3.516053	-0.327526	-1.460405
21	1	0	4.571001	-0.258273	1.583864
22	6	0	4.875706	-0.467306	-1.585820
23	1	0	2.716984	-0.289986	-2.186750
24	1	0	5.455756	-0.574430	-2.492170
25	7	0	5.467243	-0.454978	-0.345174
26	6	0	2.028959	0.017805	1.999991
27	1	0	2.643306	0.874753	2.285774
28	1	0	1.030835	0.140219	2.423523
29	1	0	2.474802	-0.897479	2.396093
30	6	0	-0.492603	0.043917	-1.306291

31	1	0	-1.151129	0.049695	-2.169326
32	6	0	-1.980119	0.527204	0.697603
33	8	0	-2.443344	1.752605	0.351914
34	6	0	-1.849695	2.858042	1.054650
35	1	0	-2.262551	2.886227	2.069639
36	1	0	-0.771406	2.697720	1.127166
37	6	0	-2.187557	4.113619	0.283271
38	1	0	-1.762492	4.983869	0.791853
39	1	0	-1.775426	4.067959	-0.729930
40	1	0	-3.270819	4.250125	0.212599
41	8	0	-1.197287	0.329824	1.618157
42	6	0	-2.773092	-0.558307	0.100805
43	7	0	-2.735713	-1.837022	0.533447
44	6	0	-4.152382	-1.762291	-1.155157
45	6	0	-3.577046	-2.597195	-0.251937
46	1	0	-4.866640	-1.949290	-1.941821
47	1	0	-3.695848	-3.656914	-0.087856
48	7	0	-3.664352	-0.495859	-0.913842
49	6	0	-2.010013	-2.365982	1.692093
50	1	0	-0.953066	-2.116391	1.617966
51	1	0	-2.422338	-1.943270	2.609538
52	1	0	-2.152864	-3.446163	1.689027
53	6	0	-3.999639	0.677463	-1.715409
54	1	0	-4.588962	0.333721	-2.564567
55	1	0	-4.575361	1.388855	-1.123773
56	1	0	-3.082838	1.155654	-2.063075

Cartesian coordinates of INT3_C2

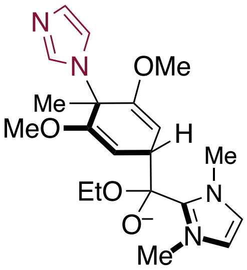

Center Number	Atomic Number	Atomic Type	Coordinates (Angstroms) X	Y	Z
1	6	0	-1.678755	1.348809	-0.238436
2	6	0	-0.457557	1.539397	0.269928
3	6	0	-0.117740	-0.935254	0.379154
4	6	0	-1.335395	-1.129336	-0.138178
5	1	0	-0.084185	2.542769	0.441724
6	1	0	0.483372	-1.793366	0.669264
7	8	0	-1.928661	-2.331877	-0.356975
8	8	0	-2.575055	2.321831	-0.556053
9	6	0	-1.194241	-3.491969	-0.006478
10	1	0	-0.972822	-3.504751	1.067020
11	1	0	-1.828540	-4.341405	-0.259475
12	1	0	-0.257401	-3.549430	-0.574296
13	6	0	-2.180379	3.663559	-0.325756
14	1	0	-3.020183	4.284742	-0.637412
15	1	0	-1.972652	3.832469	0.737371
16	1	0	-1.293198	3.917626	-0.916978
17	6	0	-2.266508	-0.010554	-0.567342
18	7	0	-3.527536	-0.158769	0.196085
19	6	0	-4.780709	-0.392642	-0.269781
20	6	0	-3.615436	-0.090304	1.566065
21	1	0	-4.999422	-0.491576	-1.322372
22	6	0	-4.938690	-0.288743	1.864025
23	1	0	-2.747104	0.092938	2.182560
24	1	0	-5.407589	-0.304362	2.837969
25	7	0	-5.660885	-0.477108	0.709397
26	6	0	-2.543234	-0.110368	-2.072559
27	1	0	-3.225746	0.678943	-2.394092
28	1	0	-1.593086	0.013277	-2.597971
29	1	0	-2.961034	-1.086563	-2.327687
30	6	0	0.490800	0.421098	0.564195

31	1	0	0.833747	0.513605	1.606213
32	6	0	1.781784	0.588381	-0.345606
33	8	0	2.416703	1.771832	0.241087
34	6	0	3.527597	2.245216	-0.496536
35	1	0	4.317176	1.473644	-0.528407
36	1	0	3.227629	2.450646	-1.529769
37	6	0	4.049619	3.497315	0.181085
38	1	0	4.910100	3.894367	-0.366446
39	1	0	3.273124	4.268048	0.208561
40	1	0	4.363060	3.287021	1.208803
41	8	0	1.582446	0.643216	-1.621882
42	6	0	2.735238	-0.610882	0.023897
43	7	0	2.946924	-1.675657	-0.769933
44	6	0	4.055433	-2.055818	1.091855
45	6	0	3.765176	-2.578706	-0.126304
46	1	0	4.663079	-2.426274	1.903237
47	1	0	4.068386	-3.501537	-0.596057
48	7	0	3.407856	-0.841571	1.167934
49	6	0	2.409442	-1.925058	-2.113022
50	1	0	1.347665	-1.690746	-2.130614
51	1	0	2.920665	-1.296783	-2.840285
52	1	0	2.583614	-2.979656	-2.328247
53	6	0	3.440343	-0.015312	2.377317
54	1	0	2.760651	-0.438580	3.120157
55	1	0	4.459521	-0.024336	2.765599
56	1	0	3.142481	0.998029	2.122687

Cartesian coordinates of INT2_C4

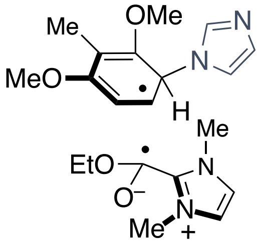

Center Number	Atomic Number	Atomic Type	Coordinates (Angstroms) X	Y	Z
1	6	0	1.257068	-0.809119	0.659719
2	6	0	-0.175297	-0.438042	-1.695025
3	6	0	-0.123560	-1.695216	-1.118909
4	6	0	0.569398	-1.863539	0.120391
5	1	0	-0.756409	-0.276732	-2.599554
6	8	0	-0.802238	-2.821706	-1.577073
7	8	0	1.899847	-0.937265	1.888470
8	6	0	-1.476272	-2.676119	-2.801416
9	1	0	-0.782973	-2.408964	-3.611873
10	1	0	-1.937821	-3.640272	-3.024325
11	1	0	-2.260462	-1.906390	-2.748576
12	6	0	3.191008	-1.536919	1.829009
13	1	0	3.524720	-1.673992	2.860733
14	1	0	3.147822	-2.512799	1.329647
15	1	0	3.907324	-0.896958	1.300077
16	6	0	1.397483	0.528873	0.018646
17	1	0	1.313796	1.318165	0.785921
18	7	0	2.855279	0.733265	-0.435575
19	6	0	3.506278	0.002140	-1.392039
20	6	0	3.755703	1.596604	0.083122
21	6	0	4.800215	0.470639	-1.407184
22	1	0	3.002177	-0.768598	-1.958318
23	1	0	3.493127	2.288383	0.872819
24	1	0	5.622260	0.147447	-2.031567
25	7	0	4.950289	1.472615	-0.477860
26	6	0	0.469761	-3.185857	0.836796
27	1	0	-0.576086	-3.507451	0.896943
28	1	0	1.007400	-3.973361	0.296393
29	1	0	0.878238	-3.118085	1.847300
30	6	0	0.419296	0.677252	-1.086042

31	1	0	0.355712	1.647593	-1.574813
32	6	0	-1.455537	1.527912	0.989755
33	8	0	-1.896733	2.522637	0.216298
34	8	0	-0.703544	1.666071	1.934732
35	6	0	-2.142526	0.253472	0.682737
36	6	0	-1.179030	3.769491	0.328235
37	7	0	-2.111201	-0.833828	1.475334
38	7	0	-2.943144	-0.026190	-0.364769
39	1	0	-1.331593	4.173854	1.332724
40	1	0	-0.112927	3.561749	0.191359
41	6	0	-1.716918	4.688123	-0.743271
42	6	0	-2.910717	-1.804972	0.930934
43	6	0	-1.390667	-0.982590	2.741808
44	6	0	-3.431358	-1.299990	-0.221243
45	6	0	-3.261257	0.839867	-1.500620
46	1	0	-1.199550	5.649742	-0.689790
47	1	0	-1.552864	4.261632	-1.737072
48	1	0	-2.787596	4.862917	-0.604557
49	1	0	-3.032697	-2.766713	1.404220
50	1	0	-1.753968	-0.246751	3.459274
51	1	0	-1.591576	-1.989537	3.106173
52	1	0	-0.323002	-0.841796	2.572586
53	1	0	-4.091038	-1.738496	-0.953757
54	1	0	-3.757051	0.221426	-2.248025
55	1	0	-3.921983	1.646363	-1.182204
56	1	0	-2.338627	1.256635	-1.903480

Cartesian coordinates of TS2_C4

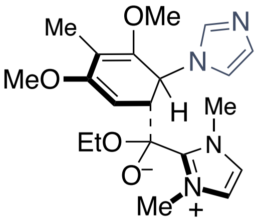

Center Number	Atomic Number	Atomic Type	Coordinates (Angstroms) X	Y	Z
1	6	0	-1.202032	-0.991037	-0.814632
2	6	0	0.168282	-0.507062	1.574766
3	6	0	0.144819	-1.773265	1.039679
4	6	0	-0.494799	-2.001058	-0.231628
5	1	0	0.715614	-0.311166	2.492644
6	8	0	0.793940	-2.880274	1.562493
7	8	0	-1.655622	-1.155422	-2.110917
8	6	0	1.416414	-2.700172	2.812177
9	1	0	0.690224	-2.396125	3.578781
10	1	0	1.851750	-3.662708	3.086868
11	1	0	2.213458	-1.944831	2.765049
12	6	0	-3.026930	-0.864985	-2.353053
13	1	0	-3.235227	-1.197227	-3.372252
14	1	0	-3.673872	-1.406784	-1.651858
15	1	0	-3.231134	0.208325	-2.281754
16	6	0	-1.430551	0.342298	-0.163306
17	1	0	-1.419949	1.125650	-0.939054
18	7	0	-2.845777	0.441186	0.389576
19	6	0	-3.453167	-0.461996	1.221338
20	6	0	-3.688863	1.491190	0.263557
21	6	0	-4.667182	0.092822	1.554476
22	1	0	-2.977135	-1.393648	1.492480
23	1	0	-3.441445	2.350715	-0.346095
24	1	0	-5.439562	-0.319876	2.189072
25	7	0	-4.808193	1.319093	0.949198
26	6	0	-0.281654	-3.324770	-0.916203
27	1	0	0.783923	-3.483006	-1.126516
28	1	0	-0.597557	-4.150906	-0.271440
29	1	0	-0.834752	-3.373508	-1.854860
30	6	0	-0.398994	0.586098	0.877284
31	1	0	-0.422672	1.561022	1.361564

32	6	0	1.289060	1.562595	-0.904794
33	8	0	1.640891	2.628461	-0.158565
34	8	0	0.597728	1.637892	-1.911076
35	6	0	2.118832	0.384057	-0.577641
36	6	0	0.815038	3.794209	-0.320834
37	7	0	2.227503	-0.711967	-1.352109
38	7	0	2.916388	0.204407	0.495334
39	1	0	0.941208	4.182260	-1.335722
40	1	0	-0.231908	3.498589	-0.190376
41	6	0	1.247214	4.795934	0.725075
42	6	0	3.112615	-1.587595	-0.768371
43	6	0	1.611357	-0.939127	-2.661055
44	6	0	3.542040	-1.015679	0.386829
45	6	0	3.085489	1.102783	1.635955
46	1	0	0.648024	5.705639	0.630763
47	1	0	1.105668	4.390863	1.731389
48	1	0	2.300636	5.060983	0.596376
49	1	0	3.350091	-2.535749	-1.225008
50	1	0	2.007941	-0.223546	-3.382448
51	1	0	1.874055	-1.953048	-2.961972
52	1	0	0.530971	-0.830466	-2.588144
53	1	0	4.223757	-1.369664	1.144306
54	1	0	3.595553	0.541135	2.418110
55	1	0	3.679277	1.970695	1.348757
56	1	0	2.106202	1.430652	1.984156

Cartesian coordinates of INT3_C4

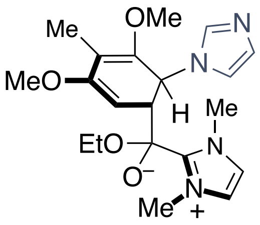

Center Number	Atomic Number	Atomic Type	Coordinates (Angstroms) X	Y	Z
1	6	0	-2.066533	0.745776	0.901138
2	6	0	0.109874	1.239495	-0.880128
3	6	0	-0.607815	2.237256	-0.334620
4	6	0	-1.683349	2.009939	0.651582
5	1	0	0.870035	1.435129	-1.629781
6	8	0	-0.461457	3.559332	-0.625799
7	8	0	-3.153407	0.523189	1.696577
8	6	0	0.572048	3.910857	-1.528030
9	1	0	0.397229	3.472985	-2.517641
10	1	0	0.555408	4.998349	-1.602165
11	1	0	1.548797	3.581204	-1.151778
12	6	0	-3.151804	-0.639946	2.522150
13	1	0	-3.992324	-0.518419	3.206424
14	1	0	-3.302152	-1.551285	1.934559
15	1	0	-2.222398	-0.713351	3.096464
16	6	0	-1.446776	-0.455009	0.226401
17	1	0	-1.285399	-1.245169	0.964635
18	7	0	-2.417296	-1.000034	-0.736070
19	6	0	-3.071571	-0.308754	-1.726302
20	6	0	-2.748992	-2.308558	-0.877038
21	6	0	-3.785947	-1.251509	-2.423065
22	1	0	-2.974710	0.762038	-1.833437
23	1	0	-2.353192	-3.066916	-0.214088
24	1	0	-4.437527	-1.099808	-3.272198
25	7	0	-3.578669	-2.500005	-1.884100
26	6	0	-2.327303	3.200609	1.304941
27	1	0	-1.568986	3.859134	1.739312
28	1	0	-2.889804	3.795650	0.577260
29	1	0	-3.010850	2.878791	2.091037
30	6	0	-0.081175	-0.180851	-0.430296
31	1	0	-0.004912	-0.844396	-1.300314

32	6	0	1.027979	-0.623963	0.598762
33	8	0	1.012619	-2.086427	0.455319
34	8	0	0.876587	-0.197250	1.813971
35	6	0	2.404617	-0.163293	0.002507
36	6	0	1.789638	-2.764016	1.424959
37	7	0	3.142168	0.847192	0.492983
38	7	0	3.032650	-0.603614	-1.103601
39	1	0	2.851237	-2.476914	1.326229
40	1	0	1.463590	-2.476096	2.430220
41	6	0	1.629564	-4.255366	1.202159
42	6	0	4.247365	1.042892	-0.306671
43	6	0	2.857346	1.686681	1.662810
44	6	0	4.179105	0.132472	-1.311253
45	6	0	2.575665	-1.631246	-2.042216
46	1	0	2.214316	-4.814723	1.939070
47	1	0	0.579053	-4.545731	1.303754
48	1	0	1.970453	-4.542371	0.202207
49	1	0	4.980010	1.802749	-0.083555
50	1	0	1.832760	2.049086	1.608065
51	1	0	2.978745	1.107522	2.575602
52	1	0	3.566272	2.514460	1.634436
53	1	0	4.838150	-0.066207	-2.142398
54	1	0	2.036078	-1.155912	-2.864665
55	1	0	3.453978	-2.147067	-2.430856
56	1	0	1.927815	-2.329058	-1.518170

Cartesian coordinates of TS3_C2

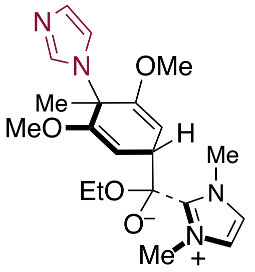

Center Number	Atomic Number	Atomic Type	Coordinates (Angstroms) X	Y	Z
1	6	0	-1.886709	1.326521	-0.223192
2	6	0	-0.640836	1.535745	0.213419
3	6	0	-0.199173	-0.930887	0.135342
4	6	0	-1.446692	-1.138144	-0.296821
5	1	0	-0.297022	2.540753	0.432449
6	1	0	0.472280	-1.768916	0.294132
7	8	0	-2.011760	-2.345810	-0.553588
8	8	0	-2.834017	2.279085	-0.417070
9	6	0	-1.213458	-3.496394	-0.333668
10	1	0	-0.900589	-3.559270	0.714963
11	1	0	-1.840299	-4.352823	-0.581547
12	1	0	-0.328848	-3.486984	-0.980972
13	6	0	-2.479070	3.619195	-0.116572
14	1	0	-3.362994	4.220863	-0.327524
15	1	0	-2.206361	3.721953	0.940067
16	1	0	-1.646000	3.953306	-0.745287
17	6	0	-2.449158	-0.035613	-0.583951
18	7	0	-3.638893	-0.273349	0.263983
19	6	0	-4.924038	-0.491604	-0.115264
20	6	0	-3.614392	-0.310027	1.638072
21	1	0	-5.227207	-0.515420	-1.151144
22	6	0	-4.906517	-0.551952	2.026137
23	1	0	-2.699854	-0.162531	2.194405
24	1	0	-5.293217	-0.650458	3.030809
25	7	0	-5.719275	-0.664194	0.922853
26	6	0	-2.837543	-0.060462	-2.067680
27	1	0	-3.562219	0.724870	-2.291412
28	1	0	-1.934184	0.119011	-2.656112
29	1	0	-3.248747	-1.033102	-2.345237
30	6	0	0.362077	0.431044	0.398326

31	1	0	0.746396	0.472505	1.427110
32	6	0	1.571701	0.717120	-0.520584
33	8	0	2.226748	1.833235	-0.021295
34	6	0	3.313603	2.298055	-0.815314
35	1	0	4.042671	1.482342	-0.931544
36	1	0	2.952103	2.568720	-1.812283
37	6	0	3.931655	3.486272	-0.109136
38	1	0	4.770412	3.872605	-0.695641
39	1	0	3.197169	4.288355	0.010682
40	1	0	4.303922	3.204986	0.880615
41	8	0	1.527265	0.516774	-1.741557
42	6	0	2.915440	-0.696600	0.232028
43	7	0	3.372248	-1.759603	-0.468652
44	6	0	4.746262	-1.579091	1.231072
45	6	0	4.491581	-2.317411	0.121418
46	1	0	5.519541	-1.660756	1.980441
47	1	0	4.997706	-3.174617	-0.297088
48	7	0	3.773570	-0.596548	1.275209
49	6	0	2.761224	-2.287734	-1.683950
50	1	0	1.910500	-1.658492	-1.939437
51	1	0	3.488286	-2.265226	-2.499198
52	1	0	2.442335	-3.318490	-1.511991
53	6	0	3.669033	0.376592	2.355953
54	1	0	3.331747	-0.117720	3.270344
55	1	0	4.646351	0.831786	2.530215
56	1	0	2.956744	1.144575	2.060701

Cartesian coordinates of INT4_C2

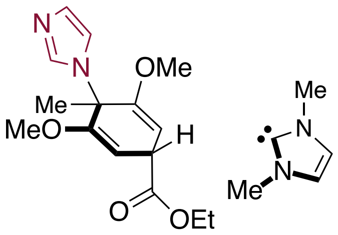

Center Number	Atomic Number	Atomic Type	Coordinates (Angstroms) X	Y	Z
1	6	0	-2.126403	1.279103	-0.317141
2	6	0	-0.863984	1.461353	0.084189
3	6	0	-0.527920	-1.034664	0.125634
4	6	0	-1.794285	-1.207187	-0.263851
5	1	0	-0.474500	2.458910	0.254630
6	1	0	0.113262	-1.887667	0.319045
7	8	0	-2.415748	-2.398380	-0.438837
8	8	0	-3.035093	2.258153	-0.538441
9	6	0	-1.661867	-3.571025	-0.170702
10	1	0	-1.328432	-3.588214	0.872882
11	1	0	-2.332184	-4.409790	-0.356682
12	1	0	-0.793240	-3.636375	-0.835616
13	6	0	-2.623785	3.596233	-0.301959
14	1	0	-3.488862	4.221114	-0.522094
15	1	0	-2.326349	3.731334	0.744050
16	1	0	-1.792153	3.870550	-0.960639
17	6	0	-2.752169	-0.076523	-0.593097
18	7	0	-3.933415	-0.212224	0.285112
19	6	0	-5.237059	-0.365481	-0.063018
20	6	0	-3.884434	-0.189052	1.659308
21	1	0	-5.560660	-0.415043	-1.091763
22	6	0	-5.181224	-0.332862	2.078558
23	1	0	-2.952013	-0.076925	2.193879
24	1	0	-5.553813	-0.364219	3.092793
25	7	0	-6.020407	-0.442672	0.994795
26	6	0	-3.167201	-0.164956	-2.067190
27	1	0	-3.854009	0.643331	-2.325667
28	1	0	-2.266563	-0.069771	-2.679145
29	1	0	-3.634385	-1.128525	-2.279754
30	6	0	0.090022	0.316179	0.305349
31	1	0	0.508429	0.396192	1.318192

32	6	0	1.272478	0.508146	-0.642735
33	8	0	2.058801	1.505308	-0.226141
34	6	0	3.215029	1.781266	-1.039475
35	1	0	3.791515	0.856805	-1.142885
36	1	0	2.877935	2.092433	-2.033183
37	6	0	4.008252	2.866807	-0.350947
38	1	0	4.906030	3.087731	-0.935409
39	1	0	3.419629	3.784315	-0.260682
40	1	0	4.316987	2.547934	0.648902
41	8	0	1.460053	-0.110144	-1.667329
42	6	0	3.498370	-1.126435	0.863411
43	7	0	4.189480	-1.608801	-0.204560
44	6	0	5.585072	-0.201473	0.727301
45	6	0	5.460062	-1.069180	-0.309824
46	1	0	6.403115	0.436935	1.027132
47	1	0	6.144112	-1.337609	-1.101218
48	7	0	4.385128	-0.250775	1.413594
49	6	0	3.651197	-2.560800	-1.163299
50	1	0	2.649896	-2.843404	-0.840479
51	1	0	3.592163	-2.103817	-2.154012
52	1	0	4.286682	-3.448875	-1.206872
53	6	0	4.100785	0.521122	2.614751
54	1	0	4.111812	-0.124924	3.495787
55	1	0	4.860599	1.295666	2.730490
56	1	0	3.119785	0.990294	2.520151

Cartesian coordinates of TS3_C4

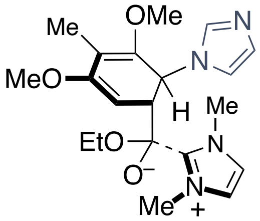

Center Number	Atomic Number	Atomic Type	Coordinates (Angstroms) X	Y	Z
1	6	0	2.340028	0.354323	-0.931111
2	6	0	0.214428	1.330265	0.717055
3	6	0	1.134295	2.145498	0.172937
4	6	0	2.168638	1.678401	-0.773536
5	1	0	-0.531218	1.692603	1.413954
6	8	0	1.250739	3.477096	0.426470
7	8	0	3.389001	-0.106247	-1.673065
8	6	0	0.310415	4.047686	1.320083
9	1	0	0.388873	3.592732	2.314288
10	1	0	0.554512	5.108328	1.382210
11	1	0	-0.711966	3.925893	0.943518
12	6	0	3.200562	-1.306005	-2.420842
13	1	0	4.049062	-1.367767	-3.103049
14	1	0	3.201913	-2.188735	-1.772878
15	1	0	2.271193	-1.266297	-2.999847
16	6	0	1.514688	-0.663697	-0.180751
17	1	0	1.300316	-1.518425	-0.827965
18	7	0	2.316069	-1.213335	0.921648
19	6	0	3.016868	-0.510193	1.872181
20	6	0	2.411702	-2.528673	1.247576
21	6	0	3.515005	-1.454586	2.734140
22	1	0	3.098481	0.566428	1.842172
23	1	0	1.935612	-3.295304	0.650105
24	1	0	4.128262	-1.297649	3.610337
25	7	0	3.131890	-2.714388	2.335128
26	6	0	3.019698	2.698200	-1.475528
27	1	0	2.394268	3.459446	-1.951056
28	1	0	3.675928	3.216876	-0.768283
29	1	0	3.638664	2.219133	-2.234591
30	6	0	0.156597	-0.112801	0.316169
31	1	0	-0.138400	-0.726687	1.173676

32	6	0	-0.884018	-0.377987	-0.789918
33	8	0	-1.139013	-1.741008	-0.807370
34	8	0	-0.925537	0.275712	-1.836706
35	6	0	-2.635324	0.174389	0.273744
36	6	0	-2.014416	-2.180251	-1.842247
37	7	0	-3.390053	1.250403	-0.047727
38	7	0	-3.448678	-0.573527	1.057525
39	1	0	-2.970973	-1.646126	-1.745712
40	1	0	-1.586588	-1.929537	-2.818024
41	6	0	-2.203056	-3.674484	-1.687530
42	6	0	-4.648758	1.181067	0.521269
43	6	0	-2.951333	2.355919	-0.892343
44	6	0	-4.685793	0.022051	1.226463
45	6	0	-3.065118	-1.824159	1.700618
46	1	0	-2.871941	-4.047671	-2.468519
47	1	0	-1.245207	-4.195900	-1.777251
48	1	0	-2.640402	-3.916209	-0.714232
49	1	0	-5.391596	1.951136	0.375177
50	1	0	-1.891934	2.225547	-1.105587
51	1	0	-3.511816	2.351577	-1.830509
52	1	0	-3.125595	3.299202	-0.370024
53	1	0	-5.466673	-0.425630	1.823063
54	1	0	-2.942971	-1.669338	2.775491
55	1	0	-3.839689	-2.574772	1.529025
56	1	0	-2.126873	-2.161136	1.263301

Cartesian coordinates of INT4_C4

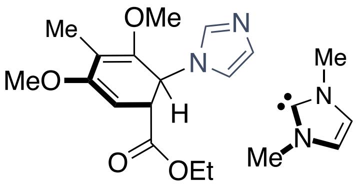

Center Number	Atomic Number	Atomic Type	Coordinates (Angstroms) X	Y	Z
1	6	0	-2.520501	-0.035562	1.013533
2	6	0	-0.909068	1.489504	-0.801706
3	6	0	-2.038572	1.978380	-0.261442
4	6	0	-2.803468	1.260681	0.780269
5	1	0	-0.338722	2.021155	-1.552876
6	8	0	-2.625833	3.155037	-0.596912
7	8	0	-3.337419	-0.761817	1.826282
8	6	0	-1.964498	3.947630	-1.570591
9	1	0	-1.900160	3.417030	-2.527318
10	1	0	-2.567854	4.847468	-1.689847
11	1	0	-0.957543	4.218211	-1.233628
12	6	0	-2.789021	-1.882338	2.516150
13	1	0	-3.557749	-2.201339	3.220257
14	1	0	-2.570017	-2.707708	1.831031
15	1	0	-1.885566	-1.600988	3.068736
16	6	0	-1.427829	-0.763948	0.266938
17	1	0	-0.927100	-1.473920	0.928147
18	7	0	-2.016807	-1.584254	-0.796796
19	6	0	-2.976087	-1.215955	-1.710713
20	6	0	-1.625075	-2.846790	-1.116070
21	6	0	-3.121188	-2.295701	-2.543877
22	1	0	-3.456458	-0.249195	-1.679230
23	1	0	-0.868977	-3.366478	-0.541757
24	1	0	-3.789044	-2.398929	-3.387362
25	7	0	-2.273025	-3.310782	-2.163652
26	6	0	-3.925138	1.979764	1.474590
27	1	0	-3.590371	2.956401	1.835205
28	1	0	-4.759755	2.158520	0.787882
29	1	0	-4.291320	1.392470	2.317088
30	6	0	-0.352367	0.205387	-0.270847
31	1	0	0.190499	-0.328569	-1.059905
32	6	0	0.699452	0.453576	0.816556

33	8	0	1.274566	-0.697994	1.186413
34	8	0	0.996115	1.526771	1.287940
35	6	0	3.094686	0.513226	-1.204269
36	6	0	2.359979	-0.593292	2.128355
37	7	0	4.052925	1.248344	-0.576782
38	7	0	3.579183	-0.755272	-1.091497
39	1	0	3.116447	0.074659	1.704039
40	1	0	1.980957	-0.146557	3.052390
41	6	0	2.898062	-1.986096	2.357133
42	6	0	5.099279	0.474817	-0.104546
43	6	0	3.981810	2.689185	-0.393046
44	6	0	4.794247	-0.807275	-0.431126
45	6	0	2.860769	-1.932974	-1.552277
46	1	0	3.739566	-1.940949	3.054329
47	1	0	2.129285	-2.636628	2.784399
48	1	0	3.251276	-2.426228	1.419941
49	1	0	5.951323	0.896245	0.408289
50	1	0	3.065963	3.048255	-0.861286
51	1	0	3.959977	2.930901	0.672679
52	1	0	4.844655	3.172965	-0.857637
53	1	0	5.326657	-1.731451	-0.259951
54	1	0	2.195660	-1.642443	-2.365807
55	1	0	3.571330	-2.677536	-1.916954
56	1	0	2.268833	-2.360899	-0.737633

Cartesian coordinates of NHC–H$^+$

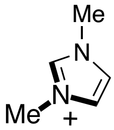

Center Number	Atomic Number	Atomic Type	Coordinates (Angstroms) X	Y	Z
1	7	0	-1.081175	-0.083936	-0.000154
2	7	0	1.081147	-0.083919	0.000233
3	6	0	-0.680883	1.233607	0.000009
4	6	0	-2.467158	-0.550547	0.000119
5	6	0	0.680794	1.233596	-0.000071
6	6	0	2.467230	-0.550497	0.000054
7	1	0	-1.394050	2.043730	0.000025
8	1	0	-2.462735	-1.639463	-0.000668
9	1	0	-2.967917	-0.180763	0.895036
10	1	0	-2.968554	-0.179547	-0.893923
11	1	0	1.393955	2.043722	-0.000030
12	1	0	2.462882	-1.639388	-0.000823
13	1	0	2.968122	-0.179517	-0.894240
14	1	0	2.968458	-0.180624	0.894659
15	6	0	0.000014	-0.861382	-0.000222
16	1	0	0.000045	-1.941827	0.000077

Cartesian coordinates of TS4_C2

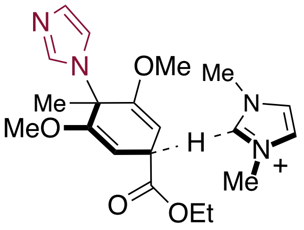

Center Number	Atomic Number	Atomic Type	Coordinates (Angstroms) X	Y	Z
1	6	0	-0.297592	-1.883676	-0.329058
2	6	0	0.954779	-1.401156	-0.376524
3	6	0	0.547818	-0.147429	1.731792
4	6	0	-0.697164	-0.645504	1.797831
5	1	0	1.631138	-1.703153	-1.168110
6	1	0	0.943452	0.465736	2.534610
7	8	0	-1.581838	-0.444928	2.818874
8	8	0	-0.839804	-2.770699	-1.214902
9	6	0	-1.171818	0.415924	3.864535
10	1	0	-0.944774	1.418499	3.480210
11	1	0	-2.009124	0.471504	4.560498
12	1	0	-0.290478	0.018190	4.381391
13	6	0	-0.024823	-3.187304	-2.294862
14	1	0	-0.631175	-3.871898	-2.888469
15	1	0	0.277087	-2.332906	-2.912638
16	1	0	0.869935	-3.707687	-1.933204
17	6	0	-1.321828	-1.523844	0.733524
18	7	0	-2.440131	-0.764295	0.092211
19	6	0	-3.436589	-1.284360	-0.674485
20	6	0	-2.626395	0.598490	0.111618
21	1	0	-3.518686	-2.343008	-0.869975
22	6	0	-3.741946	0.835043	-0.649463
23	1	0	-1.964292	1.261593	0.650053
24	1	0	-4.210551	1.785574	-0.864133
25	7	0	-4.241641	-0.348795	-1.140294
26	6	0	-1.903279	-2.795059	1.375433
27	1	0	-2.300686	-3.471721	0.618017
28	1	0	-1.103343	-3.312809	1.912040
29	1	0	-2.693458	-2.532144	2.080583
30	6	0	1.408293	-0.355804	0.556022
31	1	0	1.126745	0.717943	-0.186637

32	6	0	2.836702	-0.144779	0.775222
33	8	0	3.609106	-0.663535	-0.205749
34	6	0	5.014424	-0.375643	-0.132026
35	1	0	5.157125	0.709125	-0.152567
36	1	0	5.405256	-0.753017	0.817659
37	6	0	5.675629	-1.048288	-1.313907
38	1	0	6.751635	-0.853489	-1.292110
39	1	0	5.520004	-2.130455	-1.282137
40	1	0	5.273858	-0.663433	-2.255633
41	8	0	3.318196	0.525684	1.682493
42	6	0	0.446082	1.871381	-0.867692
43	7	0	0.269011	3.015554	-0.172499
44	6	0	-1.192733	3.108362	-1.802694
45	6	0	-0.726351	3.796247	-0.726587
46	1	0	-1.967498	3.344316	-2.516896
47	1	0	-1.002456	4.758140	-0.321095
48	7	0	-0.460499	1.939876	-1.866619
49	6	0	1.037824	3.376092	1.012448
50	1	0	1.749073	2.574794	1.220686
51	1	0	1.580574	4.306773	0.833833
52	1	0	0.366291	3.502639	1.864614
53	6	0	-0.717020	0.868299	-2.820070
54	1	0	-1.541863	0.241434	-2.465784
55	1	0	-0.976405	1.302193	-3.787155
56	1	0	0.186075	0.266743	-2.919323

Cartesian coordinates of INT5_C2

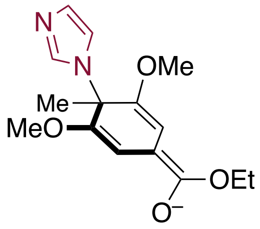

Center Number	Atomic Number	Atomic Type	Coordinates (Angstroms) X	Y	Z
1	6	0	-0.346685	-1.069355	0.719589
2	6	0	0.941220	-0.783421	0.412674
3	6	0	0.383075	1.595197	0.223465
4	6	0	-0.908684	1.329613	0.528953
5	1	0	1.678213	-1.578642	0.385812
6	1	0	0.706263	2.616720	0.049038
7	8	0	-1.916422	2.265082	0.631933
8	8	0	-0.842531	-2.326324	0.997692
9	6	0	-1.568547	3.605776	0.366580
10	1	0	-1.183885	3.721571	-0.654984
11	1	0	-2.482701	4.190931	0.478834
12	1	0	-0.813720	3.970152	1.075588
13	6	0	0.063062	-3.403063	0.904813
14	1	0	-0.505318	-4.306527	1.131410
15	1	0	0.484949	-3.480726	-0.105737
16	1	0	0.883249	-3.299599	1.627198
17	6	0	-1.463997	-0.055869	0.750569
18	7	0	-2.408813	-0.368081	-0.390599
19	6	0	-3.733825	-0.653479	-0.364293
20	6	0	-2.022943	-0.397961	-1.707155
21	1	0	-4.300741	-0.696651	0.553460
22	6	0	-3.152023	-0.706112	-2.424047
23	1	0	-1.002392	-0.200188	-2.002569
24	1	0	-3.252590	-0.821135	-3.494559
25	7	0	-4.221648	-0.865079	-1.575115
26	6	0	-2.266426	-0.140108	2.058826
27	1	0	-2.675210	-1.142149	2.205858
28	1	0	-1.586501	0.080810	2.885626
29	1	0	-3.075885	0.592834	2.072228
30	6	0	1.377487	0.559479	0.130898
31	6	0	2.713075	0.899737	-0.179121

32	8	0	3.569330	-0.186715	-0.213539
33	6	0	4.930876	0.097023	-0.518428
34	1	0	4.997547	0.586169	-1.496577
35	1	0	5.341288	0.788520	0.226064
36	6	0	5.681853	-1.218292	-0.513690
37	1	0	6.737784	-1.046233	-0.743397
38	1	0	5.615735	-1.699435	0.466830
39	1	0	5.272221	-1.901459	-1.263852
40	8	0	3.165848	2.035729	-0.412844

Cartesian coordinates of TS4_C4

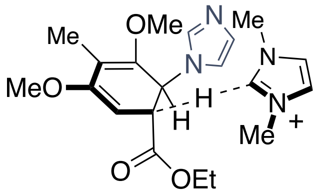

Center Number	Atomic Number	Atomic Type	Coordinates (Angstroms) X	Y	Z
1	6	0	2.548662	-0.436502	0.206481
2	6	0	0.743116	1.602261	-0.568926
3	6	0	1.989003	1.890498	-0.143017
4	6	0	2.975356	0.836627	0.150856
5	1	0	0.056585	2.391615	-0.852717
6	8	0	2.501423	3.145965	0.031494
7	8	0	3.407082	-1.475082	0.488537
8	6	0	1.654138	4.231135	-0.293265
9	1	0	0.754615	4.229474	0.335150
10	1	0	2.229907	5.137295	-0.102779
11	1	0	1.358390	4.198349	-1.348850
12	6	0	3.884102	-2.143993	-0.677981
13	1	0	4.497224	-2.979450	-0.336184
14	1	0	3.053052	-2.527667	-1.282815
15	1	0	4.490595	-1.464725	-1.288807
16	6	0	1.096958	-0.835310	0.071314
17	1	0	1.048089	-1.789026	-0.459694
18	7	0	0.584841	-1.143854	1.429384
19	6	0	0.233669	-0.234690	2.396530
20	6	0	0.486643	-2.376273	1.987119
21	6	0	-0.079582	-0.979506	3.507174
22	1	0	0.240539	0.829192	2.207009
23	1	0	0.722017	-3.272379	1.428308
24	1	0	-0.414687	-0.626647	4.472770
25	7	0	0.077631	-2.319379	3.241399
26	6	0	4.412752	1.223759	0.353493
27	1	0	4.808825	1.726882	-0.534650
28	1	0	4.507840	1.925746	1.187609
29	1	0	5.020393	0.342722	0.564935
30	6	0	0.260313	0.203530	-0.656021
31	1	0	-1.032480	0.290908	-0.123933

32	6	0	-0.295062	-0.166077	-1.953225
33	8	0	-0.462243	-1.502646	-2.109757
34	8	0	-0.732146	0.628041	-2.780697
35	6	0	-2.431057	0.547035	0.263527
36	6	0	-1.153376	-1.928659	-3.293917
37	7	0	-3.159626	1.645630	-0.032571
38	7	0	-3.227826	-0.181123	1.072854
39	1	0	-2.150786	-1.478734	-3.304736
40	1	0	-0.608466	-1.572159	-4.173377
41	6	0	-1.222525	-3.438860	-3.254936
42	6	0	-4.391675	1.612370	0.589599
43	6	0	-2.707925	2.732786	-0.894626
44	6	0	-4.436510	0.449416	1.290444
45	6	0	-2.891063	-1.494805	1.605305
46	1	0	-1.744168	-3.806207	-4.143267
47	1	0	-0.218870	-3.873716	-3.239476
48	1	0	-1.766171	-3.780029	-2.369045
49	1	0	-5.118131	2.402401	0.472654
50	1	0	-2.038952	2.321792	-1.652513
51	1	0	-3.577272	3.177866	-1.380474
52	1	0	-2.191448	3.494677	-0.305505
53	1	0	-5.210680	0.016608	1.906117
54	1	0	-1.901308	-1.767649	1.241539
55	1	0	-2.886202	-1.467376	2.696247
56	1	0	-3.622119	-2.227346	1.256840

Cartesian coordinates of INT5_C4

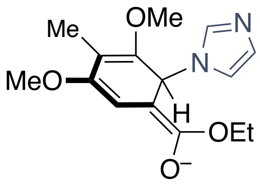

Center Number	Atomic Number	Atomic Type	Coordinates (Angstroms) X	Y	Z
1	6	0	1.165767	1.028060	0.810243
2	6	0	1.188326	-1.590090	-0.216075
3	6	0	2.347654	-0.888091	-0.093641
4	6	0	2.348102	0.434271	0.511892
5	1	0	1.175334	-2.609744	-0.588205
6	8	0	3.607855	-1.334421	-0.454615
7	8	0	1.165908	2.311282	1.321061
8	6	0	3.677659	-2.647713	-0.959002
9	1	0	3.082167	-2.756944	-1.875539
10	1	0	4.727952	-2.839442	-1.185694
11	1	0	3.325362	-3.379589	-0.219752
12	6	0	0.339330	2.530157	2.456102
13	1	0	0.654524	3.481433	2.889393
14	1	0	-0.719989	2.602940	2.183814
15	1	0	0.470744	1.732149	3.197330
16	6	0	-0.173269	0.444246	0.447315
17	1	0	-0.912992	0.648351	1.228494
18	7	0	-0.728255	1.232848	-0.722464
19	6	0	-0.187304	1.302089	-1.979321
20	6	0	-1.845726	1.992424	-0.730607
21	6	0	-1.024085	2.119500	-2.701800
22	1	0	0.718582	0.771961	-2.237824
23	1	0	-2.467909	2.096092	0.148853
24	1	0	-0.936836	2.417300	-3.737834
25	7	0	-2.064037	2.549660	-1.911059
26	6	0	3.670151	1.095383	0.795647
27	1	0	4.315475	0.432956	1.381154
28	1	0	4.202800	1.312532	-0.136881
29	1	0	3.527073	2.029143	1.340109
30	6	0	-0.067690	-1.022111	0.181363
31	6	0	-1.221921	-1.837764	0.189434
32	8	0	-2.372773	-1.142606	0.511773

33	8	0	-1.292319	-3.055860	-0.051760
34	6	0	-3.572817	-1.906638	0.583688
35	1	0	-3.757149	-2.394473	-0.379457
36	1	0	-3.466137	-2.693137	1.339609
37	6	0	-4.696315	-0.954610	0.937385
38	1	0	-5.641506	-1.501496	1.006959
39	1	0	-4.506397	-0.470229	1.900209
40	1	0	-4.801604	-0.179207	0.172535

Cartesian coordinates of product (3aa)

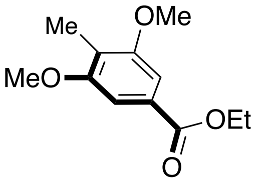

Center Number	Atomic Number	Atomic Type	Coordinates (Angstroms) X	Y	Z
1	6	0	1.072032	1.425159	0.000004
2	6	0	0.673658	-1.344948	0.000121
3	6	0	1.963503	-0.812015	0.000065
4	6	0	2.187805	0.574134	0.000072
5	1	0	0.490081	-2.411919	0.000196
6	8	0	3.088438	-1.573059	0.000128
7	8	0	1.342996	2.757549	-0.000094
8	6	0	2.934556	-2.985540	-0.000564
9	1	0	2.402357	-3.323297	-0.896463
10	1	0	3.944361	-3.395450	-0.000944
11	1	0	2.402692	-3.324225	0.895186
12	6	0	0.249505	3.663415	-0.000057
13	1	0	0.689729	4.660479	-0.000170
14	1	0	-0.368293	3.536829	-0.895833
15	1	0	-0.368086	3.536920	0.895887
16	6	0	-0.230718	0.914774	-0.000010
17	1	0	-1.093373	1.567391	-0.000060
18	6	0	3.595553	1.098131	0.000138
19	1	0	4.139483	0.738390	0.879697
20	1	0	4.139626	0.738122	-0.879231
21	1	0	3.607755	2.187401	-0.000003
22	6	0	-0.410853	-0.468104	0.000144
23	6	0	-1.777673	-1.067456	0.000262
24	8	0	-2.749436	-0.150761	0.000277
25	8	0	-1.994433	-2.263306	0.000252
26	6	0	-4.102014	-0.651077	0.000166
27	1	0	-4.241224	-1.275416	-0.887008
28	1	0	-4.241648	-1.274615	0.887842
29	6	0	-5.024383	0.545462	-0.000568
30	1	0	-6.062695	0.202713	-0.000422
31	1	0	-4.863599	1.161342	0.888574
32	1	0	-4.863504	1.160346	-0.890379

Cartesian coordinates of imidazolide anion

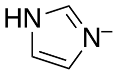

Center Number	Atomic Number	Atomic Type	Coordinates (Angstroms) X	Y	Z
1	6	0	-0.904132	-0.693105	-0.000084
2	6	0	-0.903210	0.694223	-0.000086
3	6	0	1.108920	-0.000586	-0.000010
4	7	0	0.390889	-1.137944	0.000056
5	1	0	-1.744628	-1.377752	-0.000167
6	1	0	-1.742561	1.380235	-0.000165
7	1	0	2.194088	-0.001615	0.000057
8	7	0	0.392487	1.137365	0.000137

Cartesian coordinates of TS5_C2

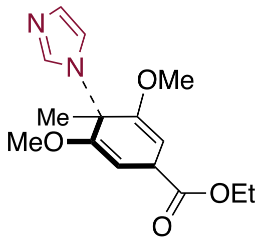

Center Number	Atomic Number	Atomic Type	Coordinates (Angstroms) X	Y	Z
1	6	0	0.372405	-0.982989	-0.973807
2	6	0	-0.911880	-0.738700	-0.555970
3	6	0	-0.358922	1.610011	-0.262048
4	6	0	0.924473	1.358837	-0.677293
5	1	0	-1.646792	-1.534822	-0.545490
6	1	0	-0.680415	2.617926	-0.023443
7	8	0	1.900947	2.320708	-0.808267
8	8	0	0.833357	-2.214962	-1.383064
9	6	0	1.537965	3.649371	-0.500458
10	1	0	1.222241	3.743908	0.546315
11	1	0	2.429145	4.257009	-0.664694
12	1	0	0.730384	4.005862	-1.152793
13	6	0	-0.090895	-3.281179	-1.381405
14	1	0	0.452195	-4.159074	-1.734606
15	1	0	-0.474923	-3.474745	-0.371764
16	1	0	-0.934556	-3.081498	-2.054750
17	6	0	1.424144	0.019425	-0.937248
18	7	0	2.425146	-0.401720	0.582525
19	6	0	3.668501	-0.881389	0.781290
20	6	0	1.856154	-0.317985	1.820105
21	1	0	4.361082	-1.054853	-0.032286
22	6	0	2.799451	-0.754800	2.729237
23	1	0	0.843321	0.040609	1.955434
24	1	0	2.717232	-0.829733	3.806223
25	7	0	3.947677	-1.110666	2.066414
26	6	0	2.533852	-0.112522	-1.962380
27	1	0	2.922652	-1.131288	-1.983436
28	1	0	2.126991	0.120803	-2.952652
29	1	0	3.343027	0.586517	-1.747929
30	6	0	-1.313303	0.566760	-0.174073

31	6	0	-2.662007	0.883548	0.232570
32	8	0	-3.491702	-0.196869	0.249073
33	6	0	-4.845107	0.049950	0.642131
34	1	0	-4.856292	0.477223	1.650097
35	1	0	-5.291888	0.781468	-0.039074
36	6	0	-5.578867	-1.272397	0.592836
37	1	0	-6.622162	-1.127167	0.887957
38	1	0	-5.560606	-1.689073	-0.418475
39	1	0	-5.123563	-1.994678	1.276631
40	8	0	-3.080428	1.998206	0.544164

Cartesian coordinates of TS5_C4

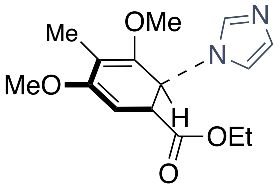

Center Number	Atomic Number	Atomic Type	Coordinates (Angstroms) X	Y	Z
1	6	0	1.246194	0.850729	0.889251
2	6	0	0.957164	-1.646677	-0.315275
3	6	0	2.186571	-1.046331	-0.262363
4	6	0	2.357241	0.208919	0.421013
5	1	0	0.822204	-2.635131	-0.740896
6	8	0	3.355669	-1.581542	-0.759697
7	8	0	1.355175	2.069755	1.538227
8	6	0	3.251358	-2.835958	-1.398049
9	1	0	2.571812	-2.789149	-2.258811
10	1	0	4.254743	-3.091652	-1.741957
11	1	0	2.897123	-3.609405	-0.704531
12	6	0	1.320679	1.952881	2.955874
13	1	0	1.368504	2.965103	3.363070
14	1	0	0.393253	1.471376	3.290407
15	1	0	2.177291	1.370369	3.318172
16	6	0	-0.104101	0.383430	0.615075
17	1	0	-0.867936	0.757842	1.290539
18	7	0	-0.666856	1.423899	-0.806942
19	6	0	-1.282124	0.936979	-1.920772
20	6	0	-0.596877	2.758039	-0.972440
21	6	0	-1.562743	2.022458	-2.729965
22	1	0	-1.466009	-0.121948	-2.053495
23	1	0	-0.141450	3.404224	-0.232923
24	1	0	-2.043360	2.035097	-3.700186
25	7	0	-1.126137	3.174557	-2.126181
26	6	0	3.743702	0.755152	0.619660
27	1	0	4.363208	0.052440	1.186818
28	1	0	4.237477	0.905615	-0.345872
29	1	0	3.711129	1.708775	1.148622
30	6	0	-0.186944	-0.997102	0.237767
31	6	0	-1.445202	-1.700948	0.251309
32	8	0	-2.476594	-0.945007	0.719773

33	8	0	-1.632068	-2.863163	-0.110658
34	6	0	-3.764077	-1.567403	0.738421
35	1	0	-4.021837	-1.890932	-0.275461
36	1	0	-3.728921	-2.456249	1.377339
37	6	0	-4.751198	-0.546025	1.260061
38	1	0	-5.755048	-0.980176	1.283705
39	1	0	-4.486395	-0.231739	2.273894
40	1	0	-4.770156	0.337141	0.614862

■ **Supplementary References** ■

1. Morofuji, T.; Kurokawa, T.; Chitose, Y.; Adachi, C.; Kano, N. Trifluoromethylated thermally activated delayed fluorescence molecule as a versatile photocatalyst for electron-transfer- and energy-transfer-driven reactions. *Org. Biomol. Chem.* **2022**, *20*, 9600–9603.
2. Speckmeier, E.; Fischer, T. G.; Zeitler, K. A Toolbox Approach To Construct Broadly Applicable Metal-Free Catalysts for Photoredox Chemistry: Deliberate Tuning of Redox Potentials and Importance of Halogens in Donor–Acceptor Cyanoarenes. *J. Am. Chem. Soc.* **2018**, *140*, 15353–15365.
3. MacKenzie, I. A.; Wang, L.; Onuska, N. P. R.; Williams, O. F.; Begman, K.; Moran, A. M.; Dunietz, B. D.; Nicewicz, D. A. Discovery and characterization of an acridine radical photoreductant. *Nature* **2020**, *580*, 76–80.
4. Xu, Y.; Chen, W.; Pu, R.; Ding, J.; An, Q.; Yang, Y.; Liu, W.; Zuo, Z. Selective monodeuteration enabled by bisphosphonium catalyzed ring opening processes. *Nat. Commun.* **2024**, *15*, 9366.
5. Mizutani, T.; Wada, K.; Kitagawa, S. Porphyrin Receptors for Amines, Amino Acids, and Oligopeptides in Water. *J. Am. Chem. Soc.* **1999**, *121*, 11425–11431.
6. Avila, E. P.; de Souza, I. F.; Oliveira, A. V. B.; Kartnaller, V.; Cajaiba, J.; de Souza, R. O. M. A.; Correa, C. C.; Amarante, G. W. Catalyst free decarboxylative trichloromethylation of aldimines. *RSC Adv.* **2016**, *6*, 108530–108537.
7. Zhang, L.; Xiong, W.; Yao, B.; Liu, H.; Li, M.; Qin, Y.; Yu, Y.; Li, X.; Chen, W.; Wu, W.; Li, J.; Wang, J.; Jiang, H. Facile synthesis of isoquinolines and isoquinoline N-oxides via a copper-catalyzed intramolecular cyclization in water. *RSC Adv.* **2022**, *12*, 30248–30252.
8. Cvengros, J.; Neufeind, S.; Becker, A.; Schmalz, H-G. Microwave-Assisted Cleavage of Aryl Methyl Ethers with Lithium Thioethoxide (LiSEt). *Synlett* **2008**, *13*, 1993–1998.
9. Masiukiewicz, E.; Mrugala, D.; Rzeszotarska, B. AN IMPROVED SYNTHESIS OF 1-METHYL-2-TRICHLOROACETYLIMIDAZOLE. *Organic Preparations and Procedures International* **2005**, *37*, 403–405.
10. Zhu, J. L.; Scheidt, K. A. Photocatalytic acyl azolium-promoted alkoxycarbonylation of trifluoroborates. *Tetrahedron* **2021**, *92*, 132288.
11. Deng, Z.; Liang, X.; Gillies, E. R. Click to Self-immolation: A “Click” Functionalization Strategy towards Triggerable Self-Immolative Homopolymers and Block Copolymers. *Angew. Chem. Int. Ed.* **2024**, *63*, e202317063.
12. de Azevedo, O. D. C. C.; Elliott, P. I. P.; Gabbutt, C. D.; Heron, B. M.; Lord, K. J.; Pullen, C. Synthesis and Photochromism of Novel Pyridyl-Substituted Naphthopyrans. *J. Org. Chem.* **2020**, *85*, 10772–10796.
13. Li, J-F.; Wang, Y-F.; Wu, Y-Y.; Liu, W-J.; Wang, J-W. Nickel-Catalyzed Esterification of Amides Under Mild Conditions. *Catalysis Letters* **2020**, *150*, 874–880.
14. Teng, B.; Shi, J.; Yao, C. Facile, highly efficient and environmentally friendly transesterification mediated by platinum dioxide and nickel oxide under essentially neutral conditions. *Green Chem.* **2018**, *20*, 2465–2471.

15. Frisch, M. J.; Trucks, G. W.; Schlegel, H. B.; Scuseria, G. E.; Robb, M. A.; Cheeseman, J. R.; Scalmani, G.; Barone, V.; Petersson, G. A.; Nakatsuji, H.; Li, X.; Caricato, M.; Marenich, A. V.; Bloino, J.; Janesko, B. G.; Gomperts, R.; Mennucci, B.; Hratchian, H. P.; Ortiz, J. V.; Izmaylov, A. F.; Sonnenberg, J. L.; Williams-Young, D.; Ding, F.; Lipparini, F.; Egidi, F.; Goings, J.; Peng, B.; Petrone, A.; Henderson, T.; Ranasinghe, D.; Zakrzewski, V. G.; Gao, J.; Rega, N.; Zheng, G.; Liang, W.; Hada, M.; Ehara, M.; Toyota, K.; Fukuda, R.; Hasegawa, J.; Ishida, M.; Nakajima, T.; Honda, Y.; Kitao, O.; Nakai, H.; Vreven, T.; Throssell, K.; Montgomery, J. A., Jr.; Peralta, J. E.; Ogliaro, F.; Bearpark, M. J.; Heyd, J. J.; Brothers, E. N.; Kudin, K. N.; Staroverov, V. N.; Keith, T. A.; Kobayashi, R.; Normand, J.; Raghavachari, K.; Rendell, A. P.; Burant, J. C.; Iyengar, S. S.; Tomasi, J.; Cossi, M.; Millam, J. M.; Klene, M.; Adamo, C.; Cammi, R.; Ochterski, J. W.; Martin, R. L.; Morokuma, K.; Farkas, O.; Foresman, J. B.; Fox, D. J. Gaussian 16, Revision C.01. *Gaussian, Inc., Wallingford CT*, **2016**.

16. (a) Montgomery, J. A.; Frisch, M. J.; Ochterski, J. W.; Petersson, G. A. A complete basis set model chemistry. VI. Use of density functional geometries and frequencies. *J. Chem. Phys.* **1999**, *110*, 2822−2827. (b) Mayer, P. M.; Parkinson, C. J.; Smith, D. M.; Radom, L. An assessment of theoretical procedures for the calculation of reliable free radical thermochemistry: A recommended new procedure. *J. Chem. Phys.***1998**, *108*, 604−615.

17. Kesharwani, M. K.; Brauer, B.; Martin, J. M. L. Frequency and Zero-Point Vibrational Energy Scale Factors for Double-Hybrid Density Functionals (and Other Selected Methods): Can Anharmonic Force Fields Be Avoided? *J. Phys. Chem. A* **2015**, *119*, 1701−1714.

18. Wood, G. P. F.; Moran, D.; Jacob, R.; Radom, L. Bond Dissociation Energies and Radical Stabilization Energies Associated with Model Peptide-Backbone Radicals. *J. Phys. Chem. A* **2005**, *109*, 6318–6325.

19. Zhao, Y.; Truhlar, D. G. The M06 suite of density functionals for main group thermochemistry, thermochemical kinetics, noncovalent interactions, excited states, and transition elements: two new functionals and systematic testing of four M06-class functionals and 12 other functionals. *Theor. Chem. Acc.* **2008**, *120*, 215–241.

20. Marenich, A. V.; Cramer, C. J.; Truhlar, D. G. Universal Solvation Model Based on Solute Electron Density and on a Continuum Model of the Solvent Defined by the Bulk Dielectric Constant and Atomic Surface Tensions. *J. Phys. Chem. B* **2009**, *113*, 6378–6396.

^{1}H NMR of **1c** (400 MHz, $CDCl_3$)

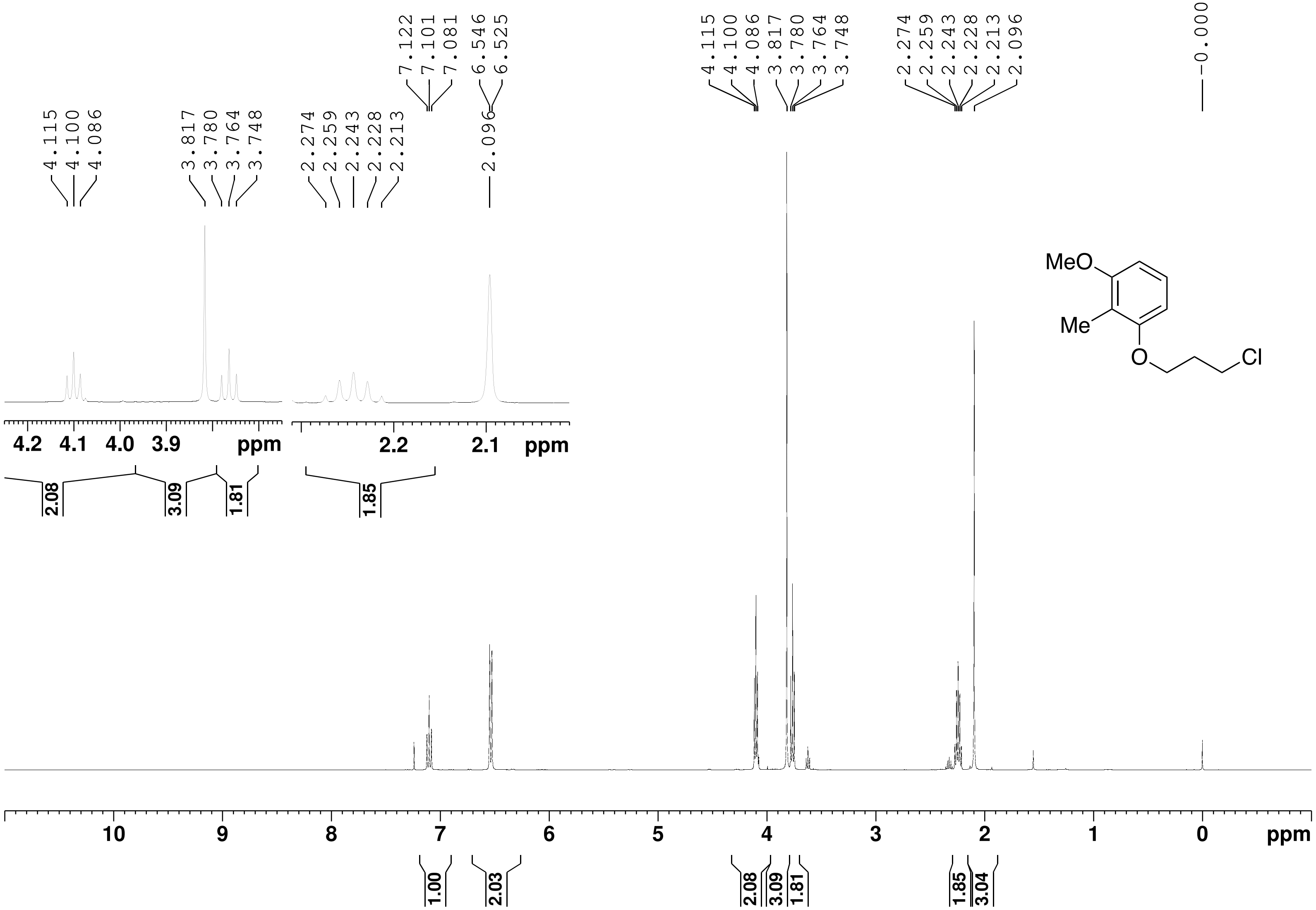

^{13}C NMR of **1c** (100.6 MHz, $CDCl_3$)

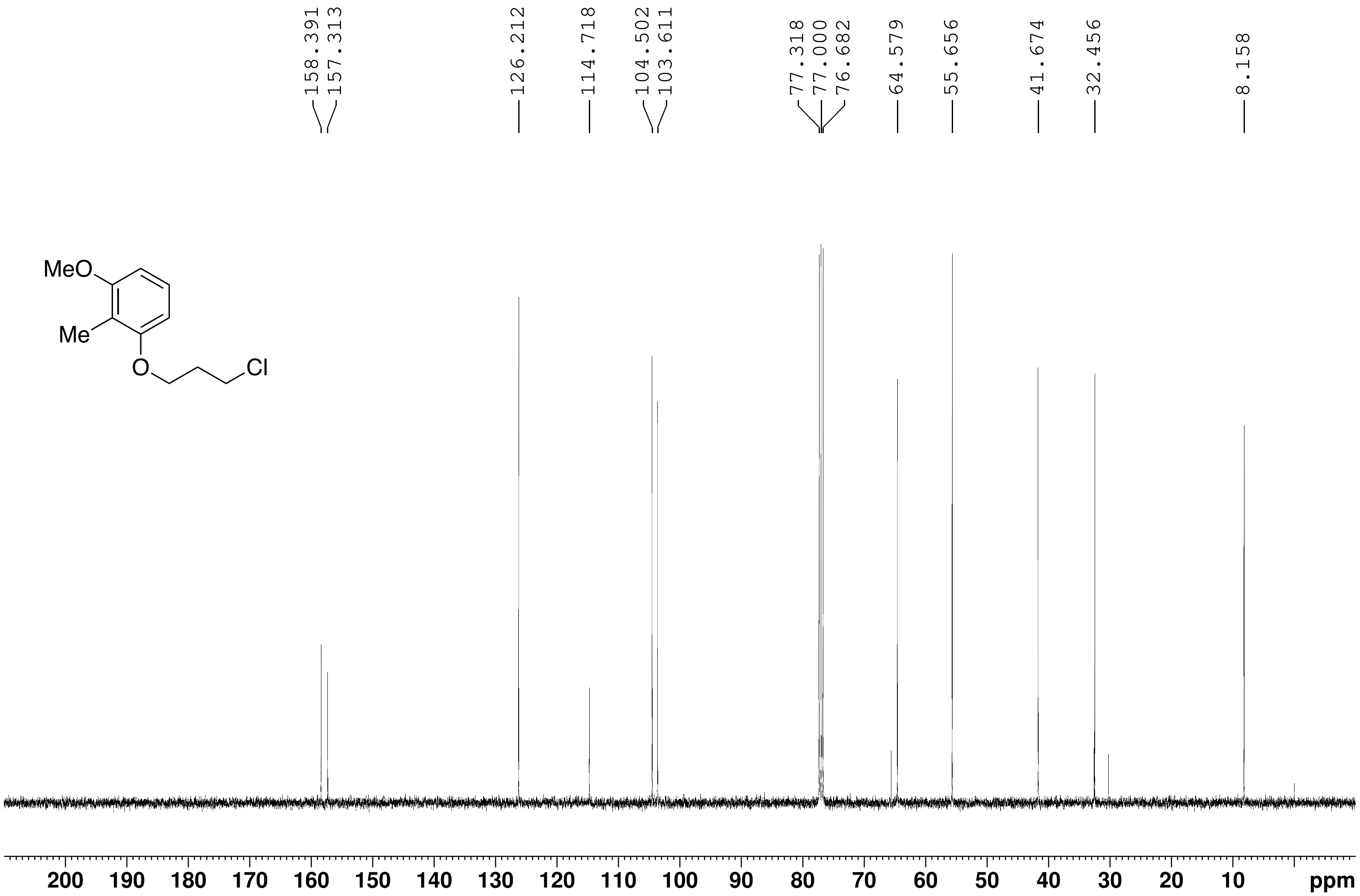

^{1}H NMR of **1d** (400 MHz, $CDCl_3$)

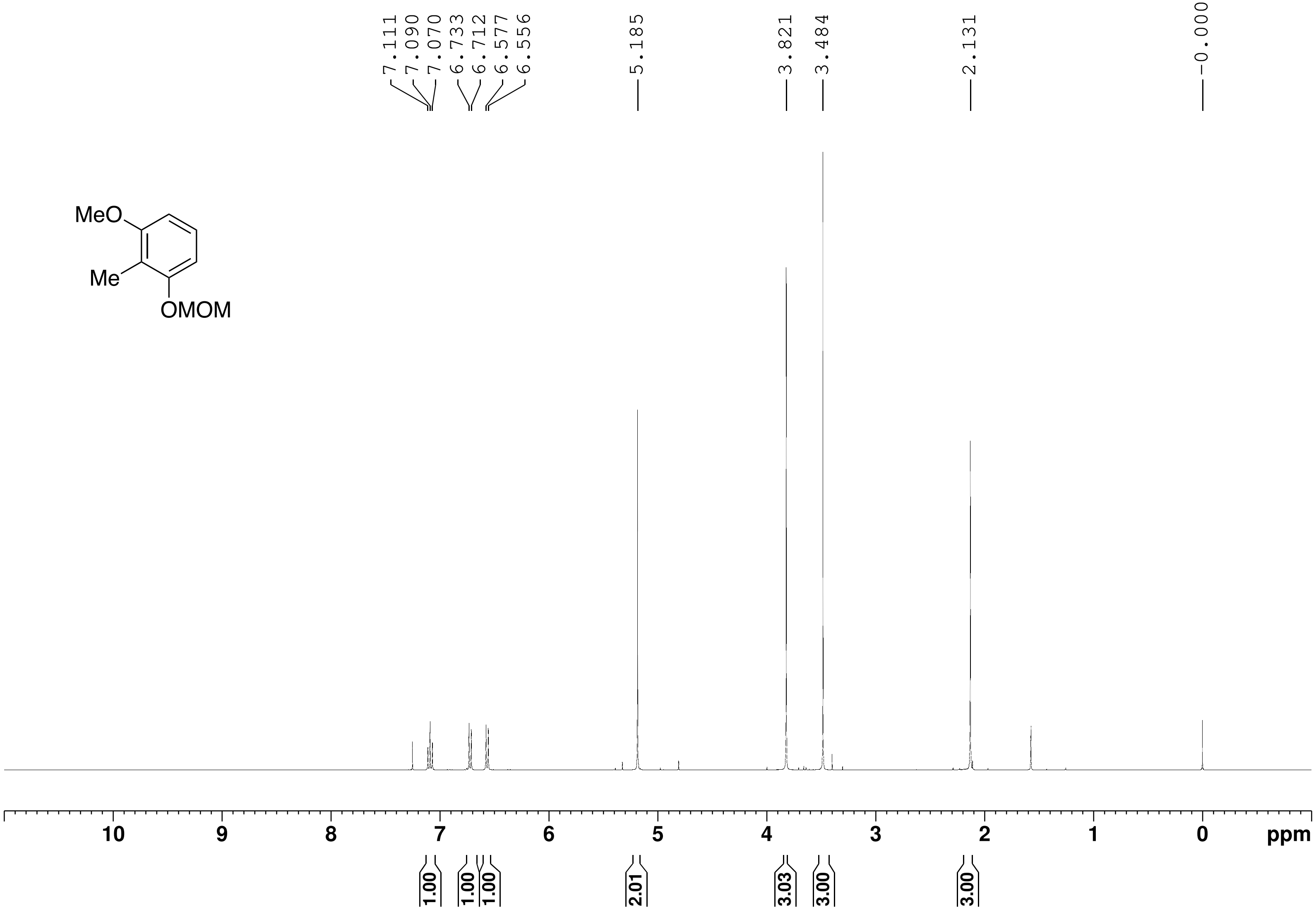

^{13}C NMR of **1d** (100.6 MHz, $CDCl_3$)

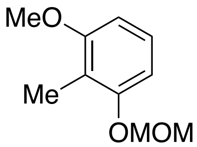

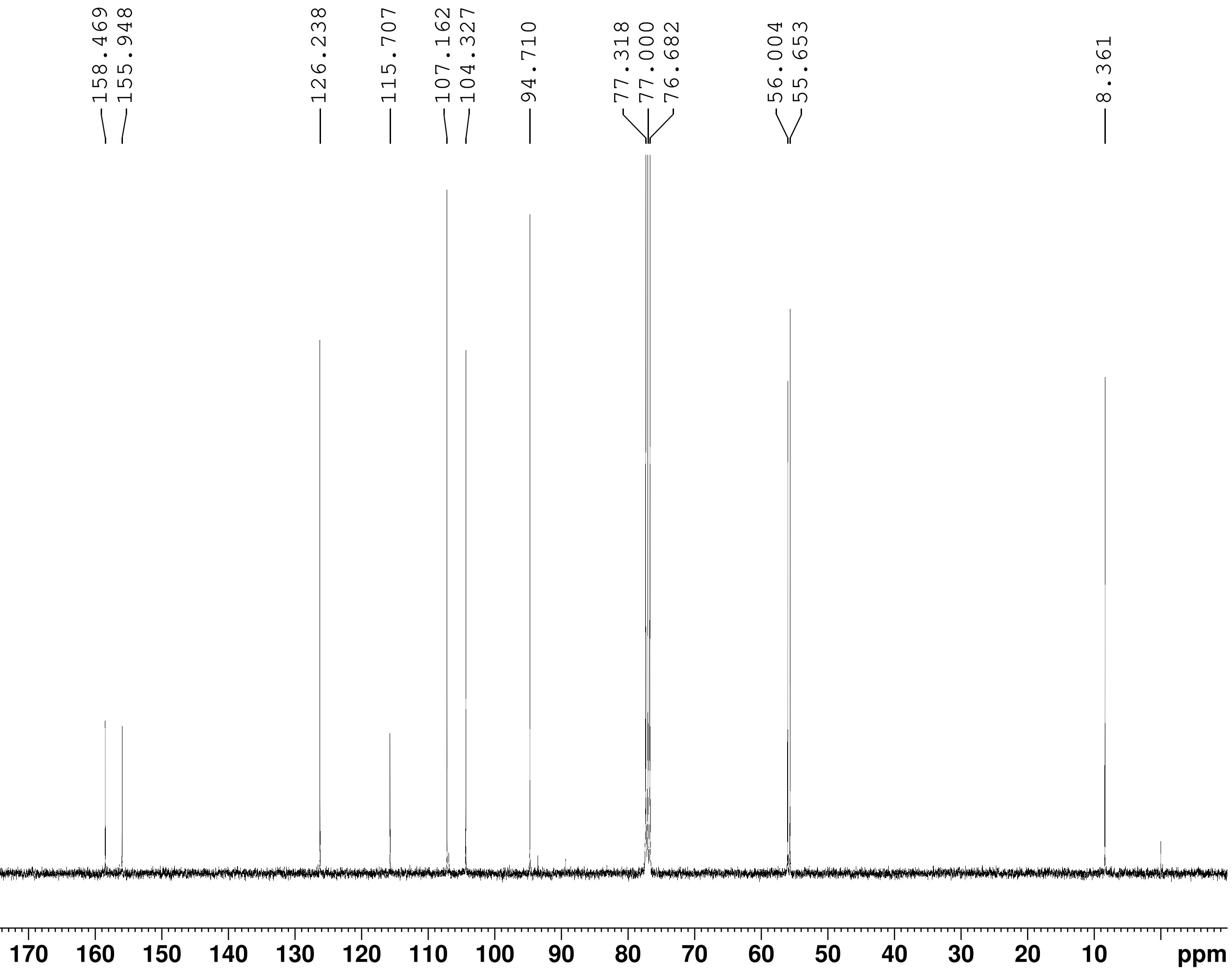

^{1}H NMR of **1e** (400 MHz, $CDCl_3$)

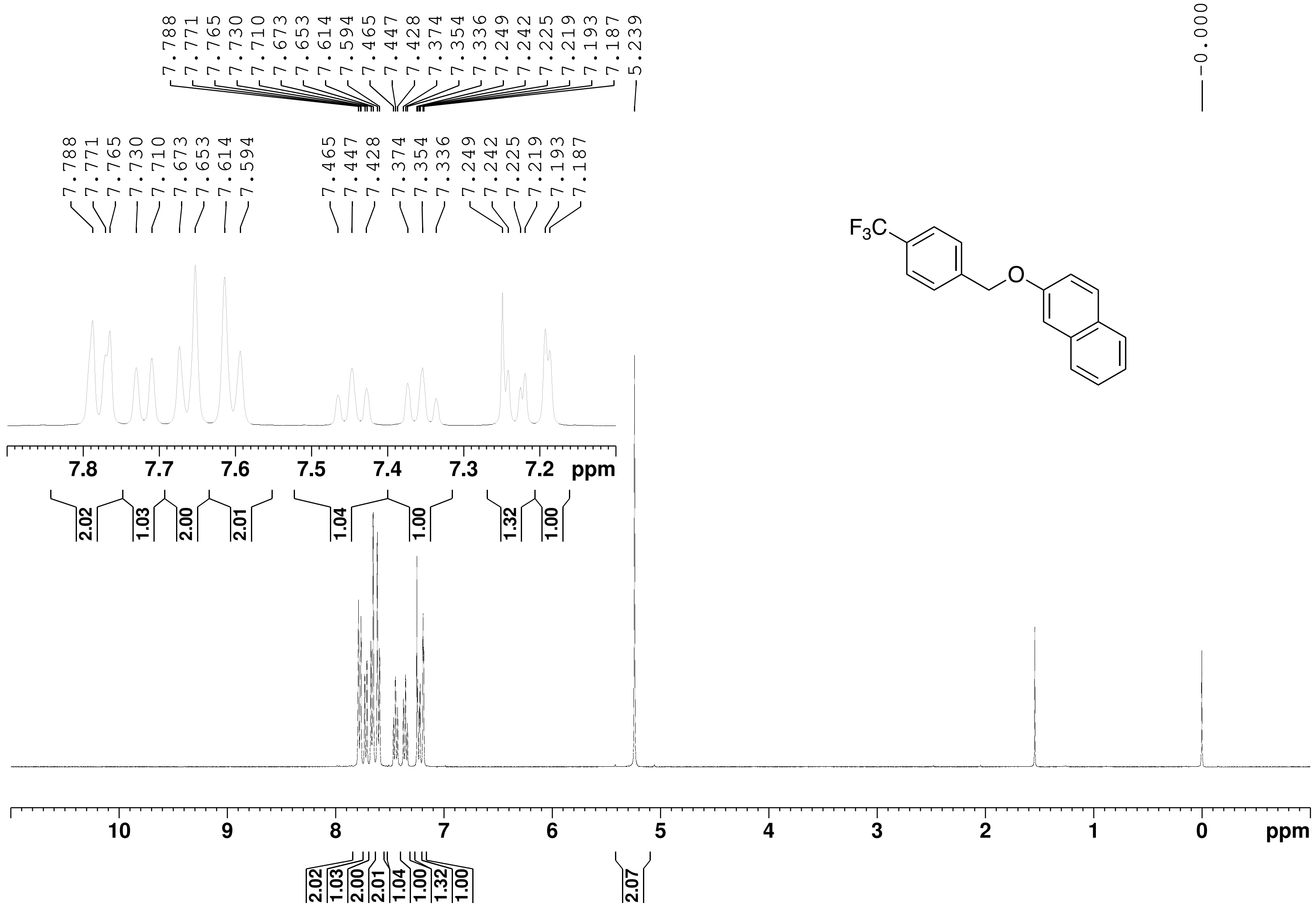

^{13}C NMR of **1e** (100.6 MHz, $CDCl_3$)

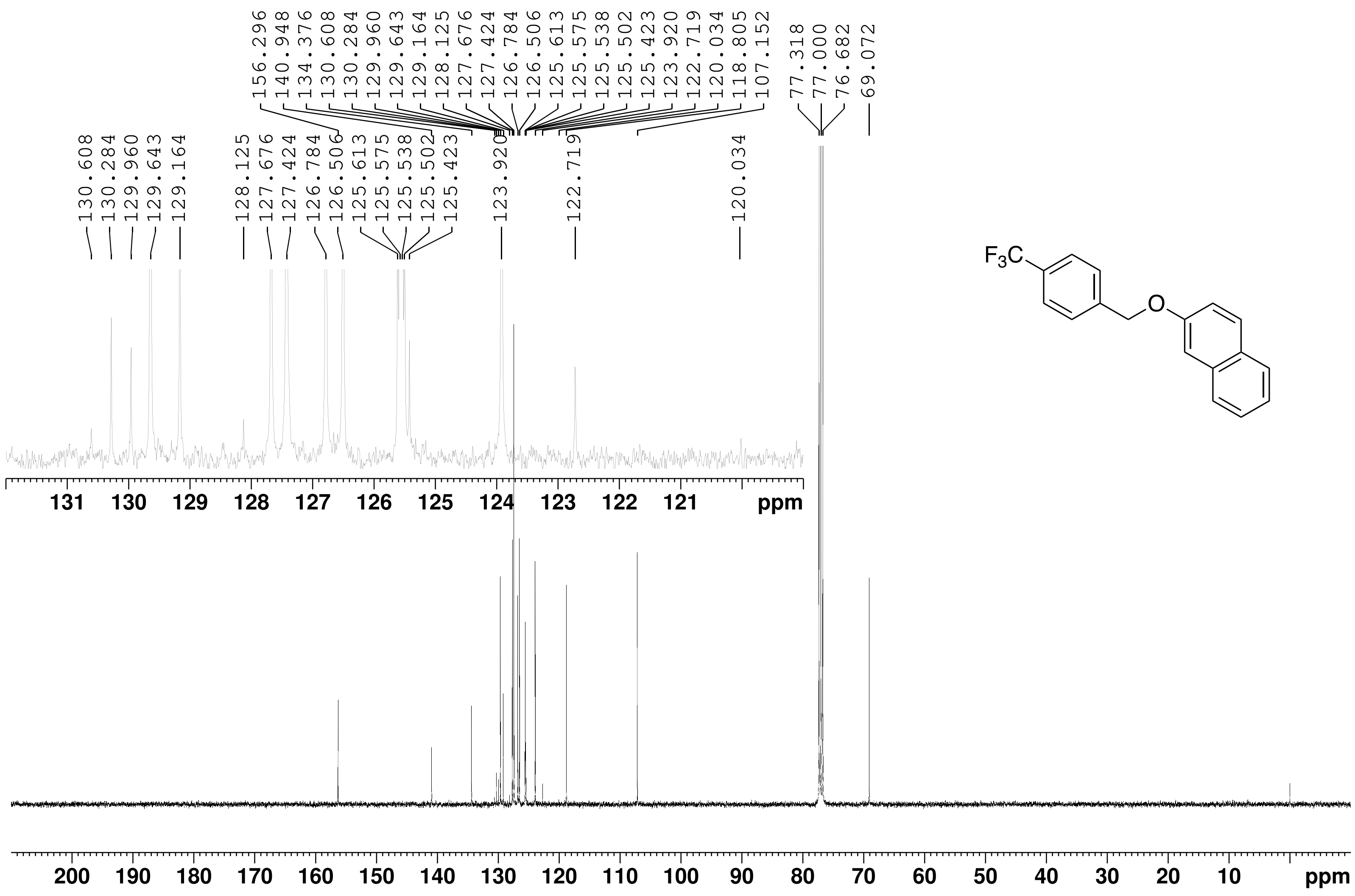

^{19}F NMR of **1e** (376.5 MHz, $CDCl_3$)

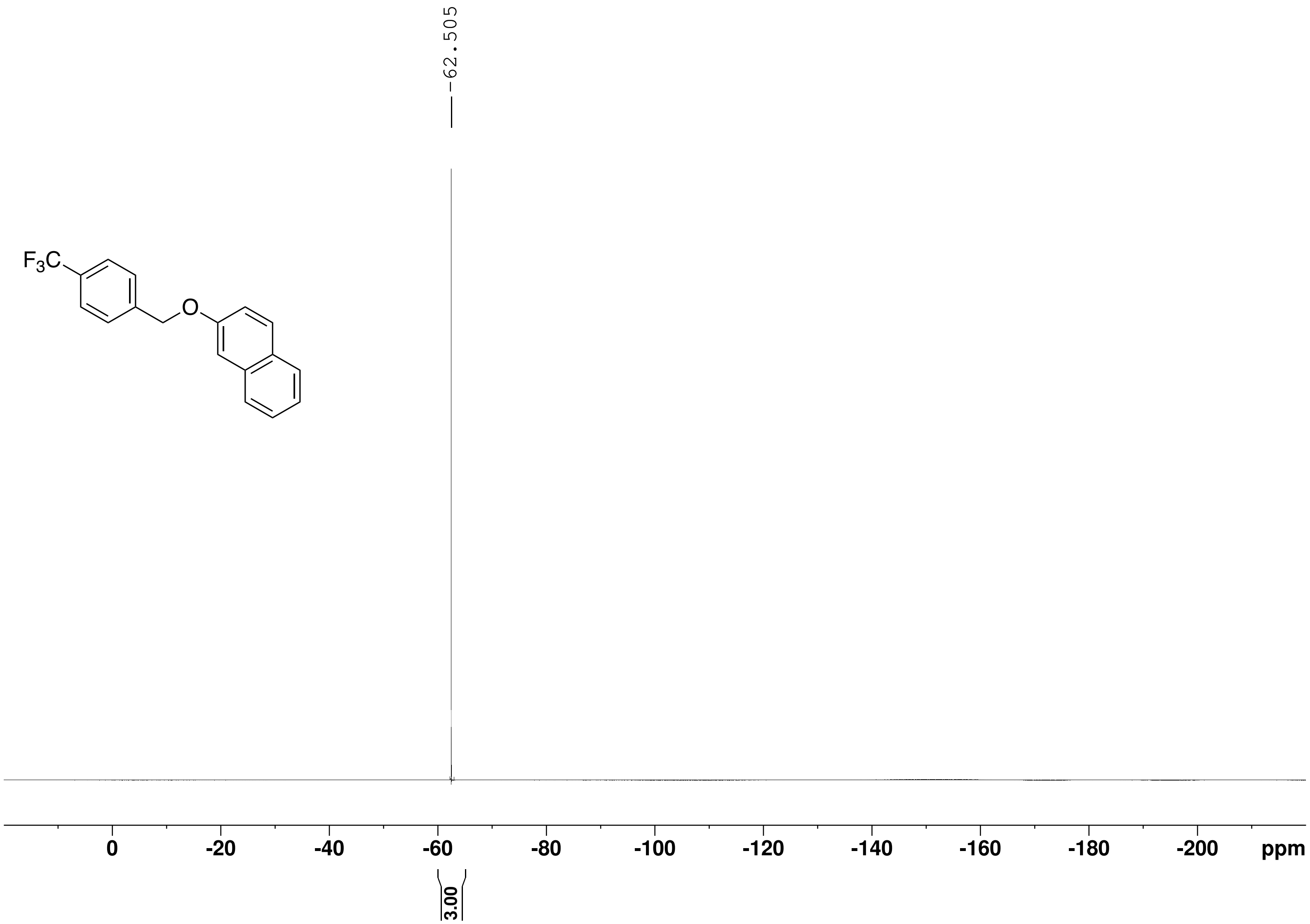

^{1}H NMR of **1f** (400 MHz, $CDCl_3$)

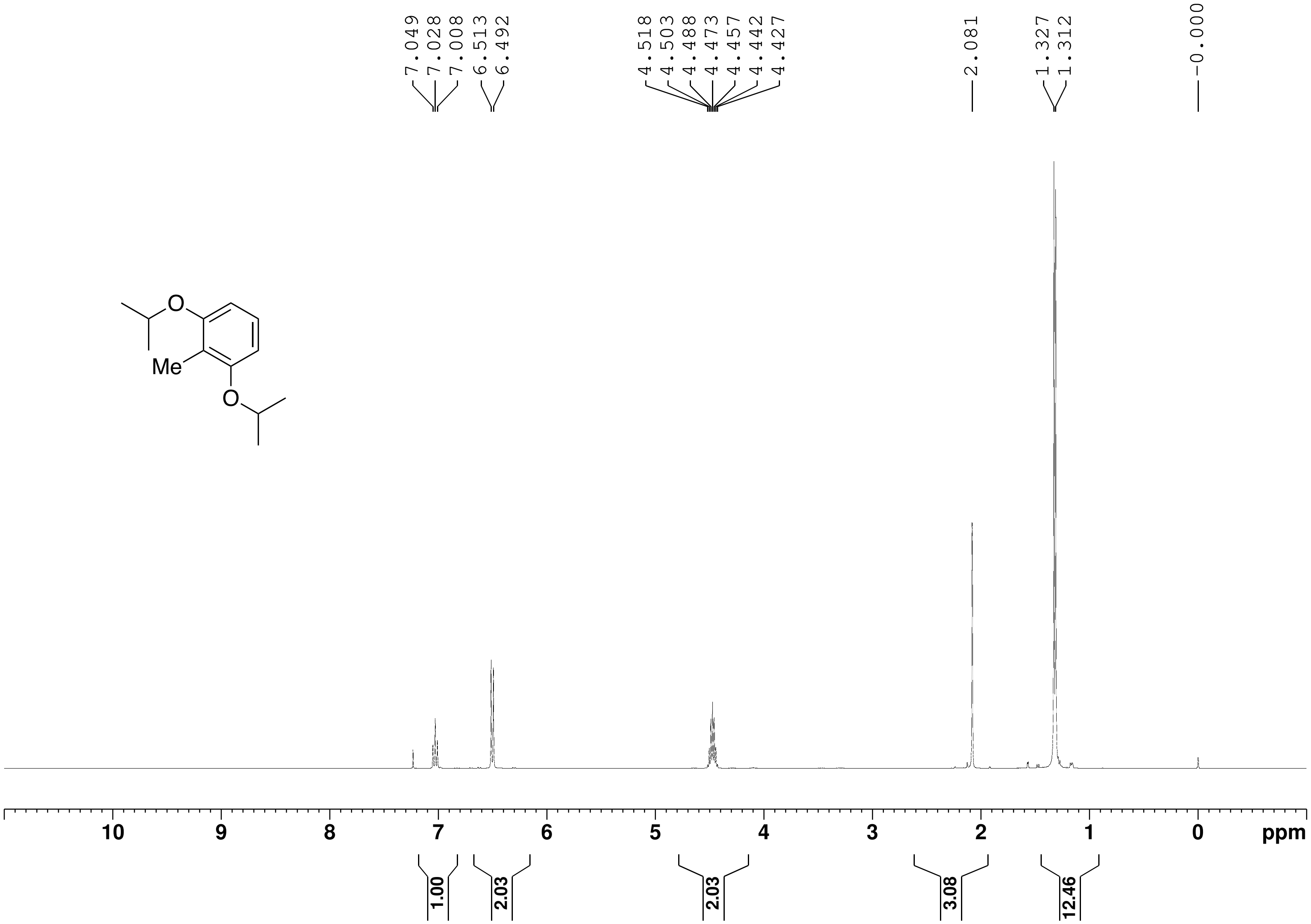

^{13}C NMR of **1f** (100.6 MHz, $CDCl_3$)

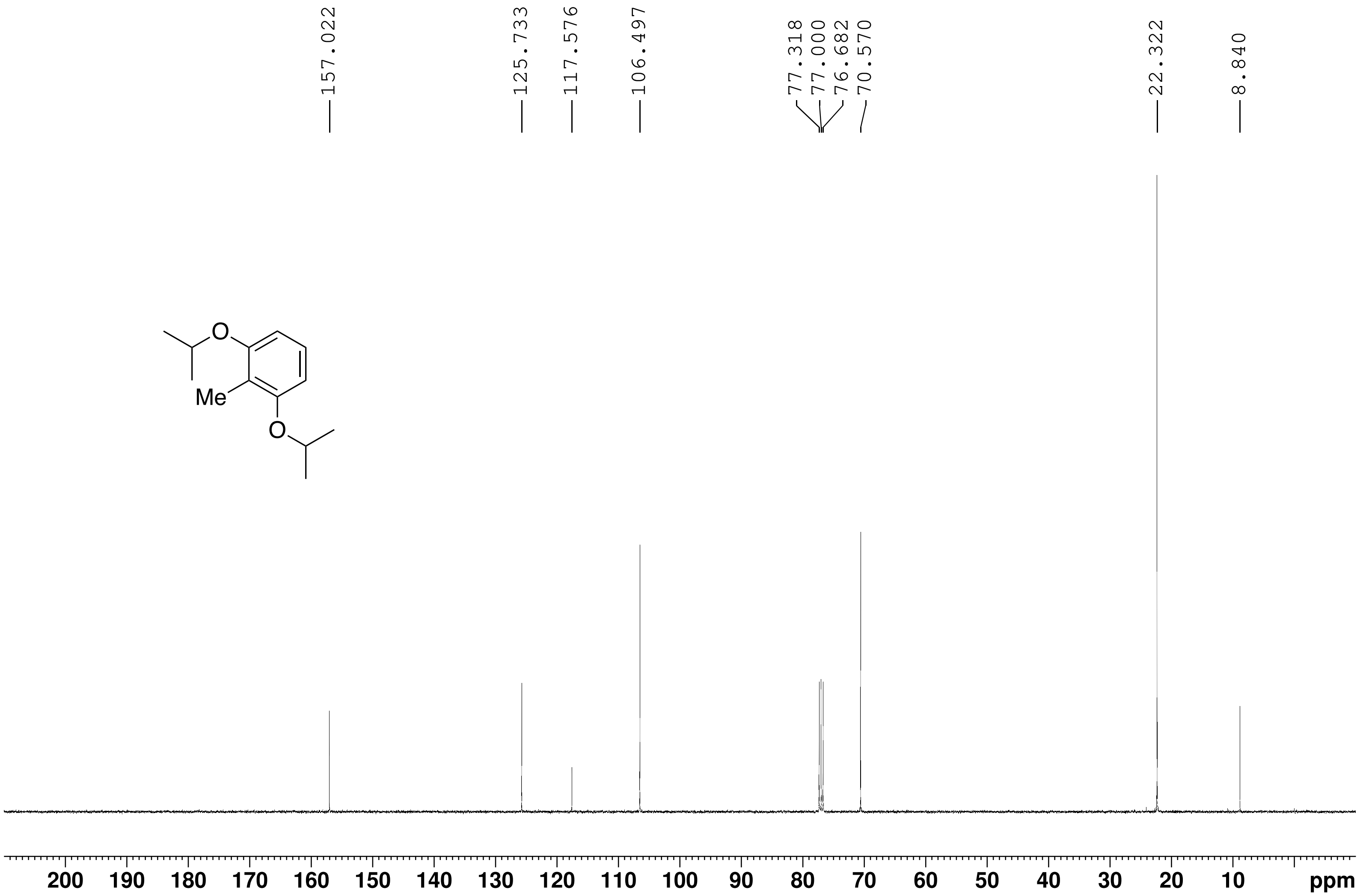

^{1}H NMR of **3,3,3-Trifluoropropyl 1-Methyl-1*H*-imidazole-2-carboxylate** (400 MHz, $CDCl_3$)

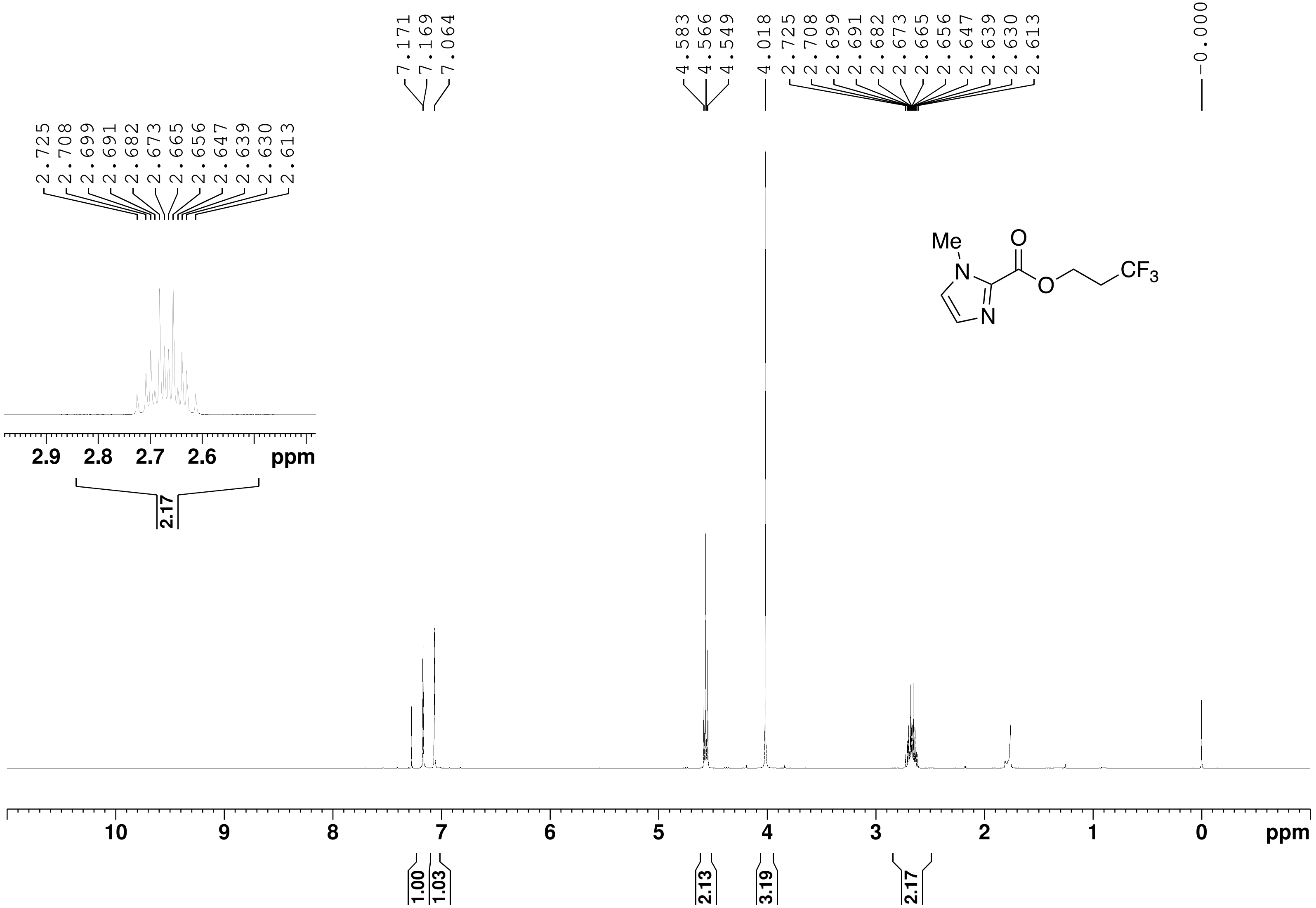

^{13}C NMR of **3,3,3-Trifluoropropyl 1-Methyl-1H-imidazole-2-carboxylate** (100.6 MHz, $CDCl_3$)

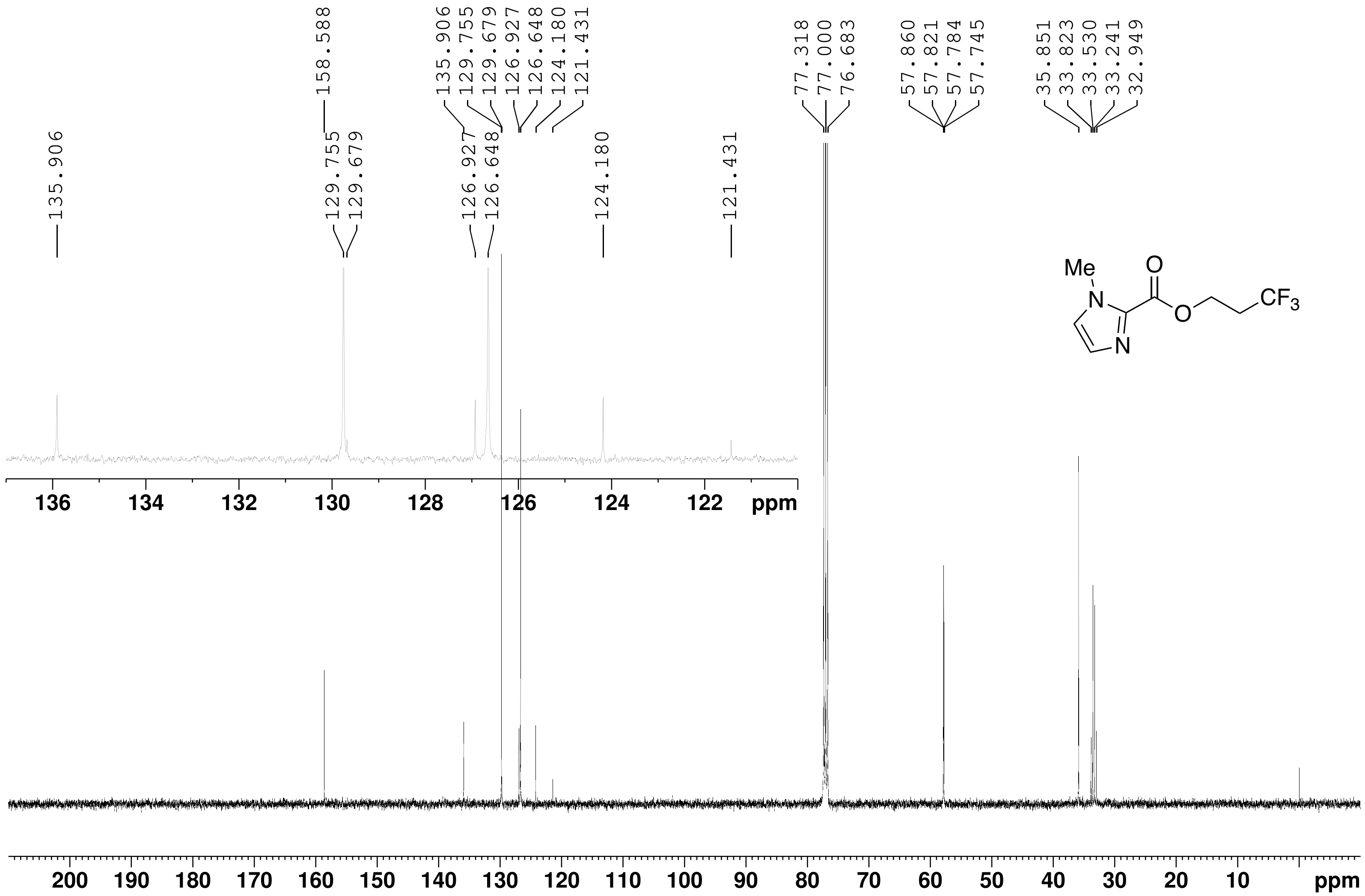

^{19}F NMR of **3,3,3-Trifluoropropyl 1-Methyl-1H-imidazole-2-carboxylate** (376.5 MHz, $CDCl_3$)

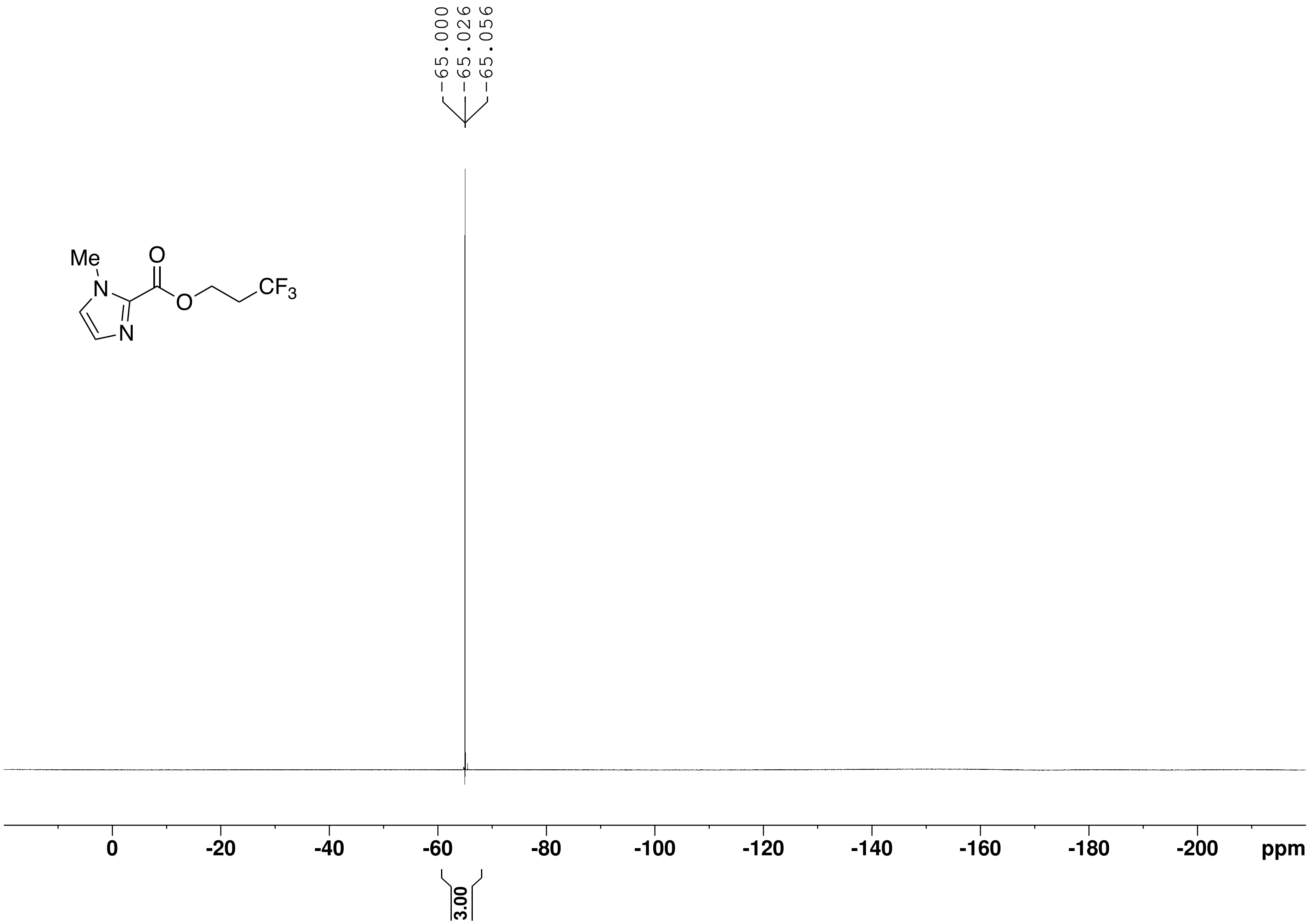

^{1}H NMR of **3-Buten-1-yl 1-Methyl-1*H*-imidazole-2-carboxylate** (400 MHz, $CDCl_3$)

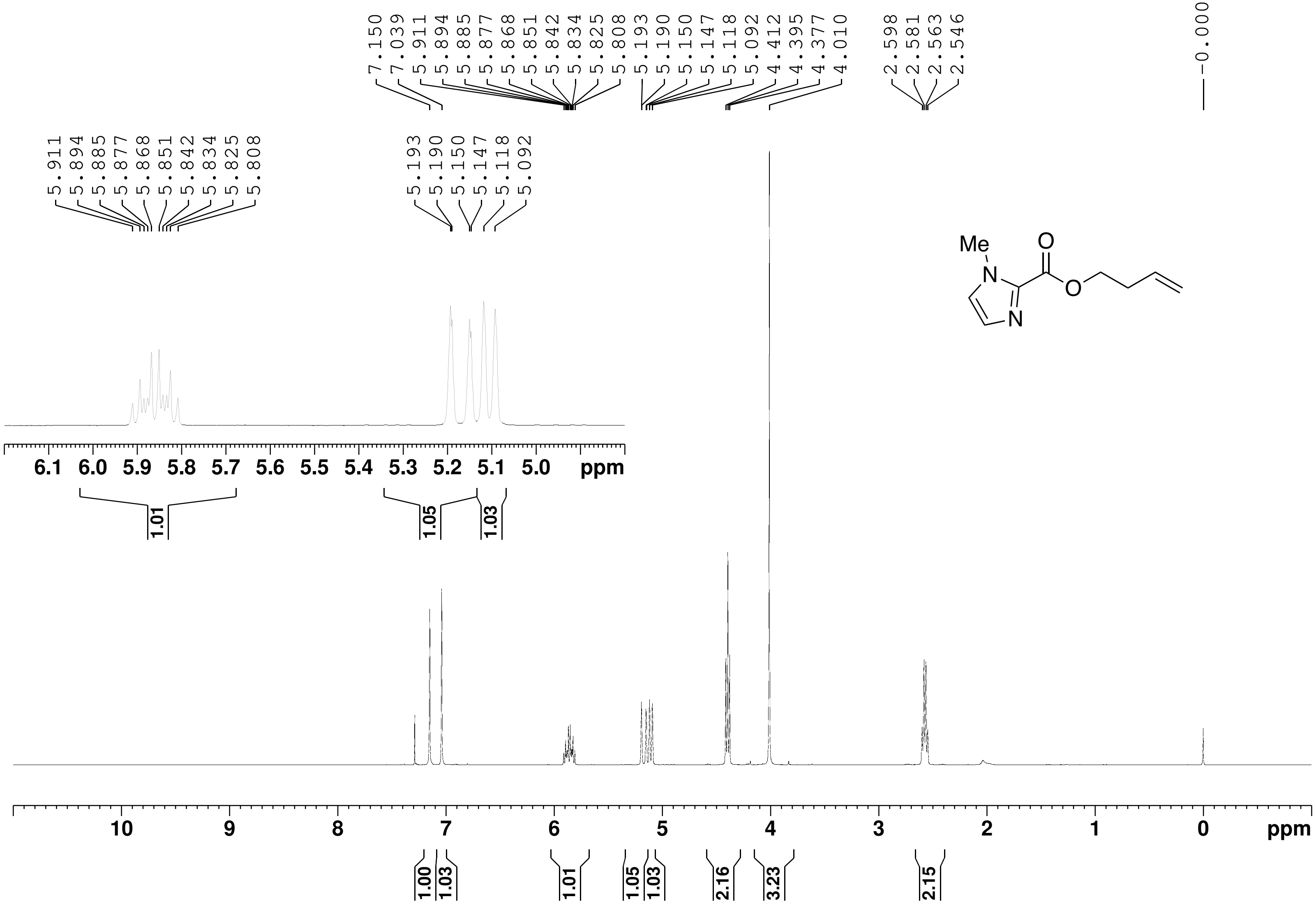

^{13}C NMR of **3-Buten-1-yl 1-Methyl-1H-imidazole-2-carboxylate** (100.6 MHz, $CDCl_3$)

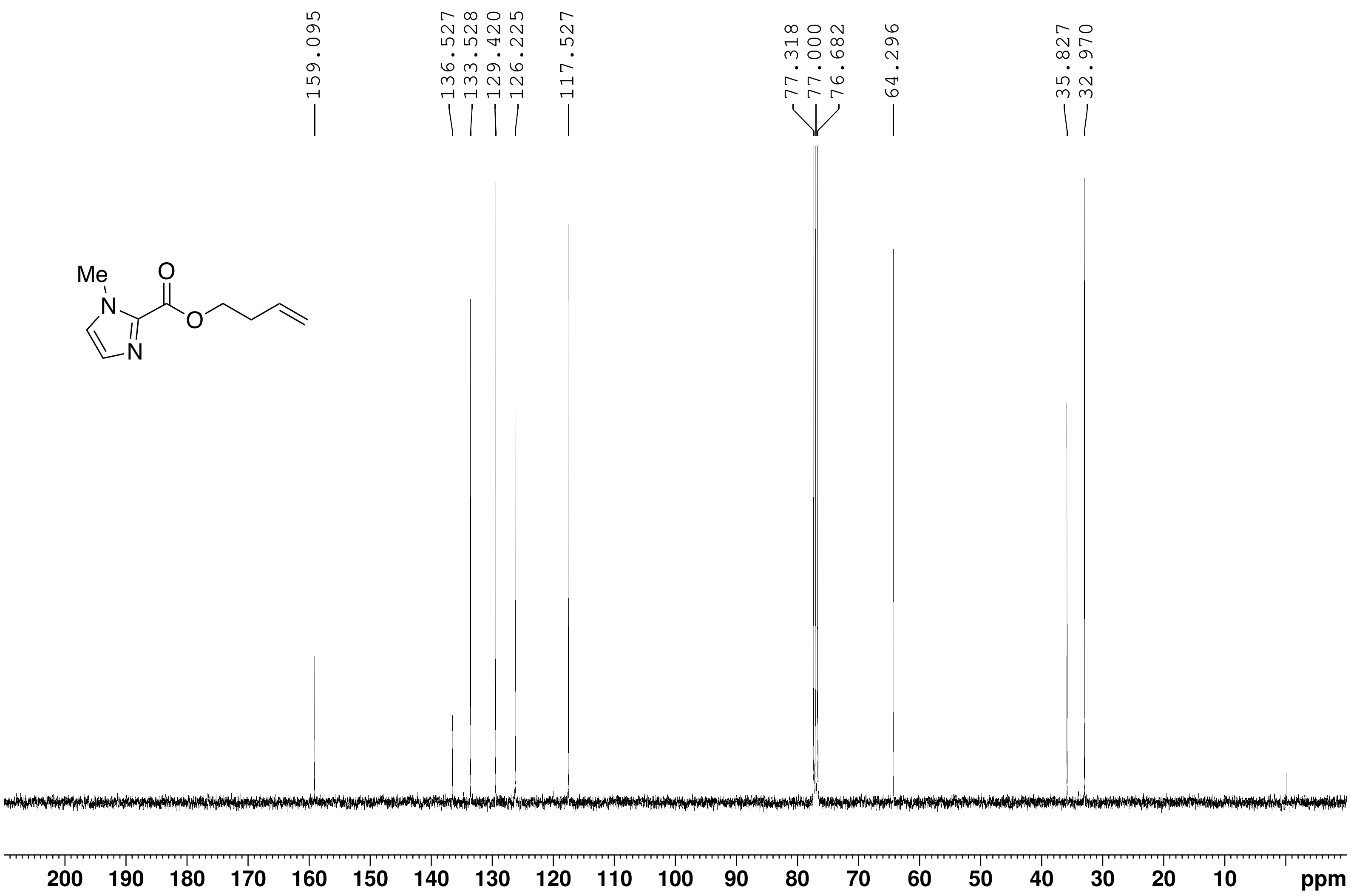

^{1}H NMR of **3-Hexyn-1-yl 1-Methyl-1*H*-imidazole-2-carboxylate** (400 MHz, $CDCl_3$)

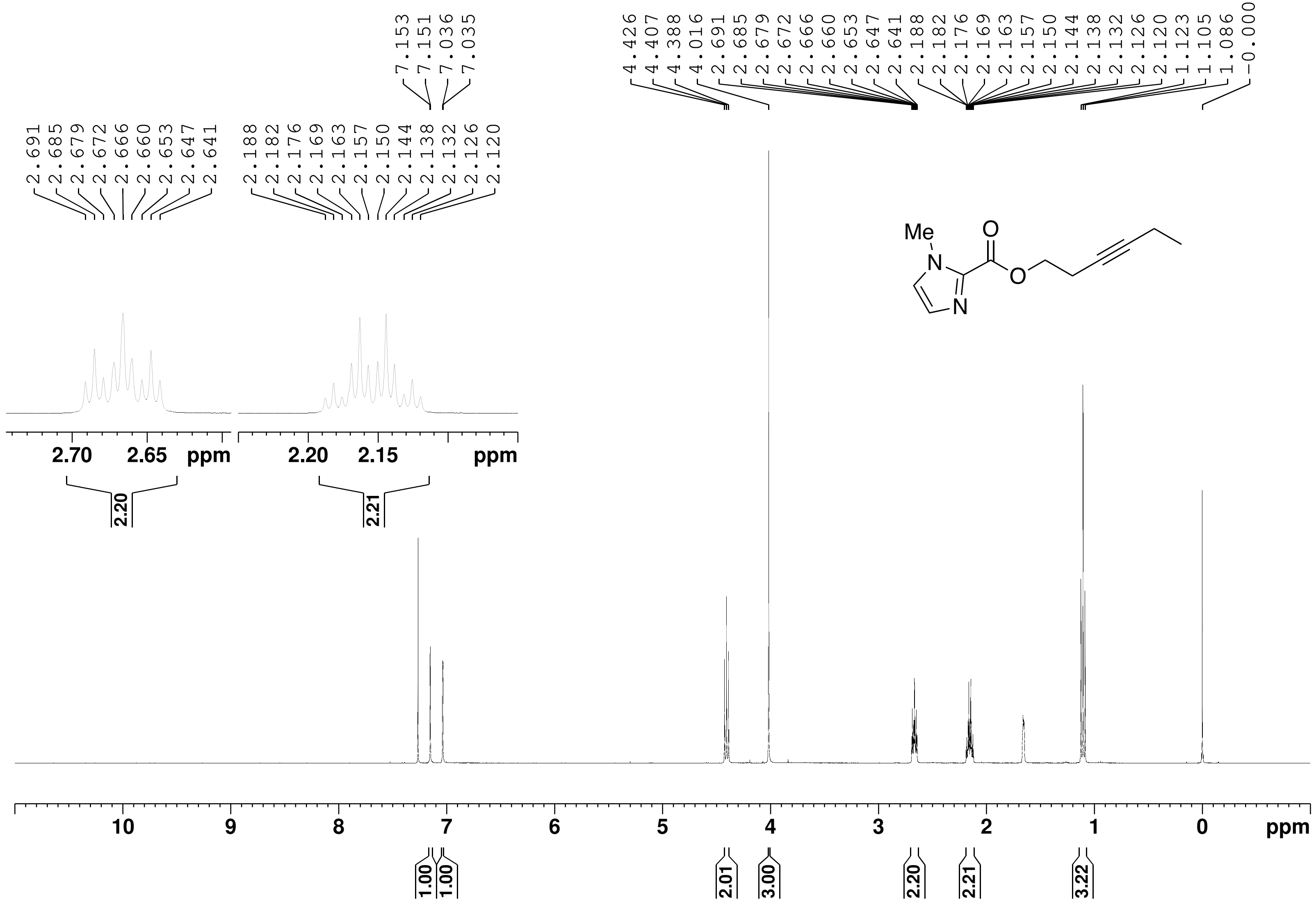

^{13}C NMR of **3-Hexyn-1-yl 1-Methyl-1*H*-imidazole-2-carboxylate** (100.6 MHz, $CDCl_3$)

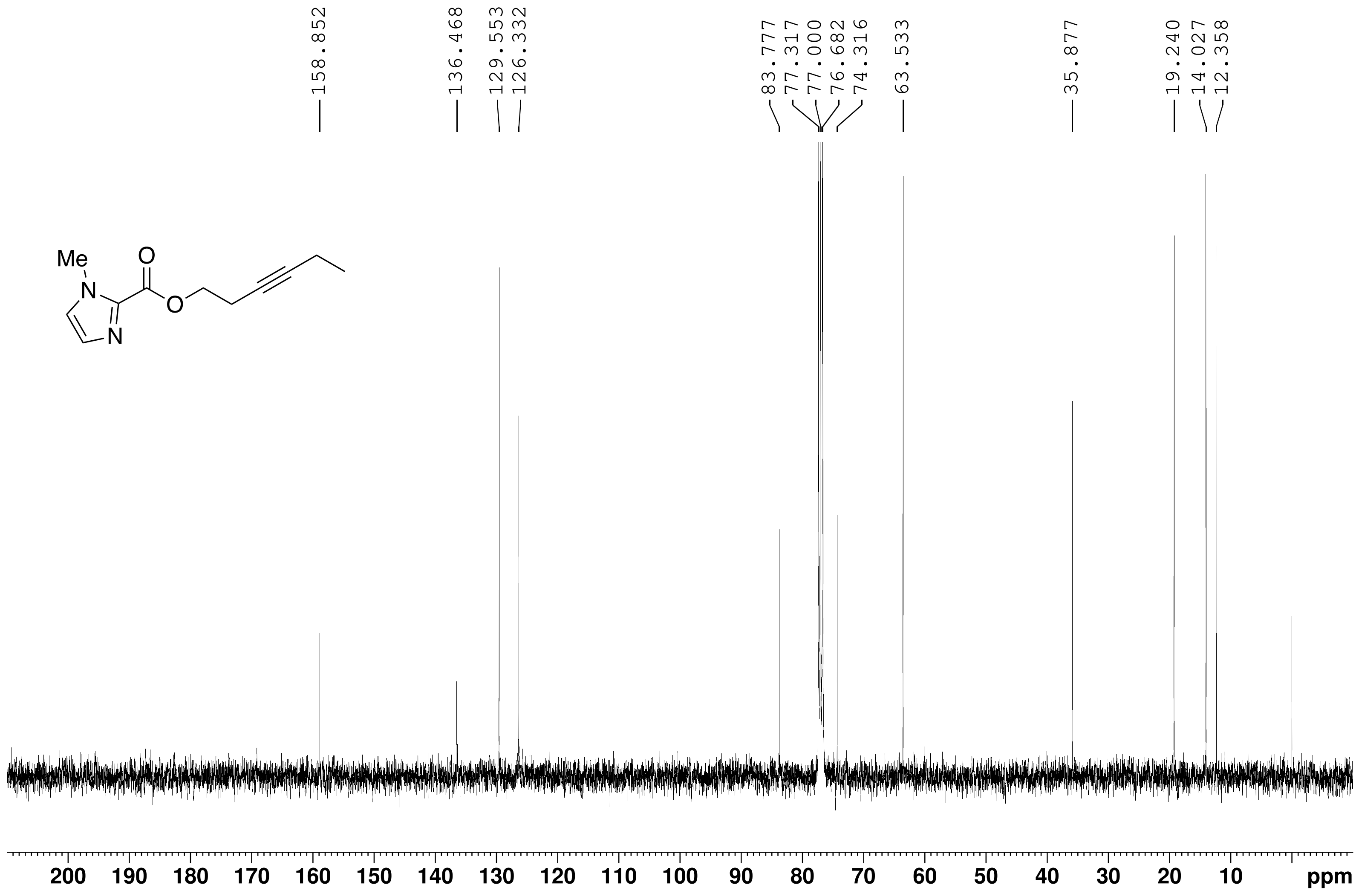

^{1}H NMR of **3,7-Dimethyloct-6-en-1-yl 1-Methyl-1*H*-imidazole-2-carboxylate** (400 MHz, $CDCl_3$)

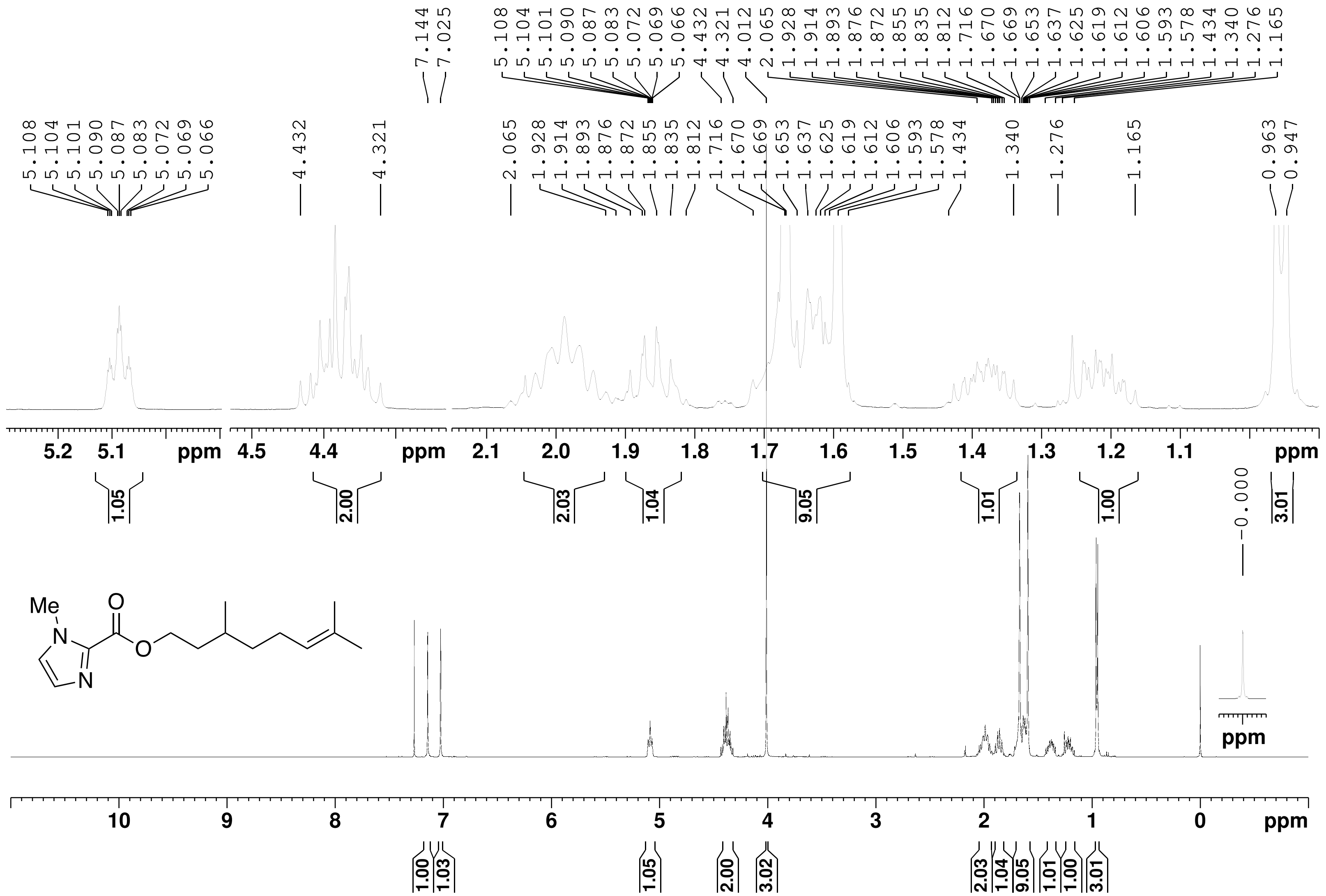

^{13}C NMR of **3,7-Dimethyloct-6-en-1-yl 1-Methyl-1H-imidazole-2-carboxylate** (100.6 MHz, $CDCl_3$)

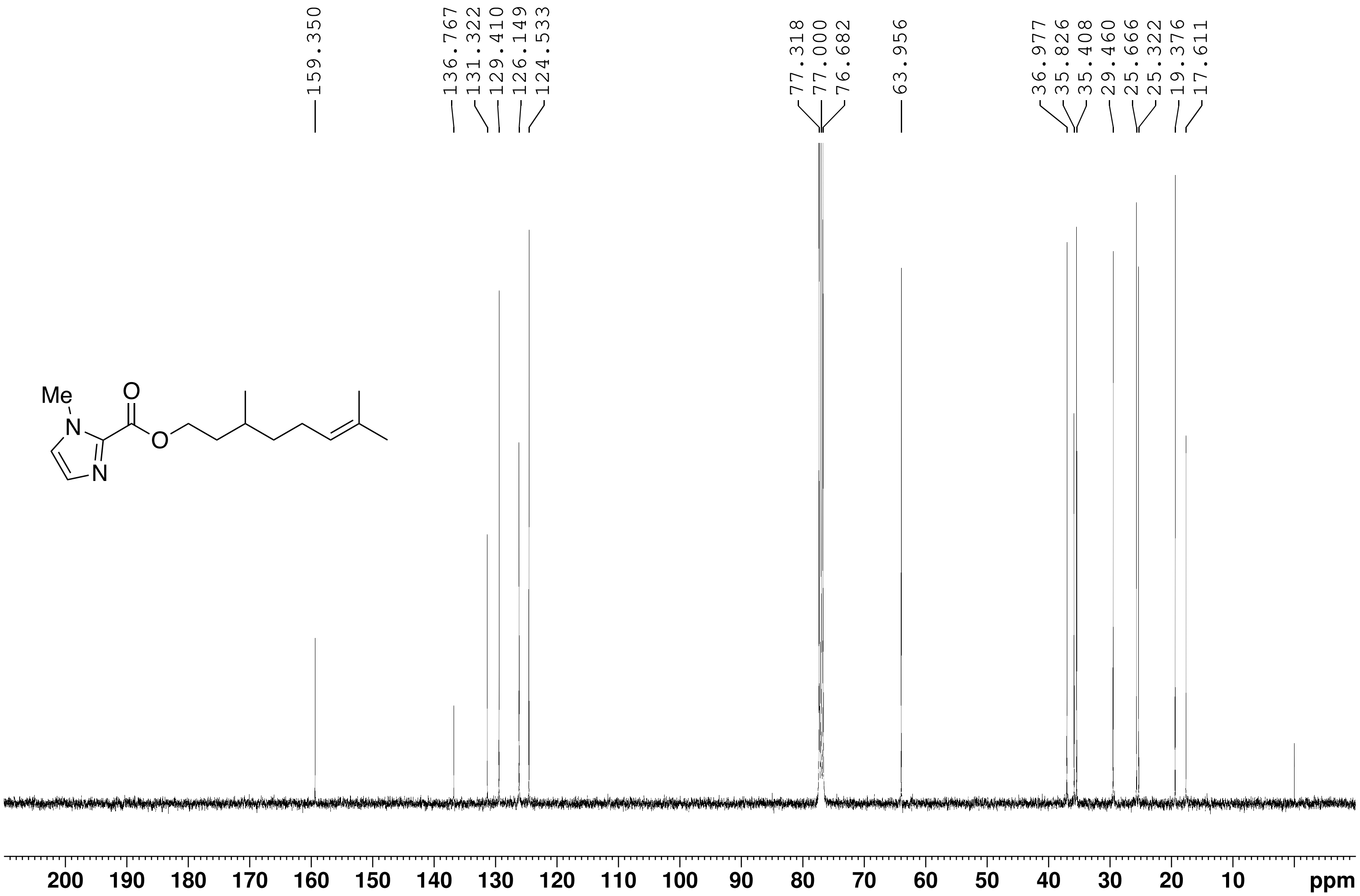

^{1}H NMR of **3-Phenylpropyl 1-Methyl-1*H*-imidazole-2-carboxylate** (400 MHz, $CDCl_3$)

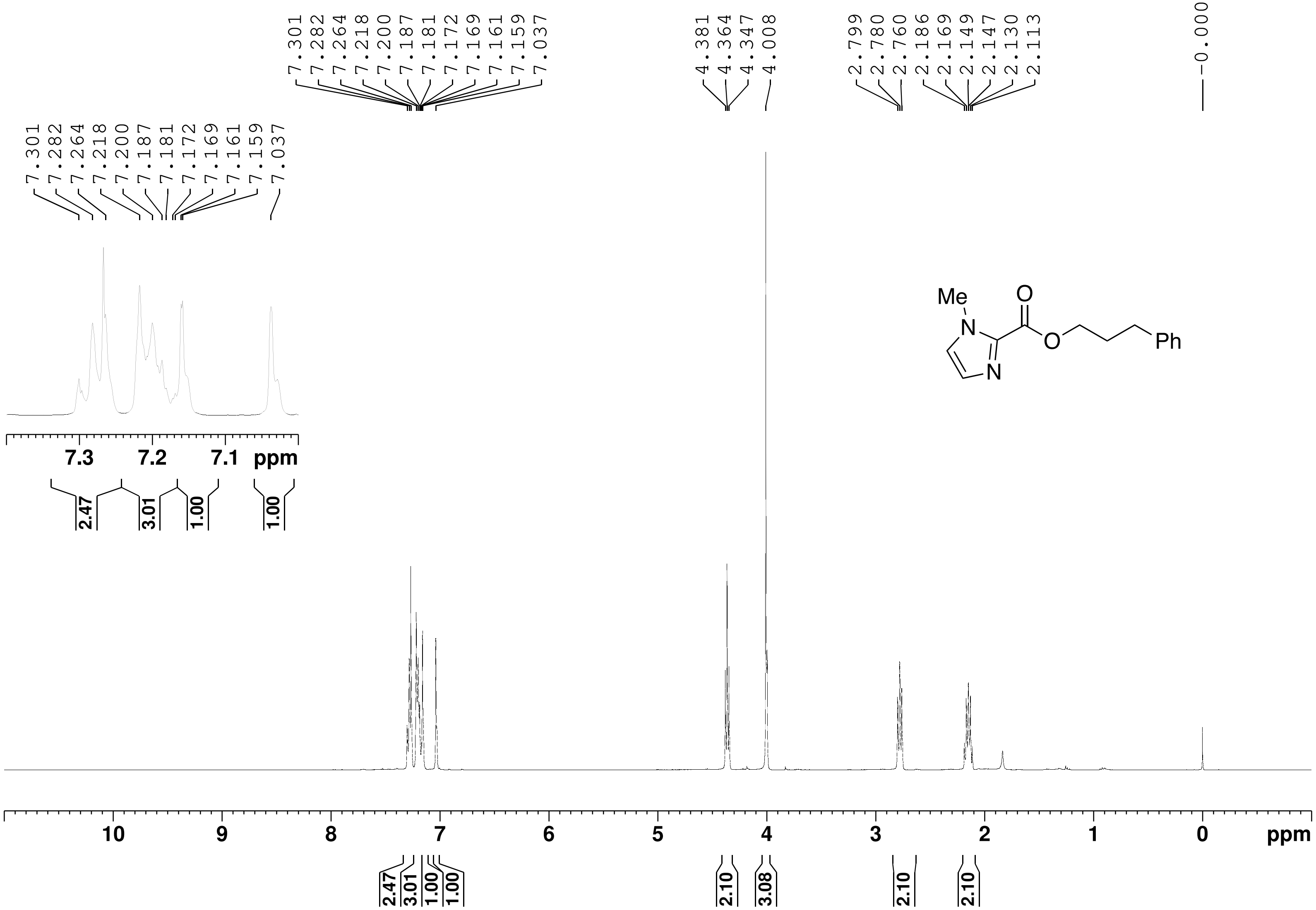

^{13}C NMR of **3-Phenylpropyl 1-Methyl-1*H*-imidazole-2-carboxylate** (100.6 MHz, $CDCl_3$)

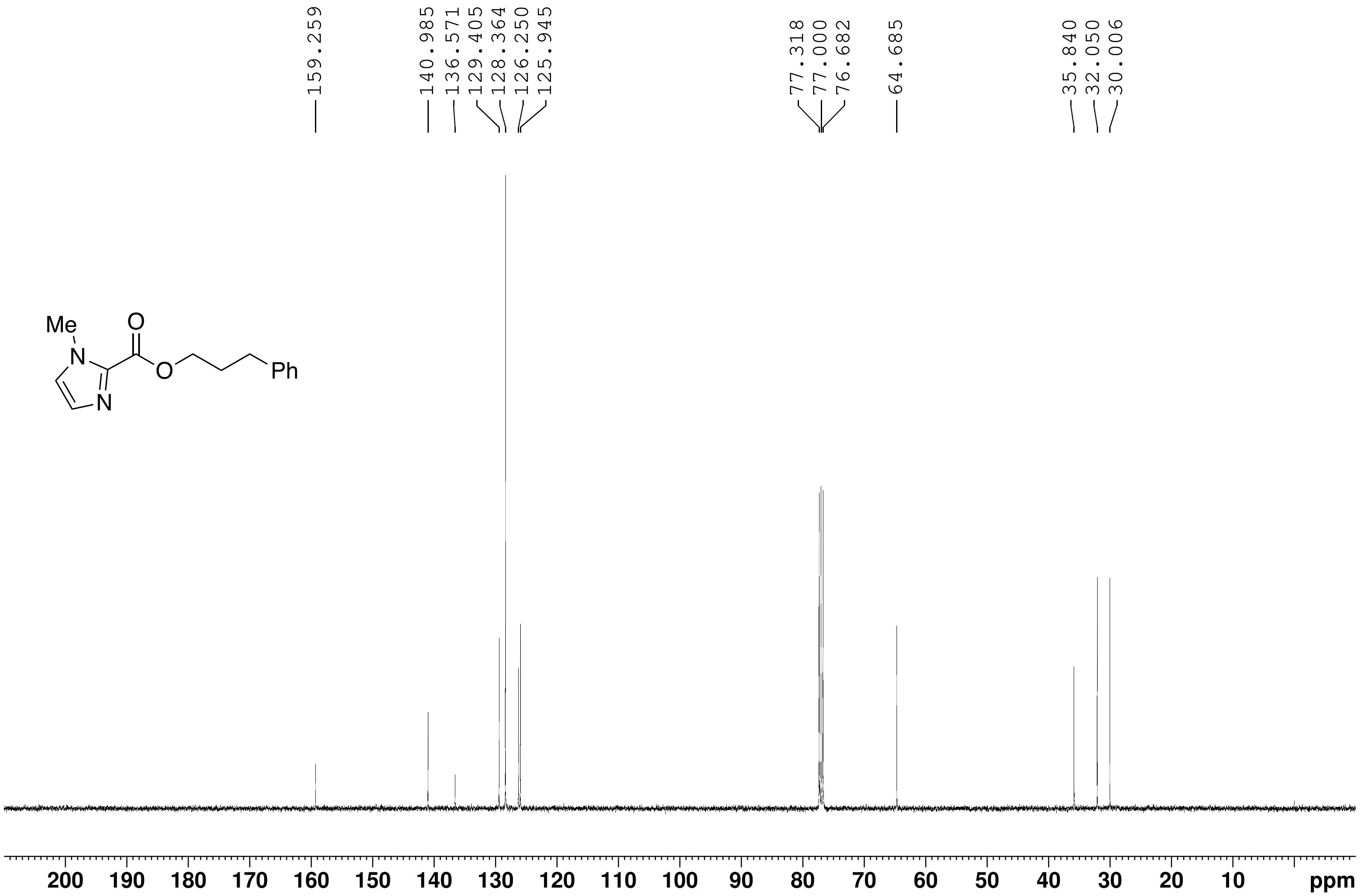

^{1}H NMR of **Cyclobutyl 1-Methyl-1*H*-imidazole-2-carboxylate** (400 MHz, $CDCl_3$)

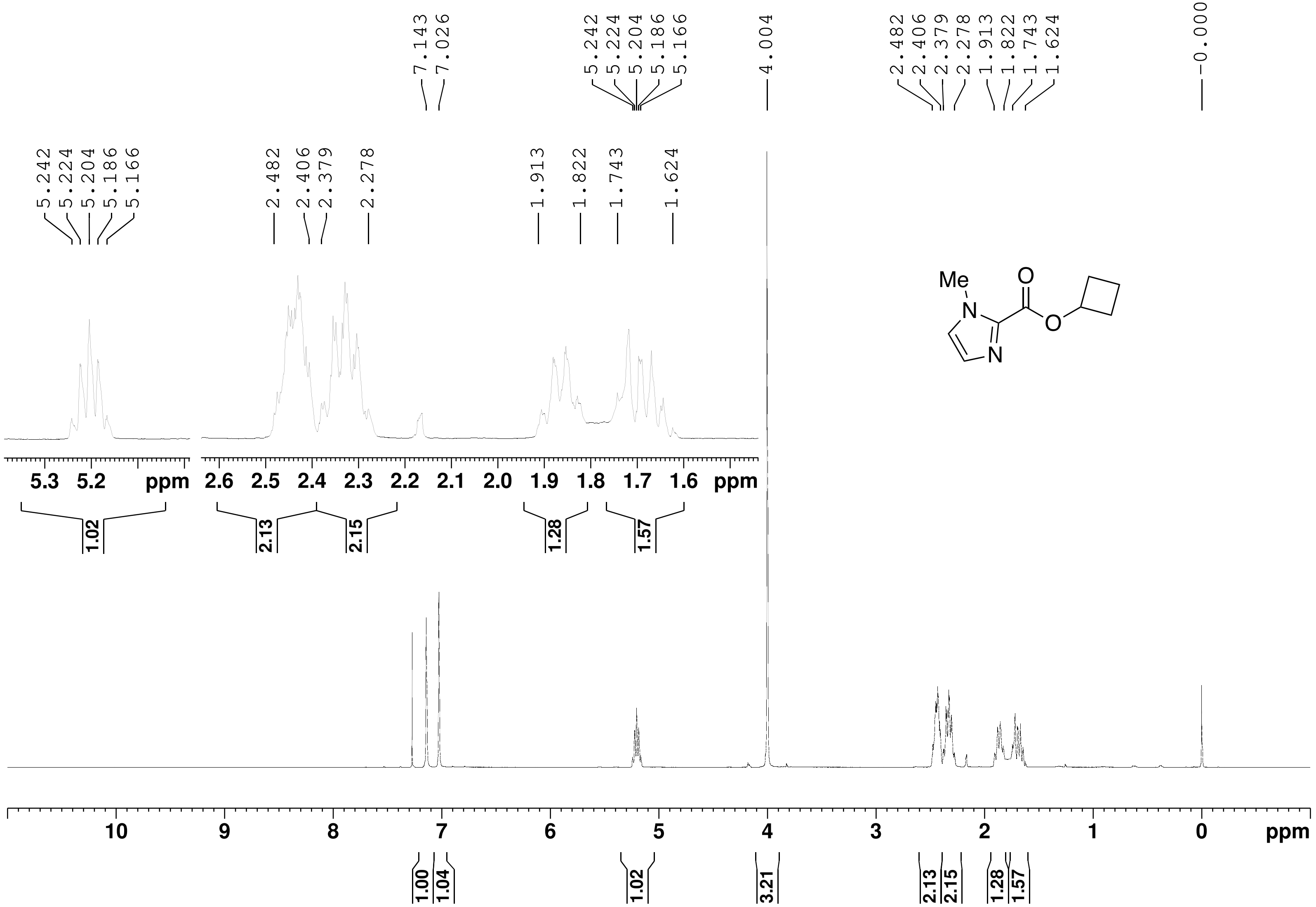

^{13}C NMR of **Cyclobutyl 1-Methyl-1H-imidazole-2-carboxylate** (100.6 MHz, $CDCl_3$)

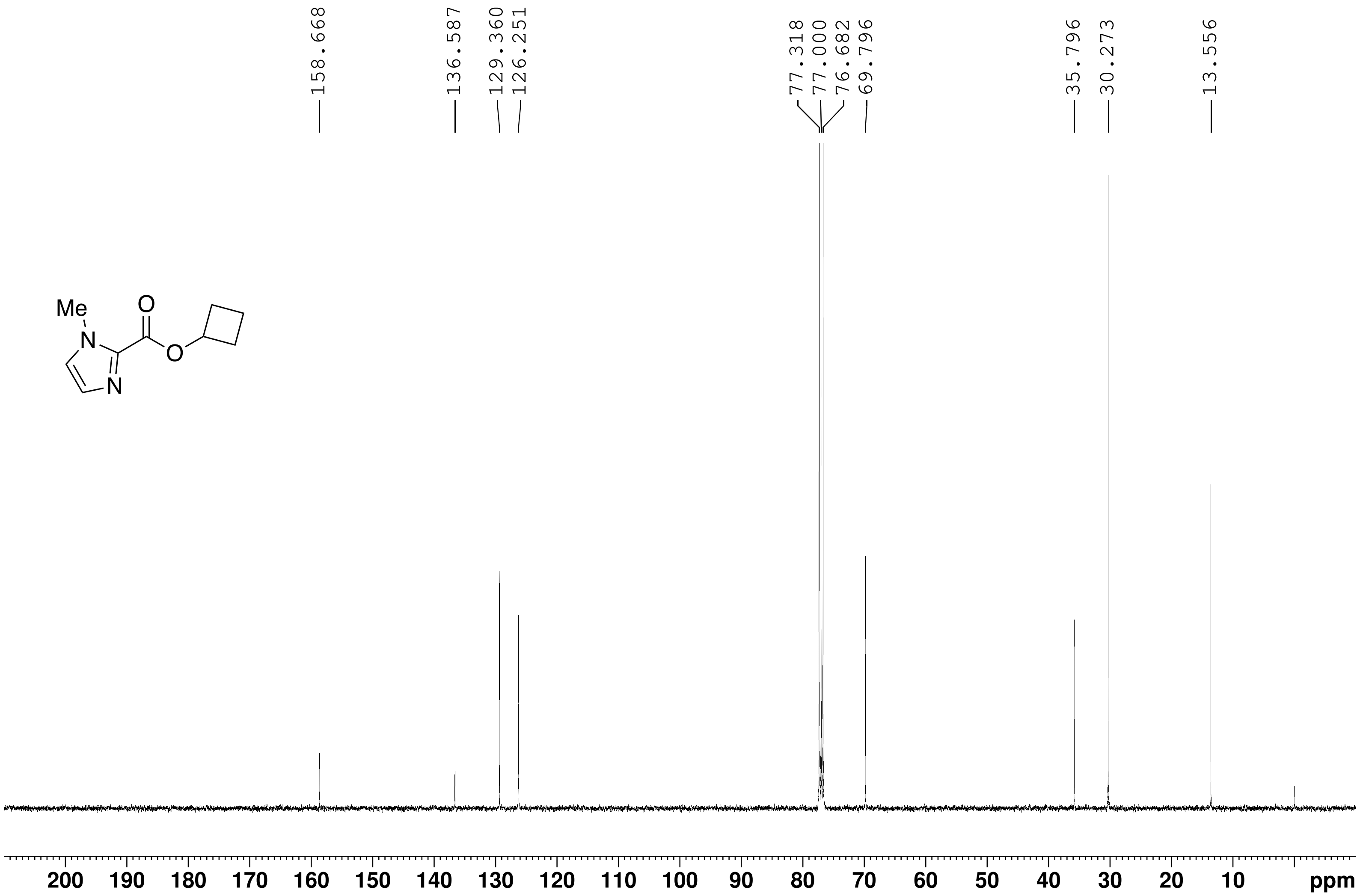

^{1}H NMR of **2b** (400 MHz, DMSO-d_6)

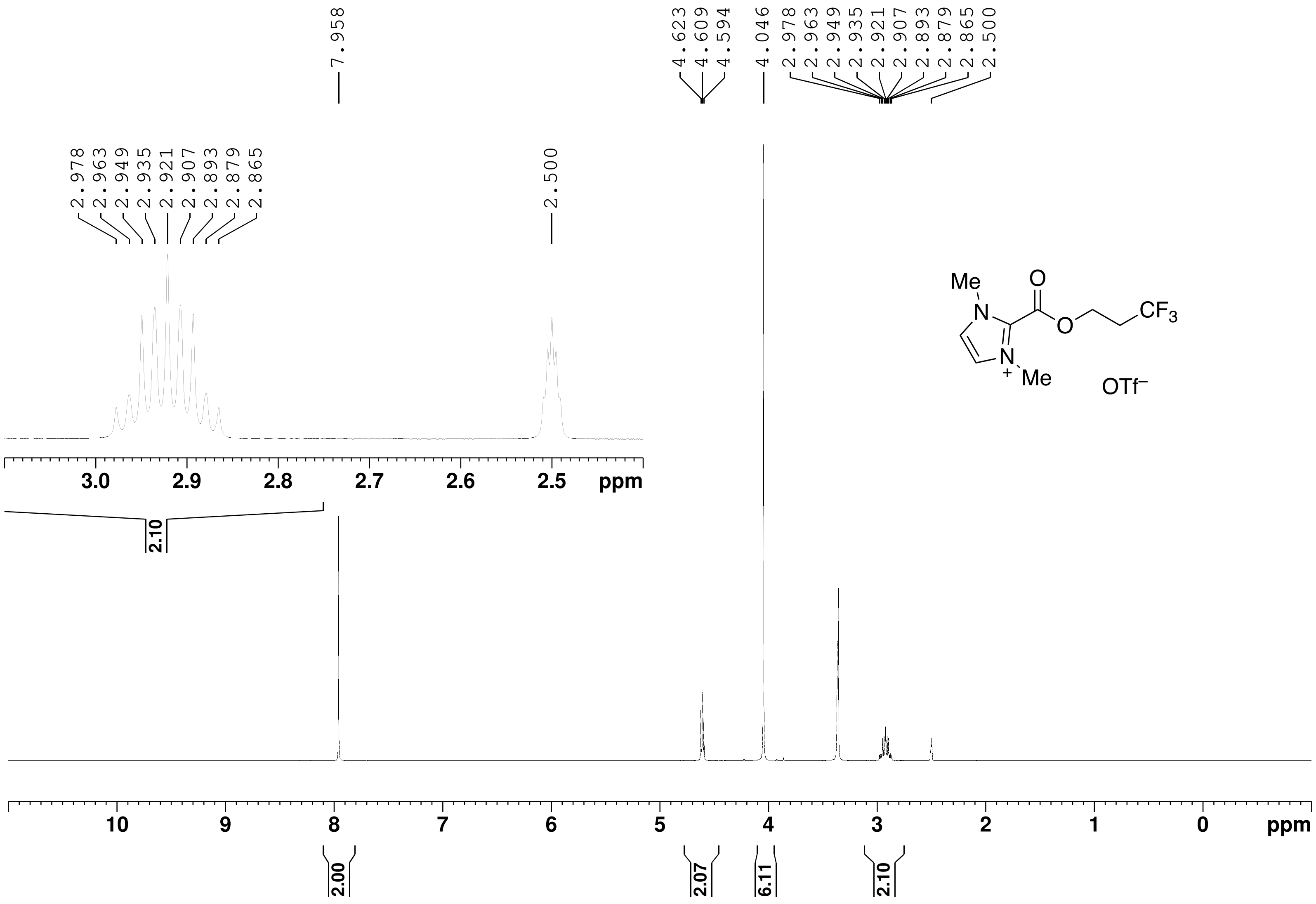

^{13}C NMR of **2b** (100.6 MHz, DMSO-d_6)

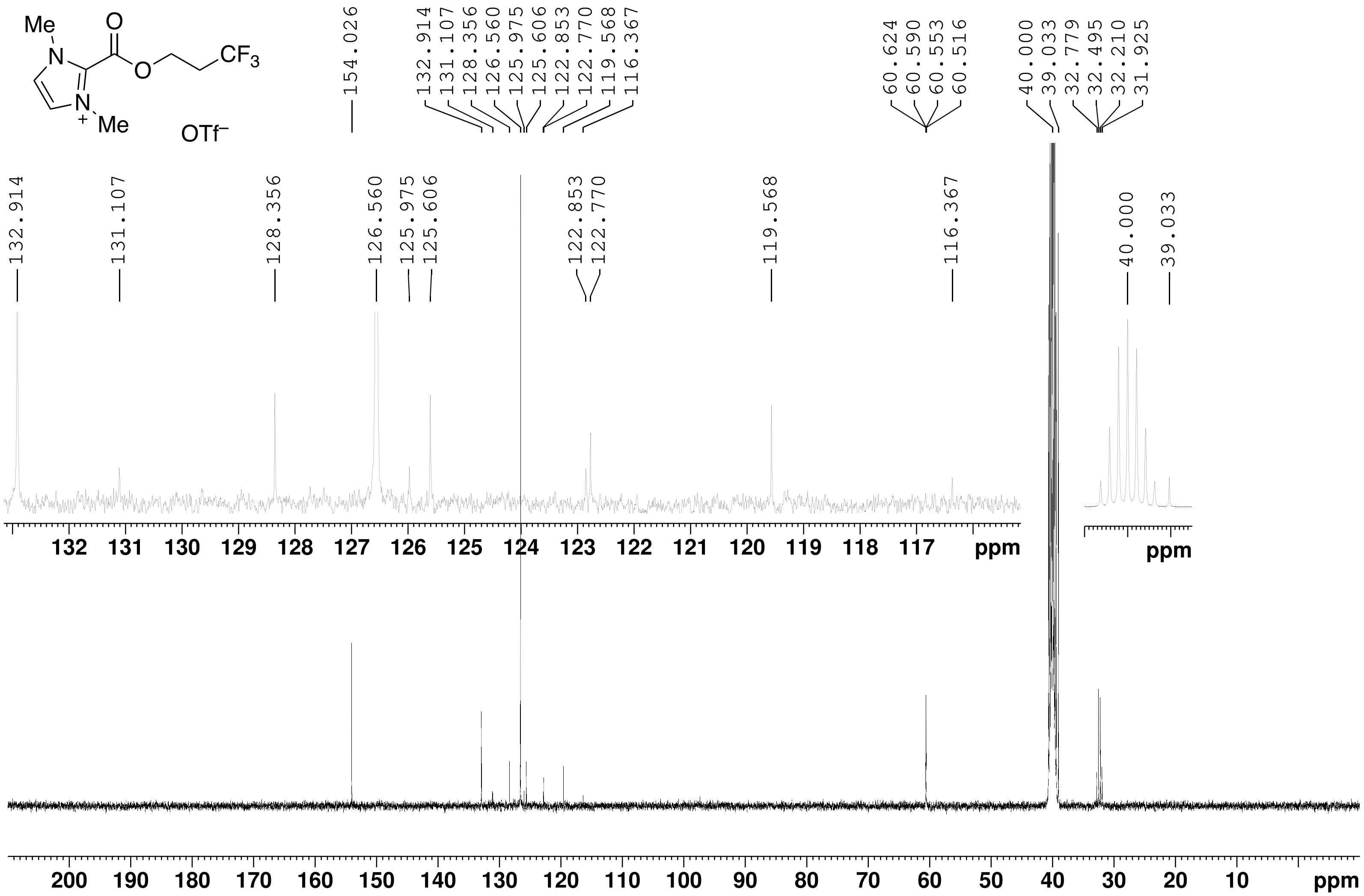

^{19}F NMR of **2b** (376.5 MHz, DMSO-d_6)

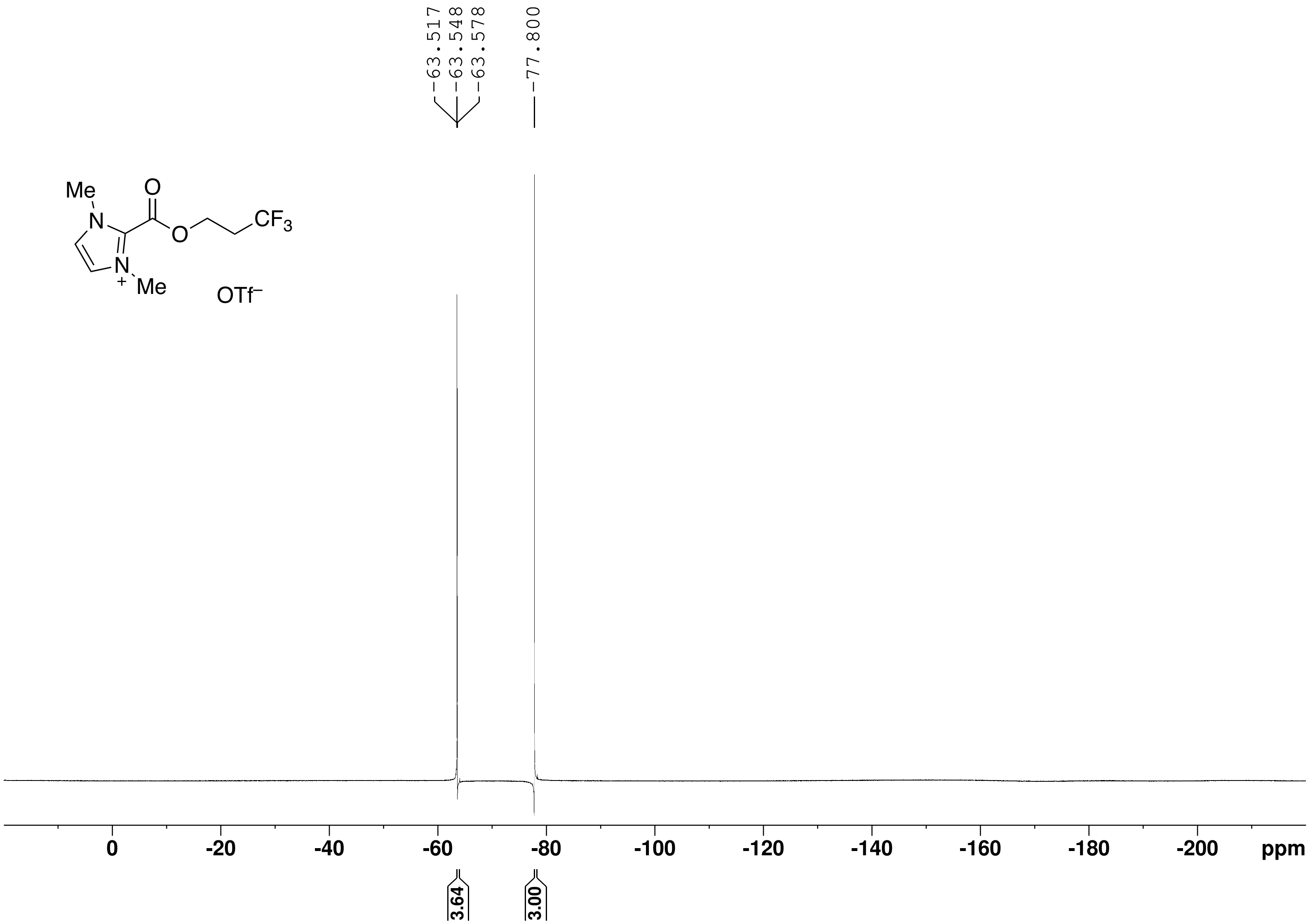

^{1}H NMR of **2c** (400 MHz, $CDCl_3$)

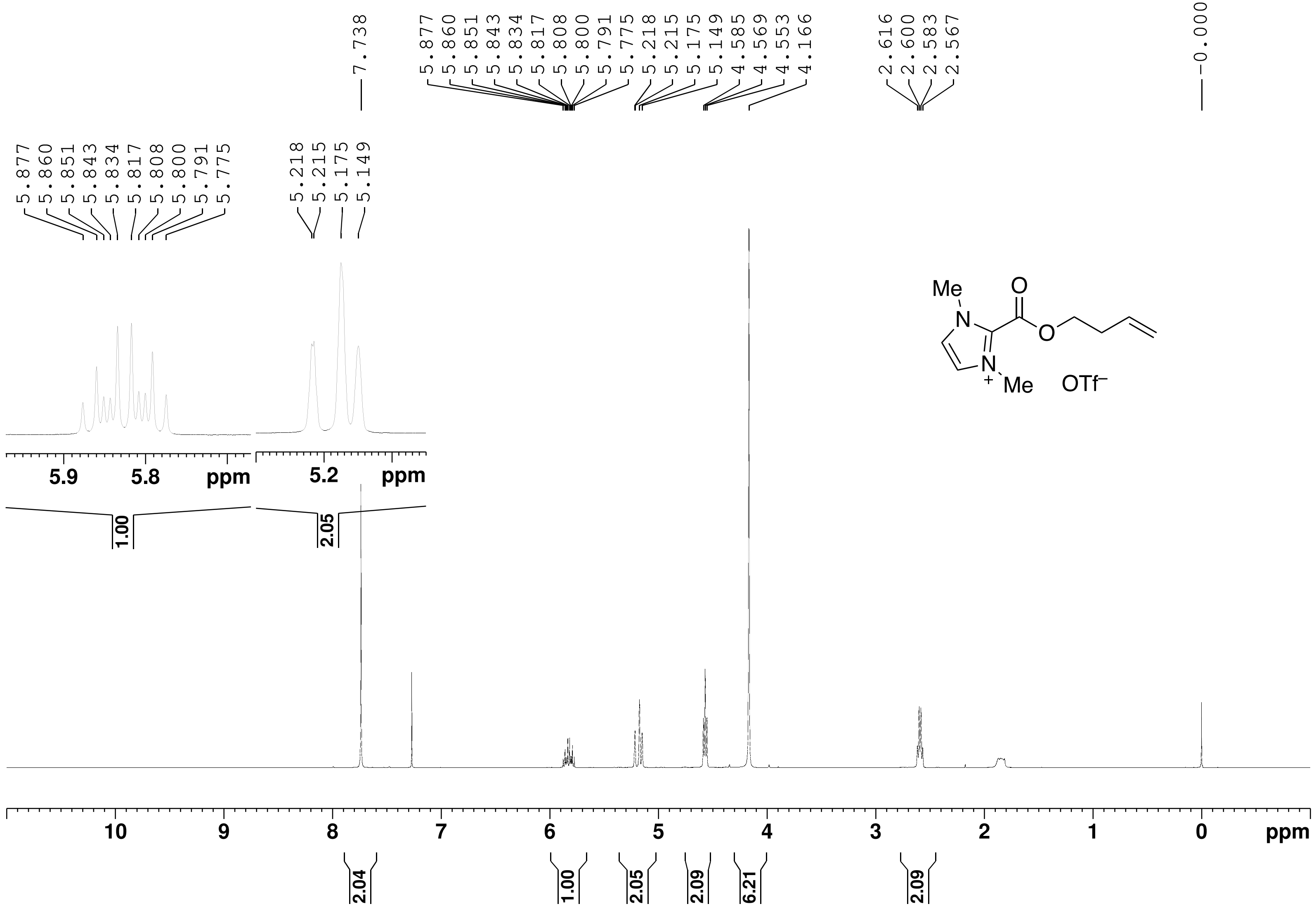

^{13}C NMR of **2c** (100.6 MHz, $CDCl_3$)

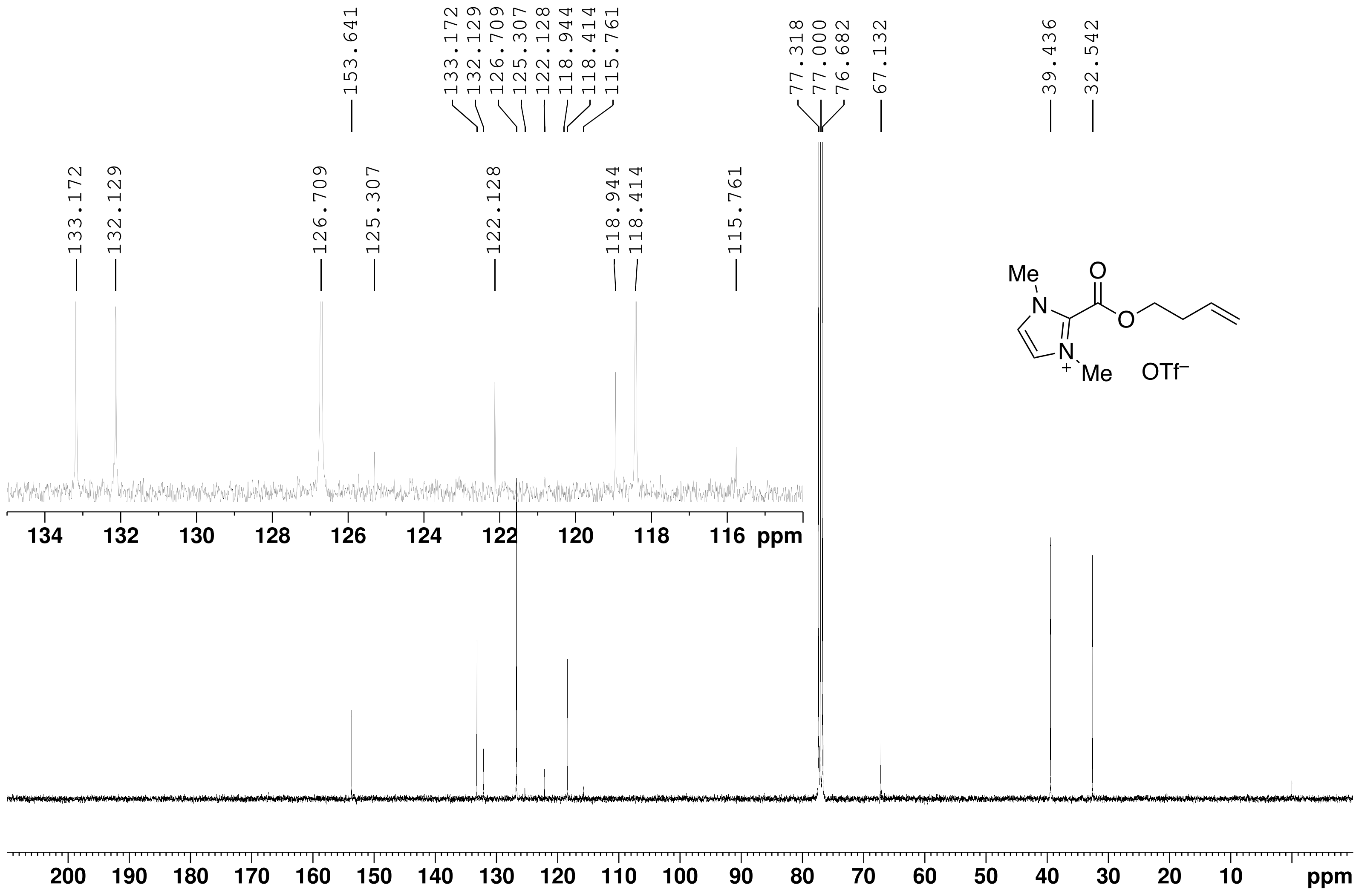

^{19}F NMR of **2c** (376.5 MHz, $CDCl_3$)

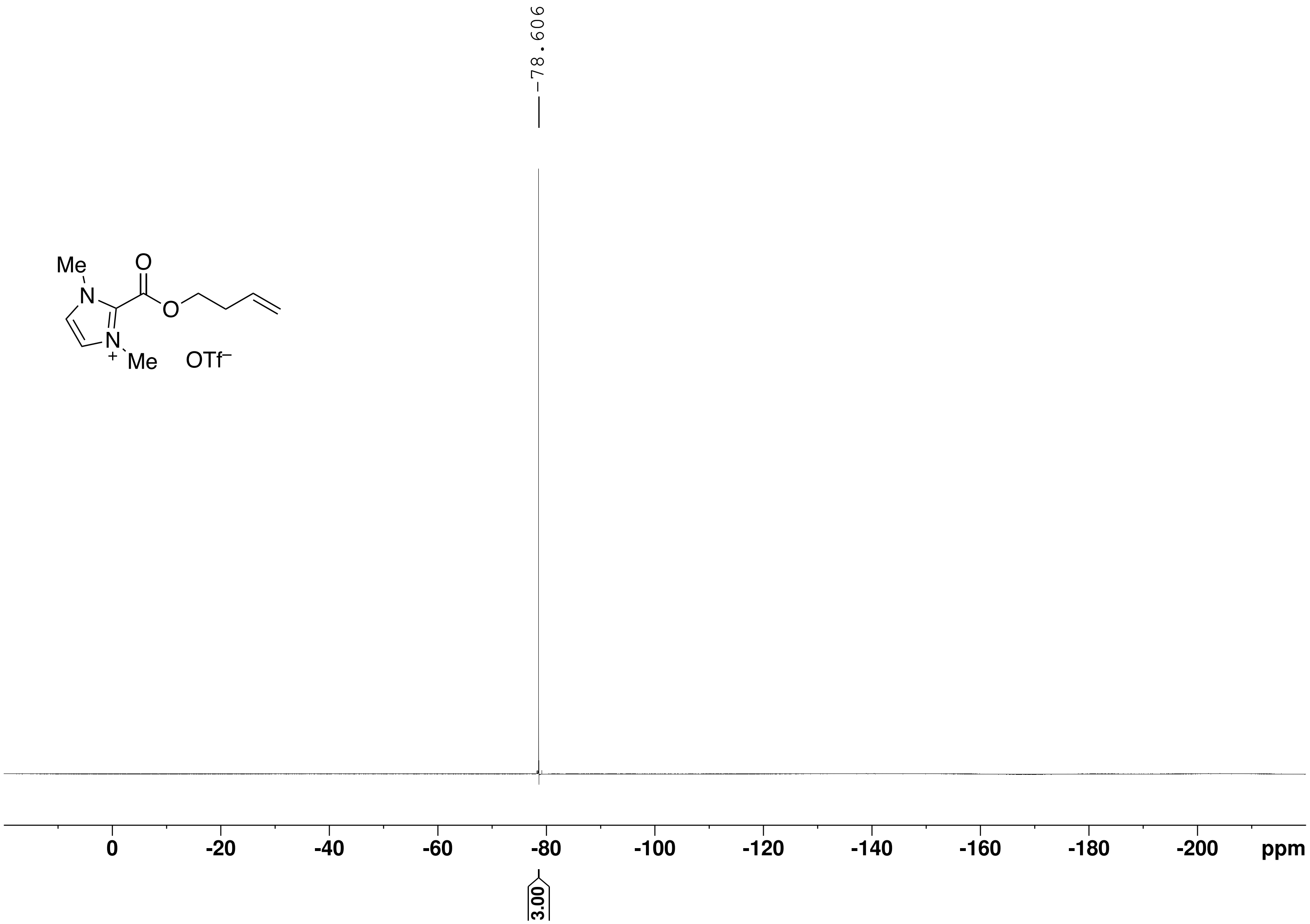

^{1}H NMR of **2d** (400 MHz, $CDCl_3$)

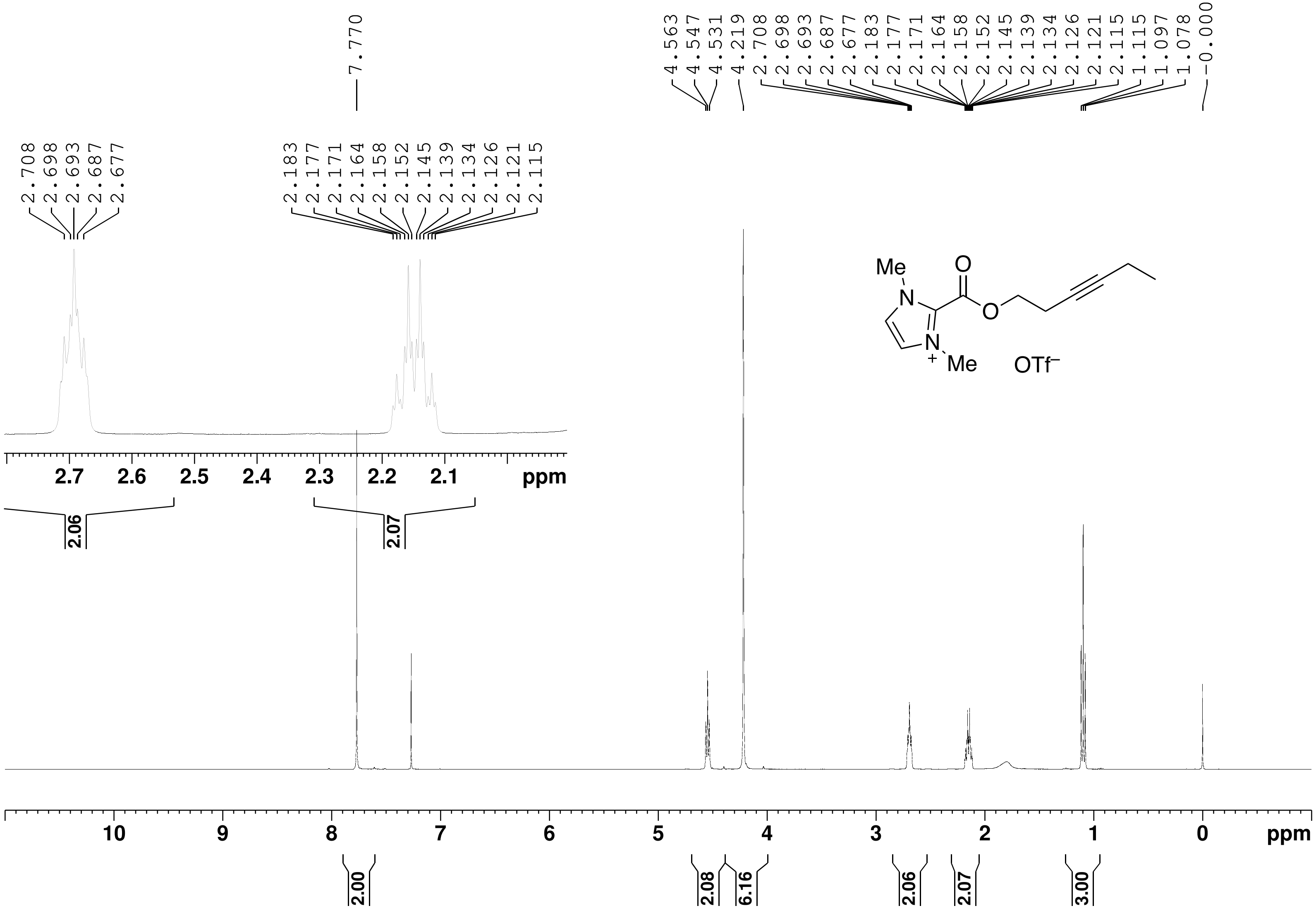

^{13}C NMR of **2d** (100.6 MHz, $CDCl_3$)

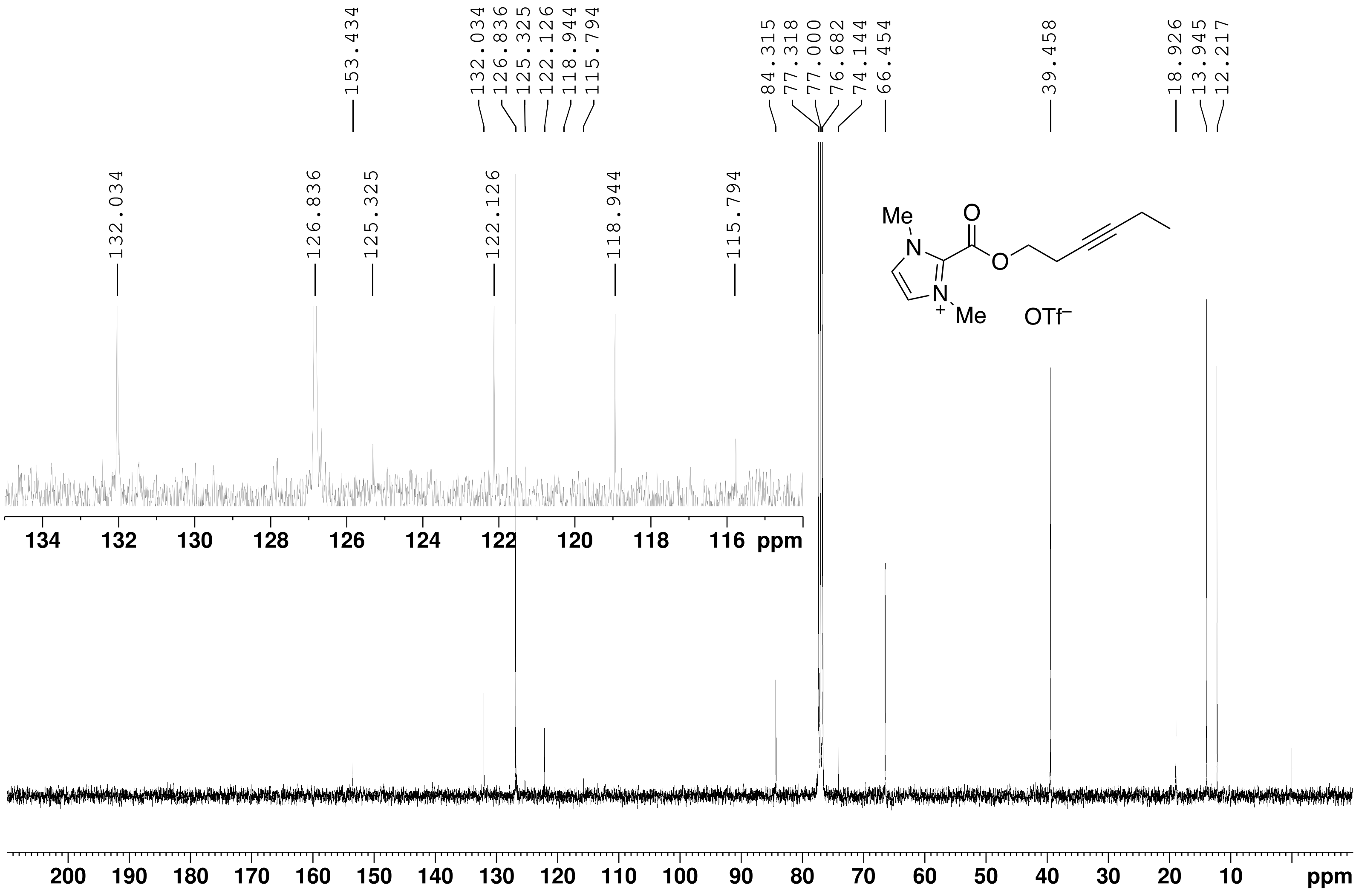

^{19}F NMR of **2d** (376.5 MHz, $CDCl_3$)

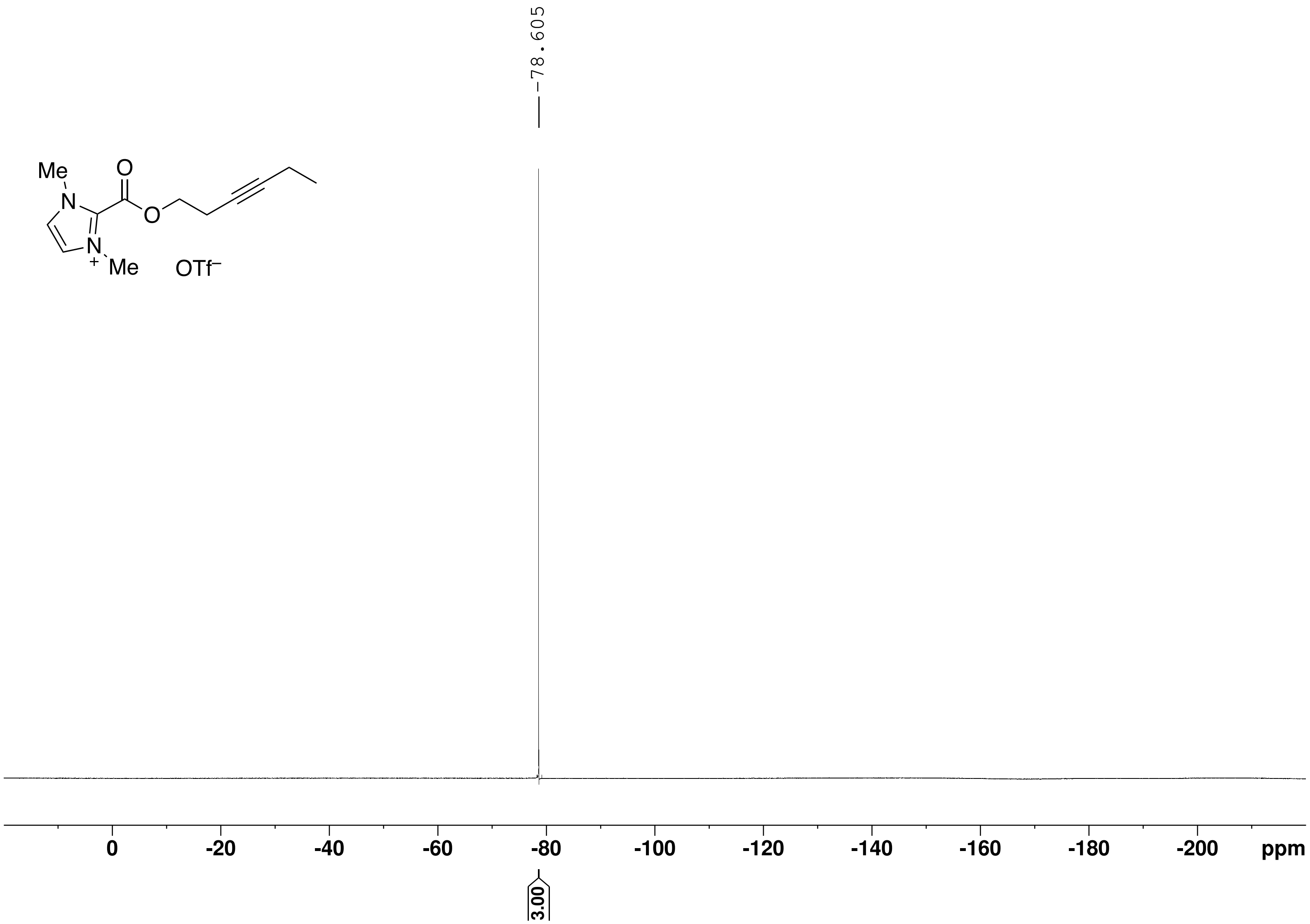

^{1}H NMR of **2e** (400 MHz, $CDCl_3$)

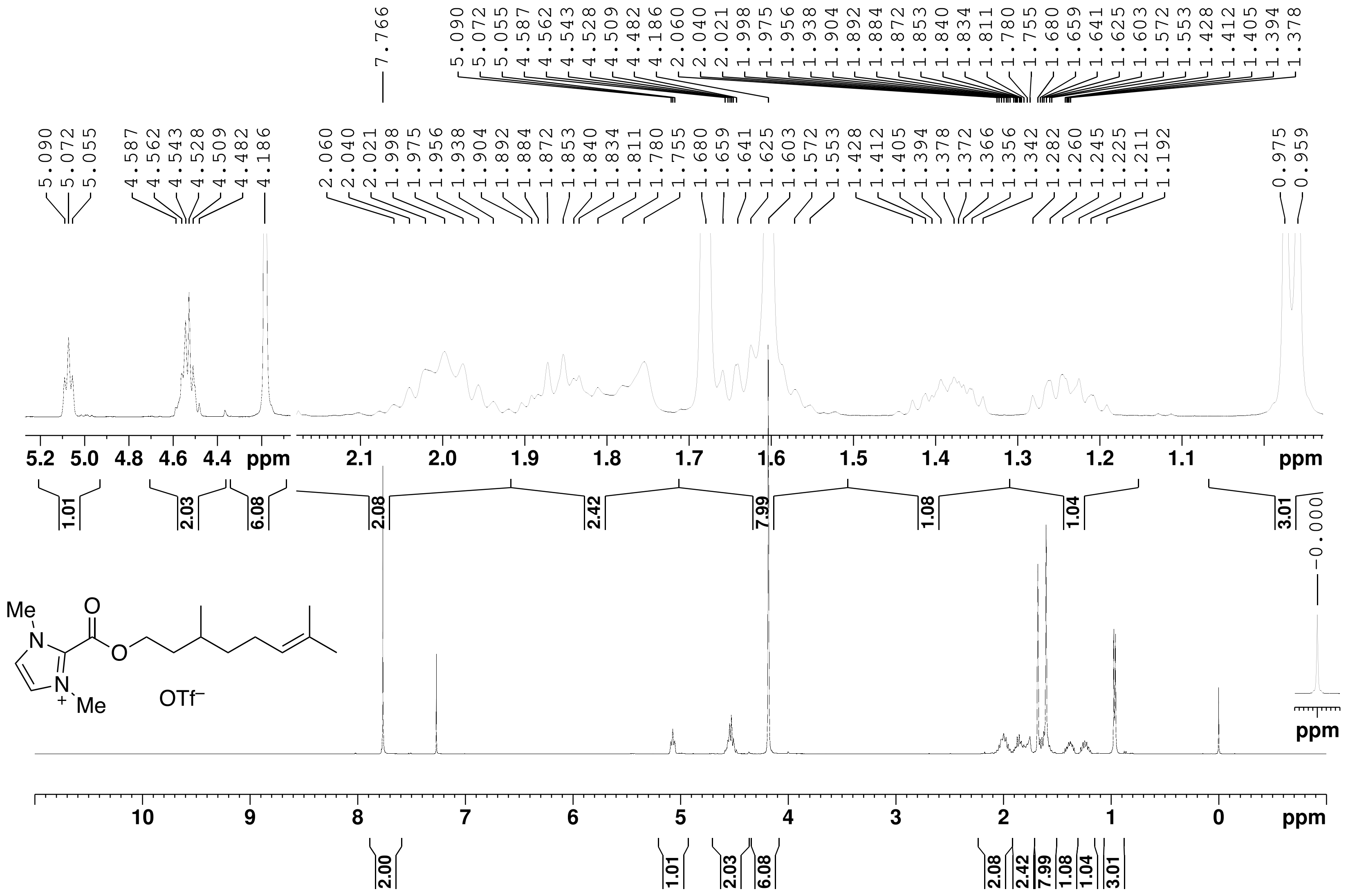

^{13}C NMR of **2e** (100.6 MHz, $CDCl_3$)

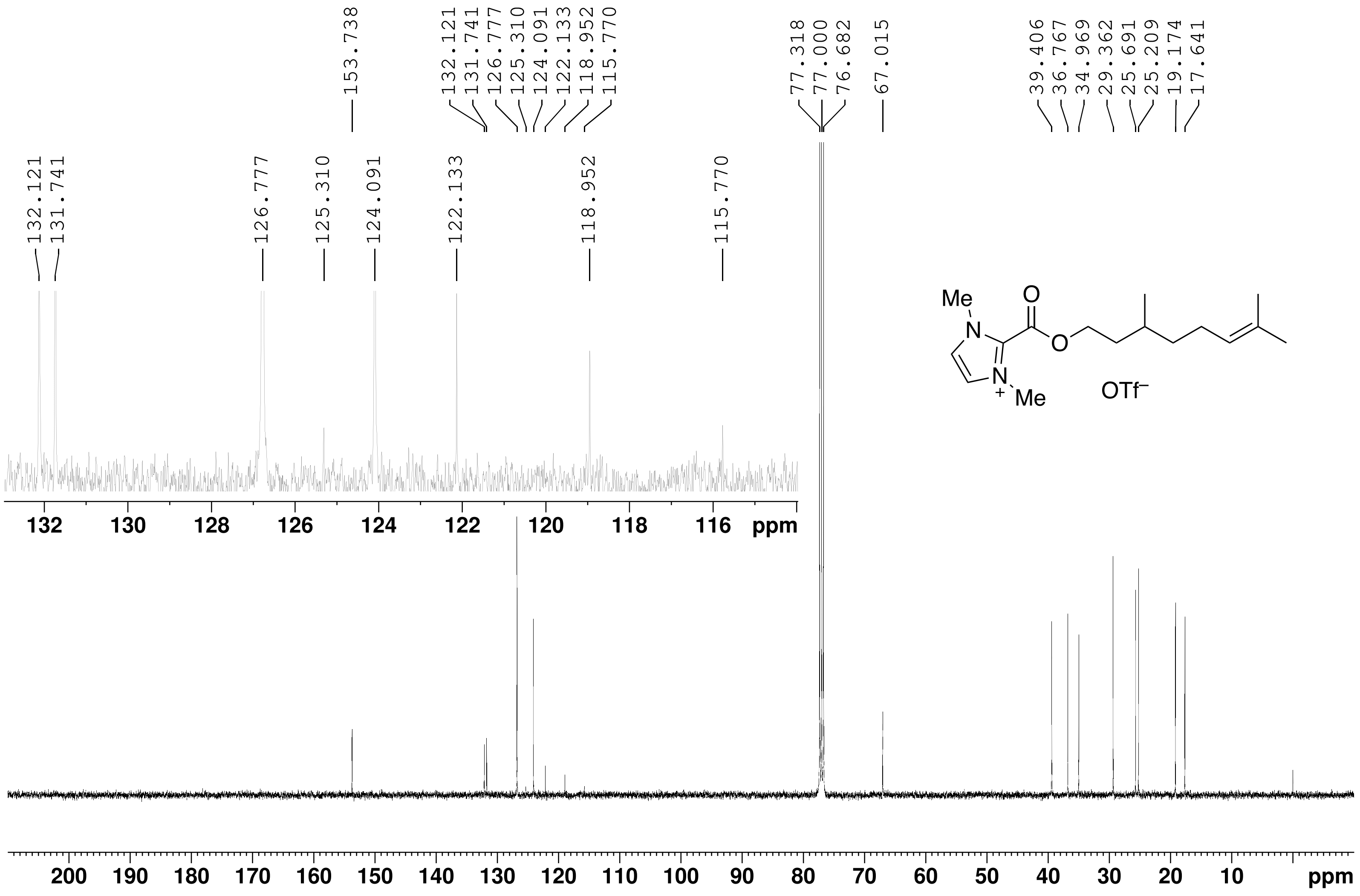

^{19}F NMR of **2e** (376.5 MHz, $CDCl_3$)

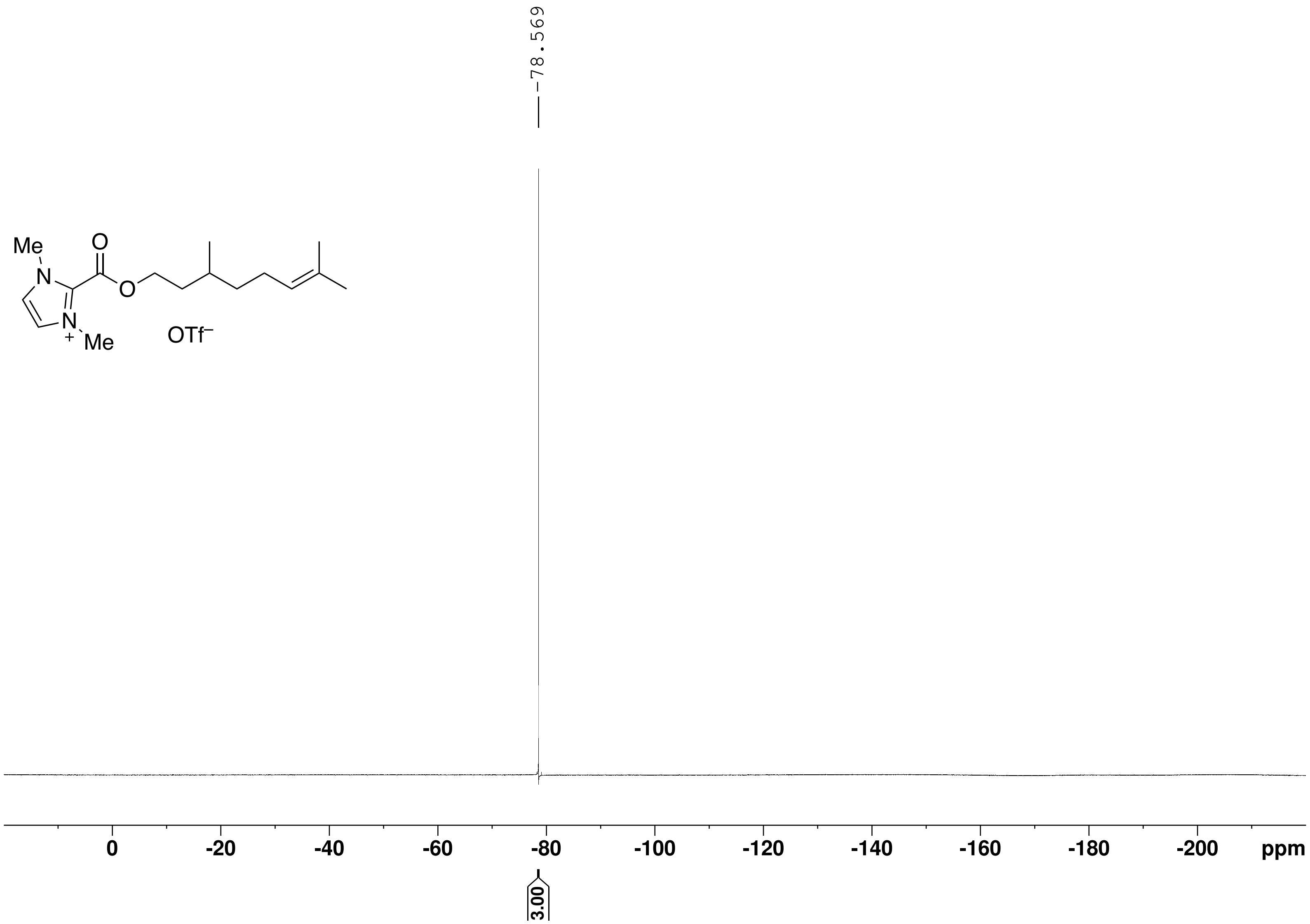

^{1}H NMR of **2f** (400 MHz, $CDCl_3$)

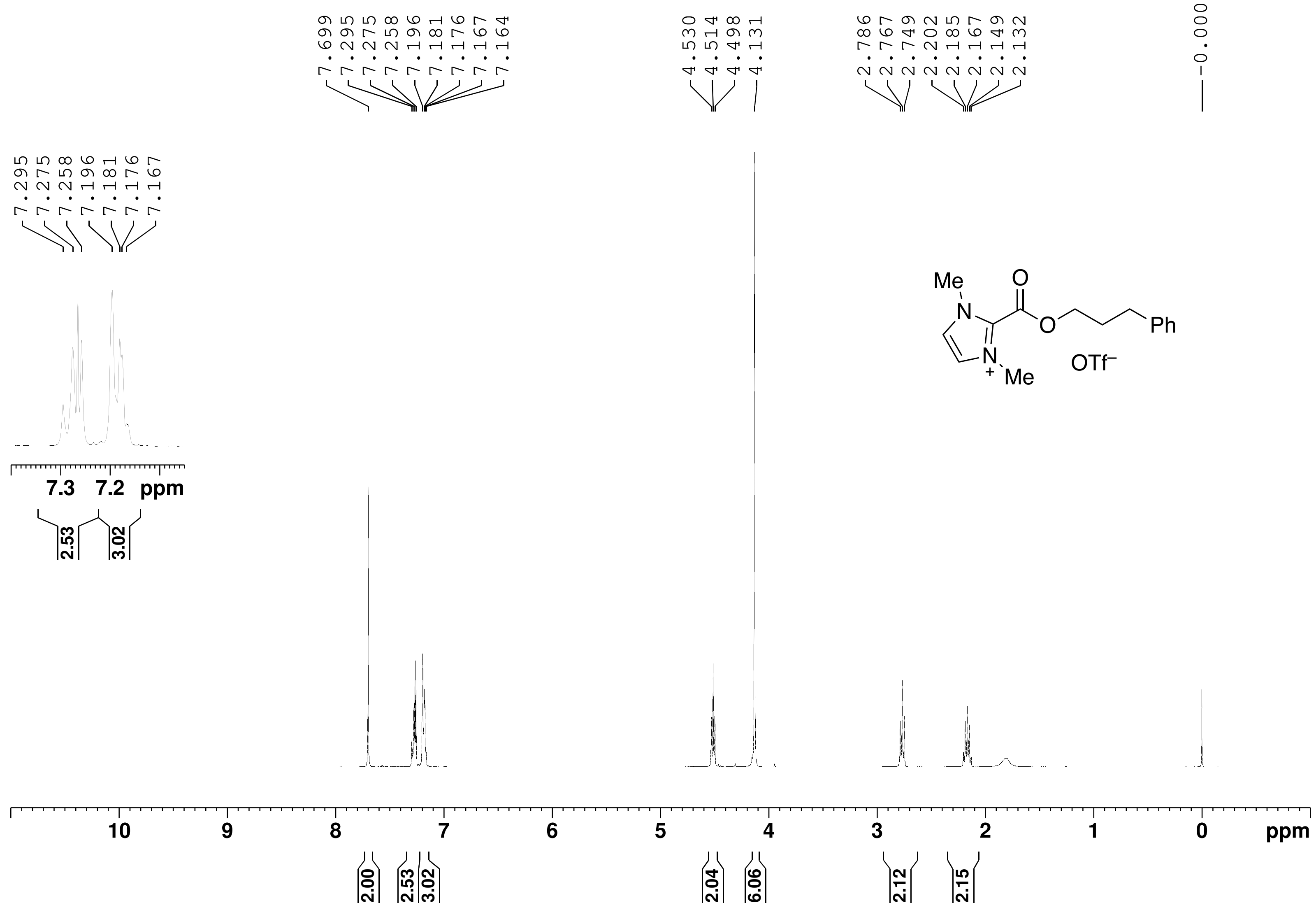

^{13}C NMR of **2f** (100.6 MHz, $CDCl_3$)

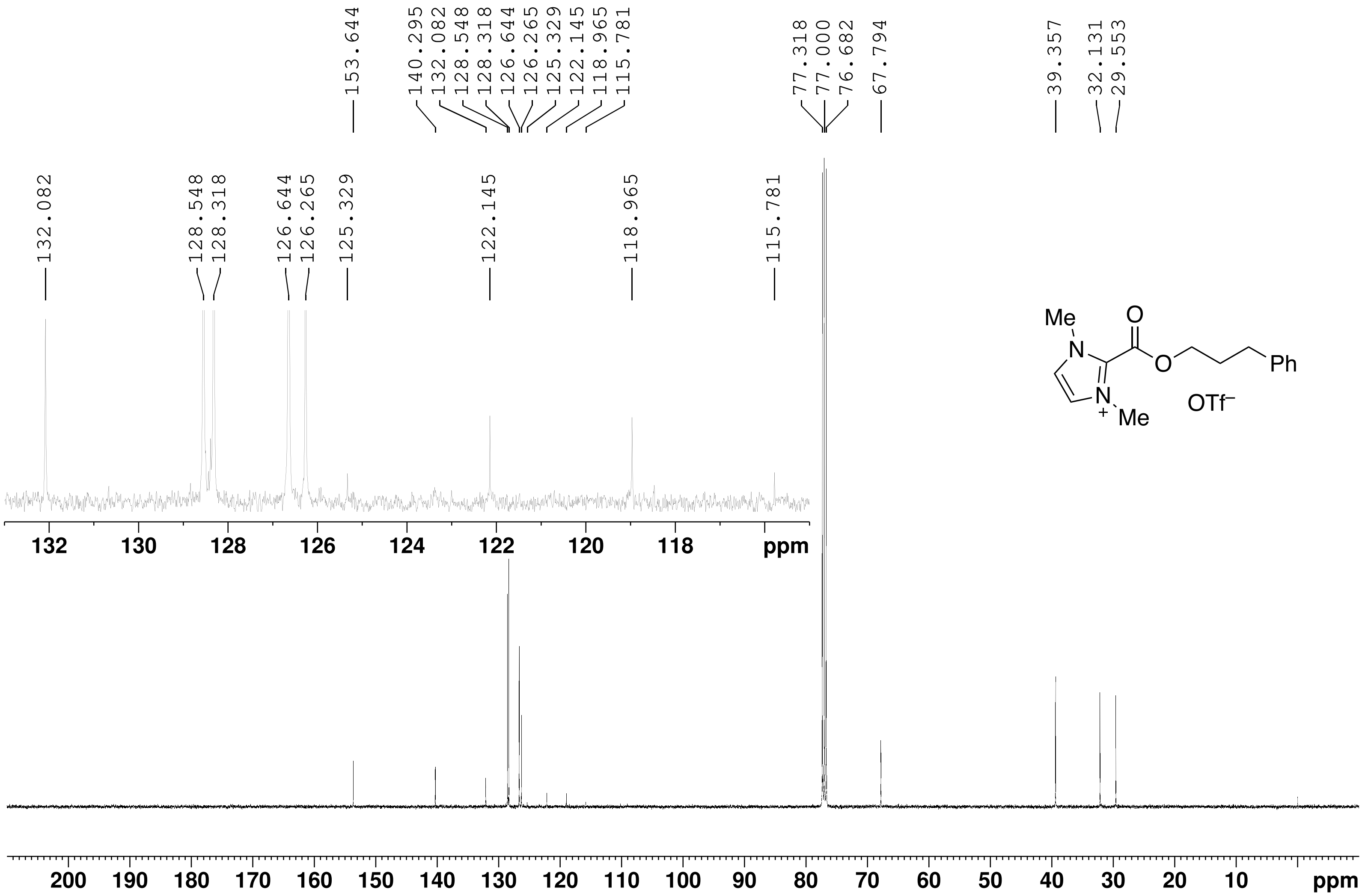

^{19}F NMR of **2f** (376.5 MHz, $CDCl_3$)

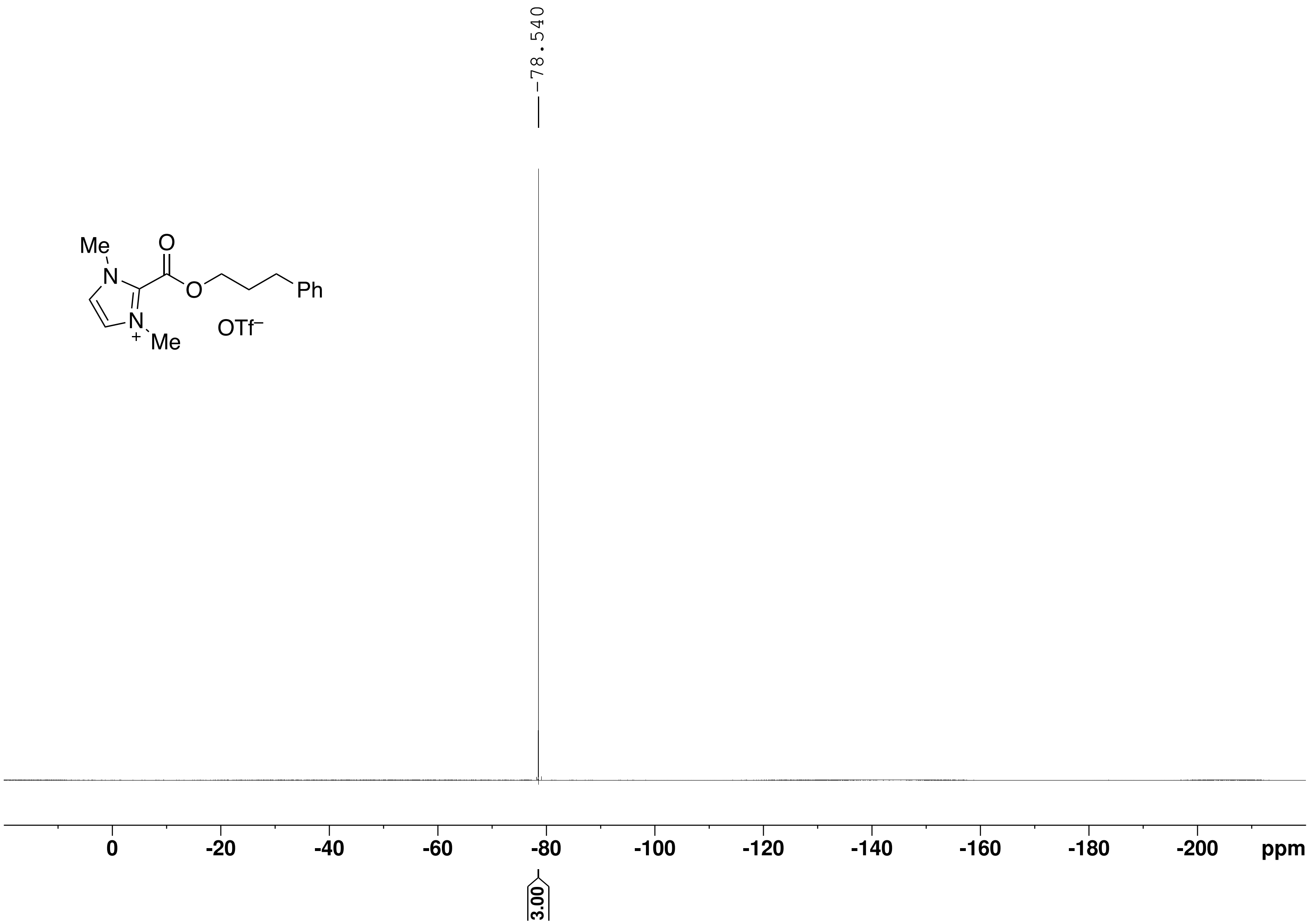

^{1}H NMR of **2g** (400 MHz, DMSO-d_6)

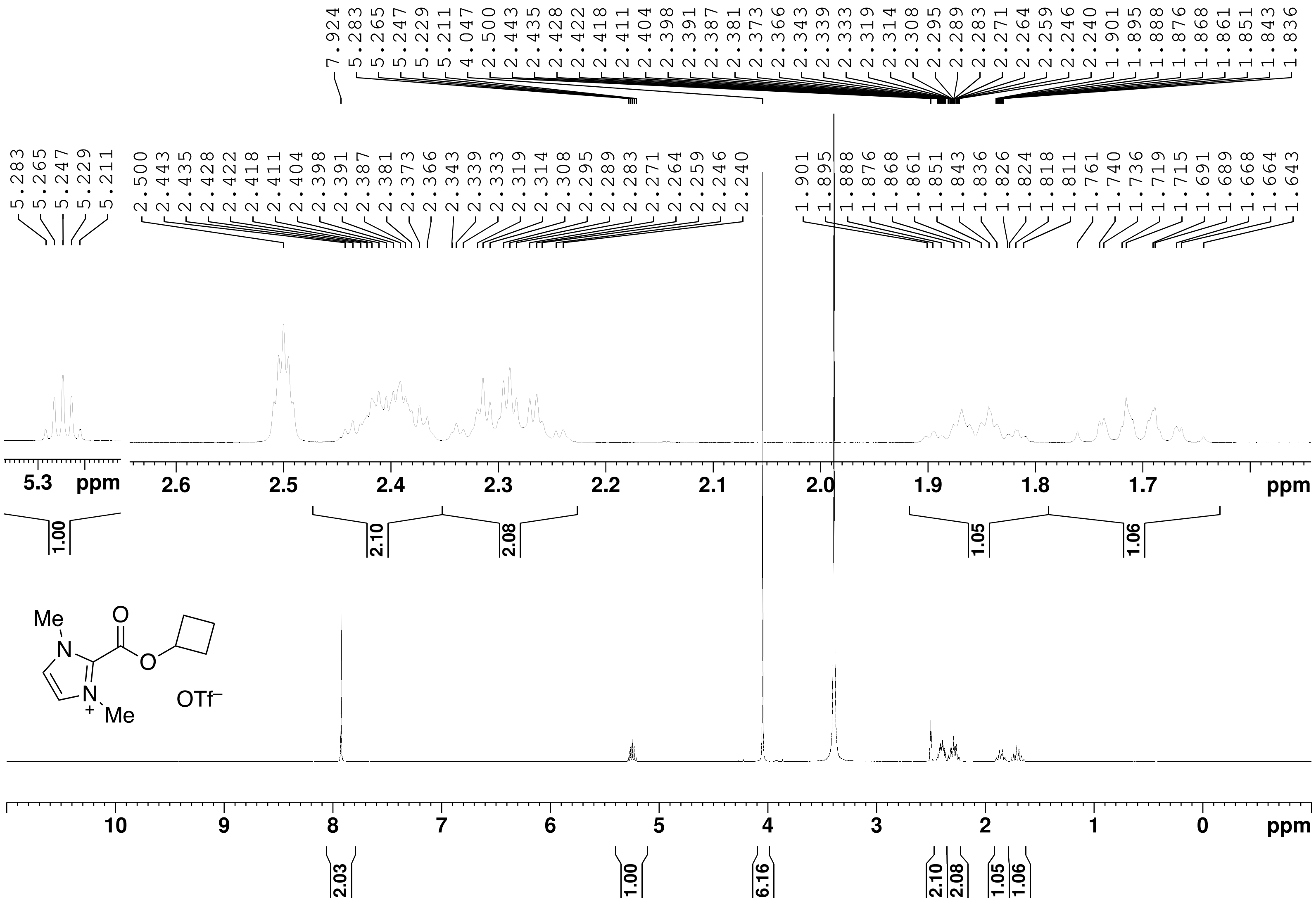

^{13}C NMR of **2g** (100.6 MHz, DMSO-d6)

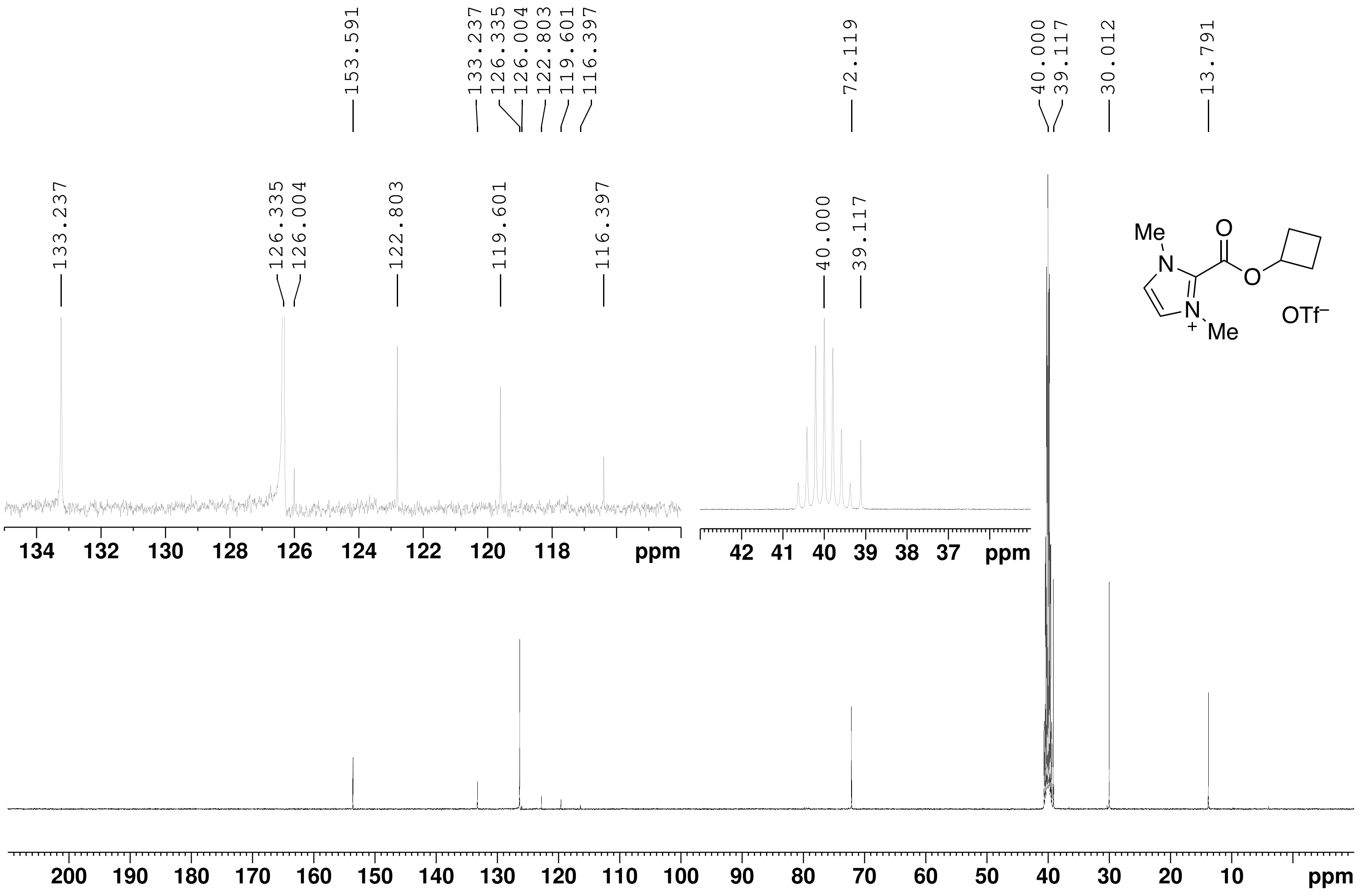

^{19}F NMR of **2g** (376.5 MHz, DMSO-d_6)

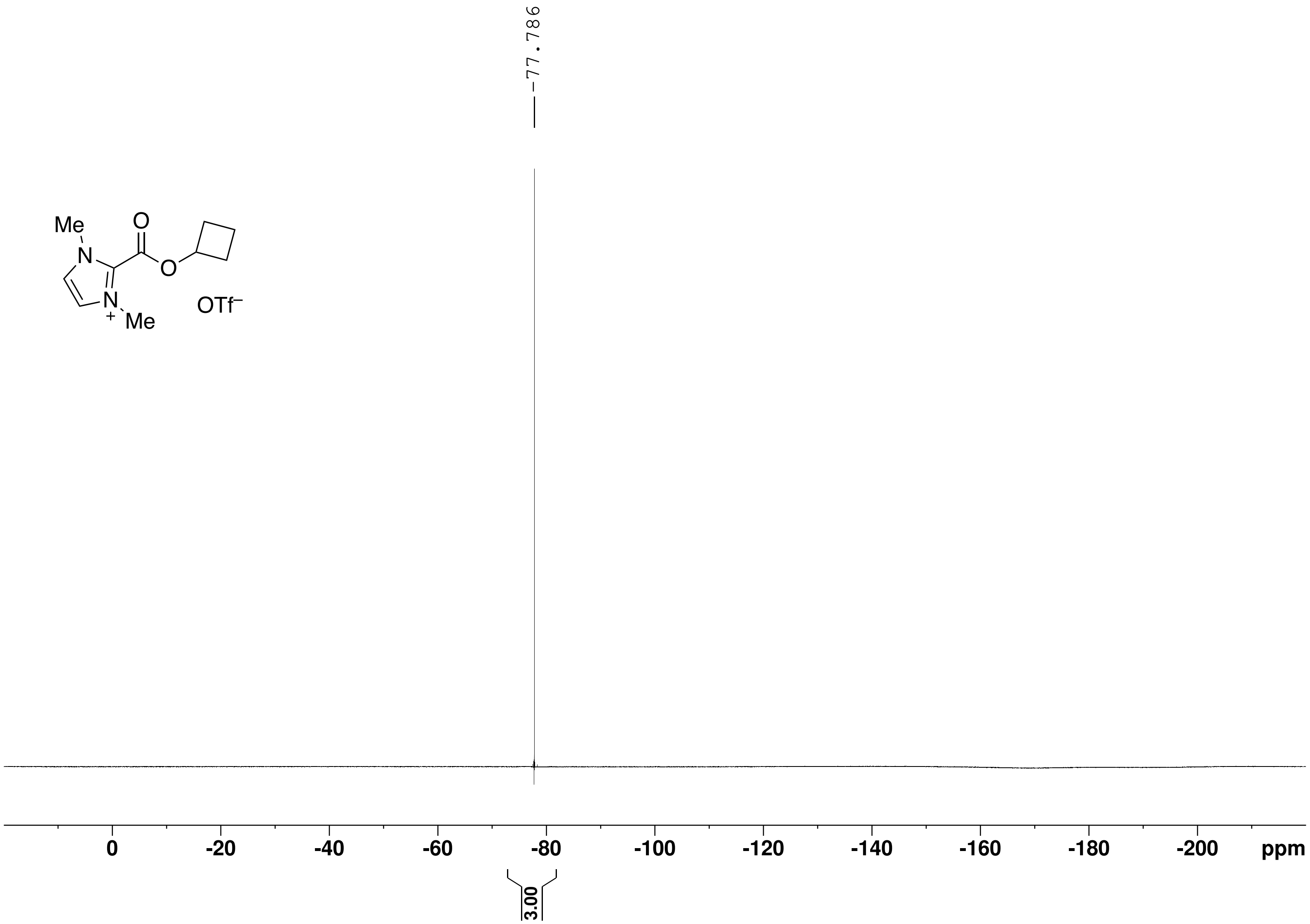

^{1}H NMR of **4a** (400 MHz, $CDCl_3$)

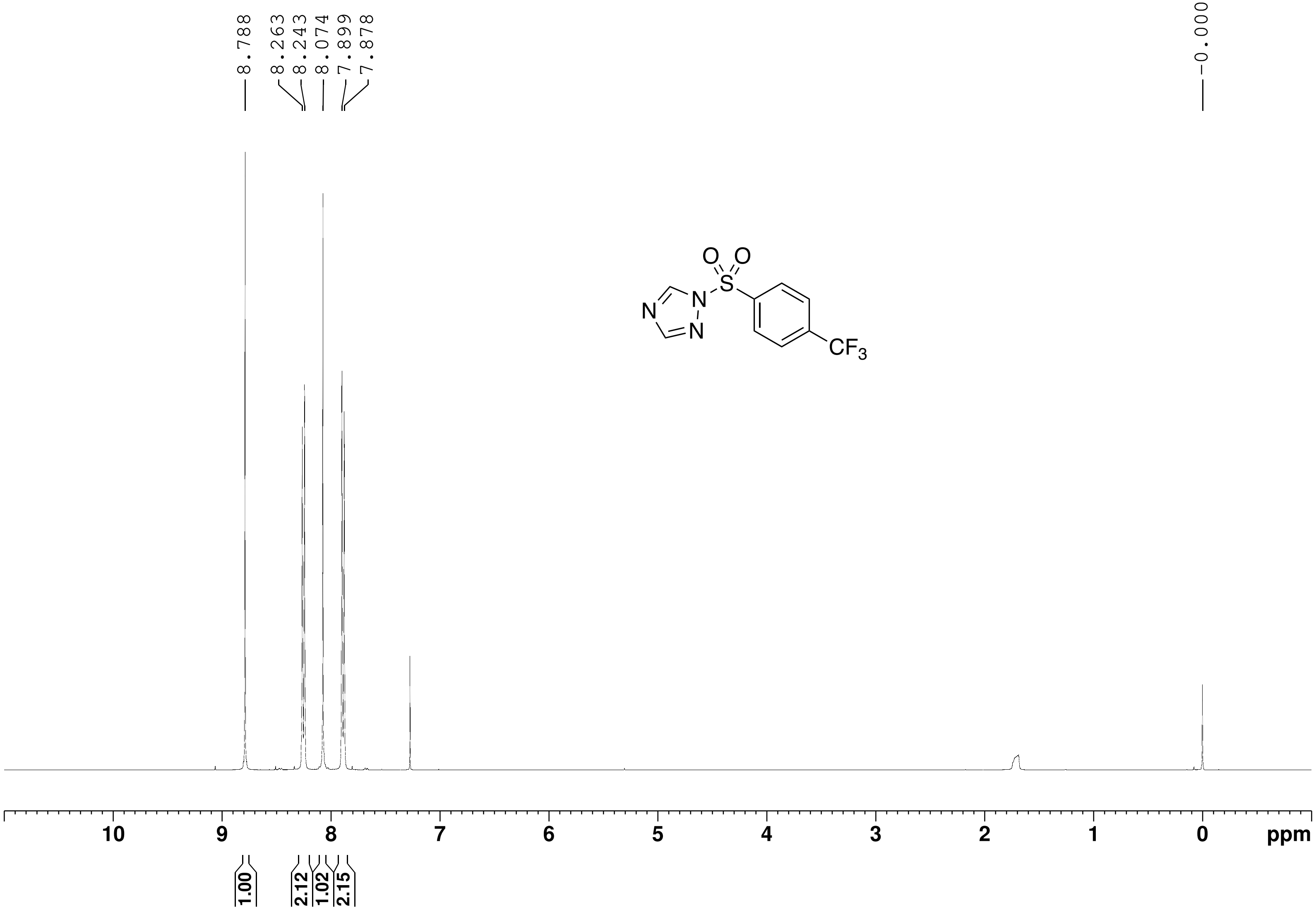

^{13}C NMR of **4a** (100.6 MHz, $CDCl_3$)

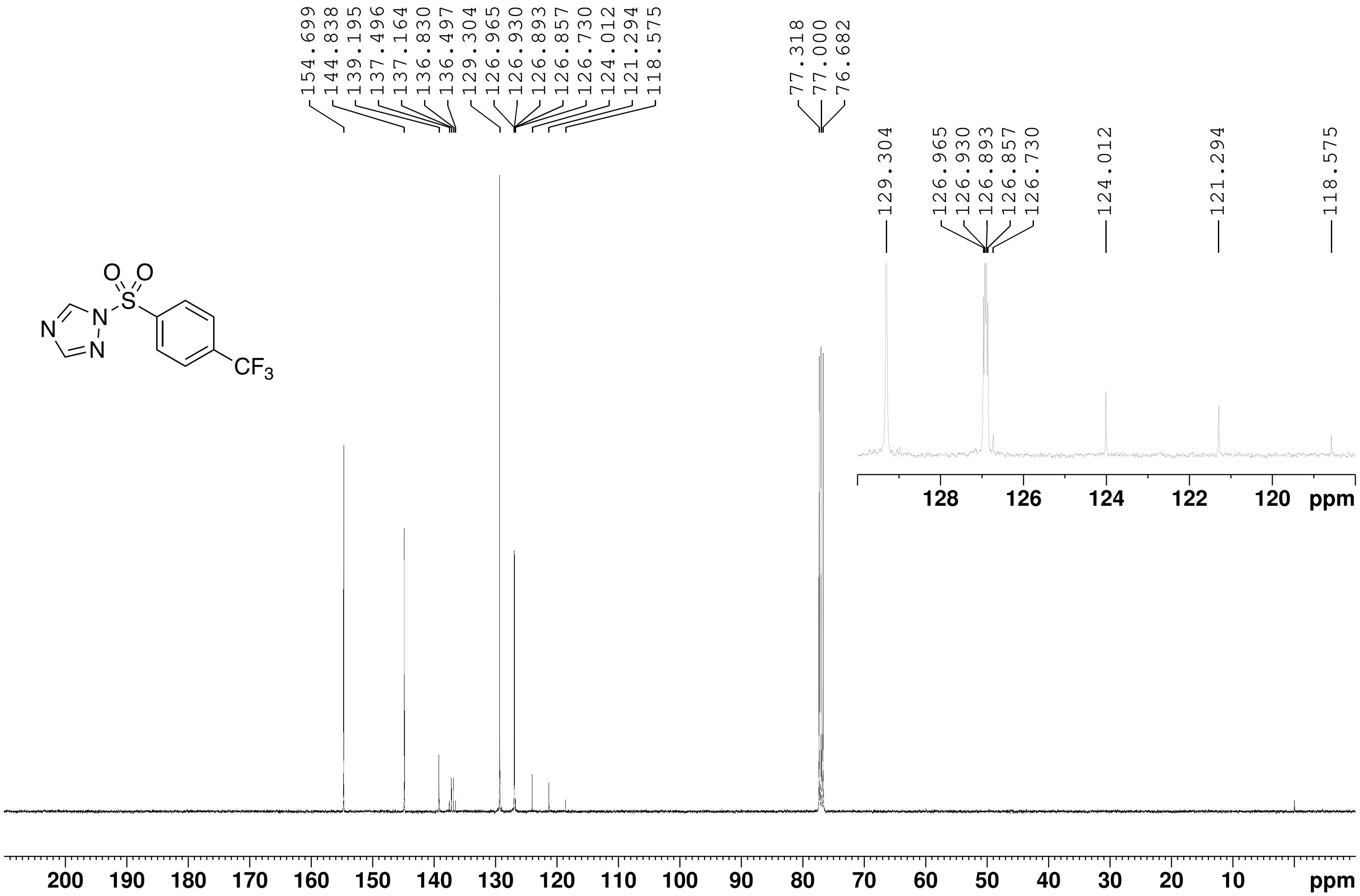

^{19}F NMR of **4a** (376.5 MHz, CDCl$_3$)

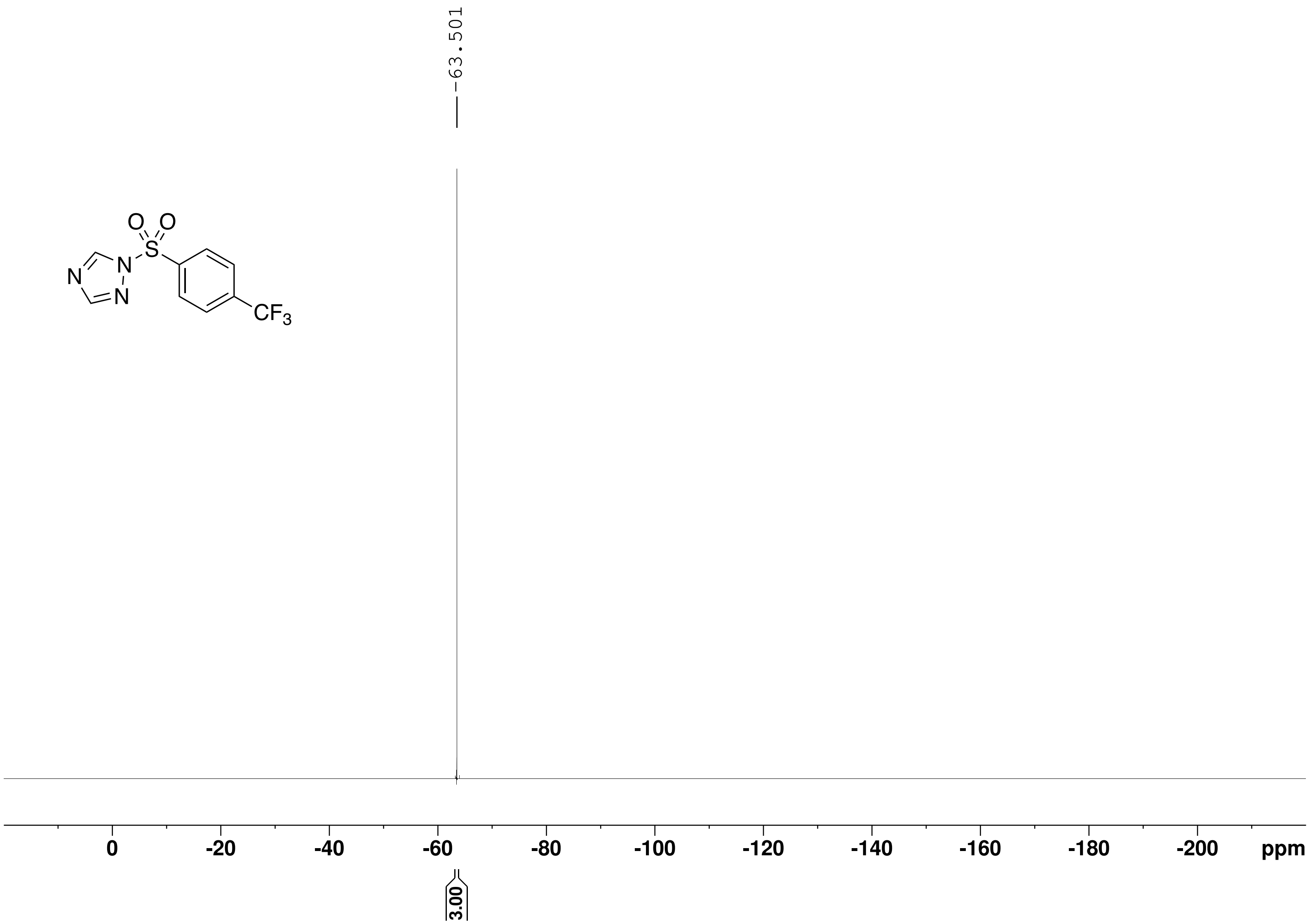

^{1}H NMR of **4b** (400 MHz, $CDCl_3$)

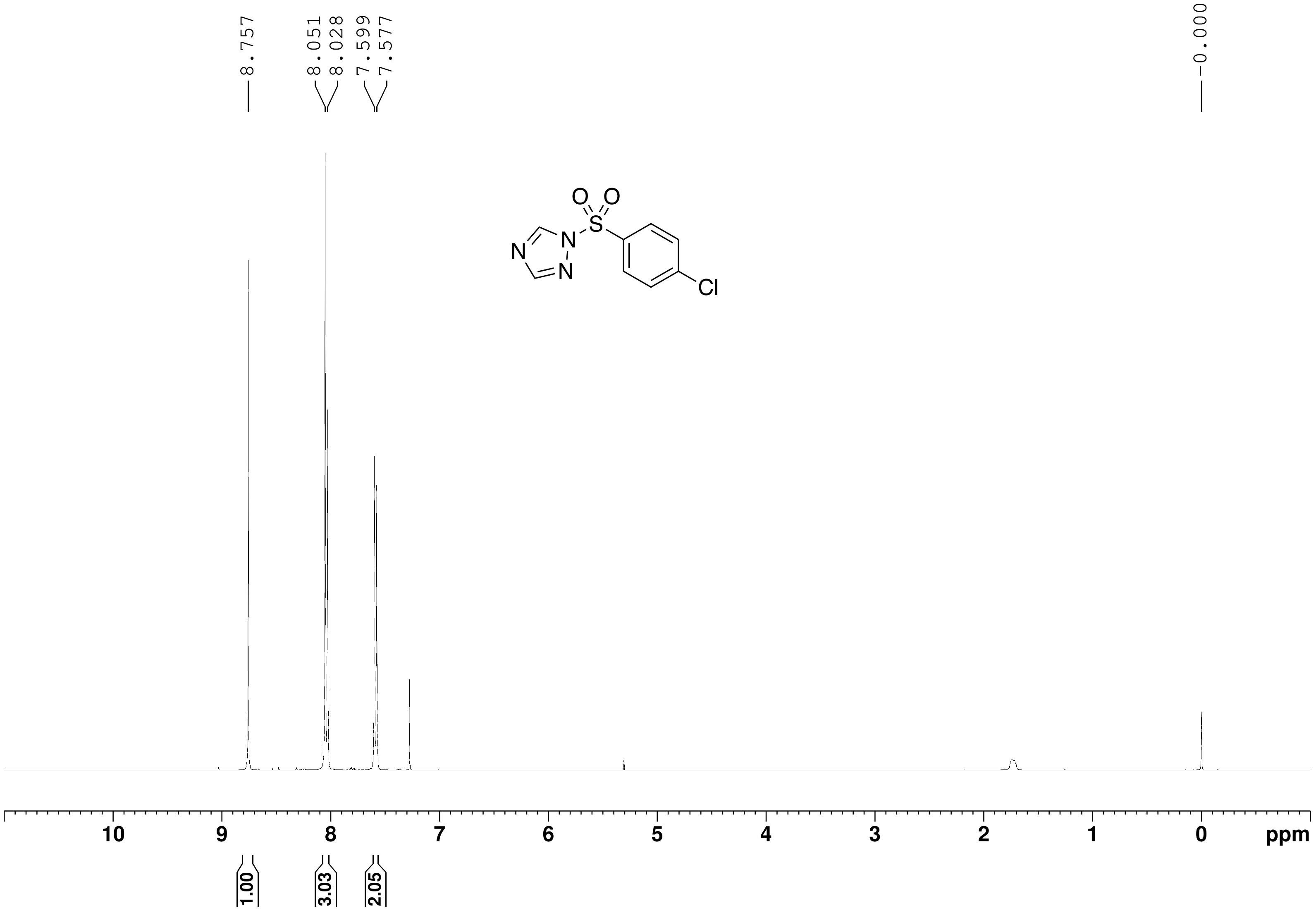

^{13}C NMR of **4b** (100.6 MHz, $CDCl_3$)

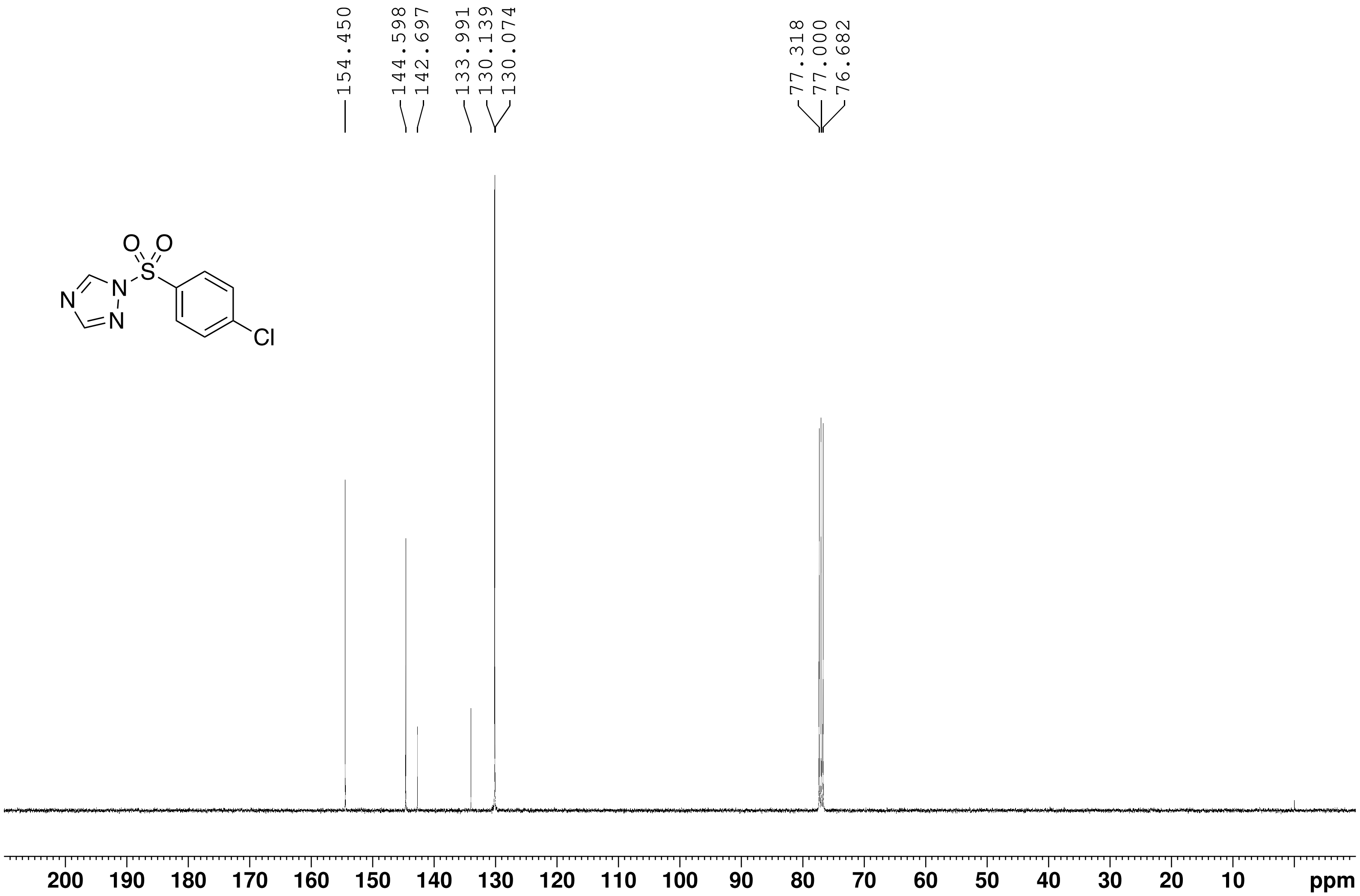

^{1}H NMR of **4c** (400 MHz, $CDCl_3$)

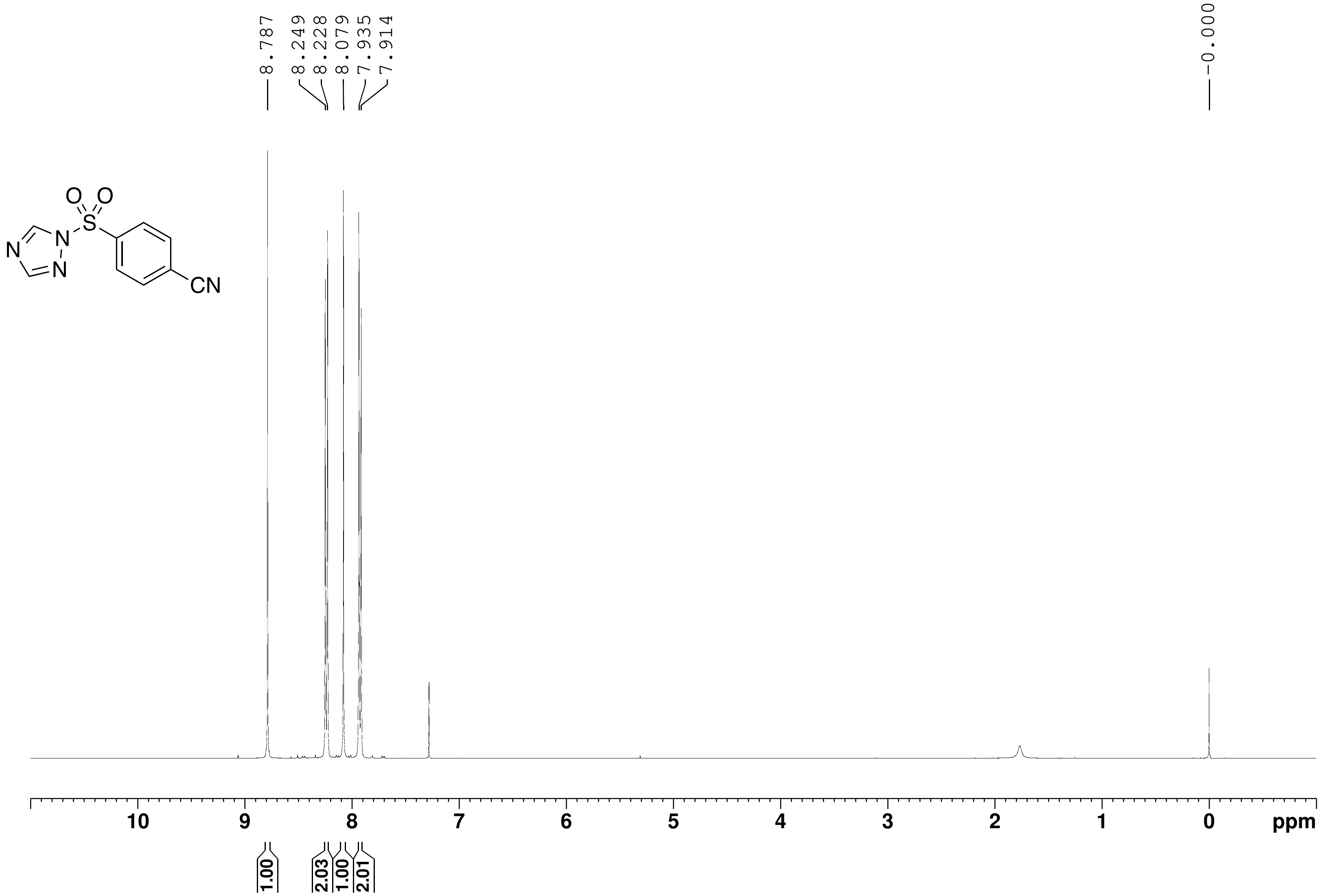

^{13}C NMR of **4c** (100.6 MHz, $CDCl_3$)

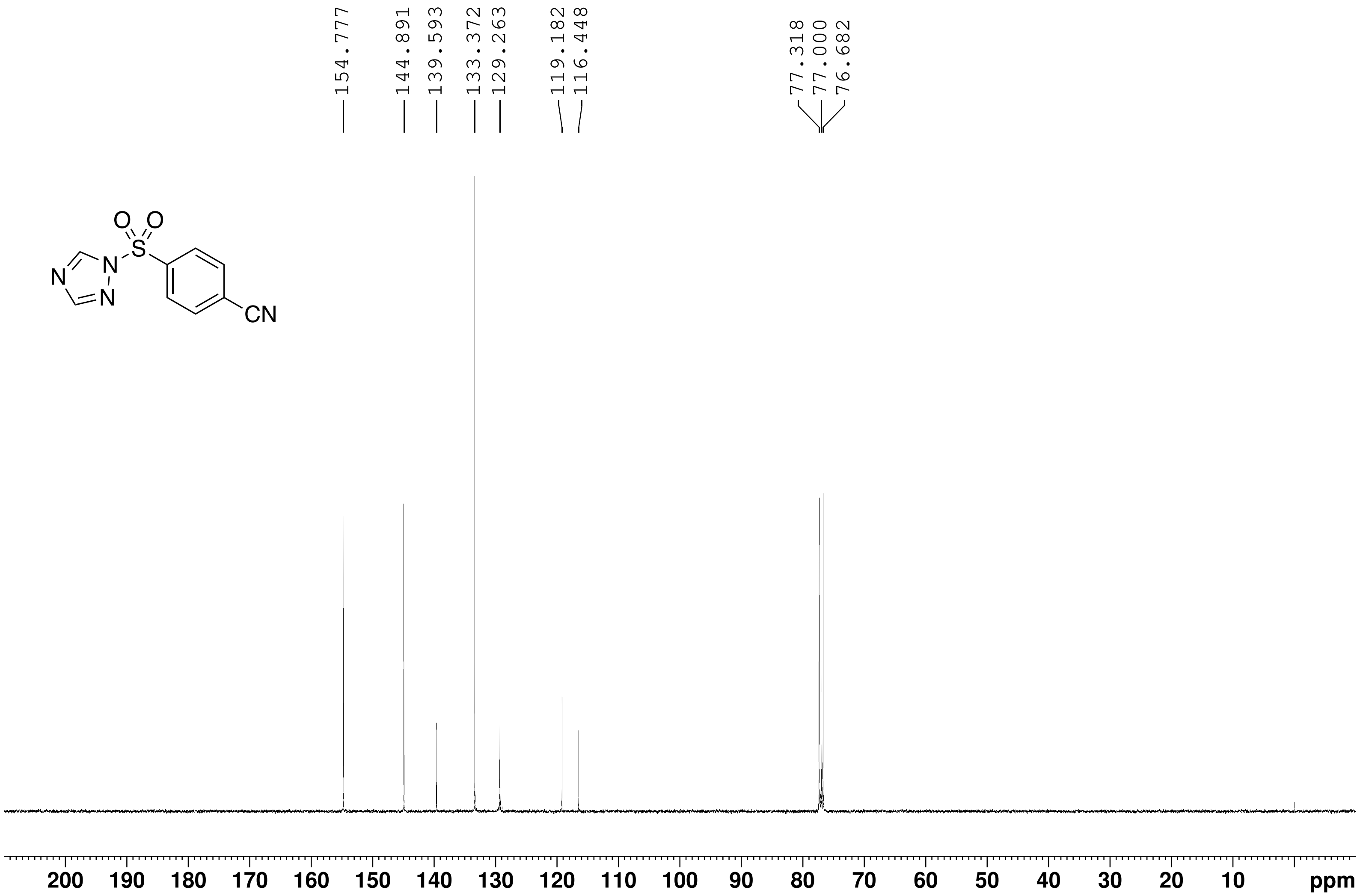

^{1}H NMR of **4d** (400 MHz, $CDCl_3$)

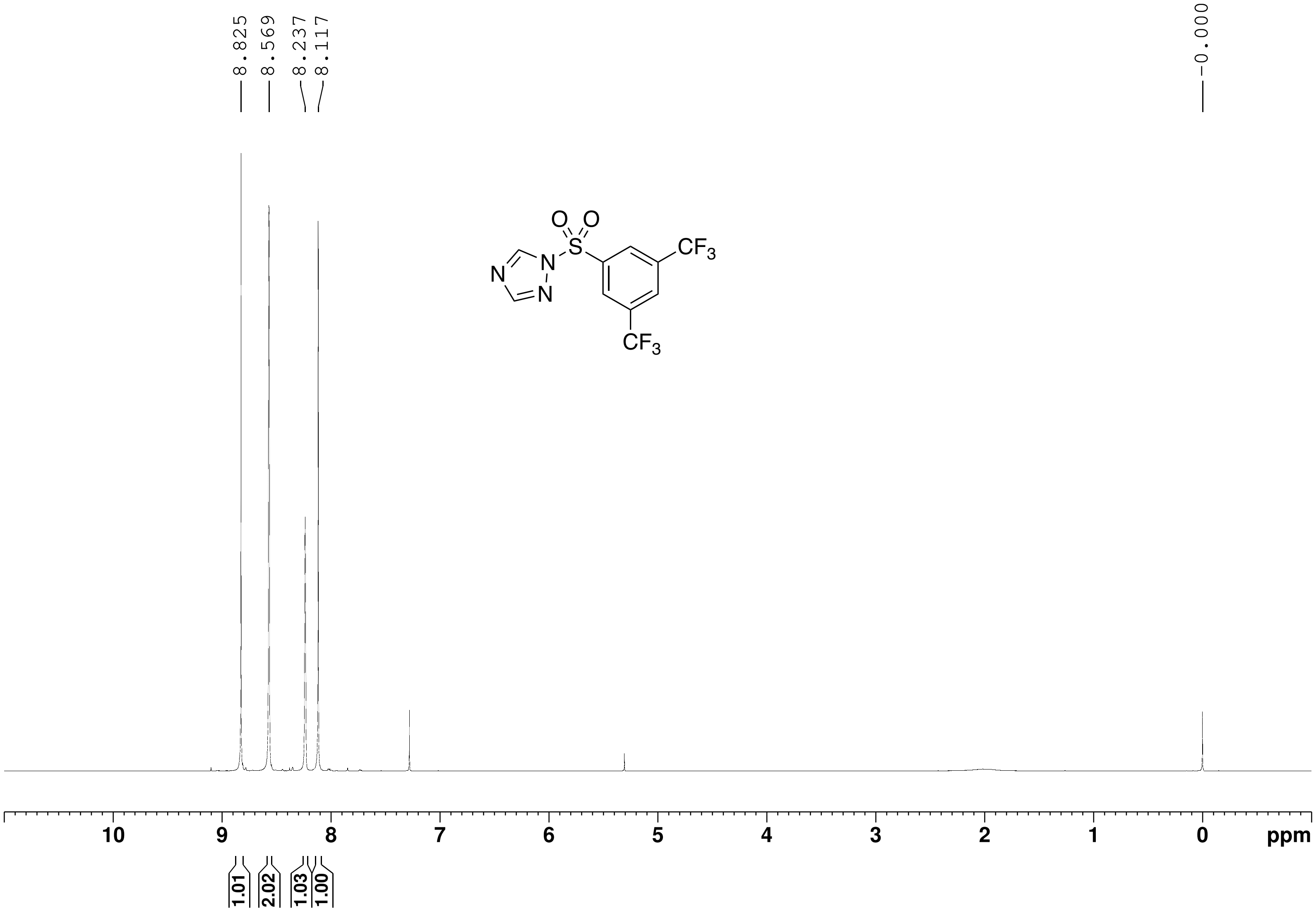

^{13}C NMR of **4d** (100.6 MHz, $CDCl_3$)

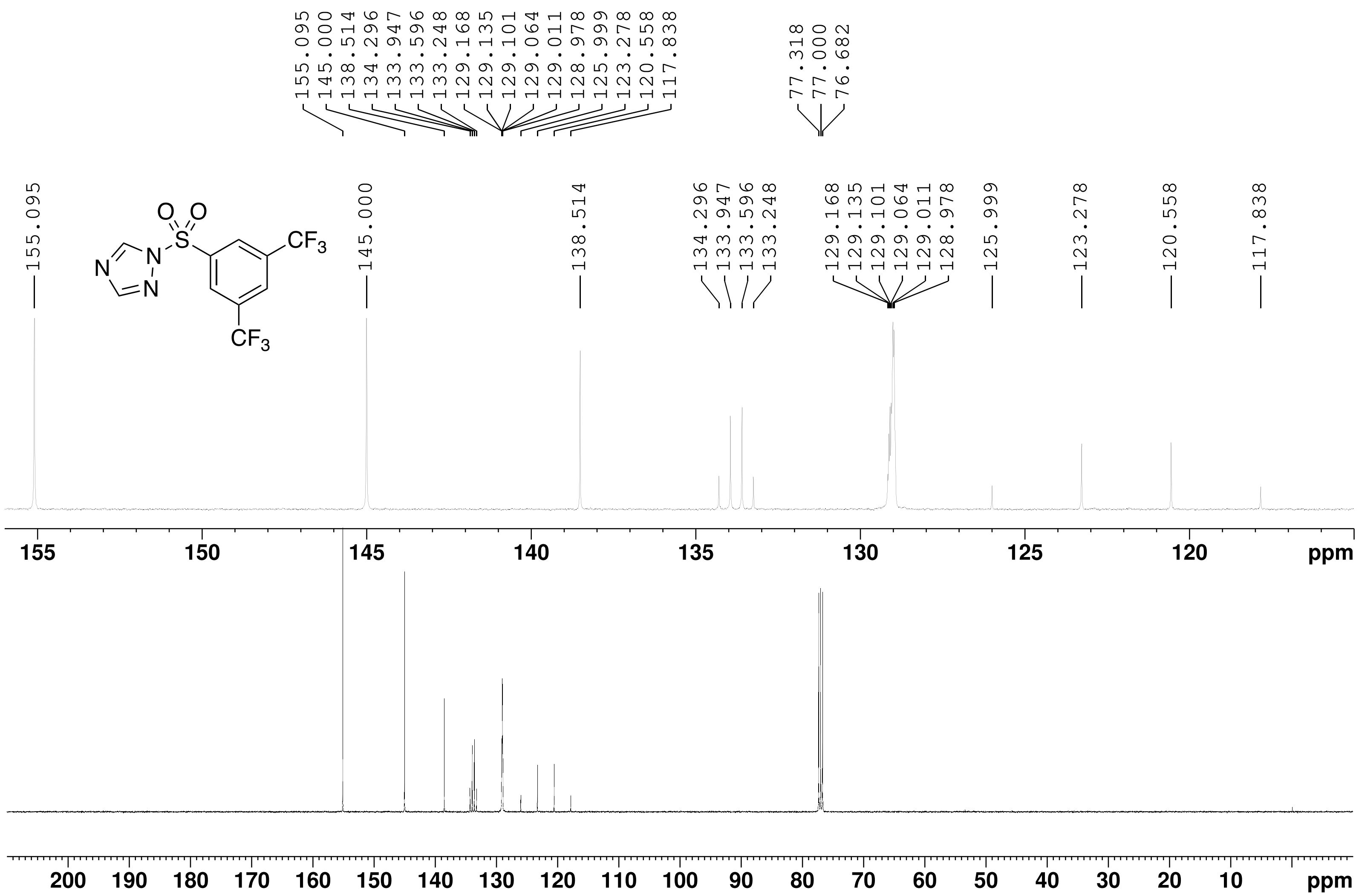

^{19}F NMR of **4d** (376.5 MHz, $CDCl_3$)

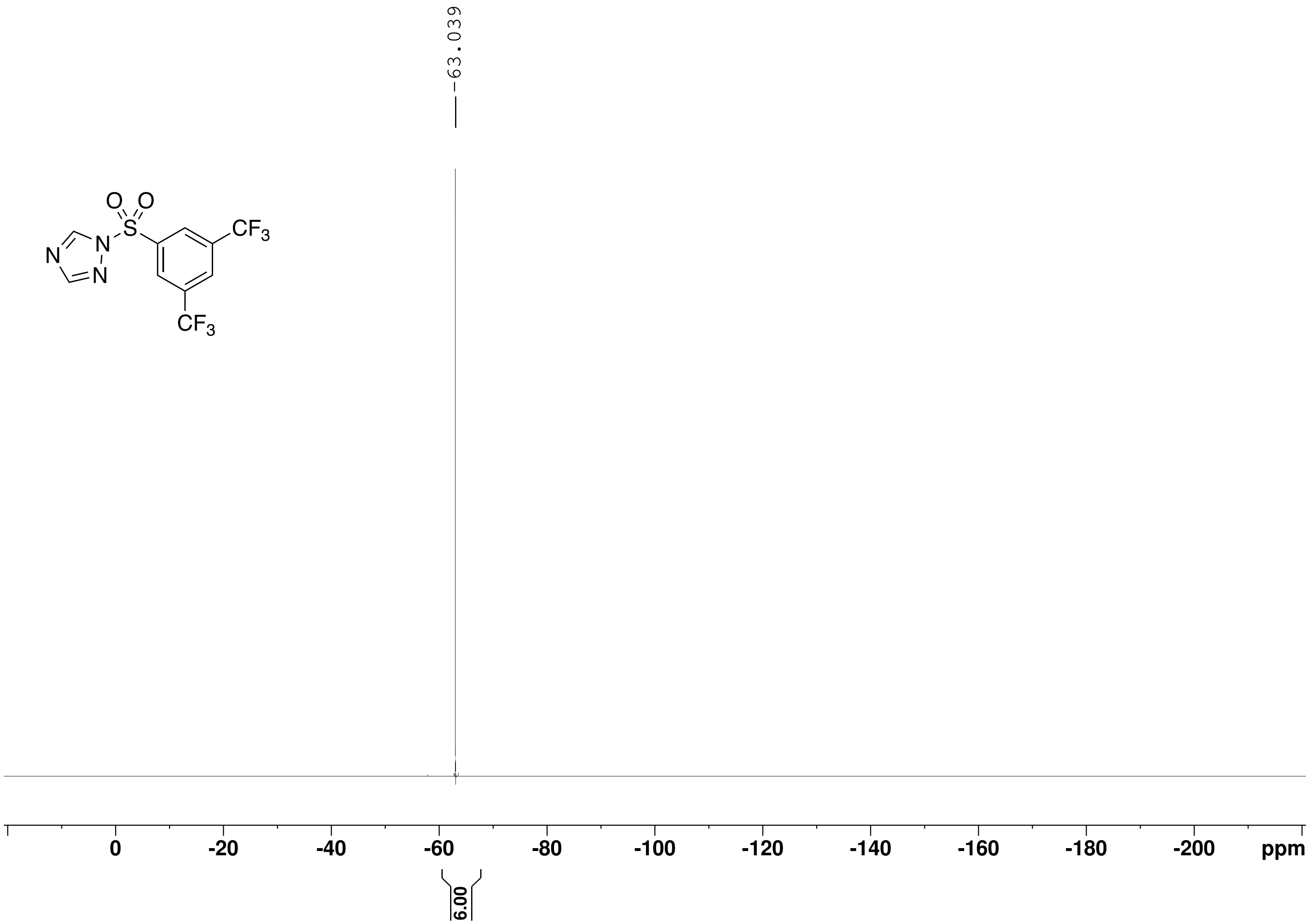

^{1}H NMR of **4f** (400 MHz, $CDCl_3$)

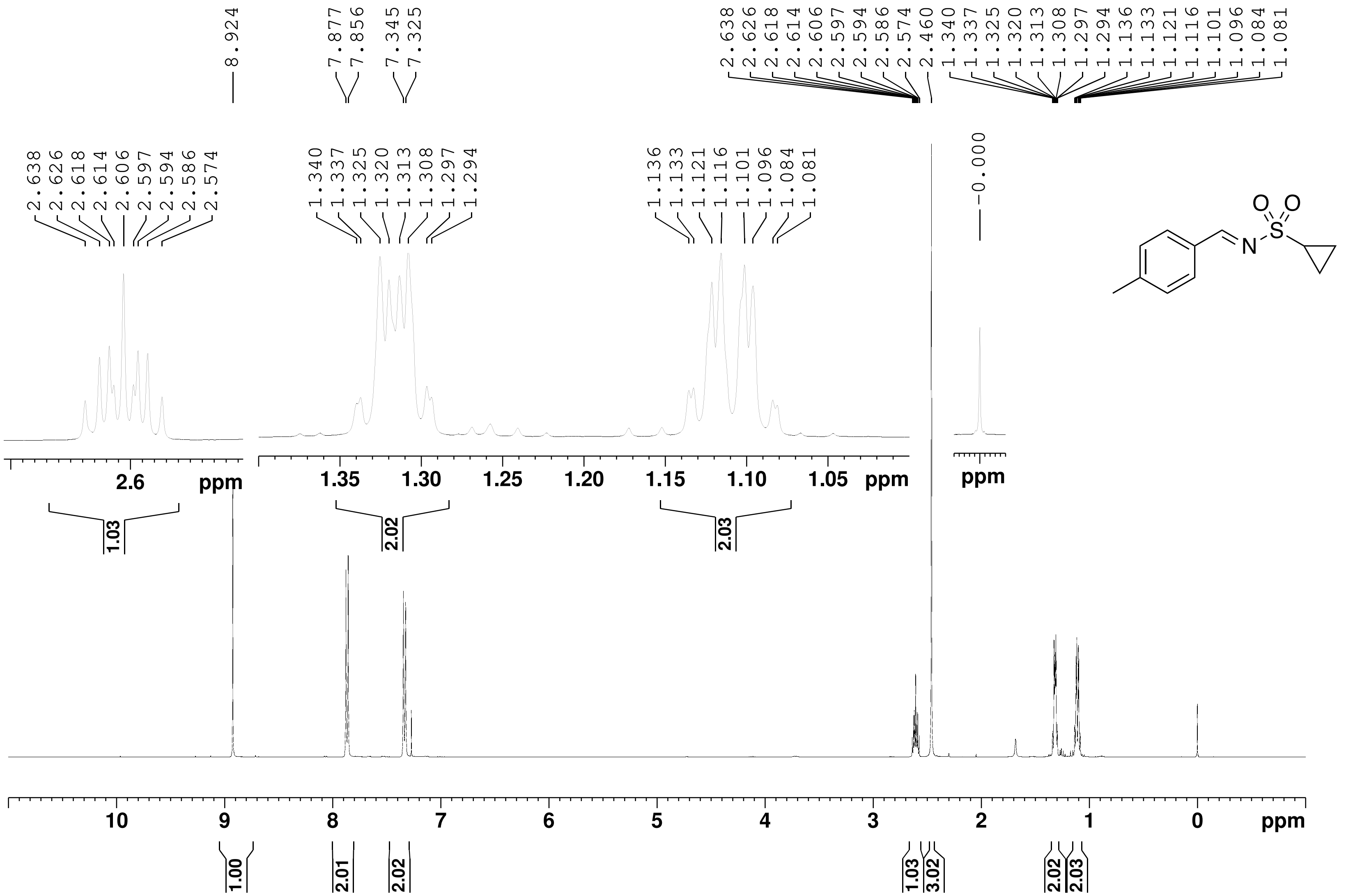

^{13}C NMR of **4f** (100.6 MHz, CDCl$_3$)

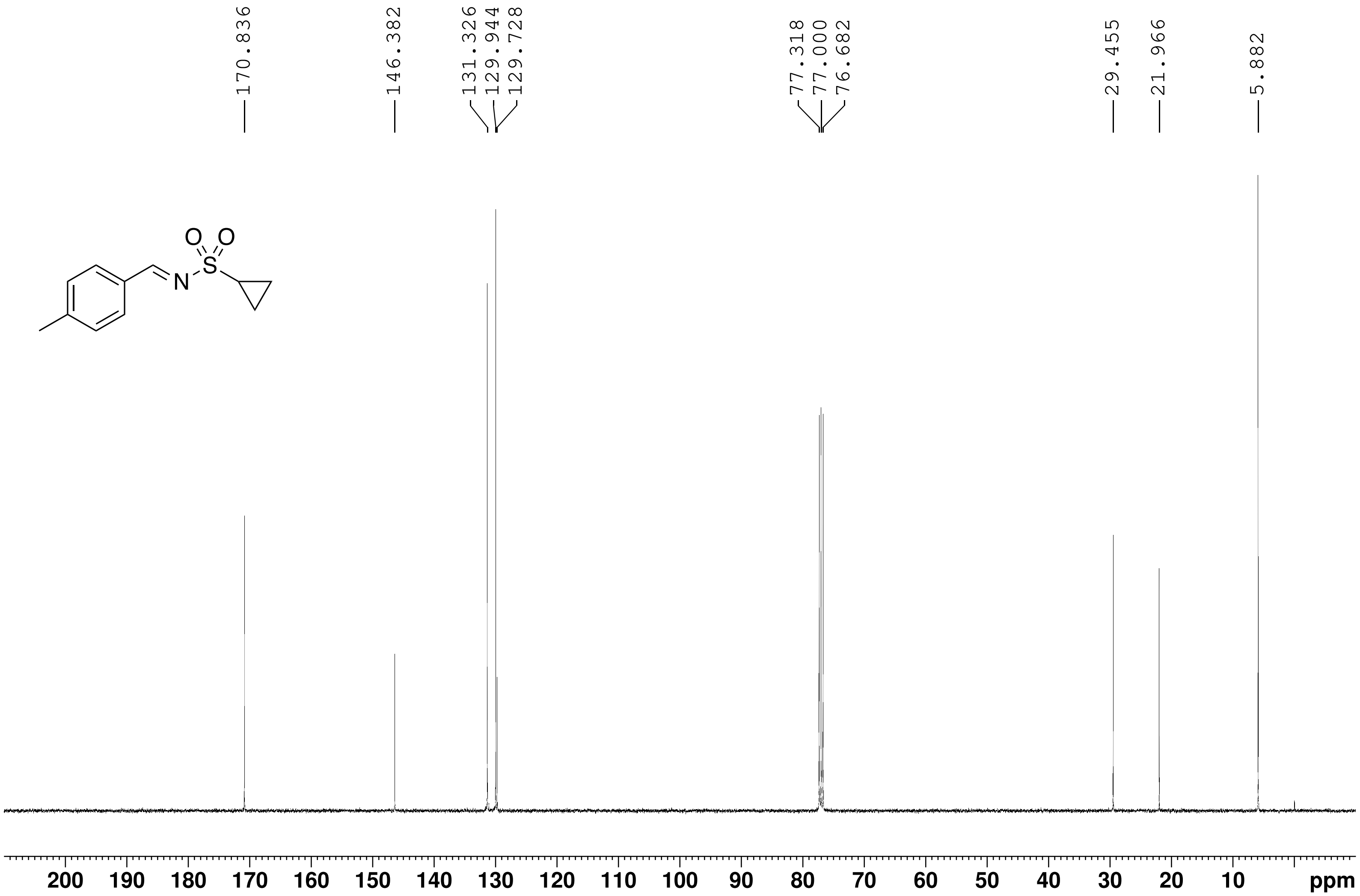

^{1}H NMR of **3aa** (400 MHz, $CDCl_3$)

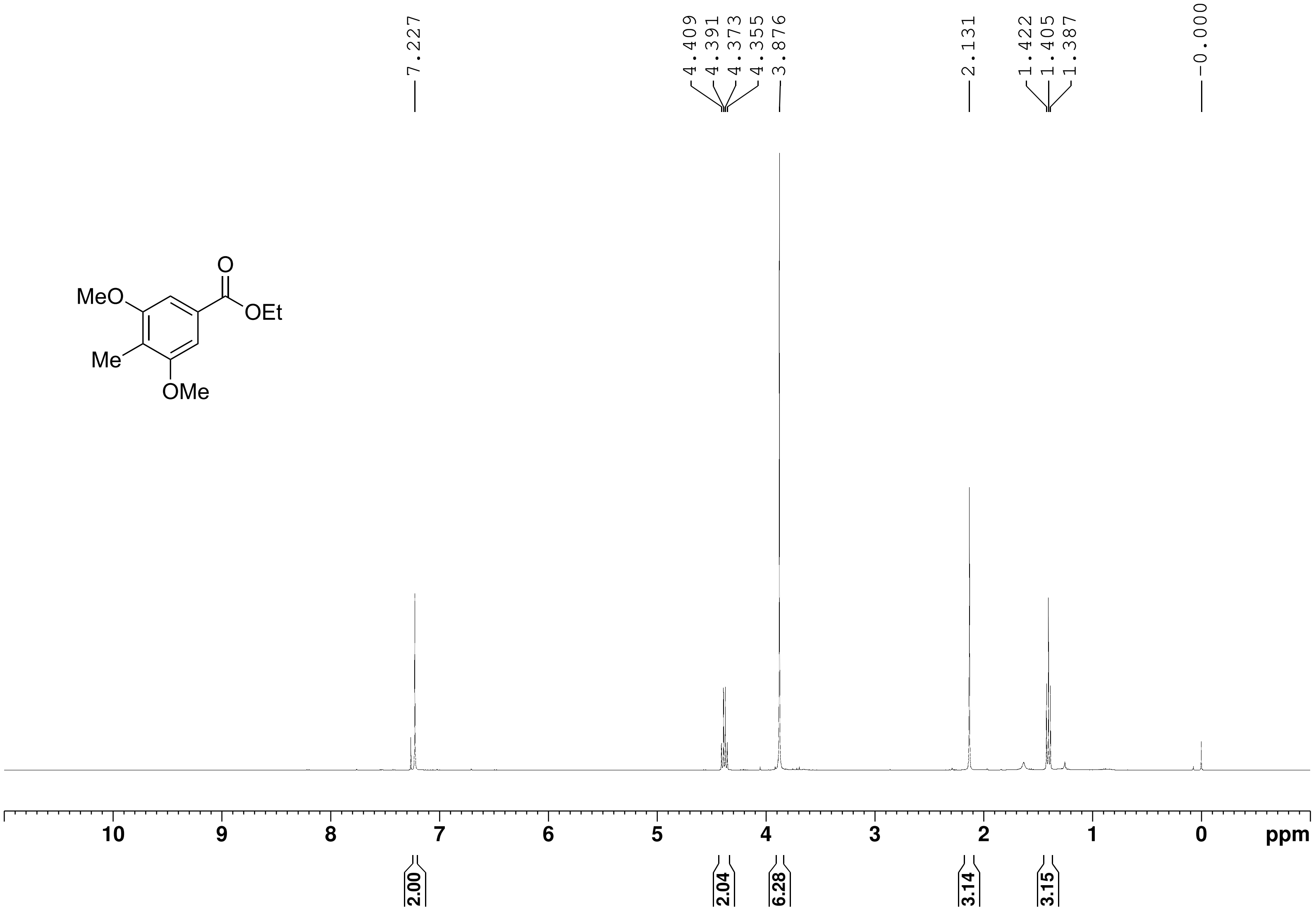

^{13}C NMR of **3aa** (100.6 MHz, $CDCl_3$)

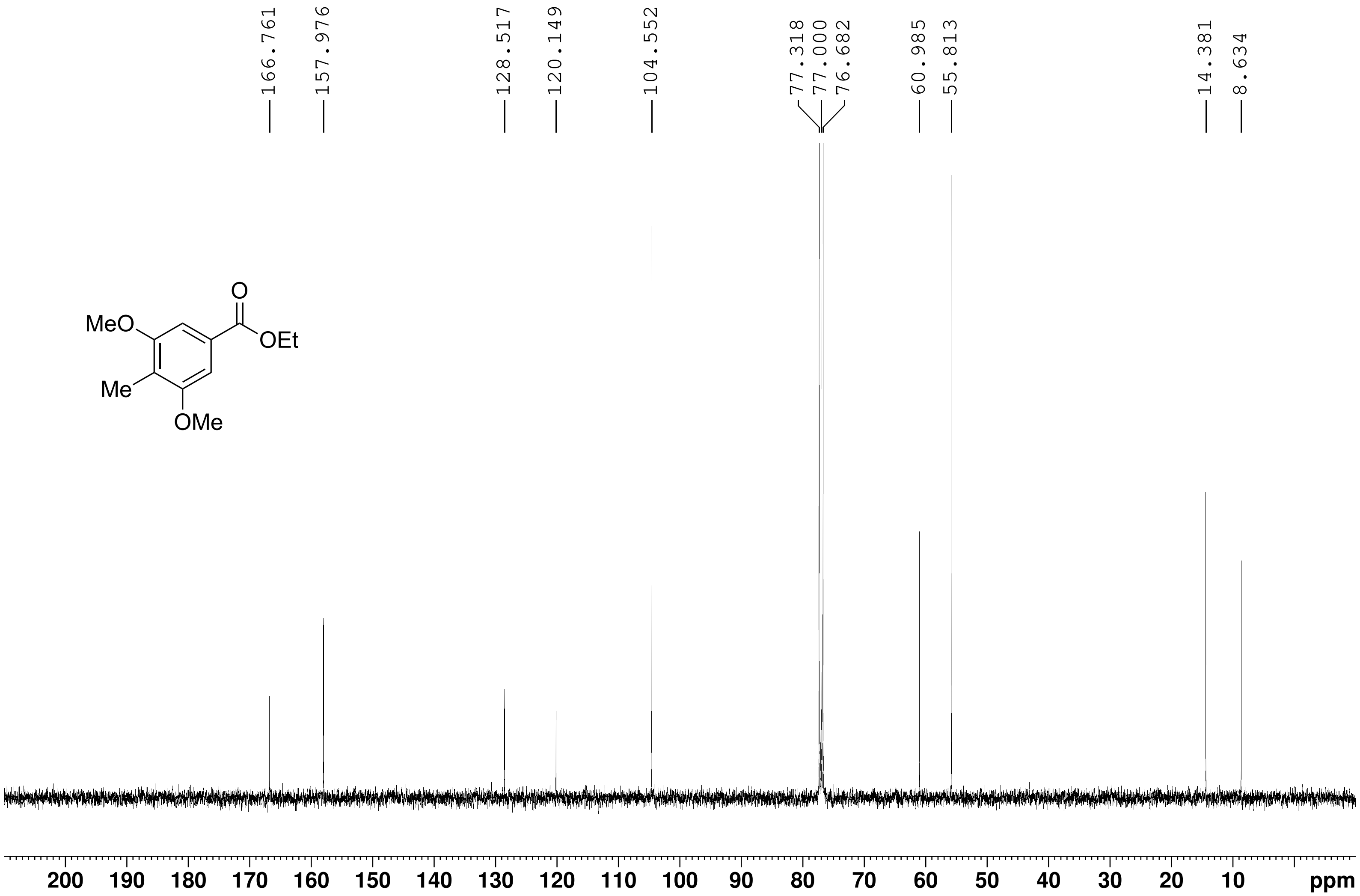

^{1}H NMR of **3ab** (400 MHz, $CDCl_3$)

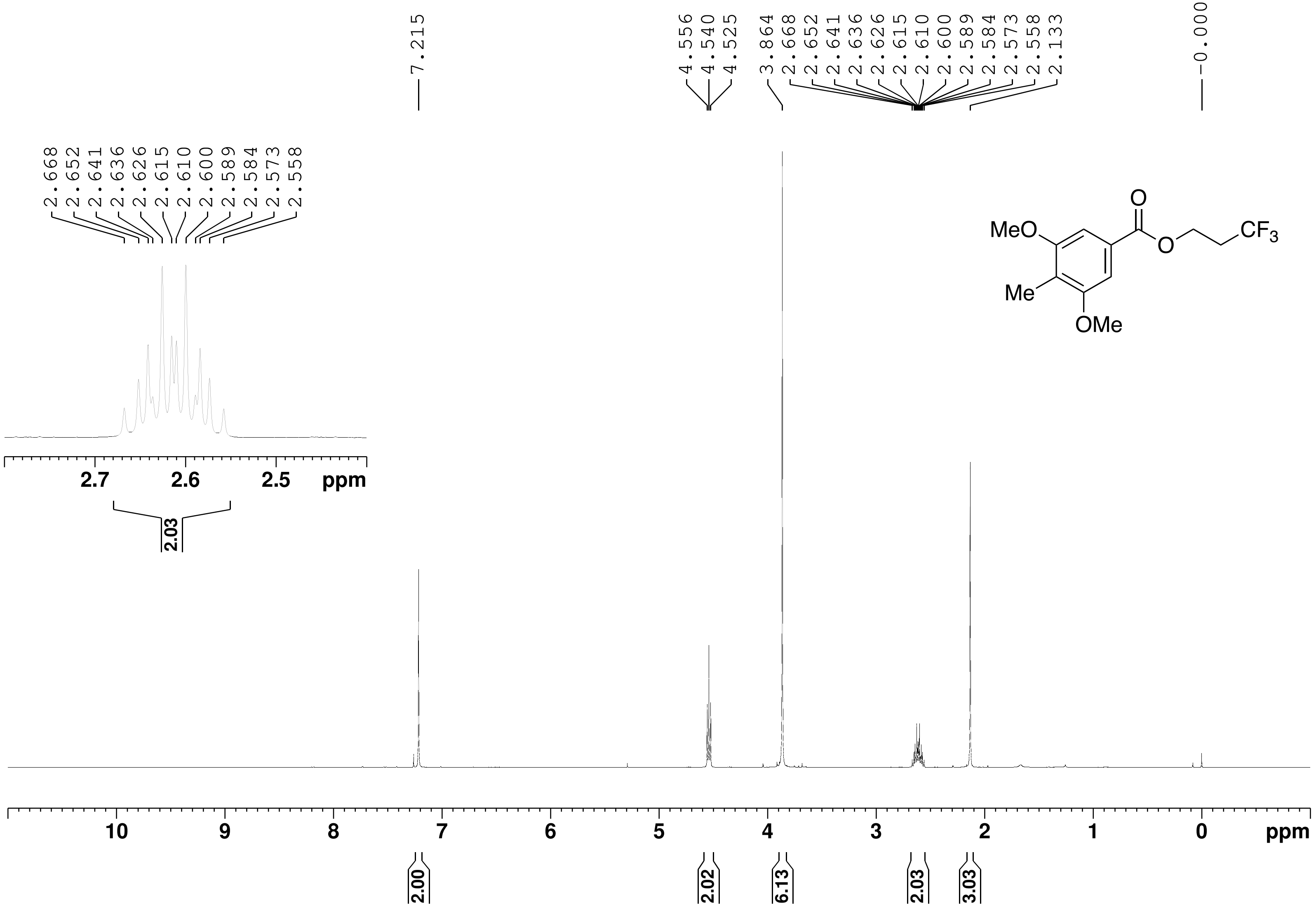

^{13}C NMR of **3ab** (100.6 MHz, $CDCl_3$)

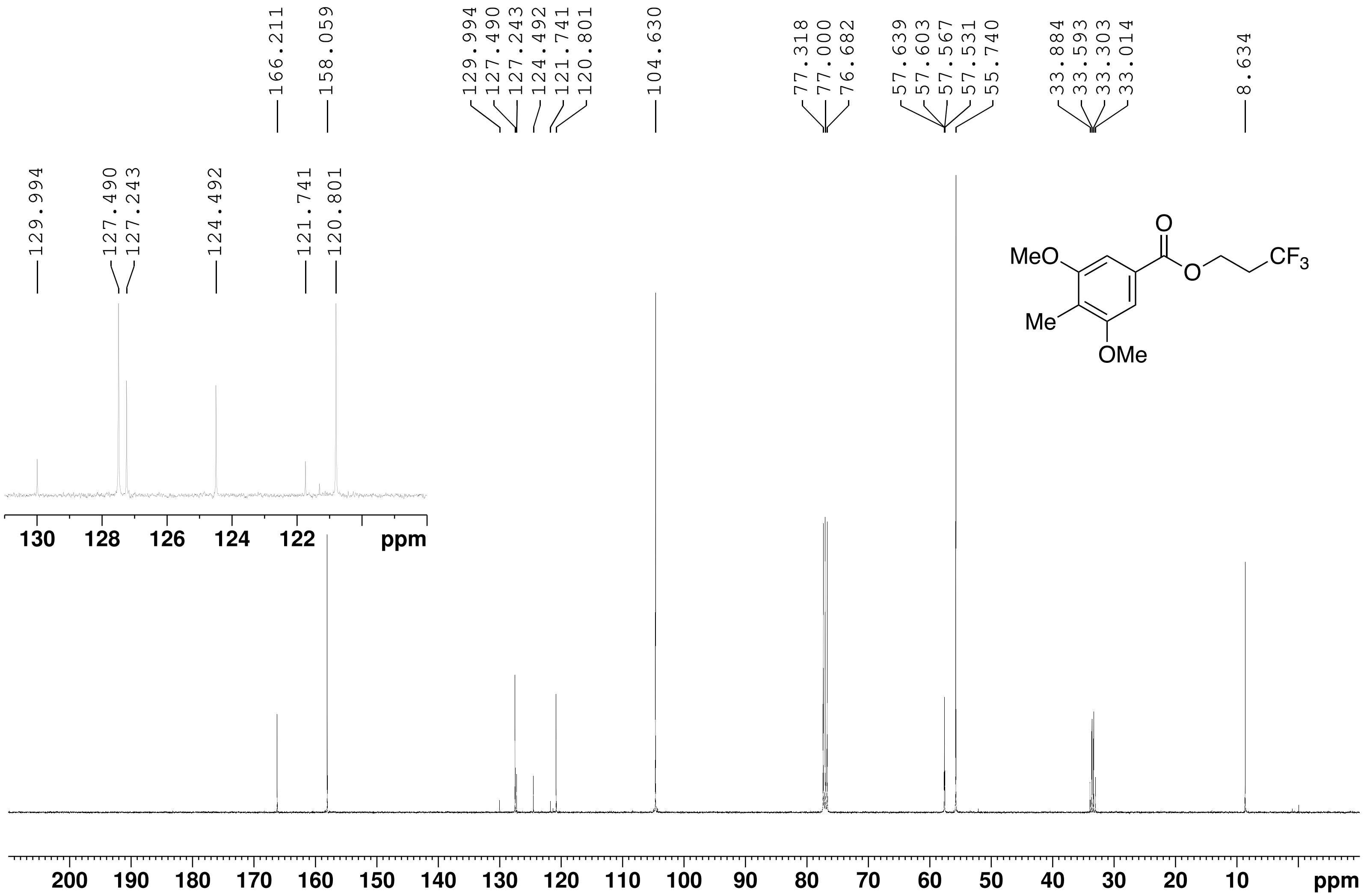

^{19}F NMR of **3ab** (376.5 MHz, $CDCl_3$)

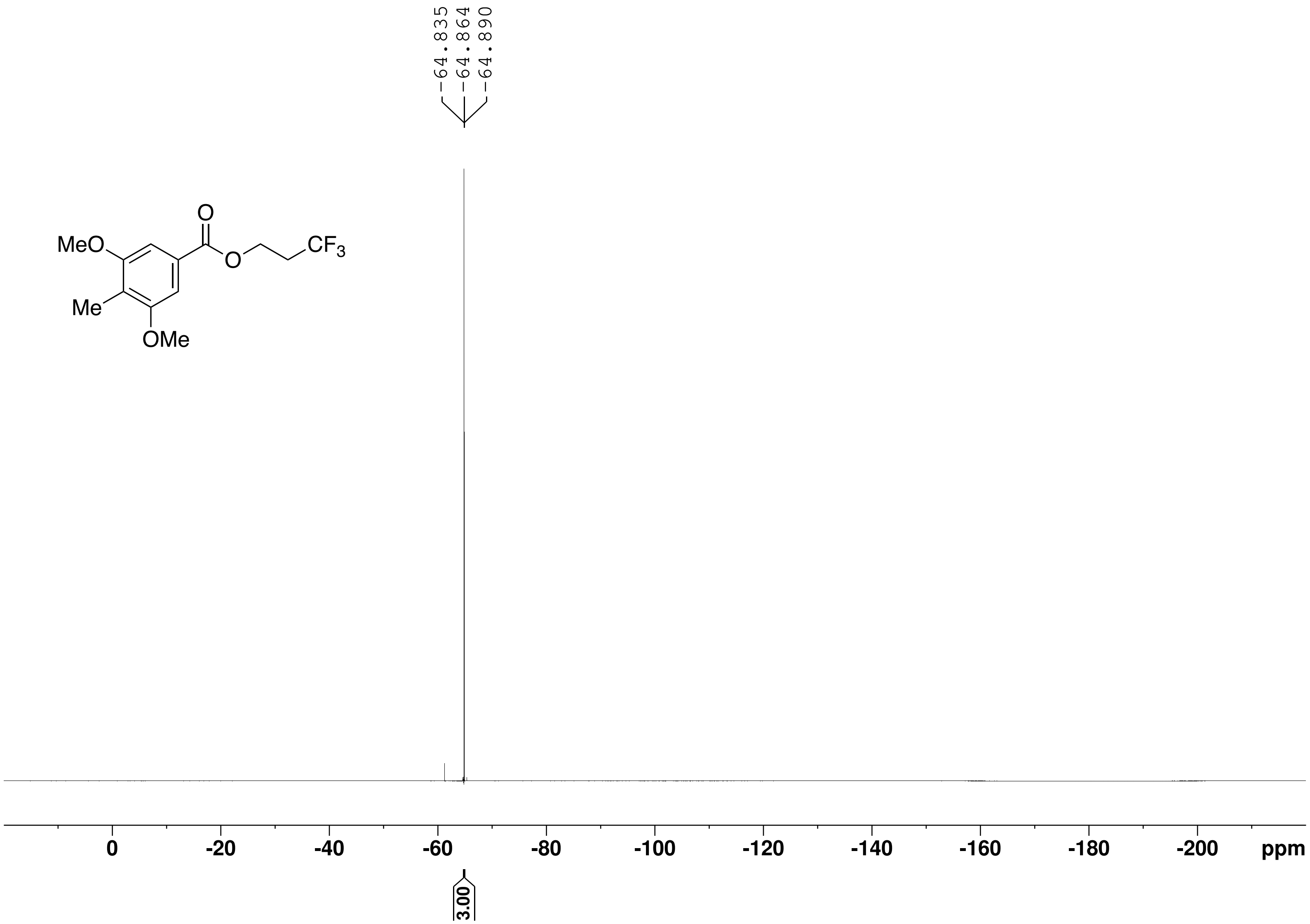

^{1}H NMR of **3ac** (400 MHz, $CDCl_3$)

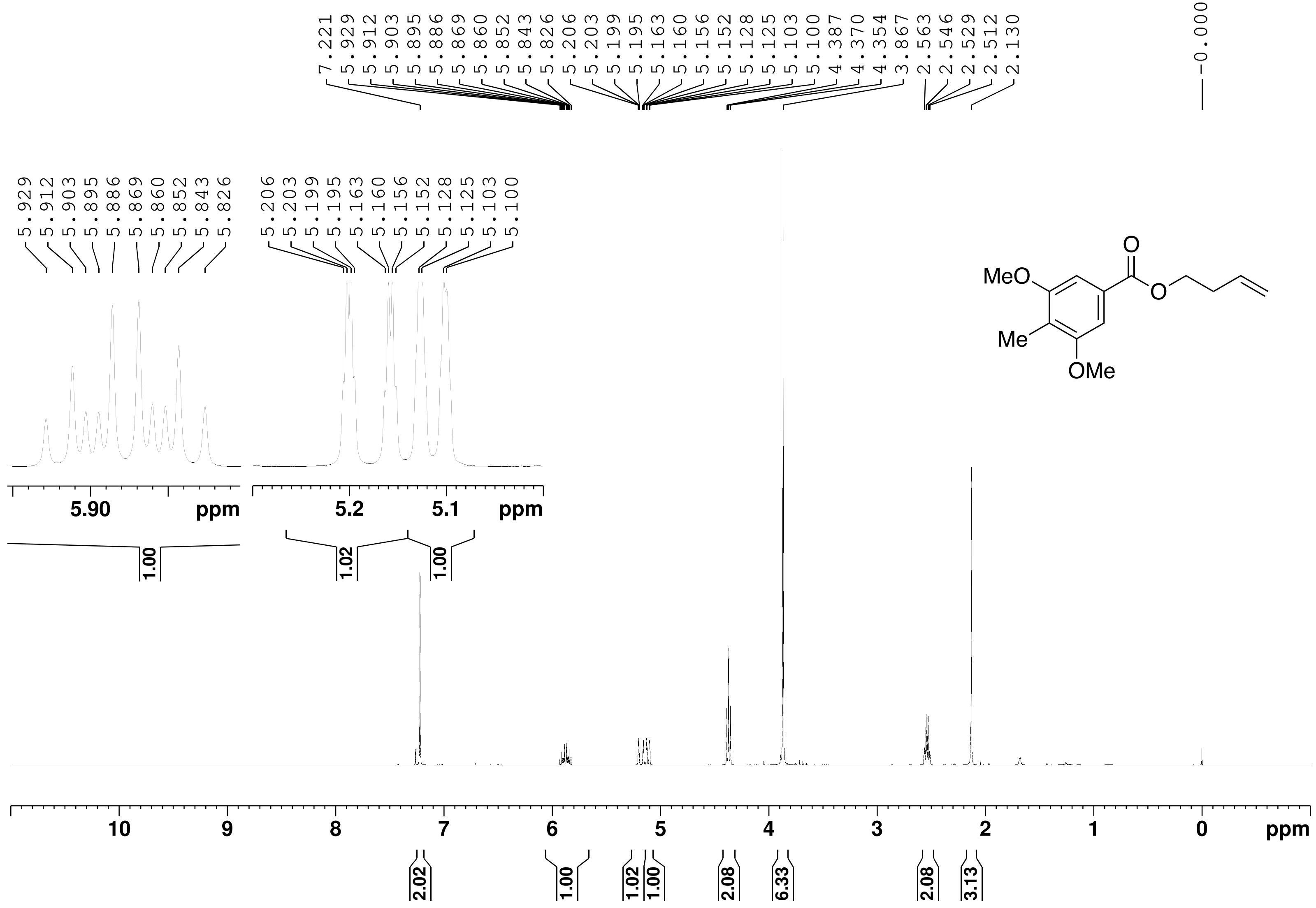

^{13}C NMR of **3ac** (100.6 MHz, $CDCl_3$)

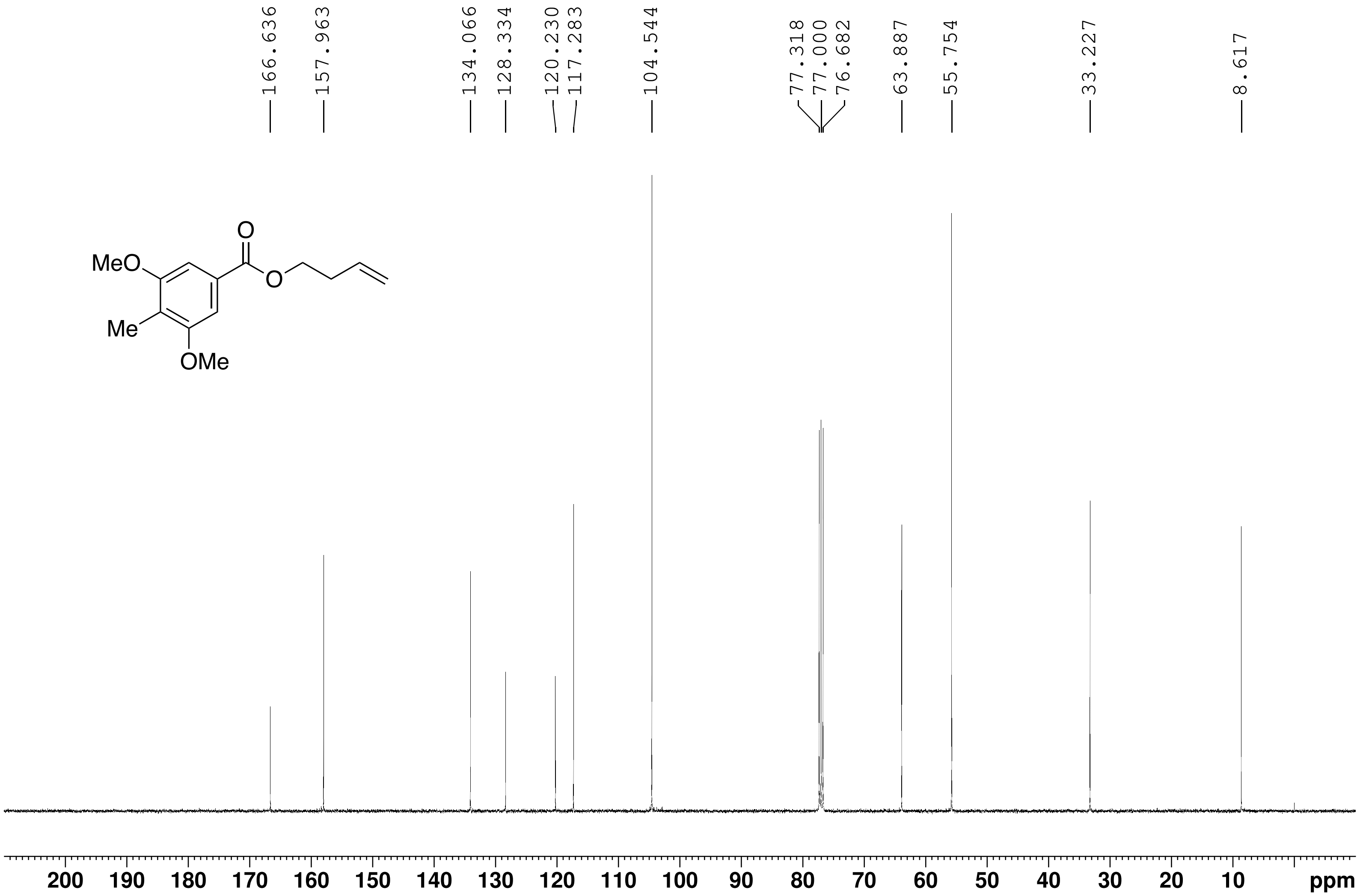

^{1}H NMR of **3ad** (400 MHz, $CDCl_3$)

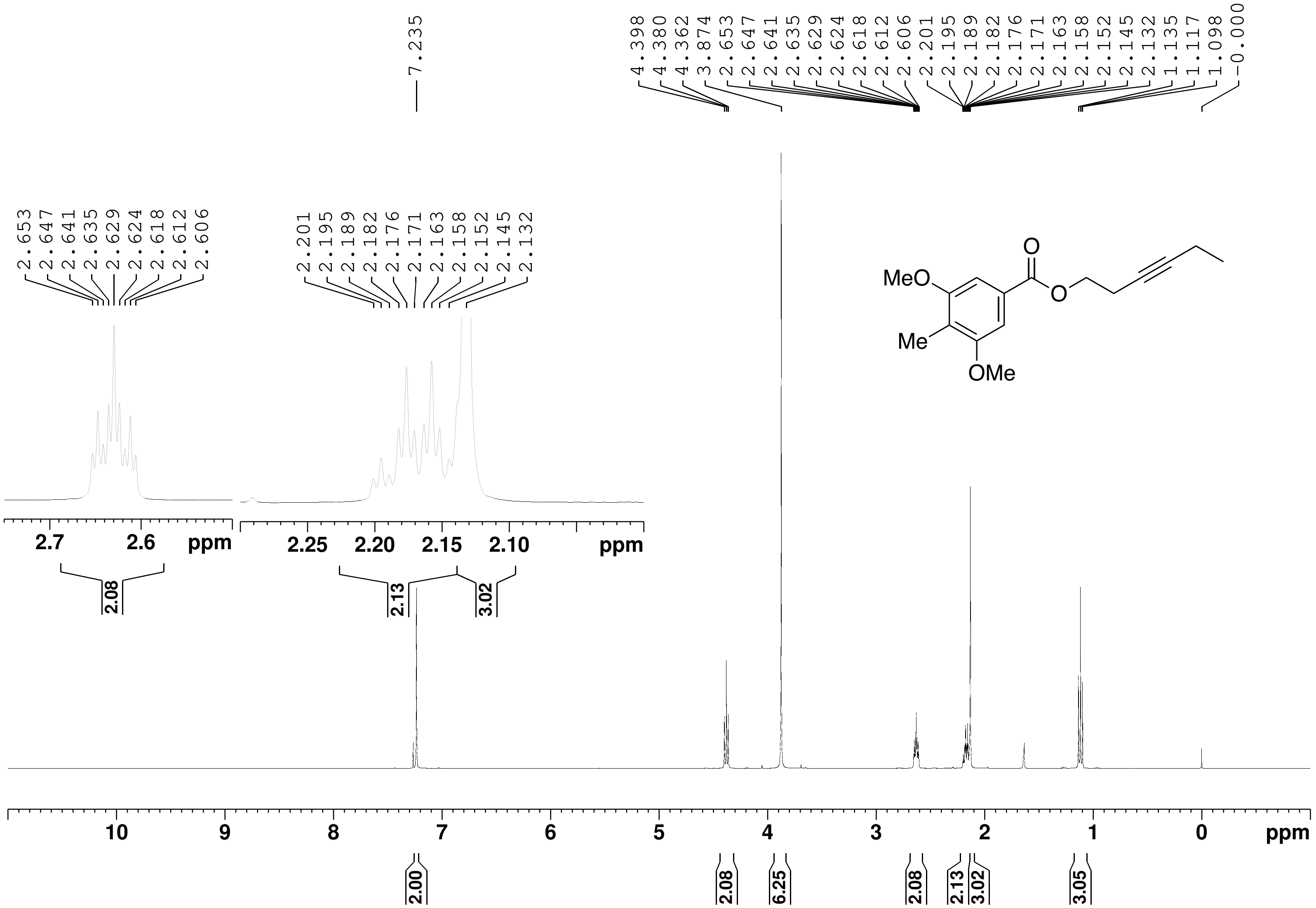

^{13}C NMR of **3ad** (100.6 MHz, $CDCl_3$)

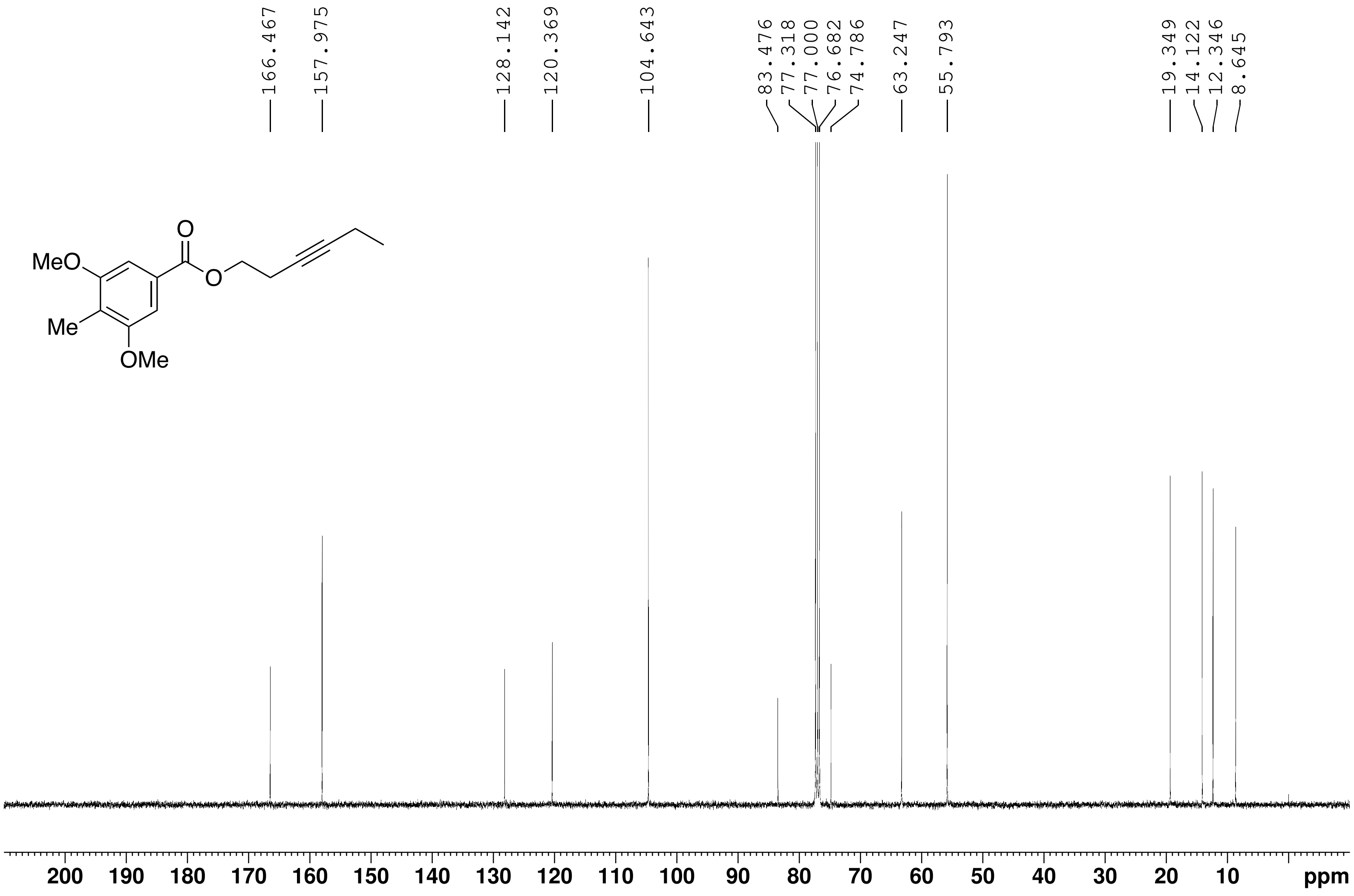

^{1}H NMR of **3ae** (400 MHz, $CDCl_3$)

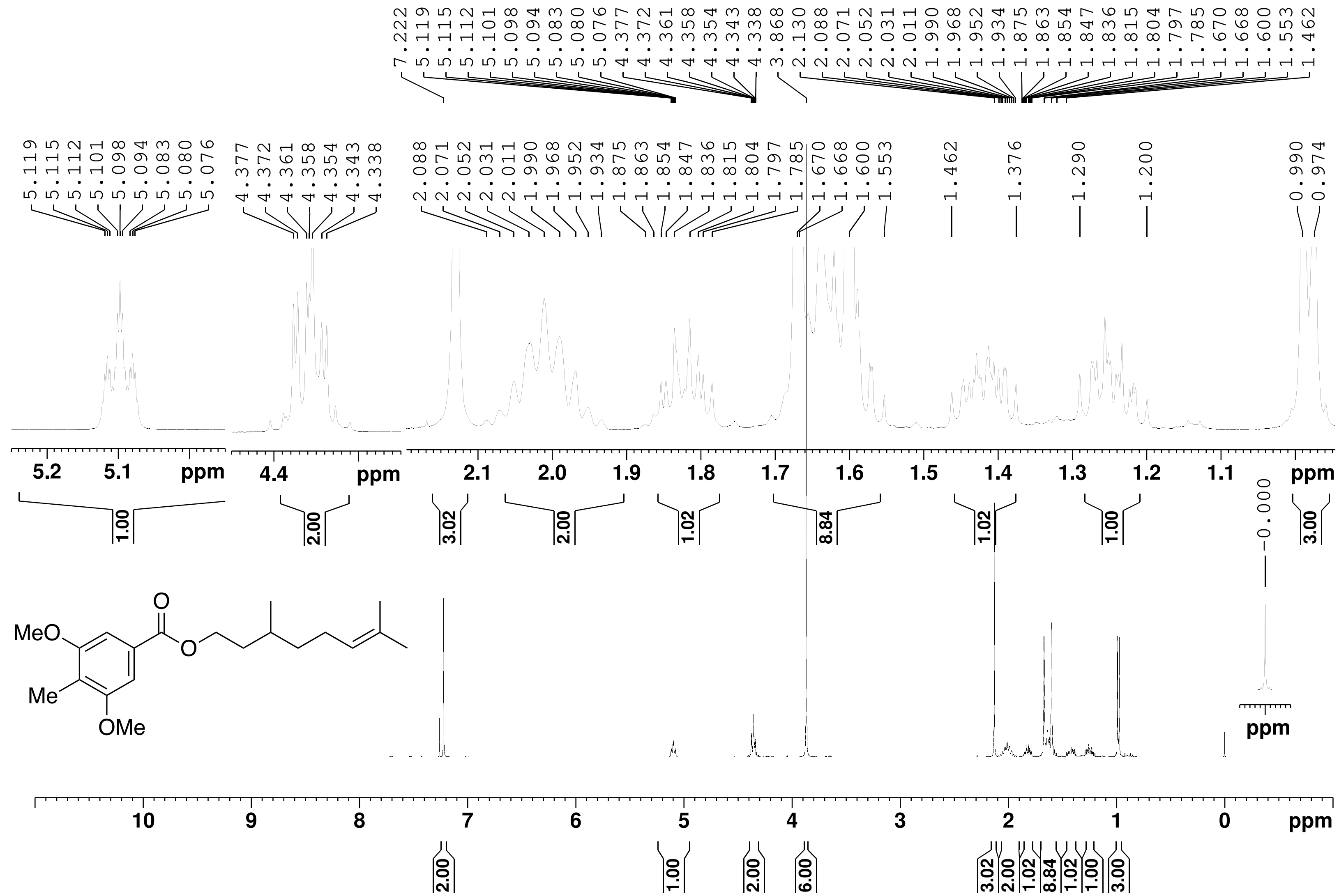

^{13}C NMR of **3ae** (100.6 MHz, $CDCl_3$)

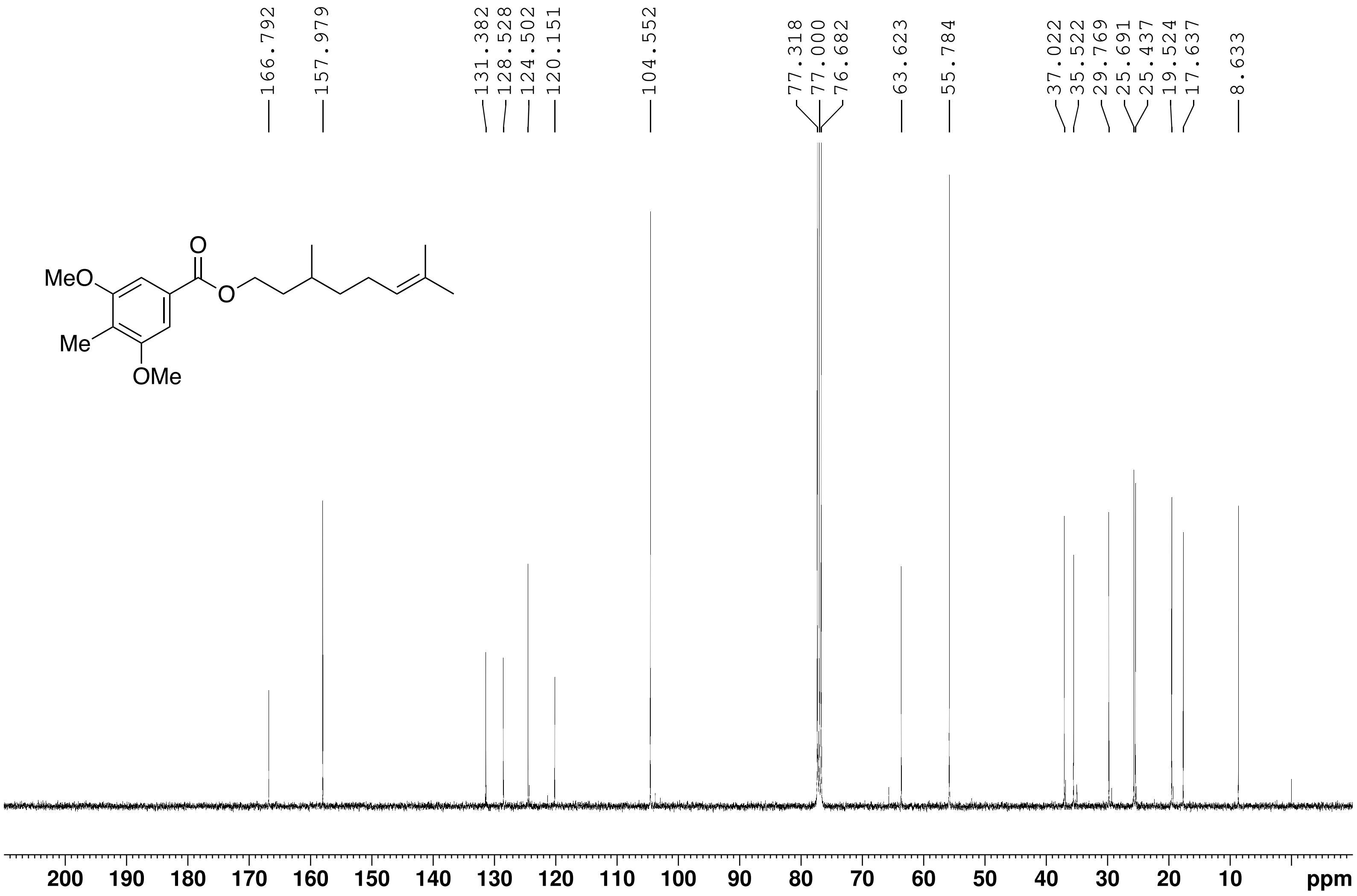

^{1}H NMR of **3af** (400 MHz, $CDCl_3$)

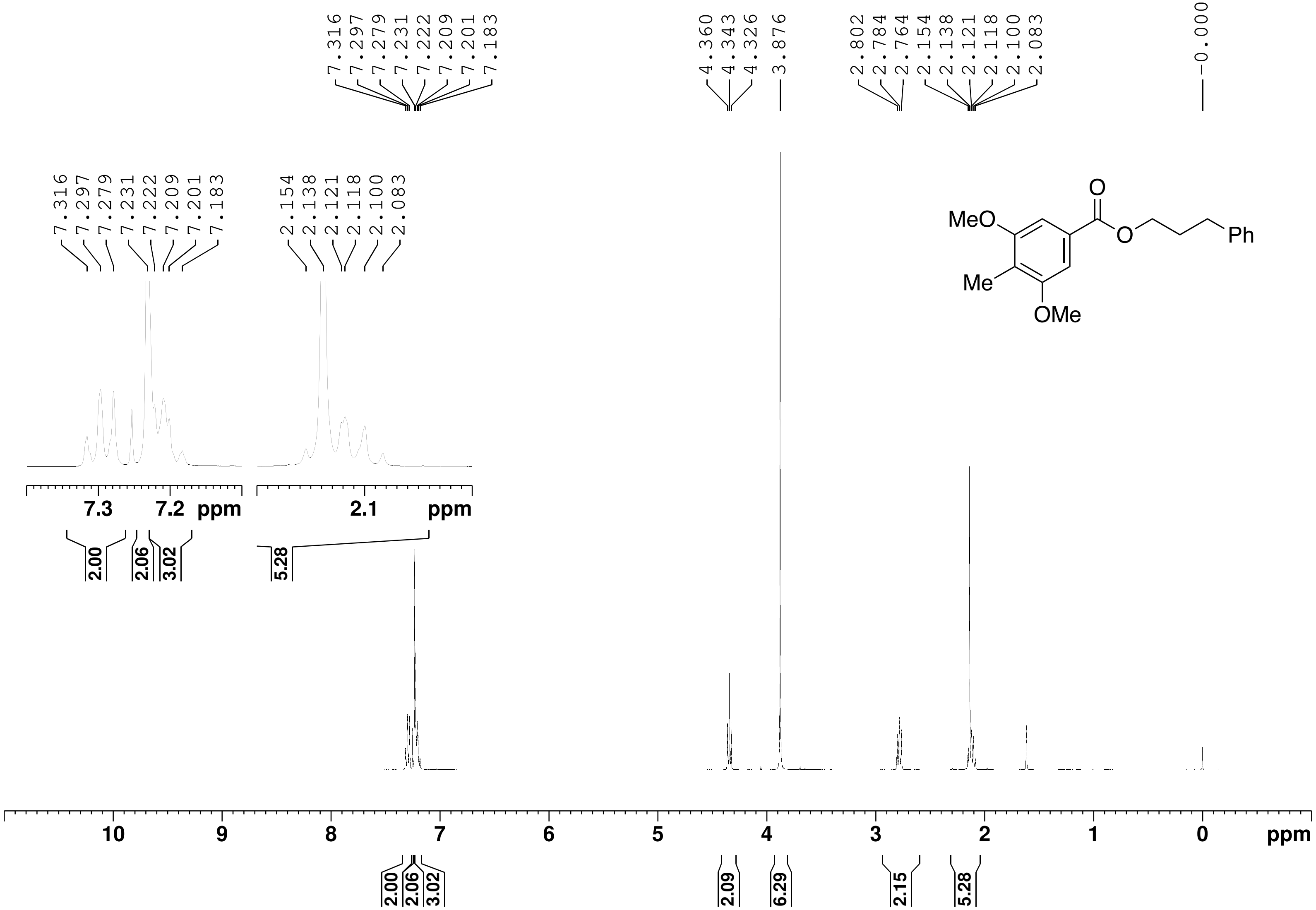

^{13}C NMR of **3af** (100.6 MHz, $CDCl_3$)

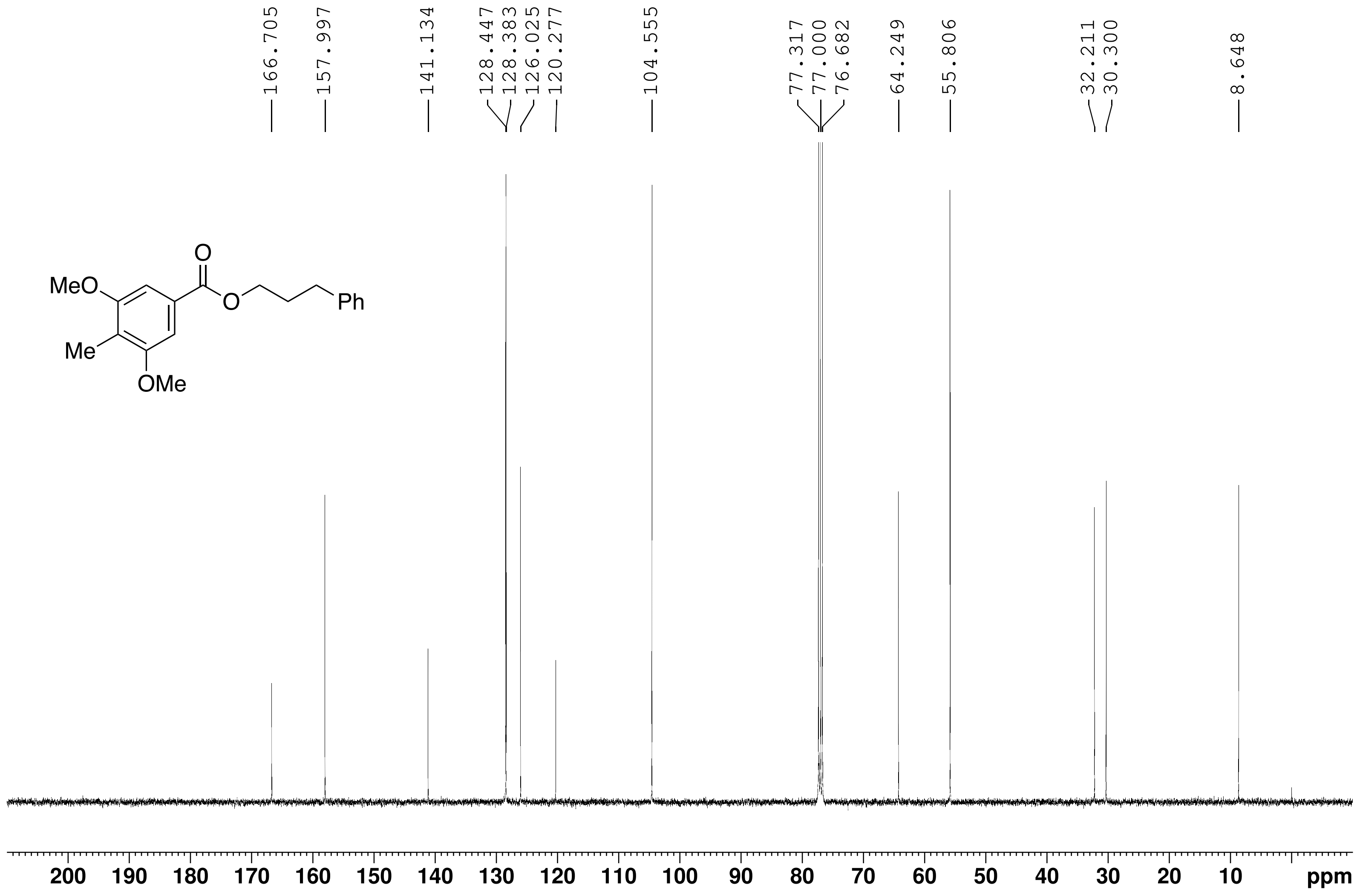

^{1}H NMR of **3ag** (400 MHz, $CDCl_3$)

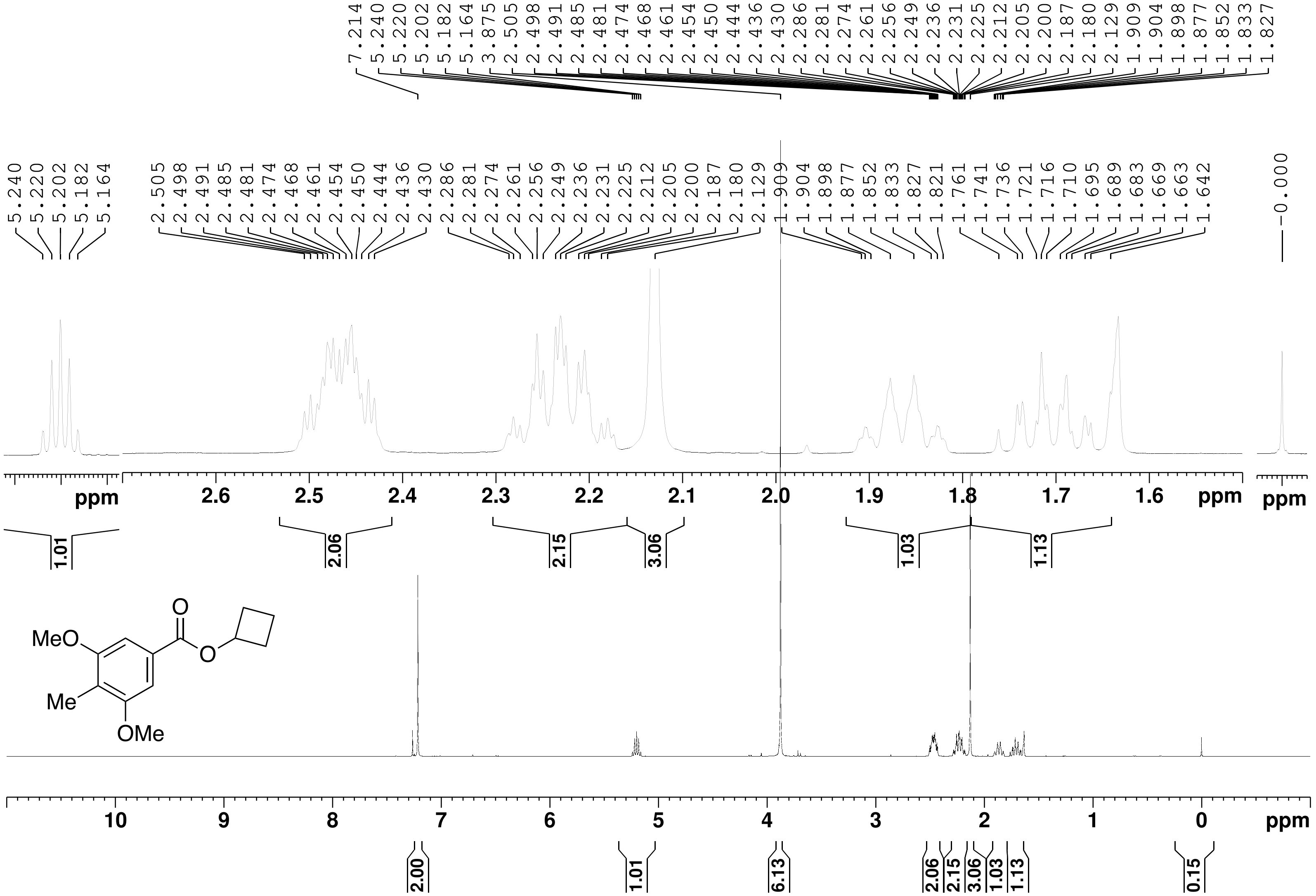

^{13}C NMR of **3ag** (100.6 MHz, $CDCl_3$)

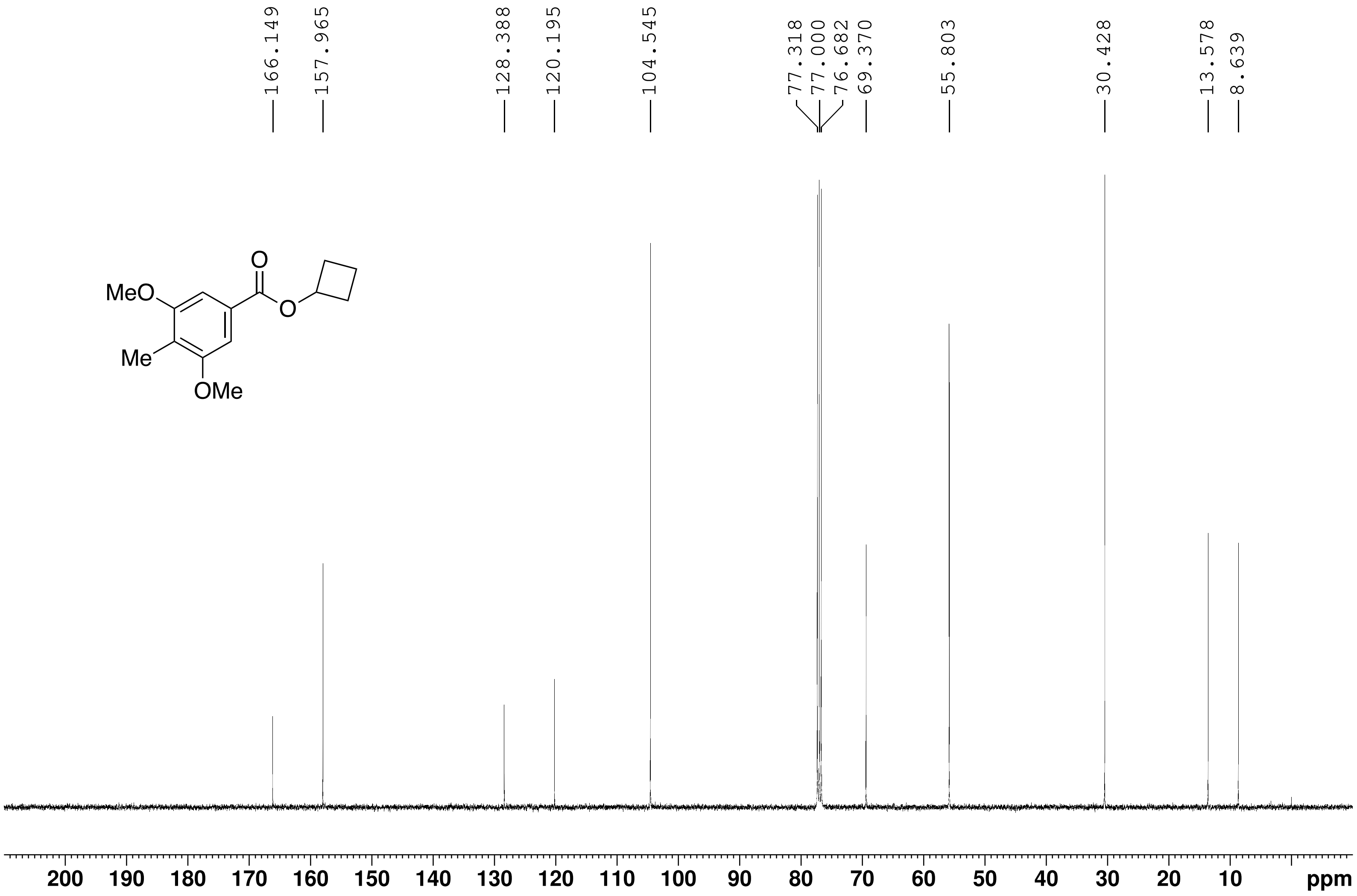

^{1}H NMR of **5aa** (400 MHz, $CDCl_3$)

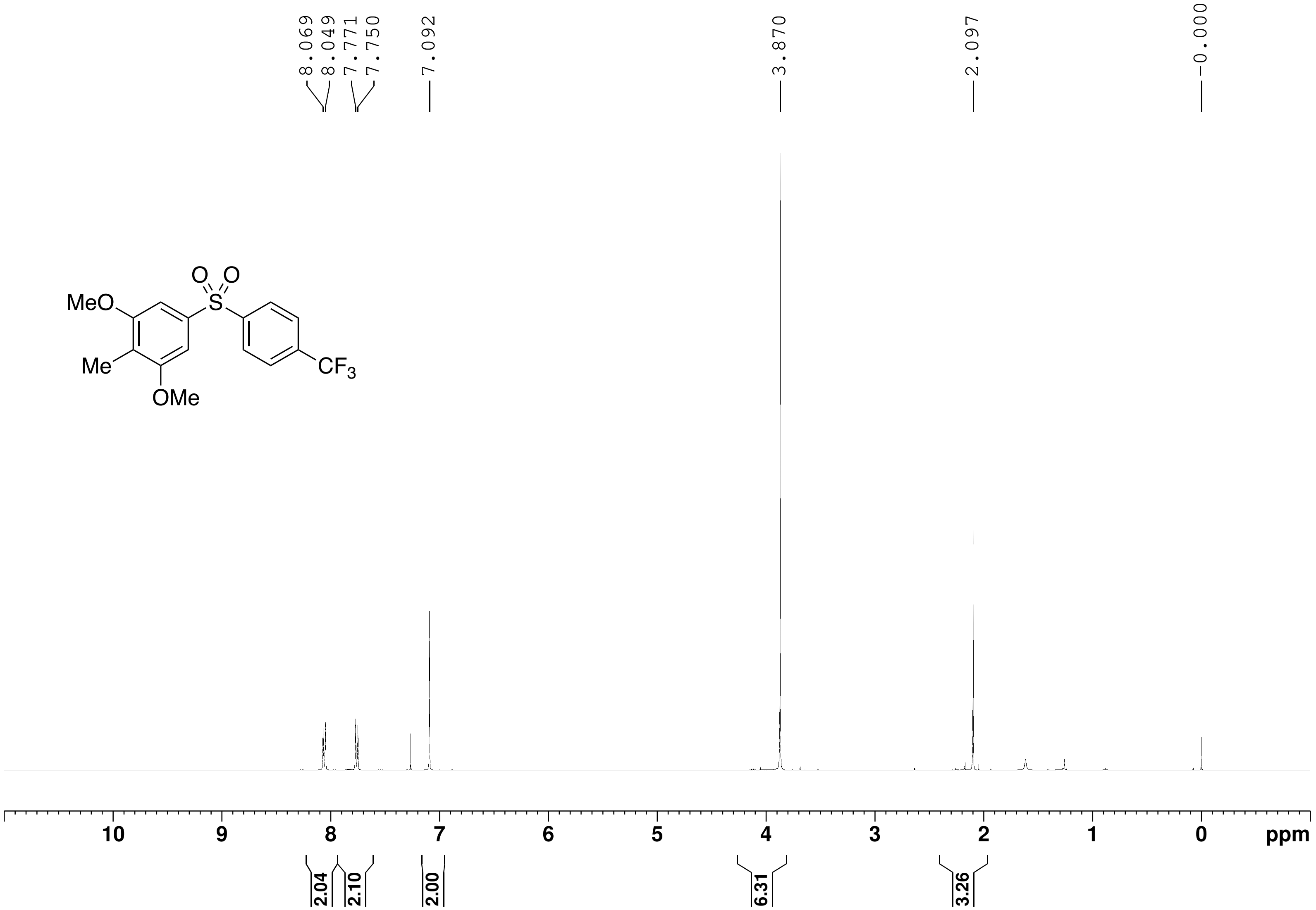

^{13}C NMR of **5aa** (100.6 MHz, CDCl$_3$)

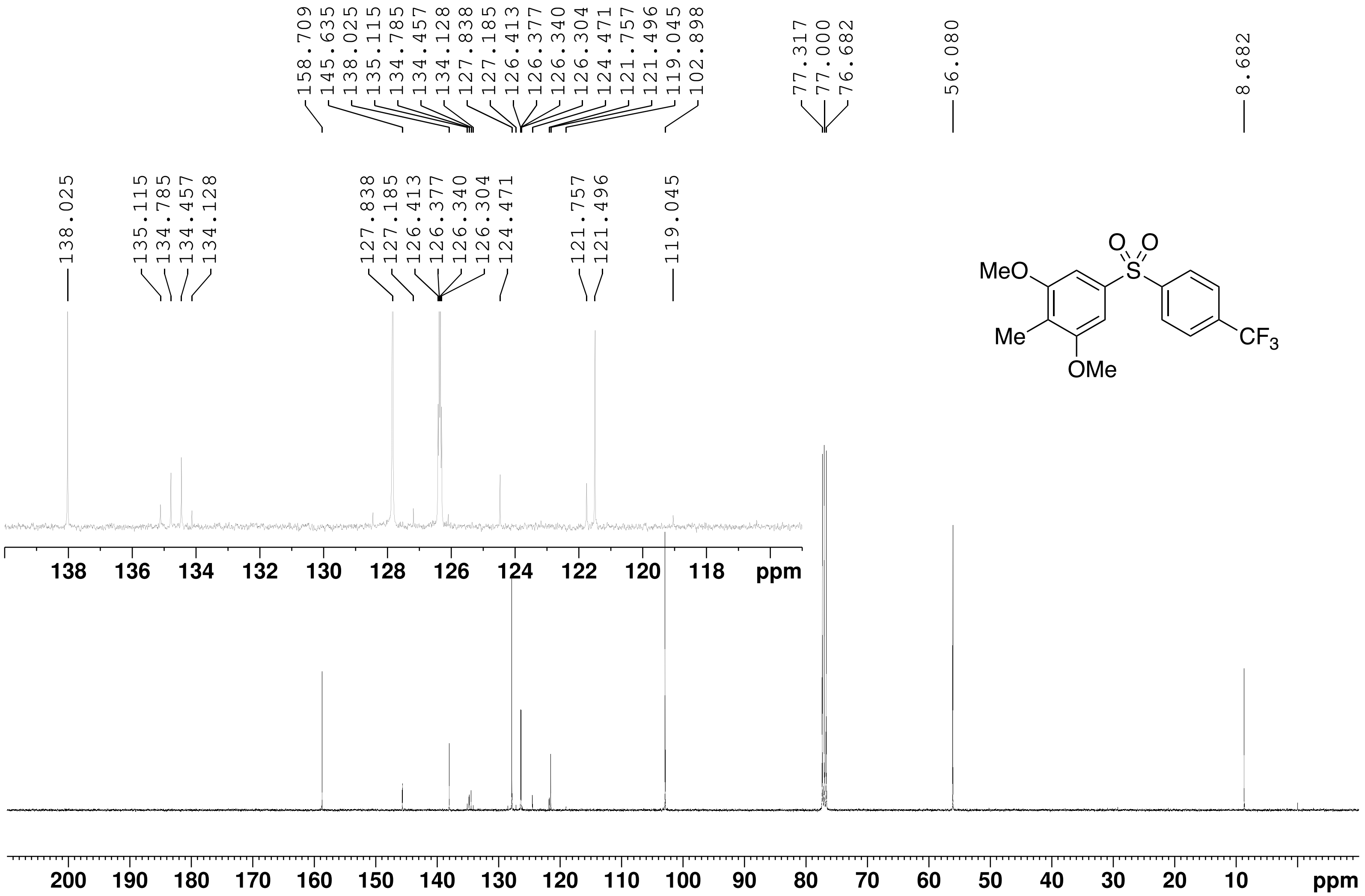

^{19}F NMR of **5aa** (376.5 MHz, $CDCl_3$)

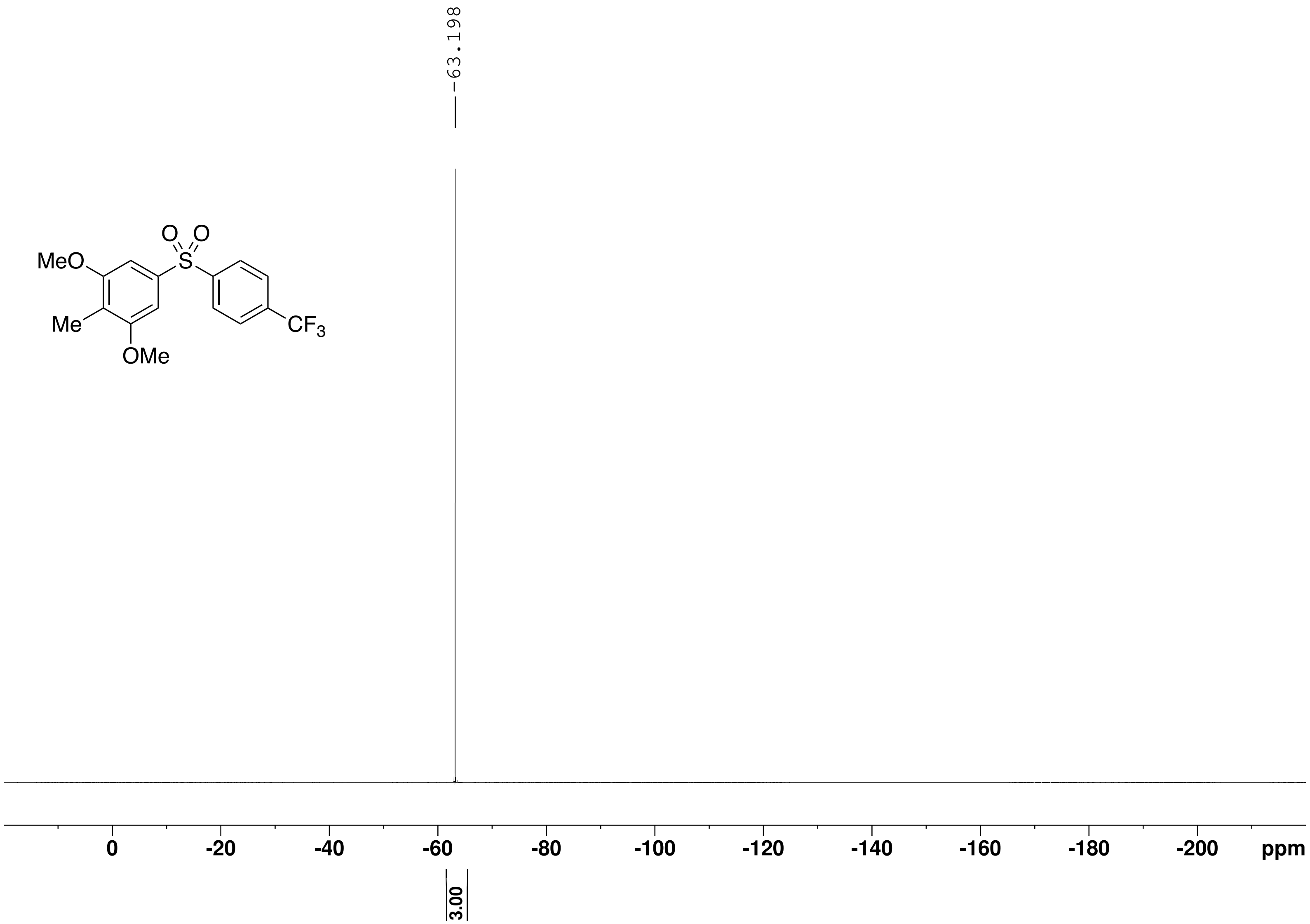

^{1}H NMR of **5ab** (400 MHz, $CDCl_3$)

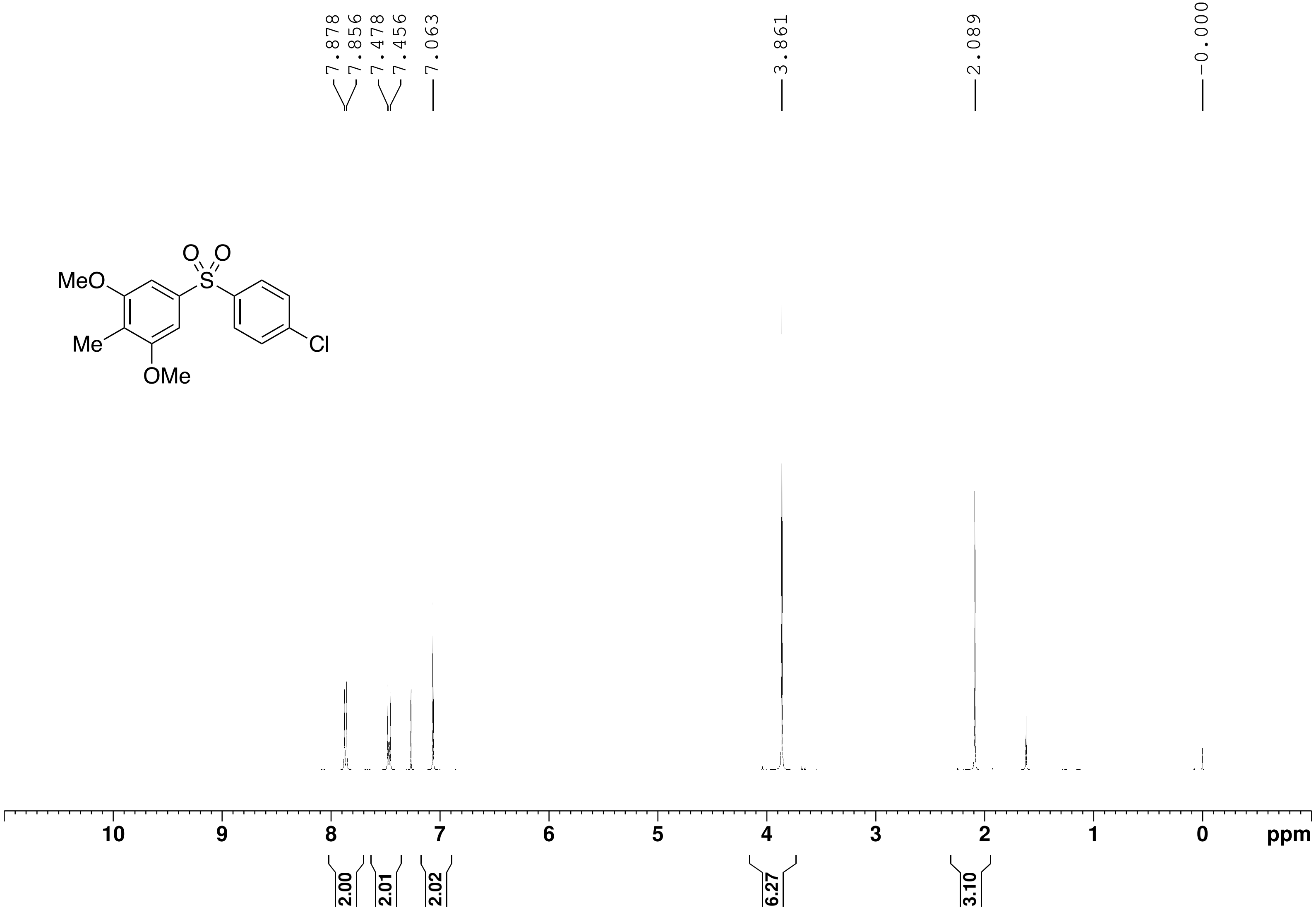

^{13}C NMR of **5ab** (100.6 MHz, $CDCl_3$)

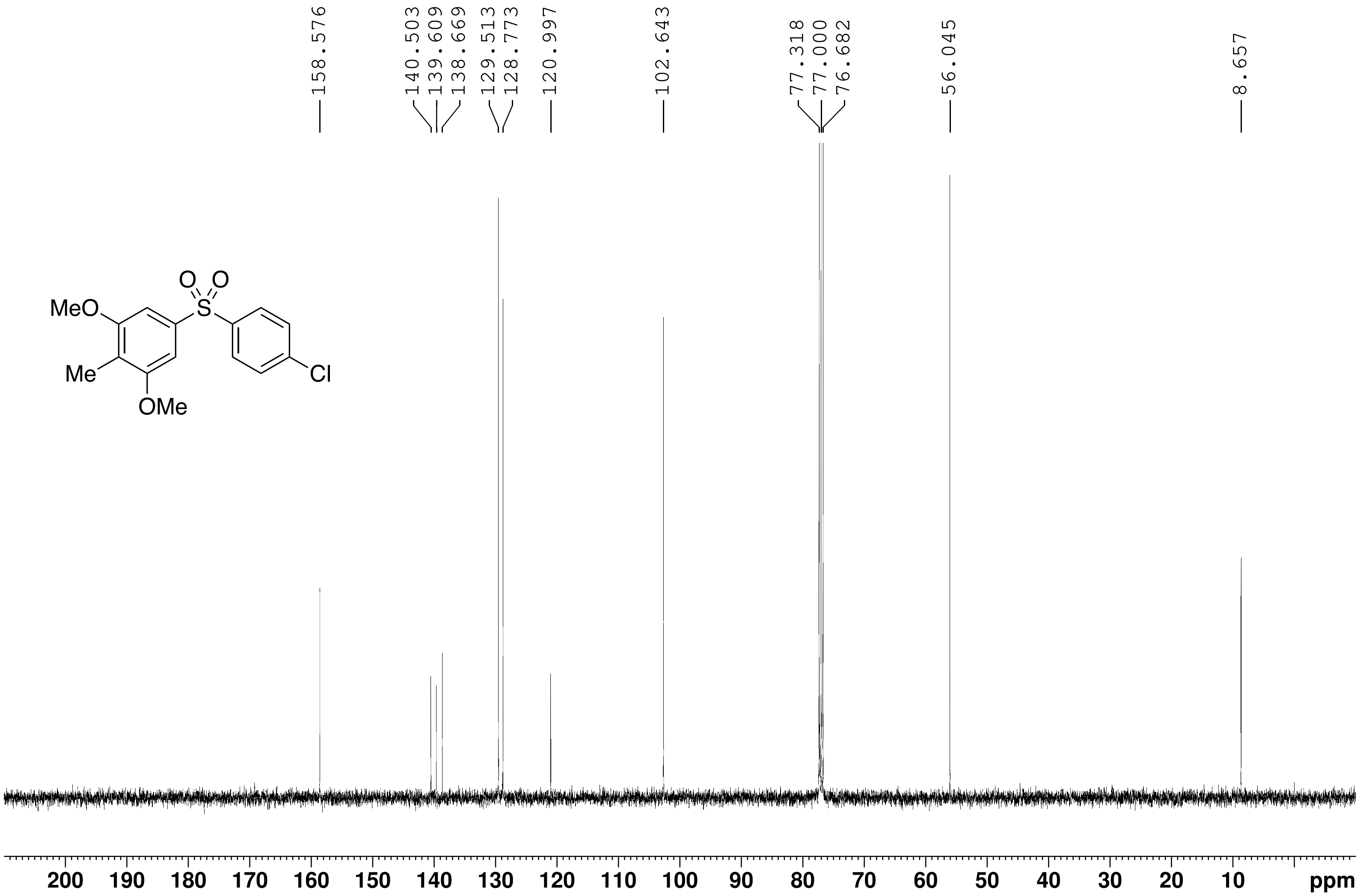

^{1}H NMR of **5ac** (400 MHz, $CDCl_3$)

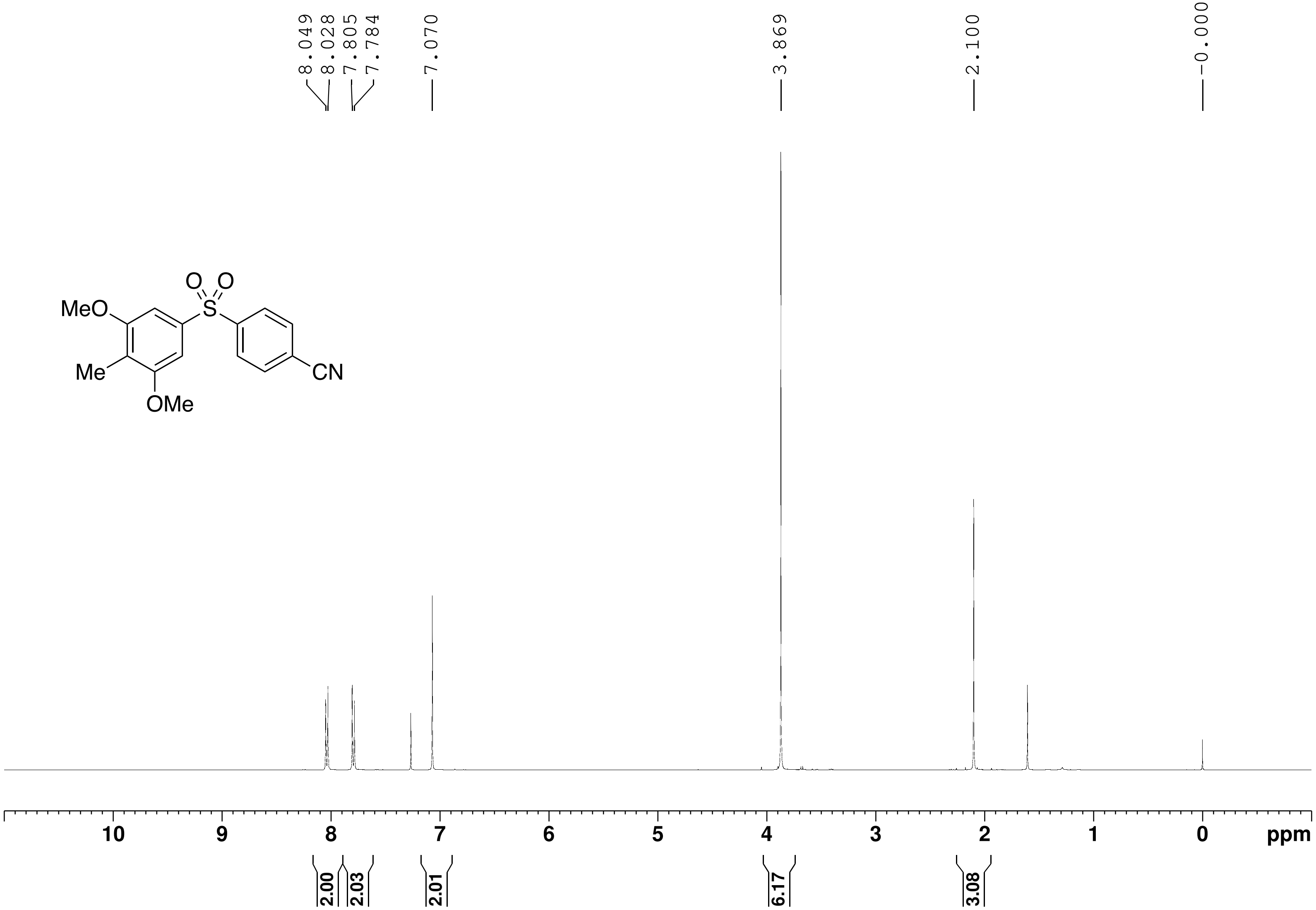

^{13}C NMR of **5ac** (100.6 MHz, $CDCl_3$)

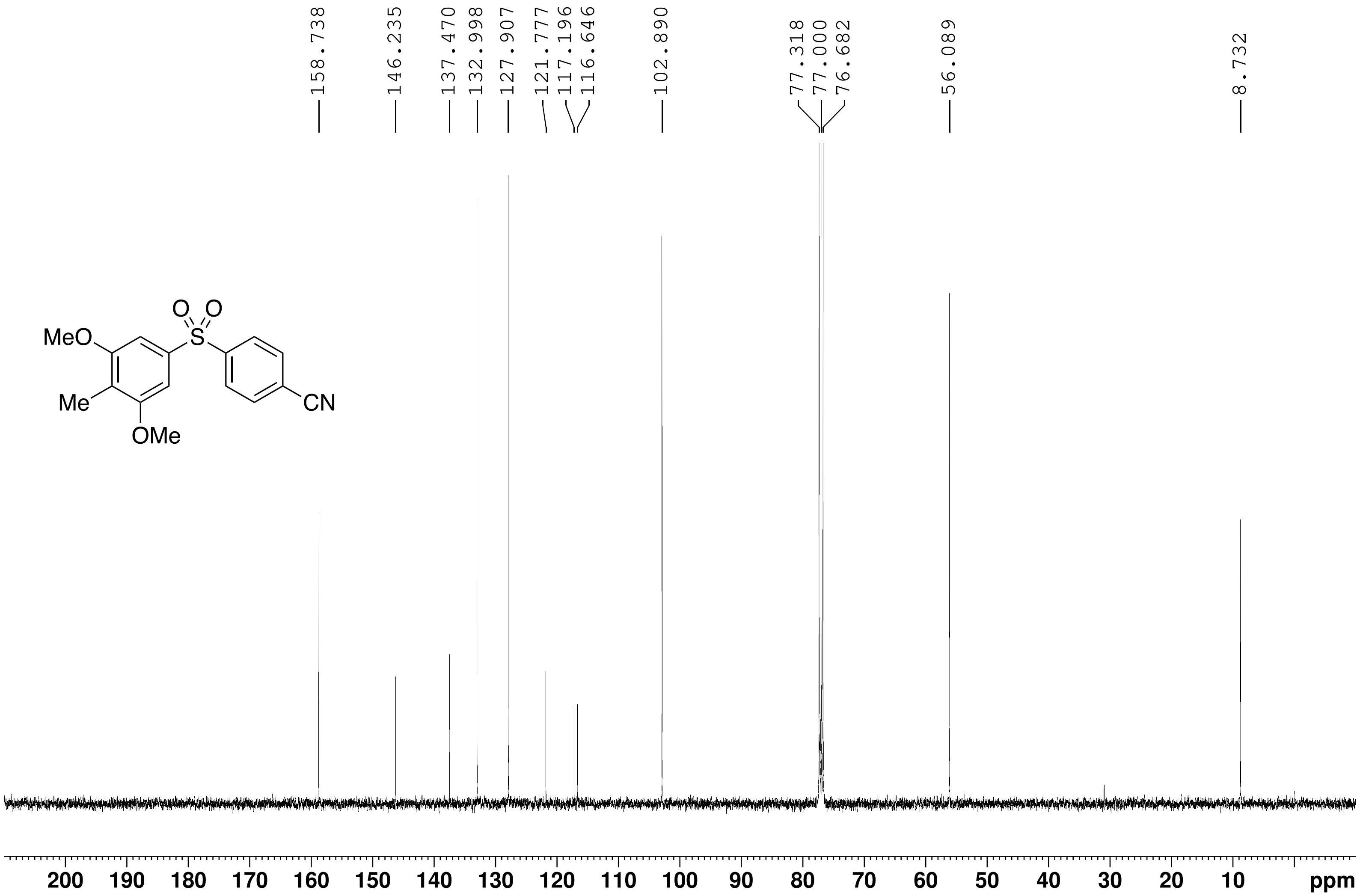

^{1}H NMR of **5ad** (400 MHz, $CDCl_3$)

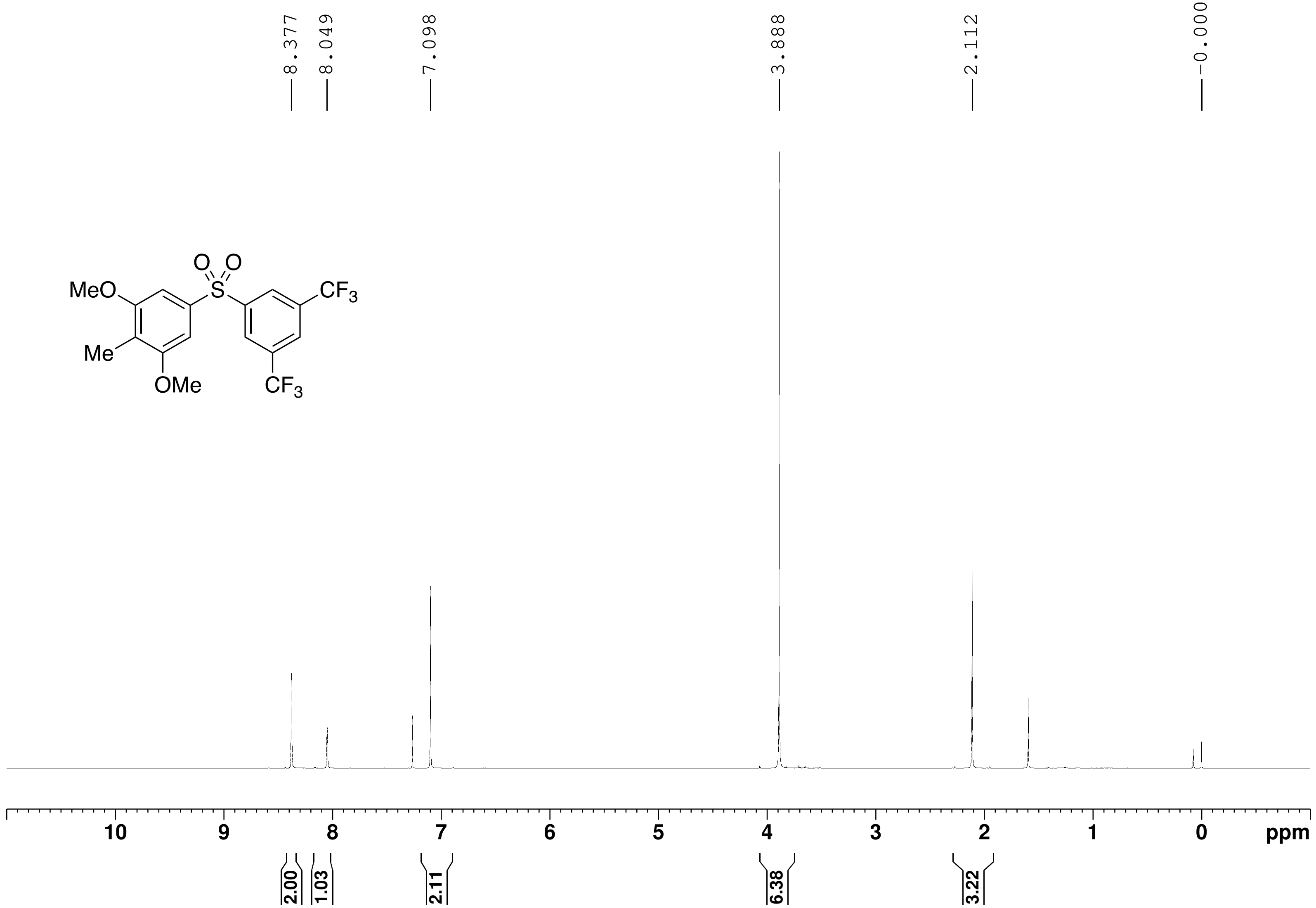

^{13}C NMR of **5ad** (100.6 MHz, CDCl$_3$)

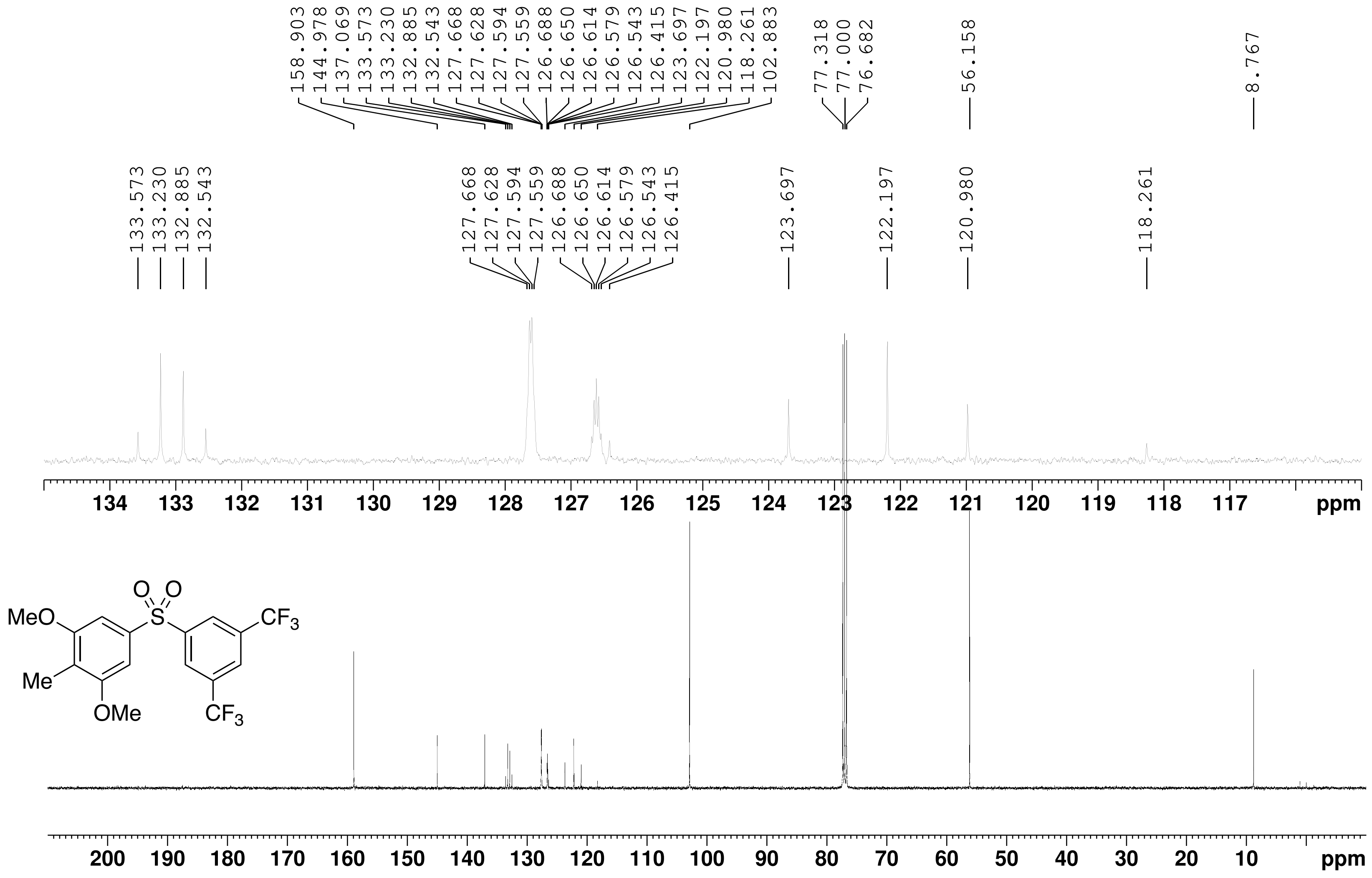

^{19}F NMR of **5ad** (376.5 MHz, $CDCl_3$)

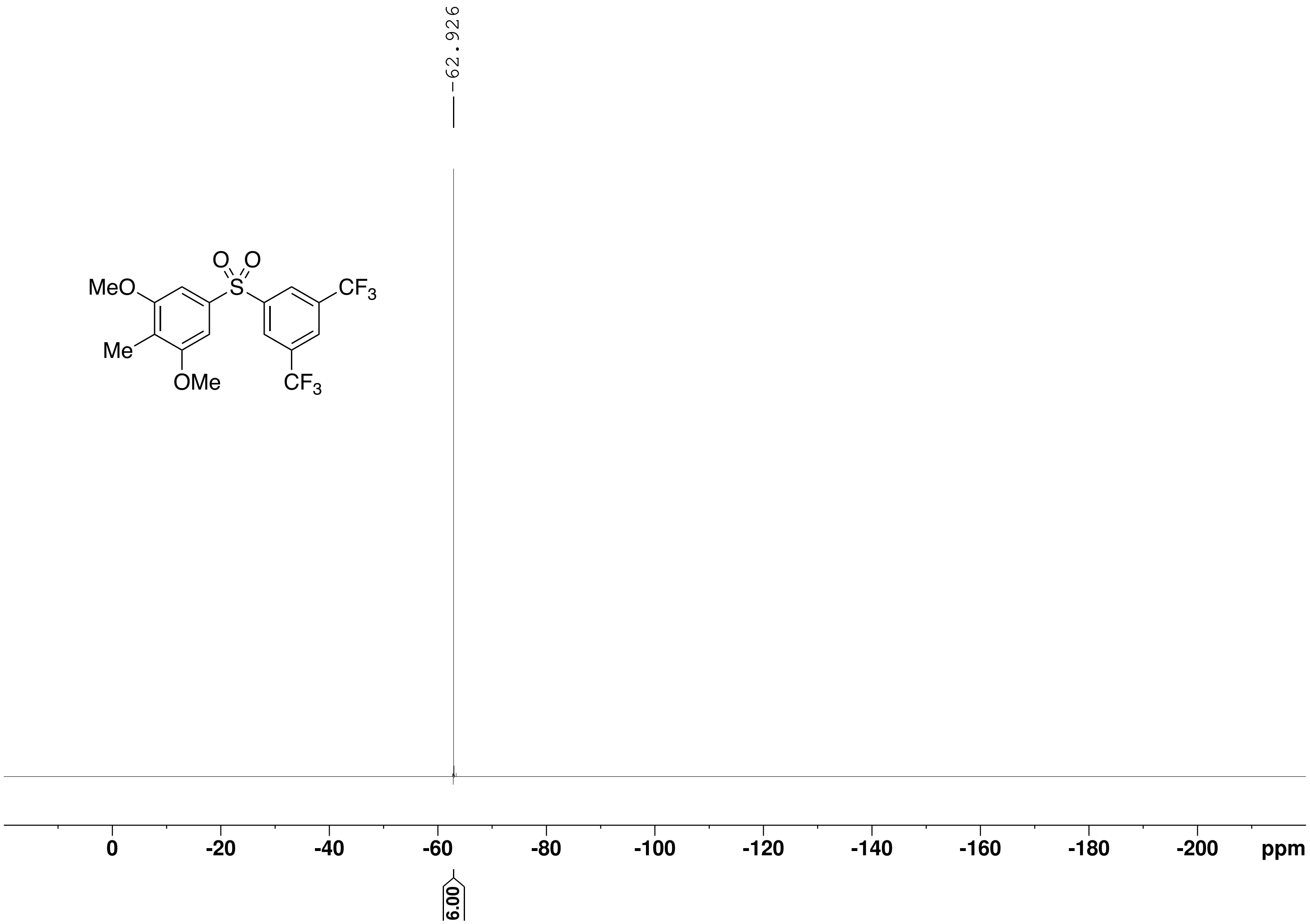

^{1}H NMR of **5ae** (400 MHz, $CDCl_3$)

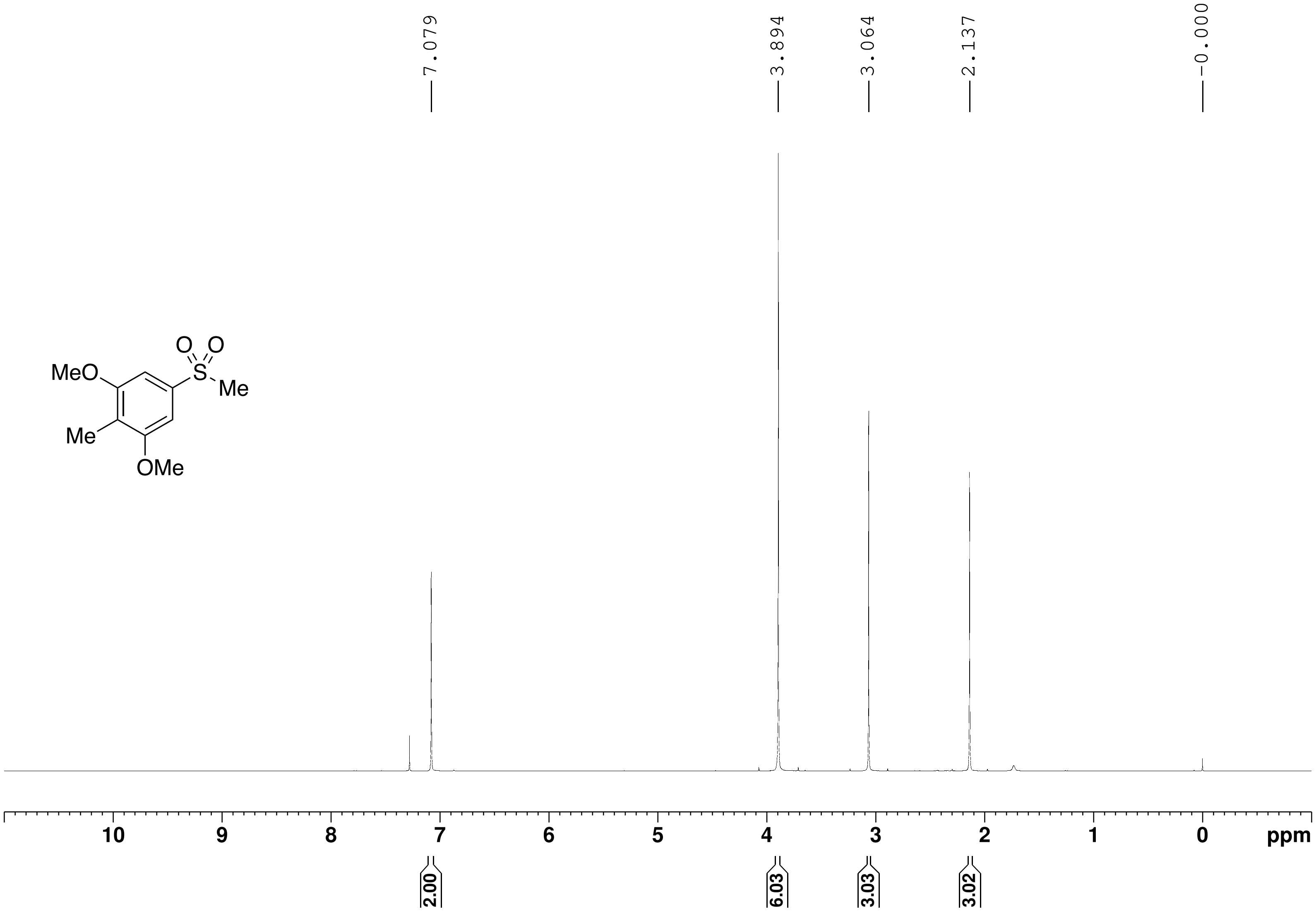

^{13}C NMR of **5ae** (100.6 MHz, $CDCl_3$)

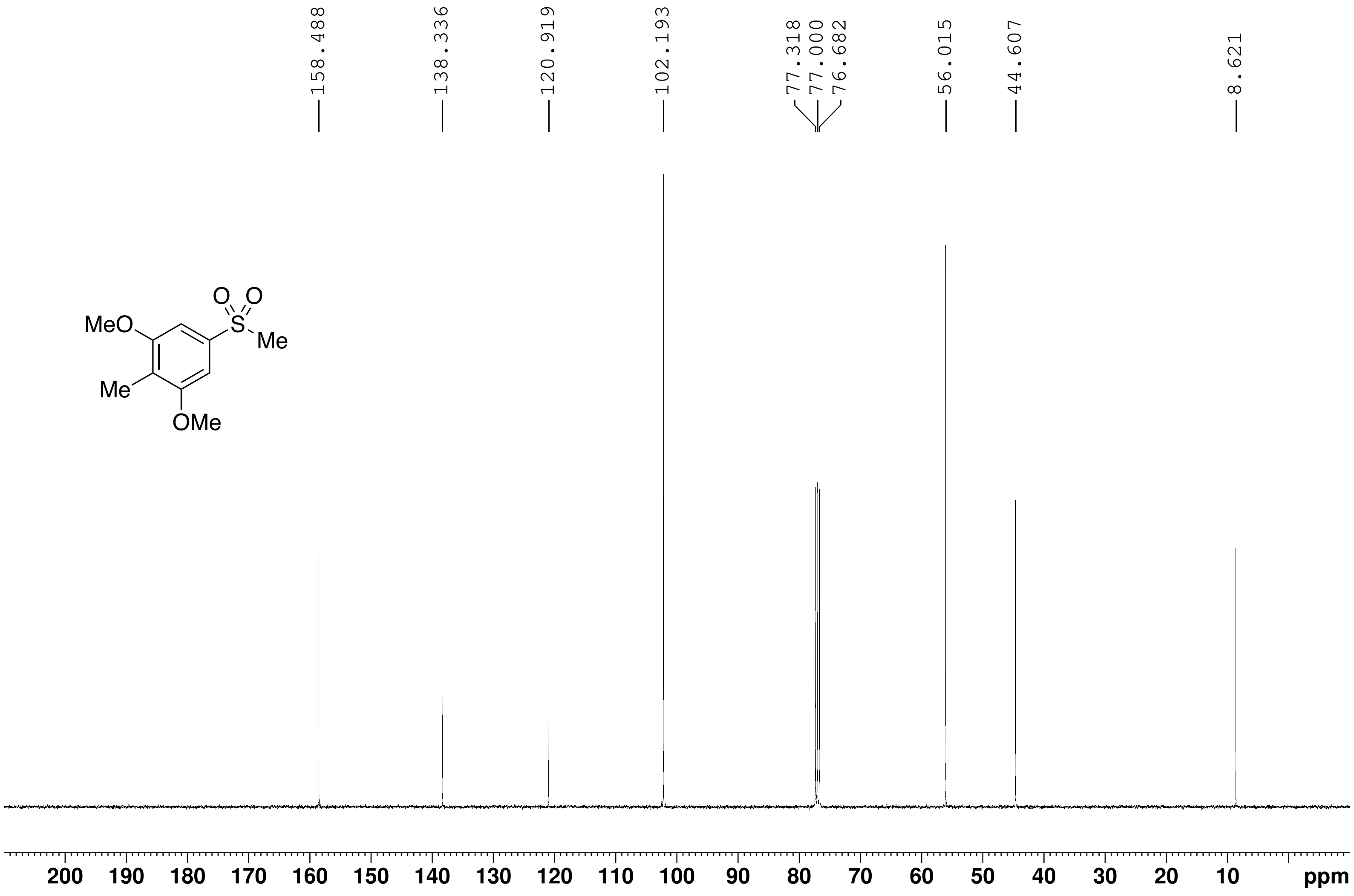

^{1}H NMR of **5af** (400 MHz, $CDCl_3$)

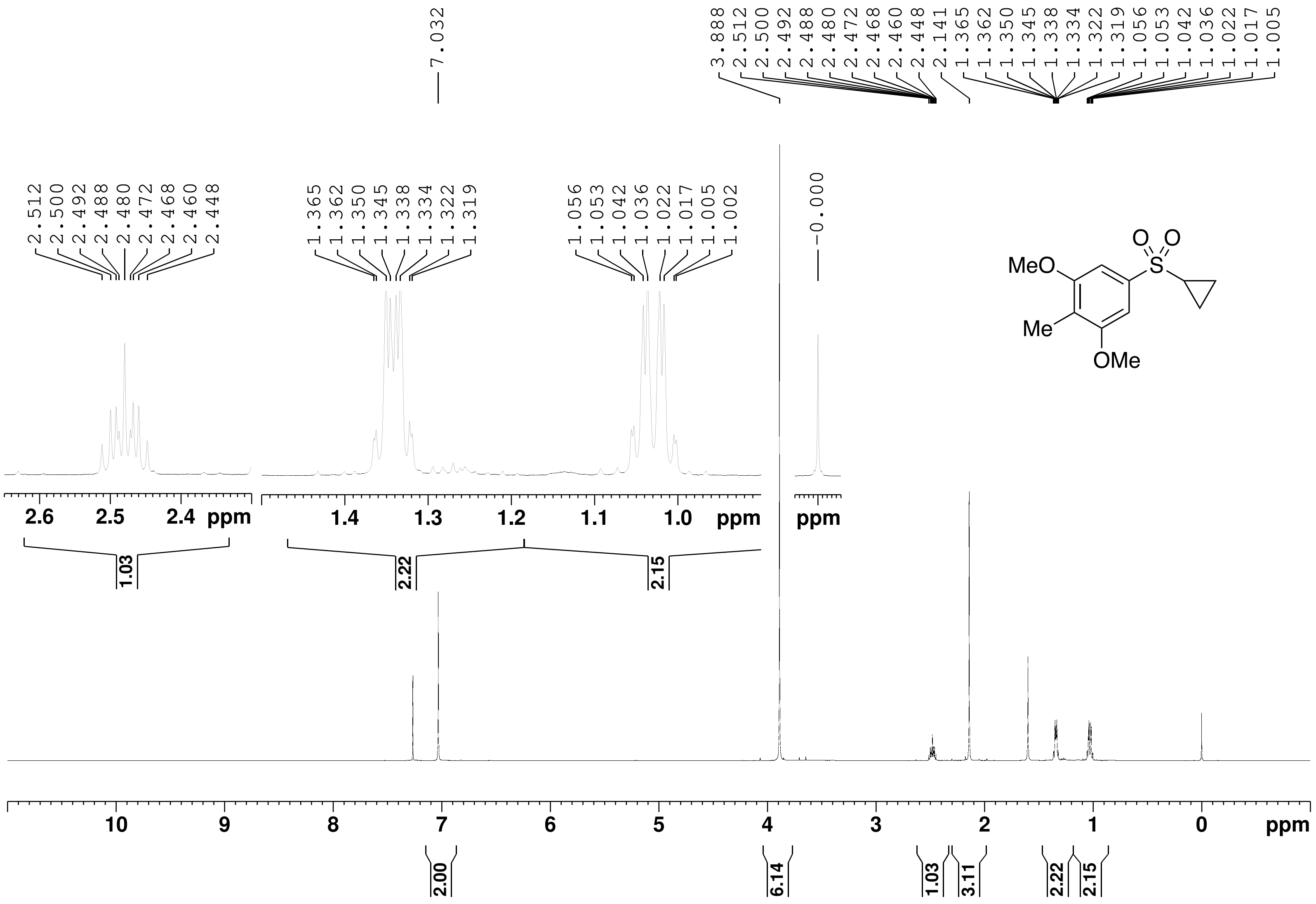

^{13}C NMR of **5af** (100.6 MHz, $CDCl_3$)

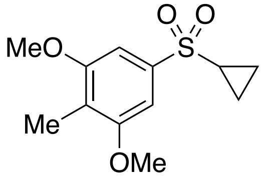

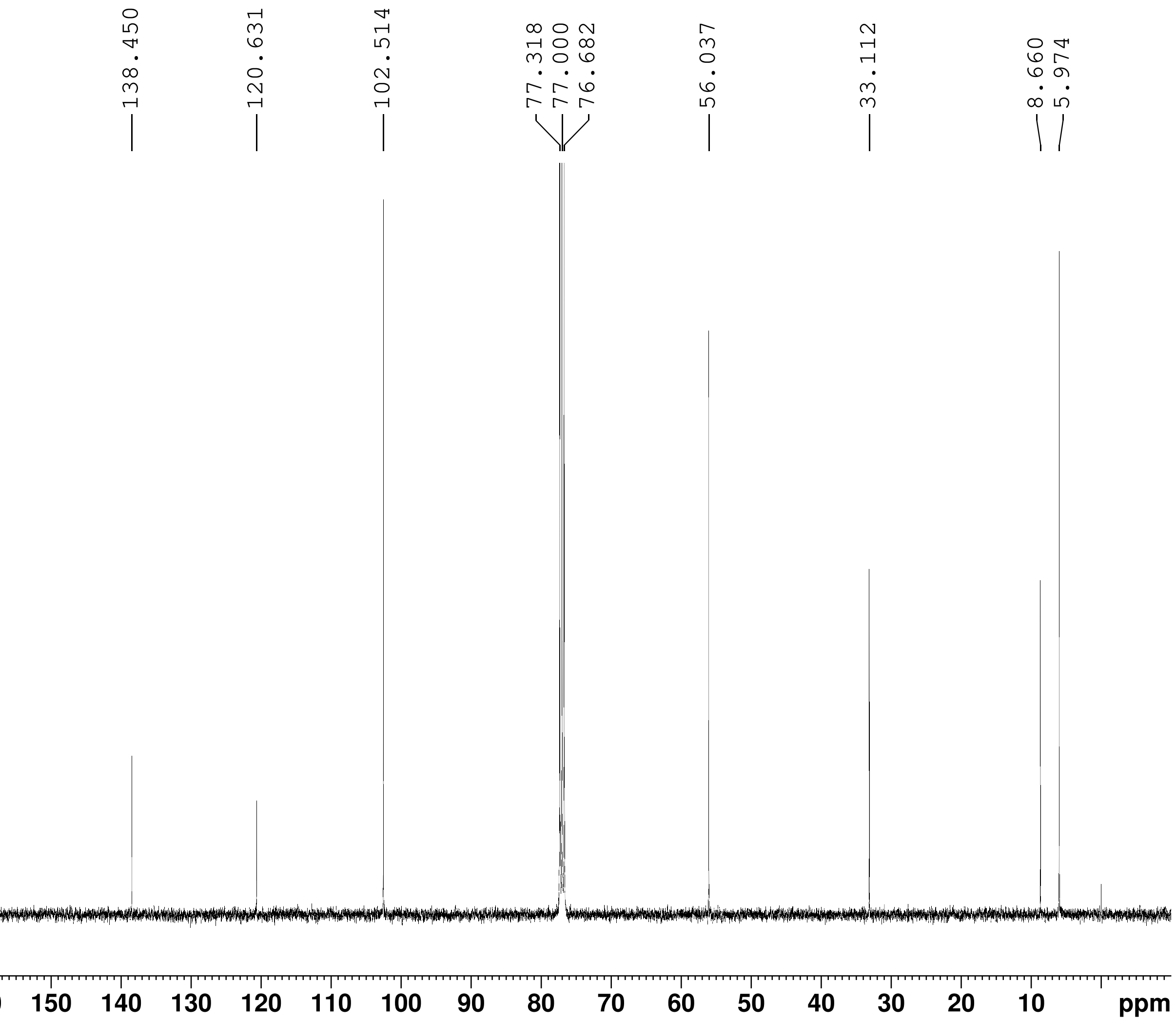

^{1}H NMR of **7aa** (400 MHz, $CDCl_3$)

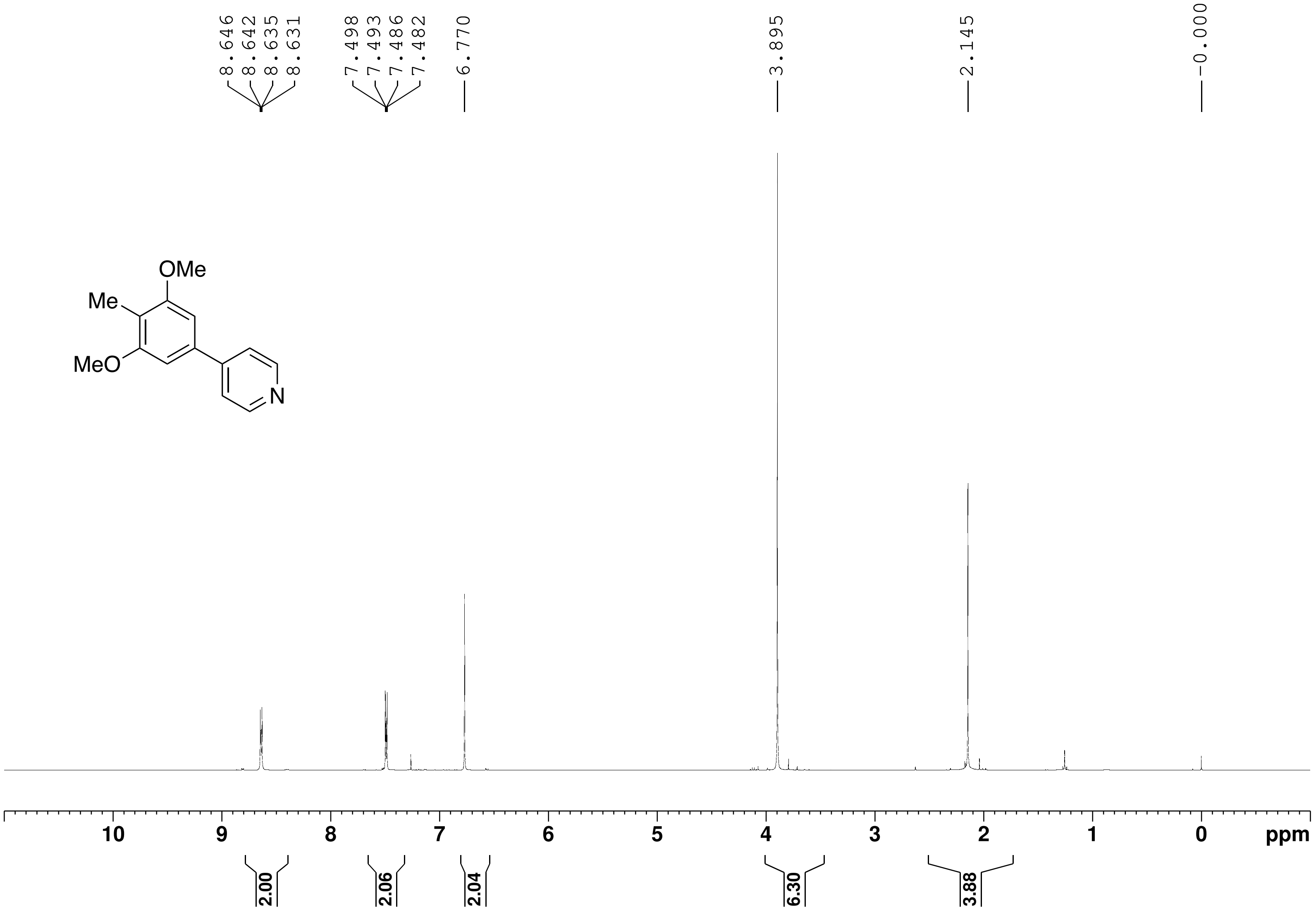

^{13}C NMR of **7aa** (100.6 MHz, $CDCl_3$)

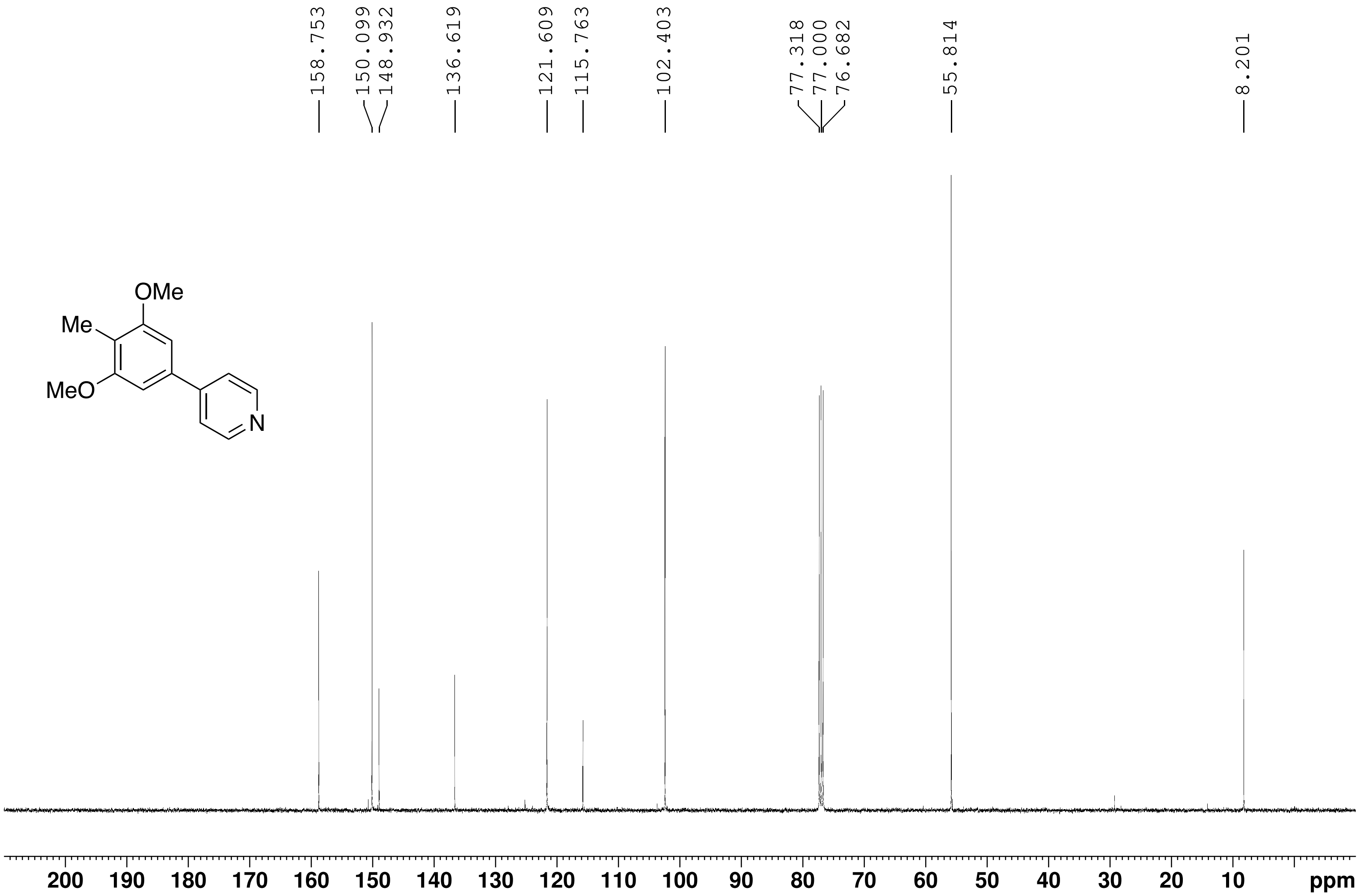

^{1}H NMR of **7ab** (400 MHz, CDCl$_3$)

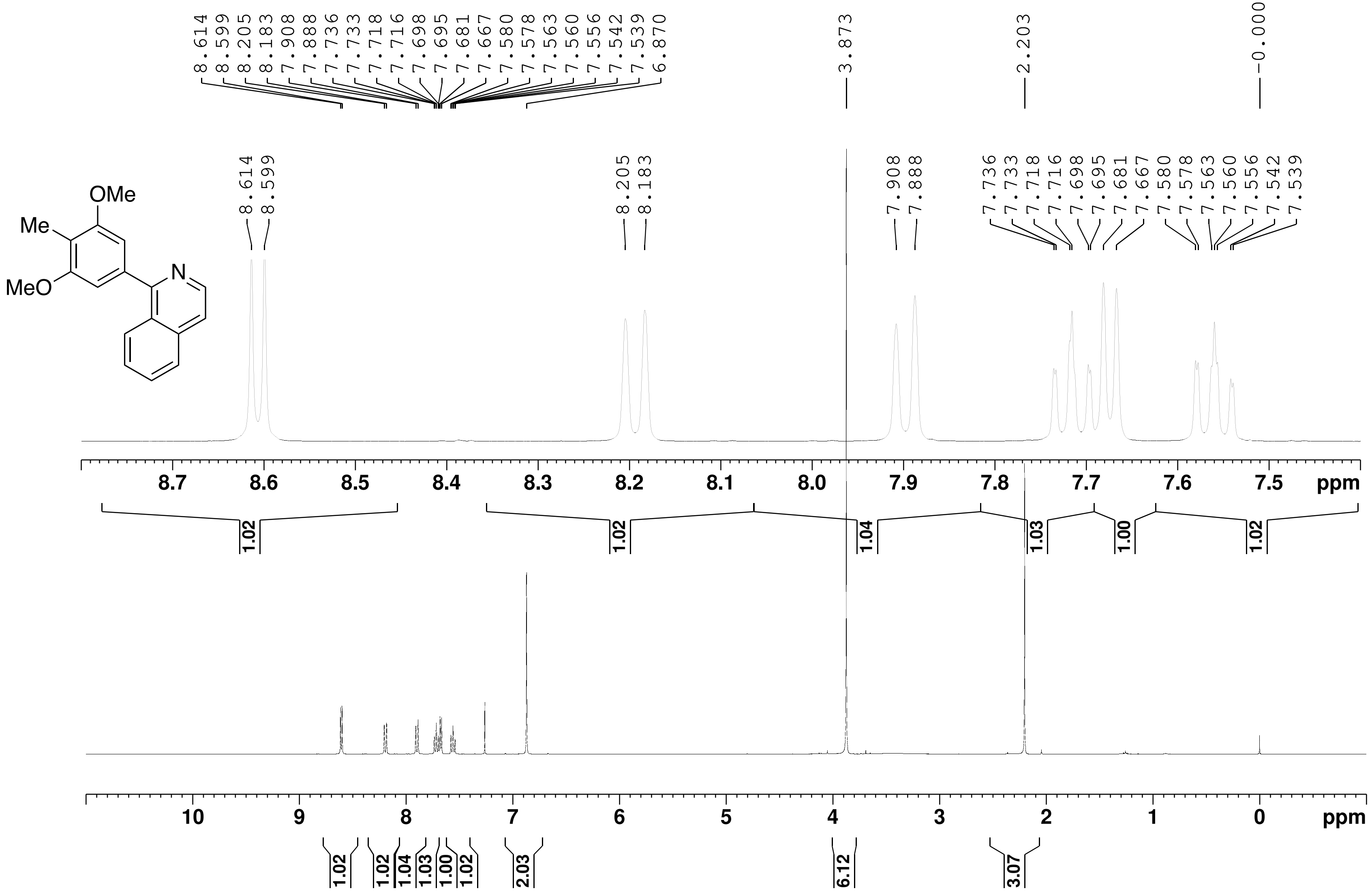

^{13}C NMR of **7ab** (100.6 MHz, $CDCl_3$)

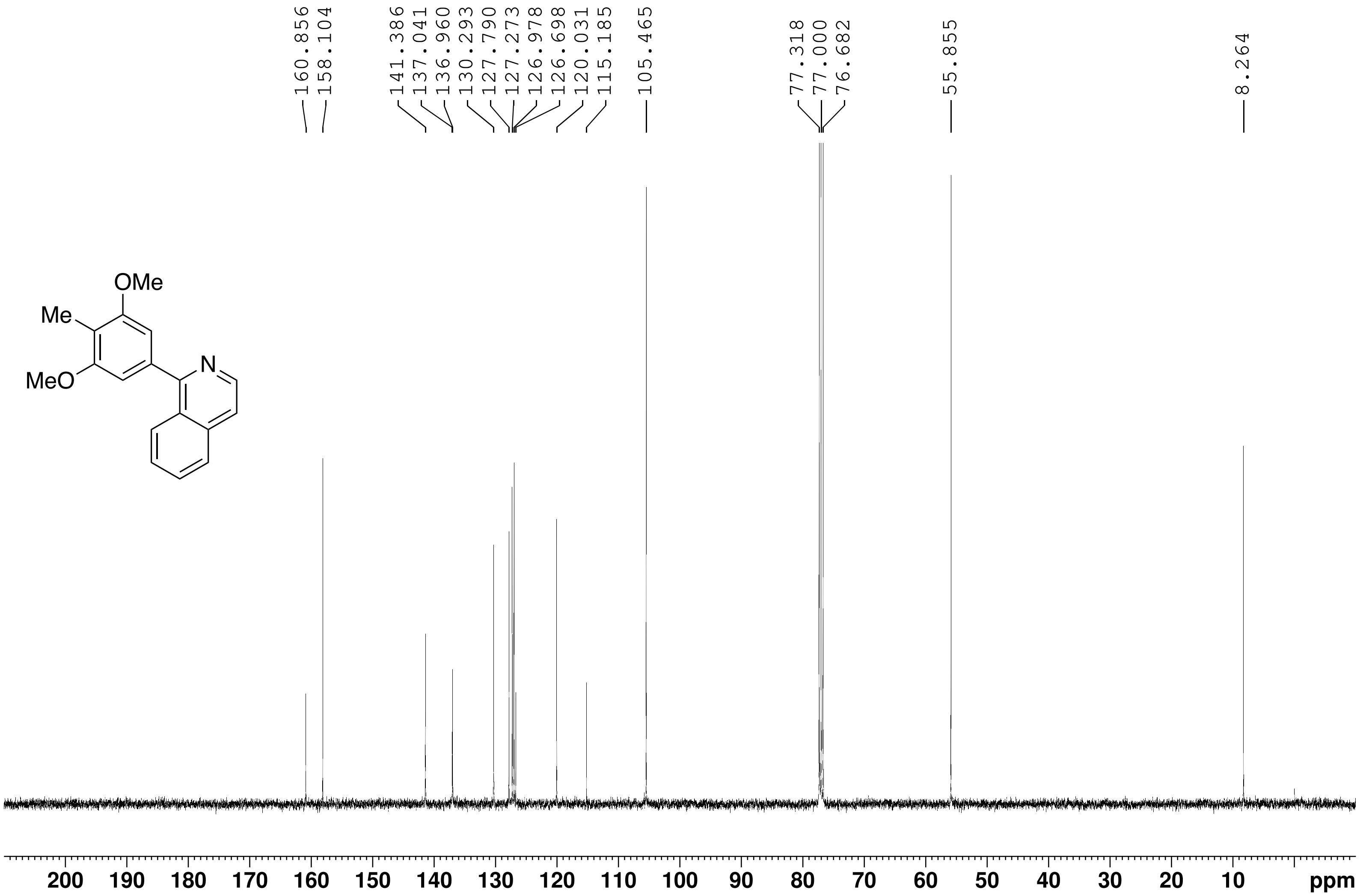

^{1}H NMR of **7ba** (400 MHz, $CDCl_3$)

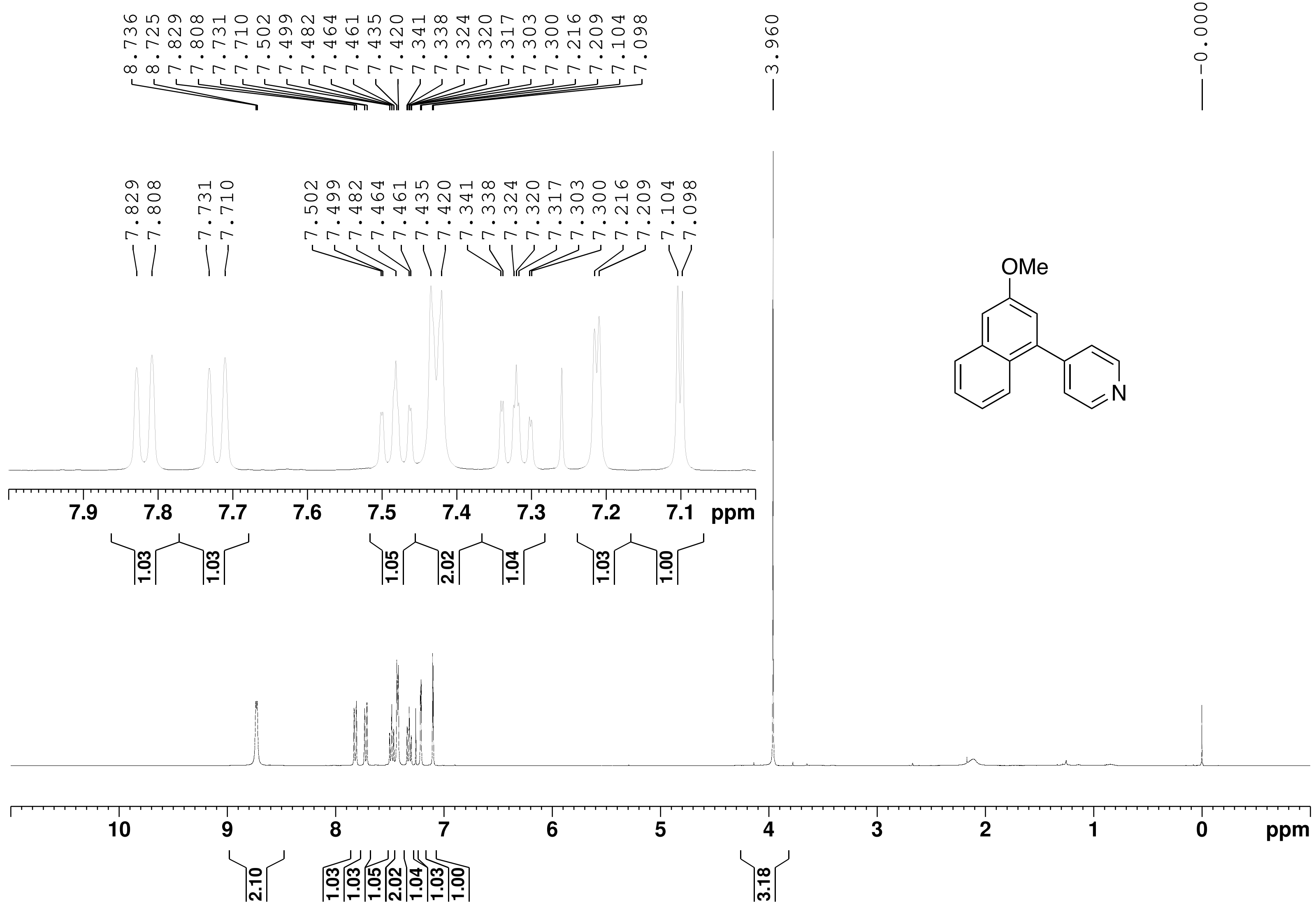

^{13}C NMR of **7ba** (100.6 MHz, $CDCl_3$)

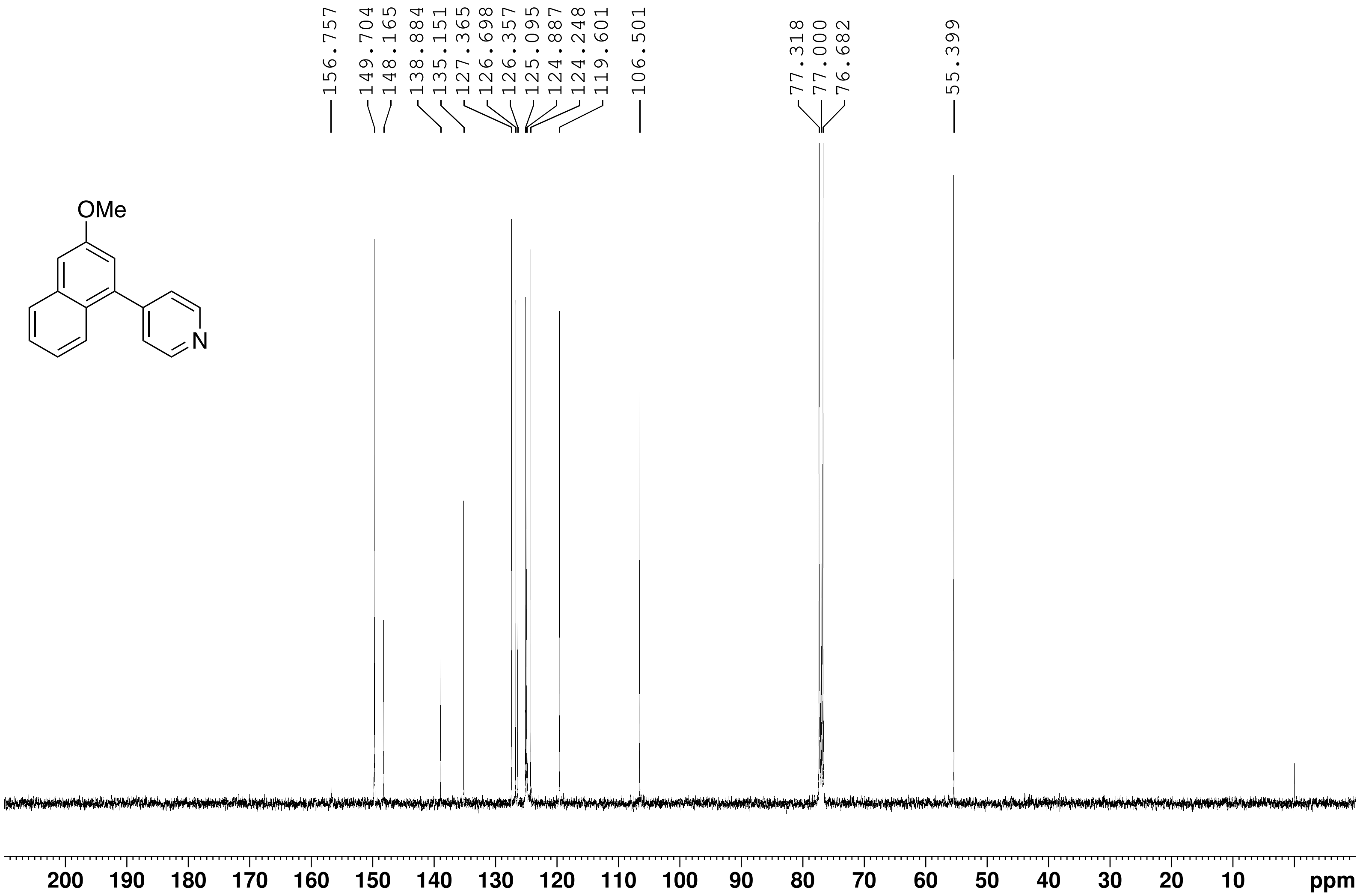

^{1}H NMR of **9aa** (400 MHz, $CDCl_3$)

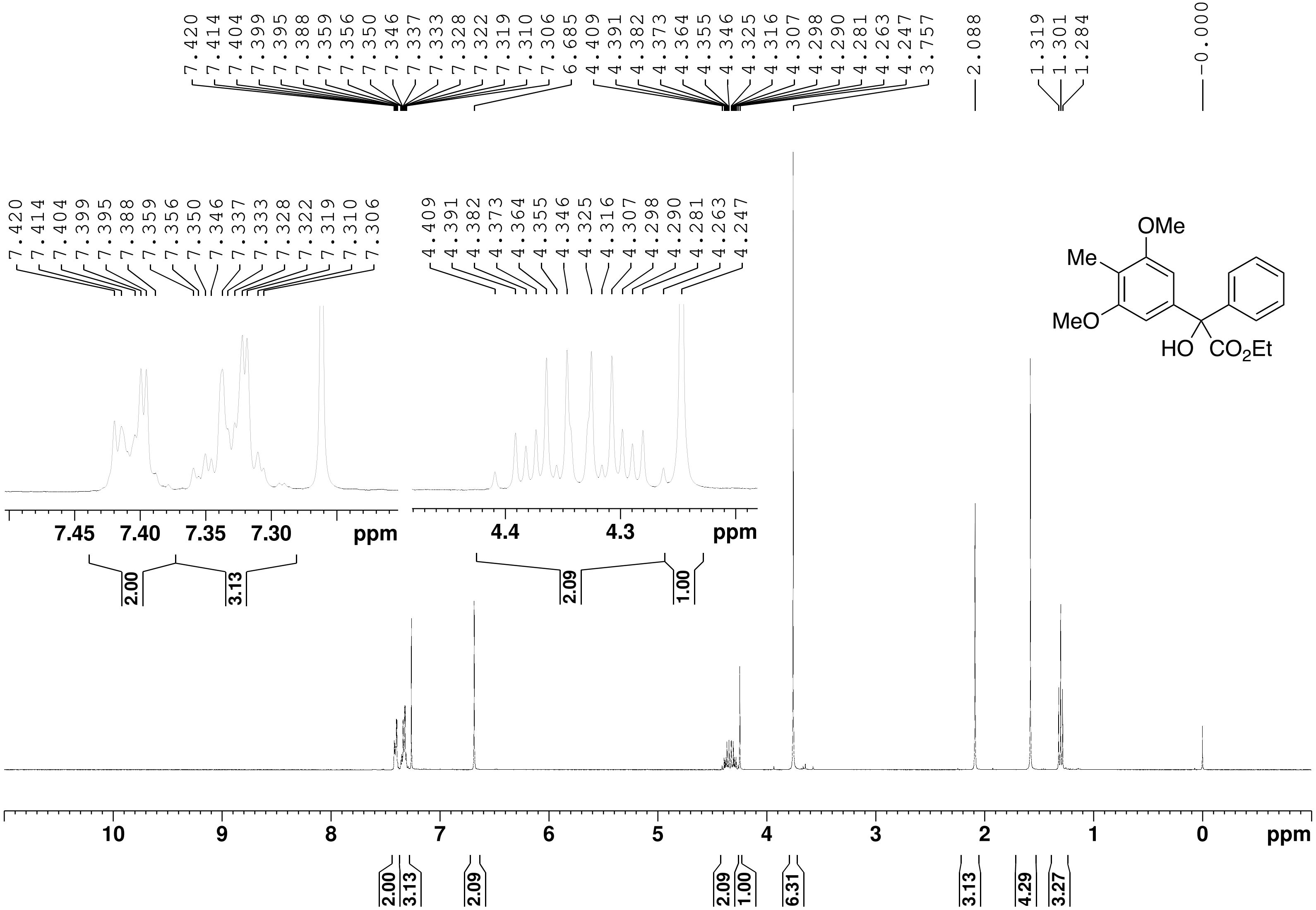

^{13}C NMR of **9aa** (100.6 MHz, $CDCl_3$)

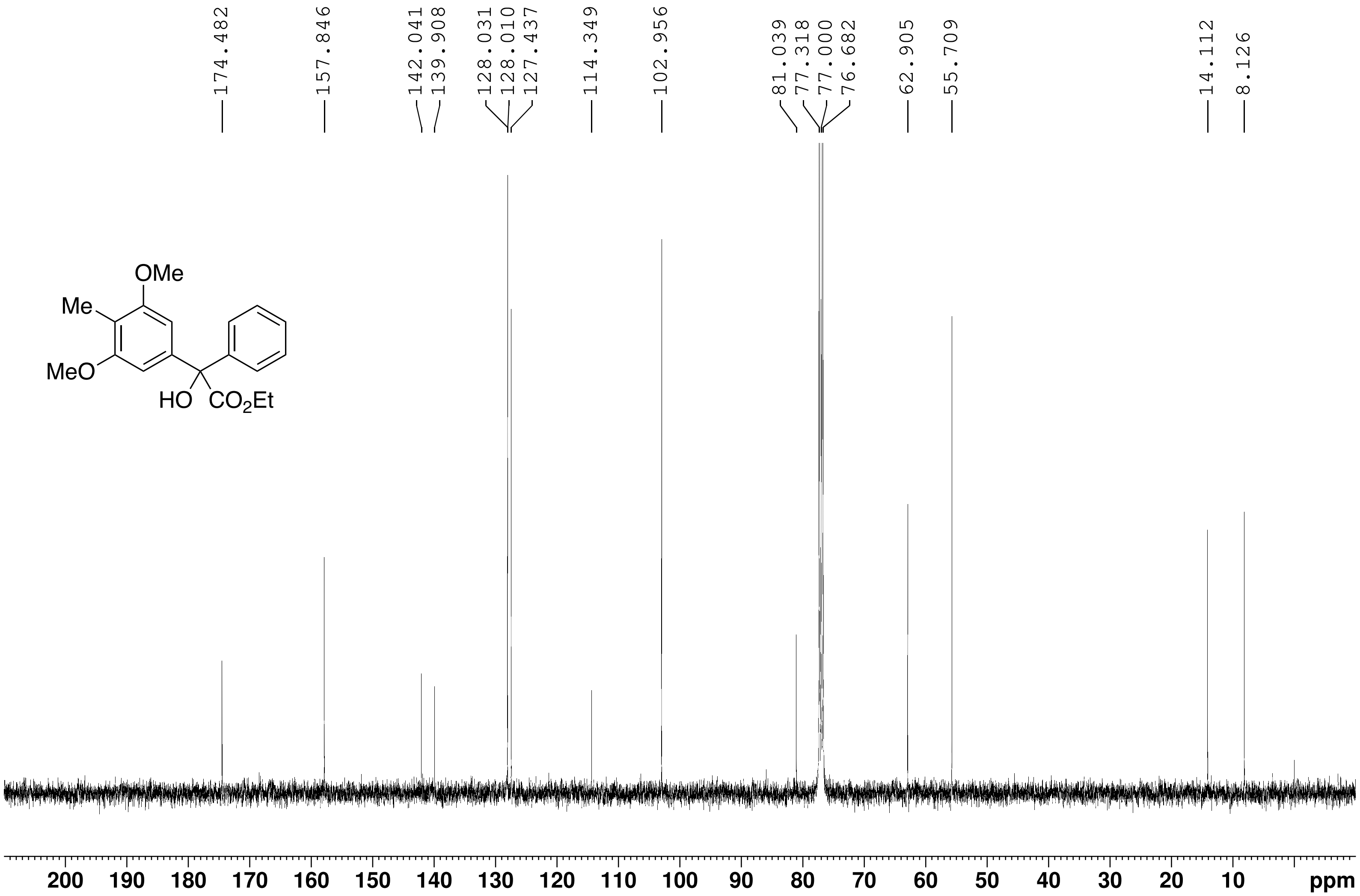

^{1}H NMR of **3ba** (400 MHz, $CDCl_3$)

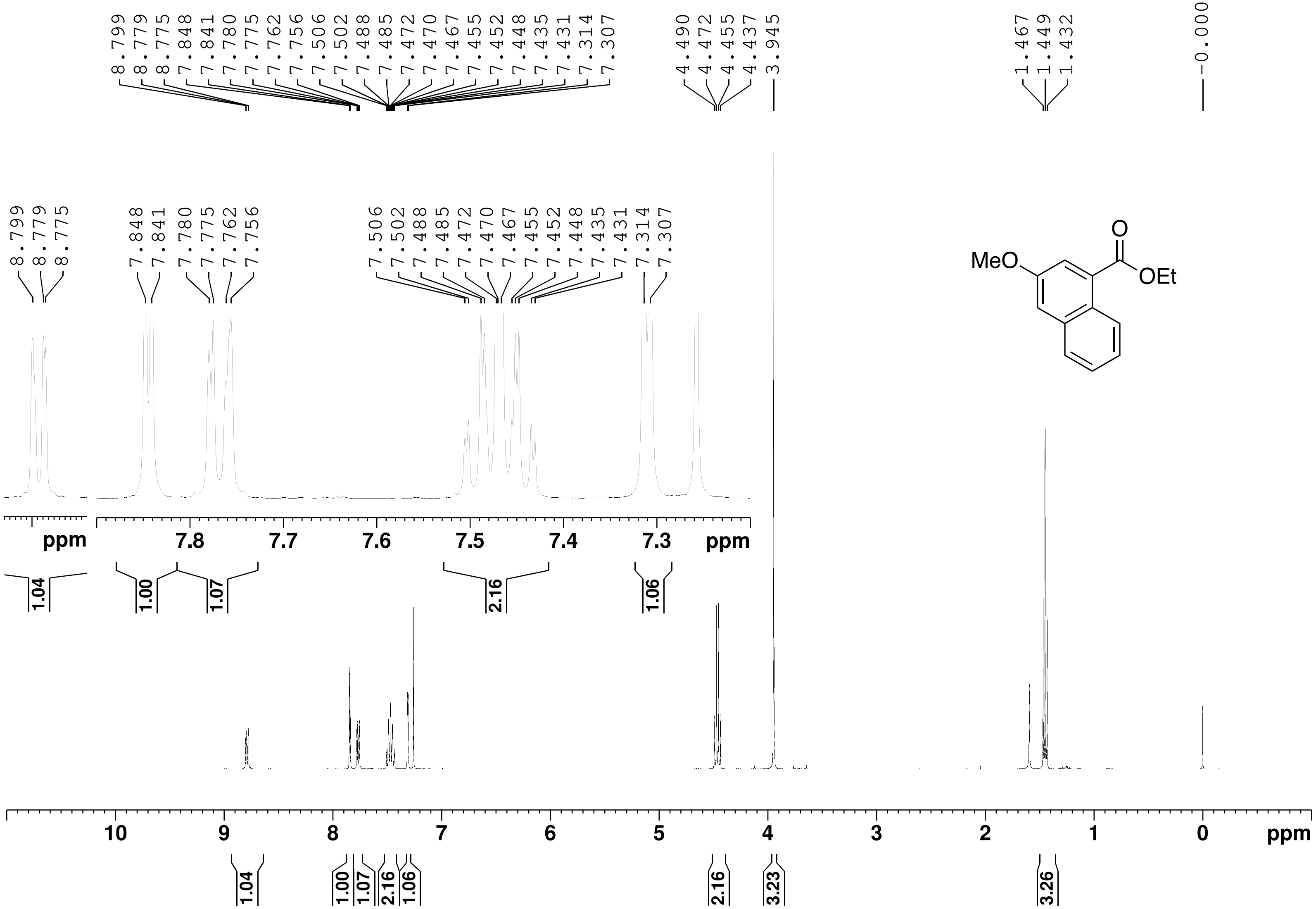

^{13}C NMR of **3ba** (100.6 MHz, $CDCl_3$)

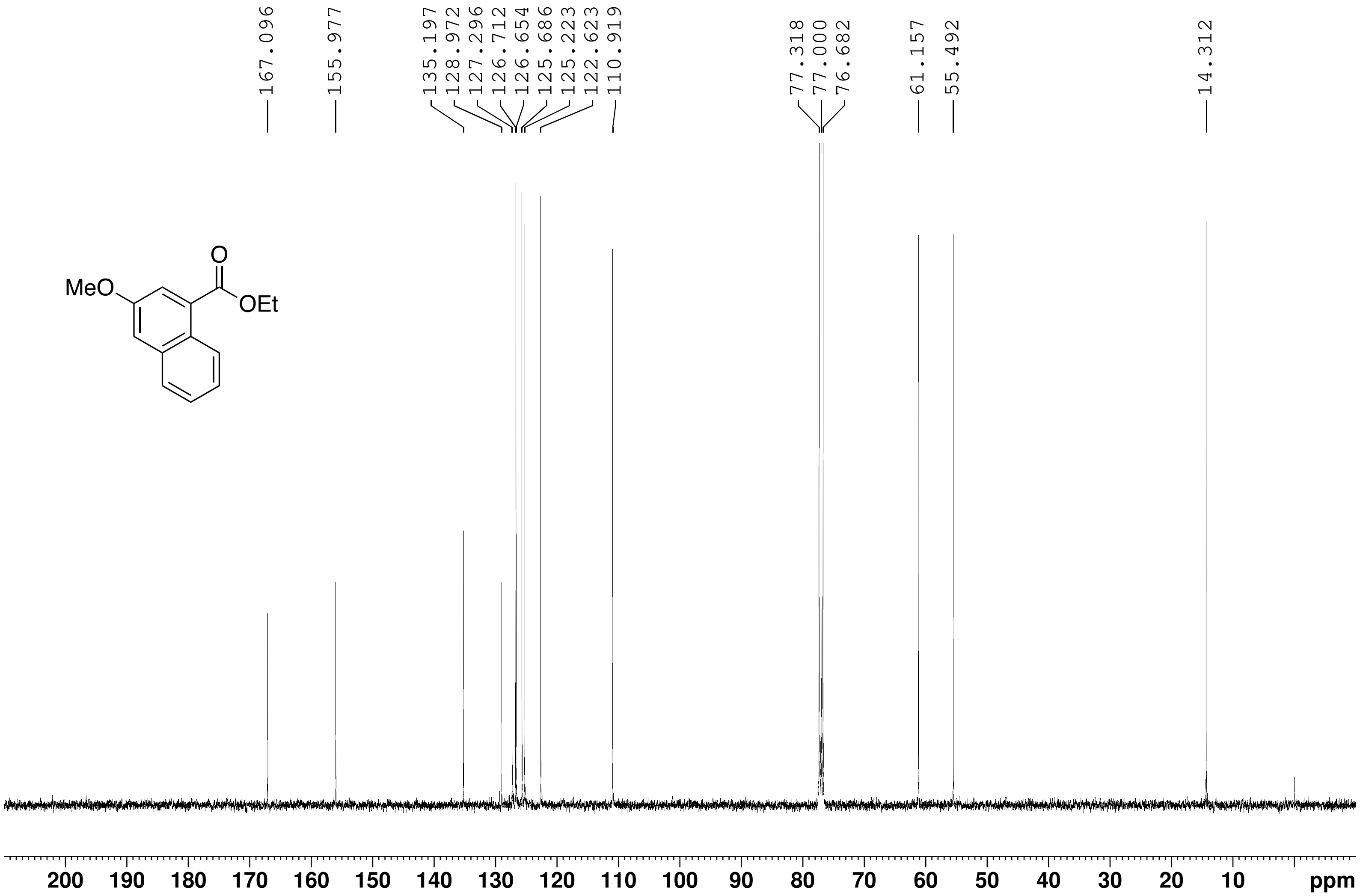

^{1}H NMR of **3ca** (400 MHz, $CDCl_3$)

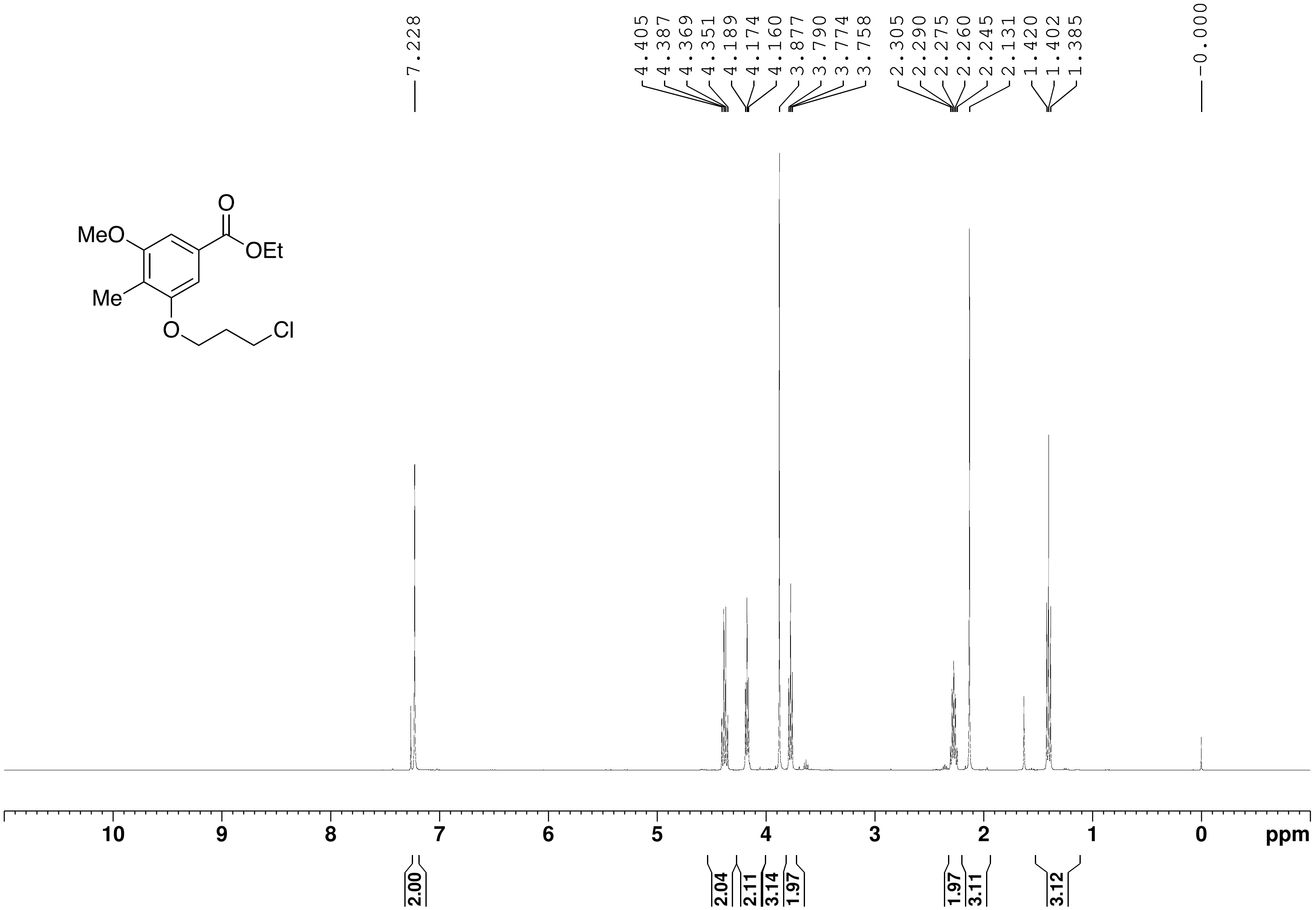

^{13}C NMR of **3ca** (100.6 MHz, $CDCl_3$)

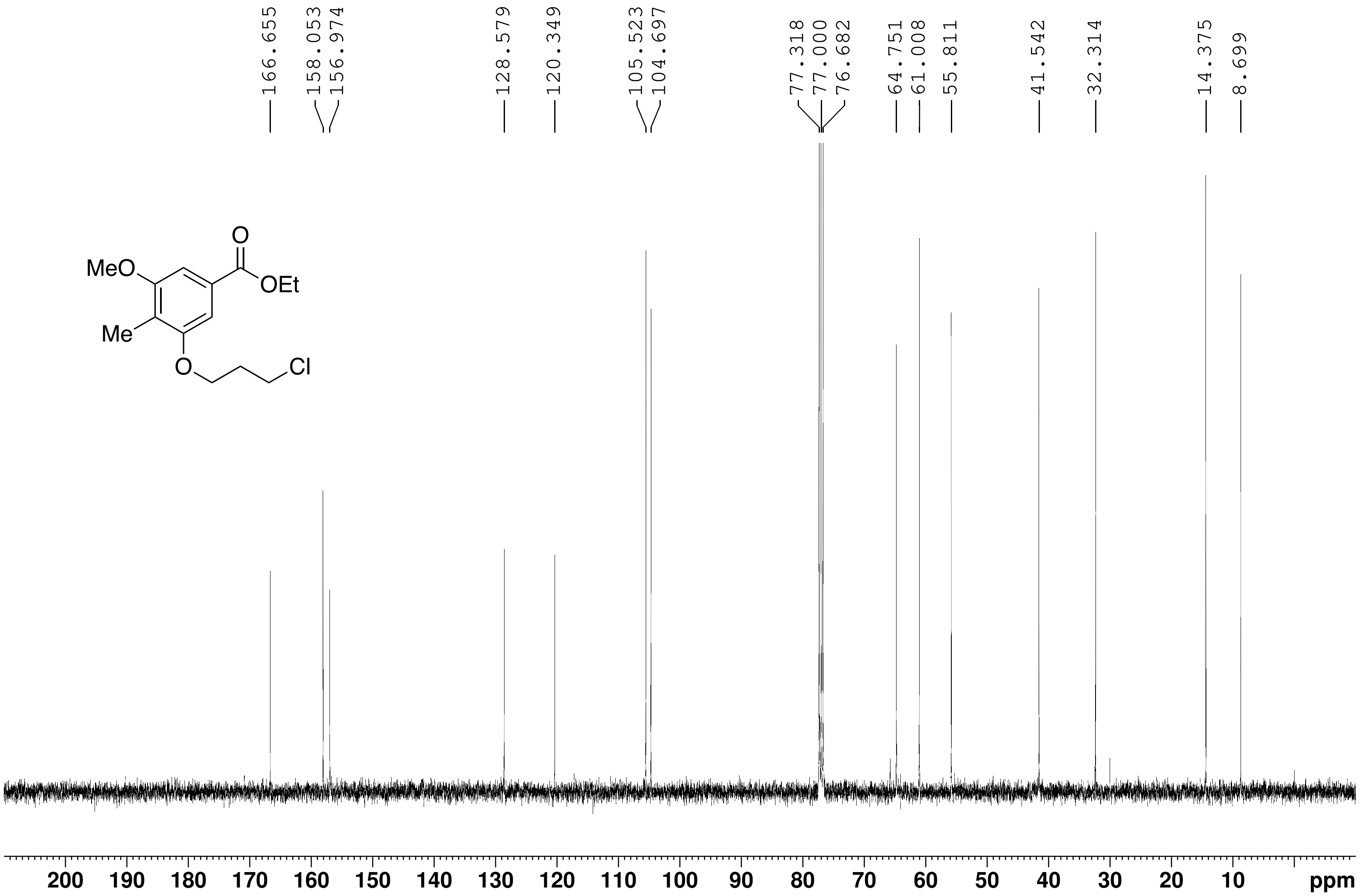

^{1}H NMR of **3da** (400 MHz, $CDCl_3$)

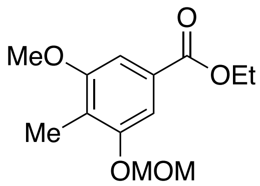

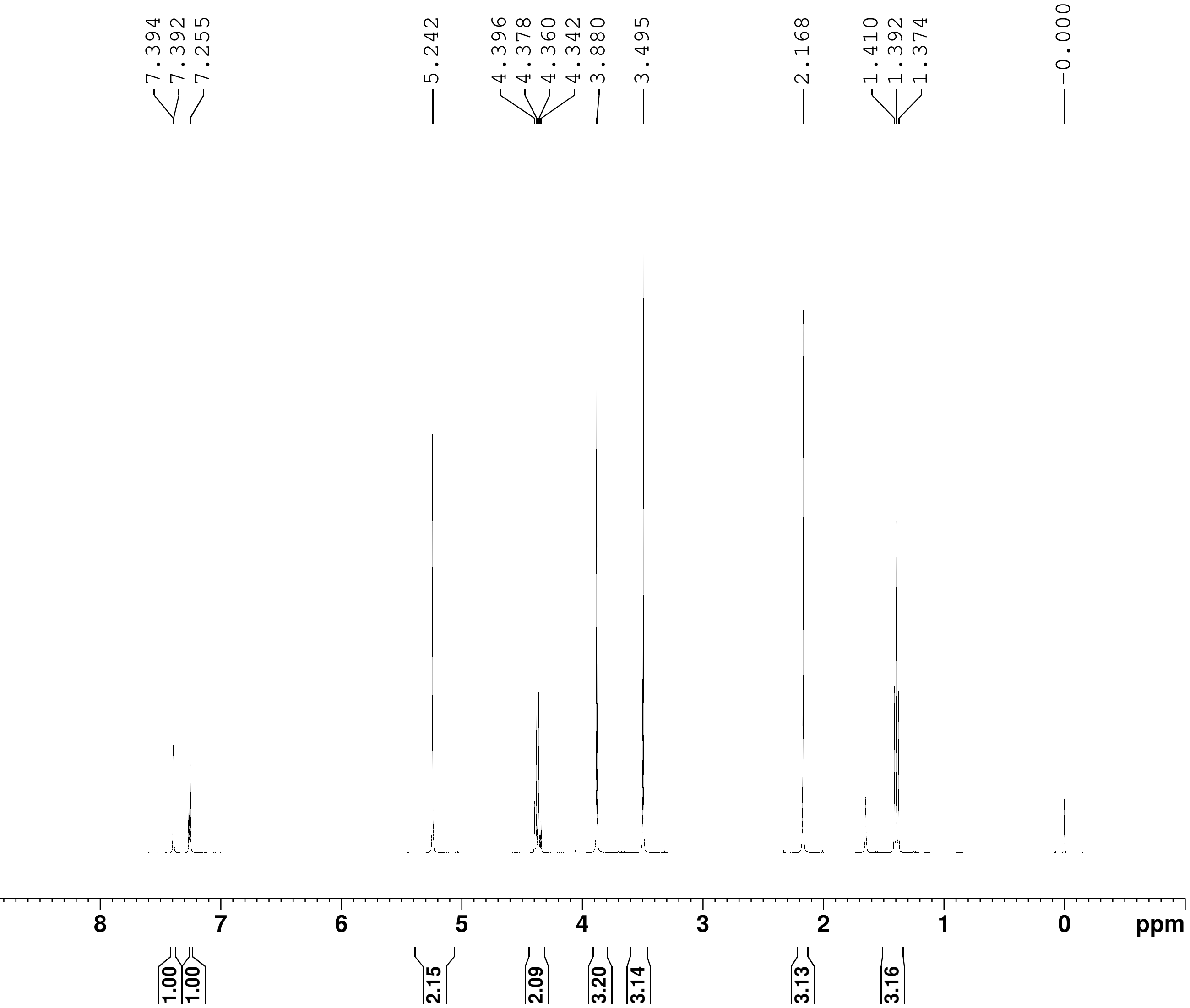

^{13}C NMR of **3da** (100.6 MHz, $CDCl_3$)

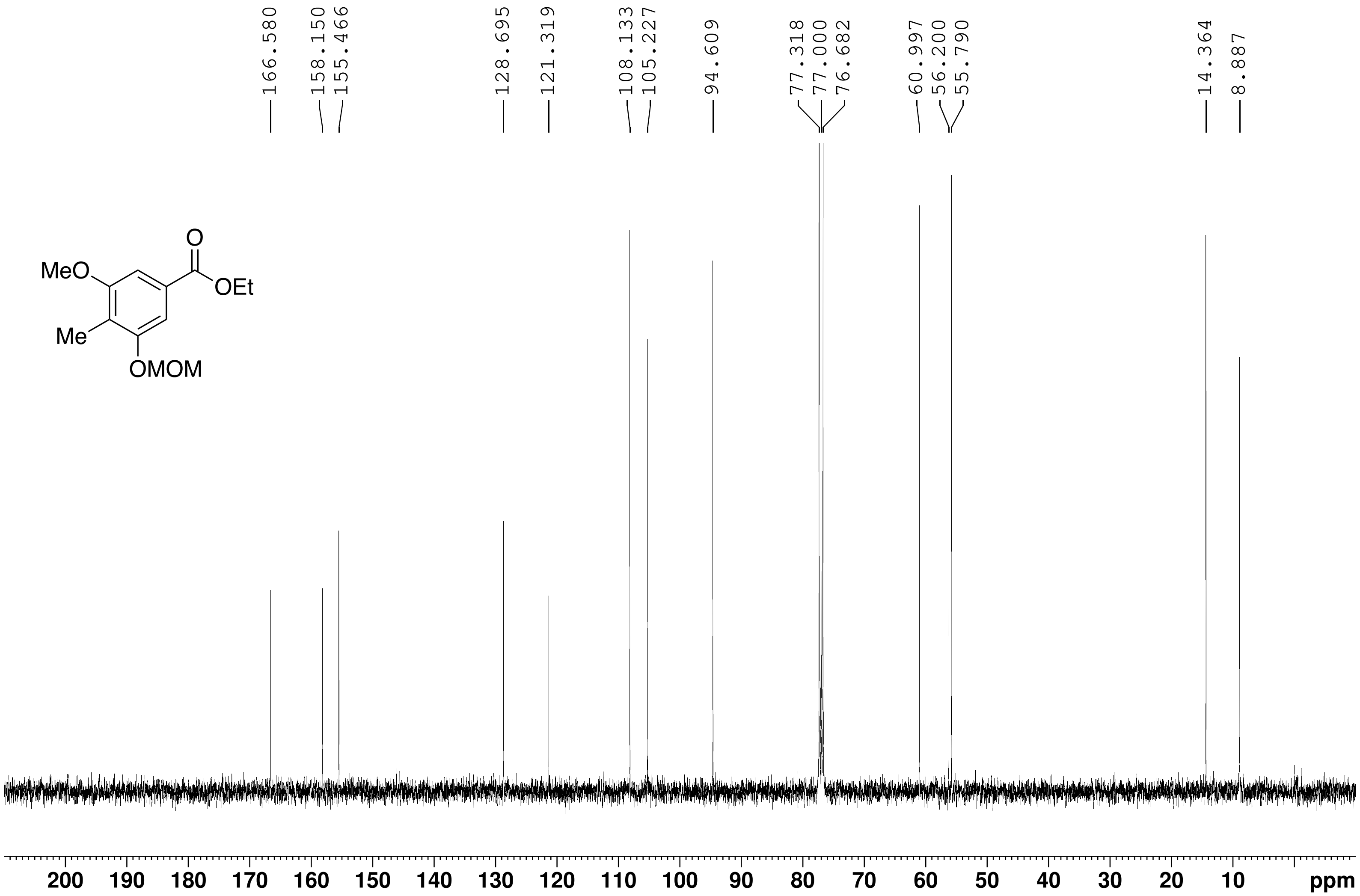

^{1}H NMR of **3ea** (400 MHz, $CDCl_3$)

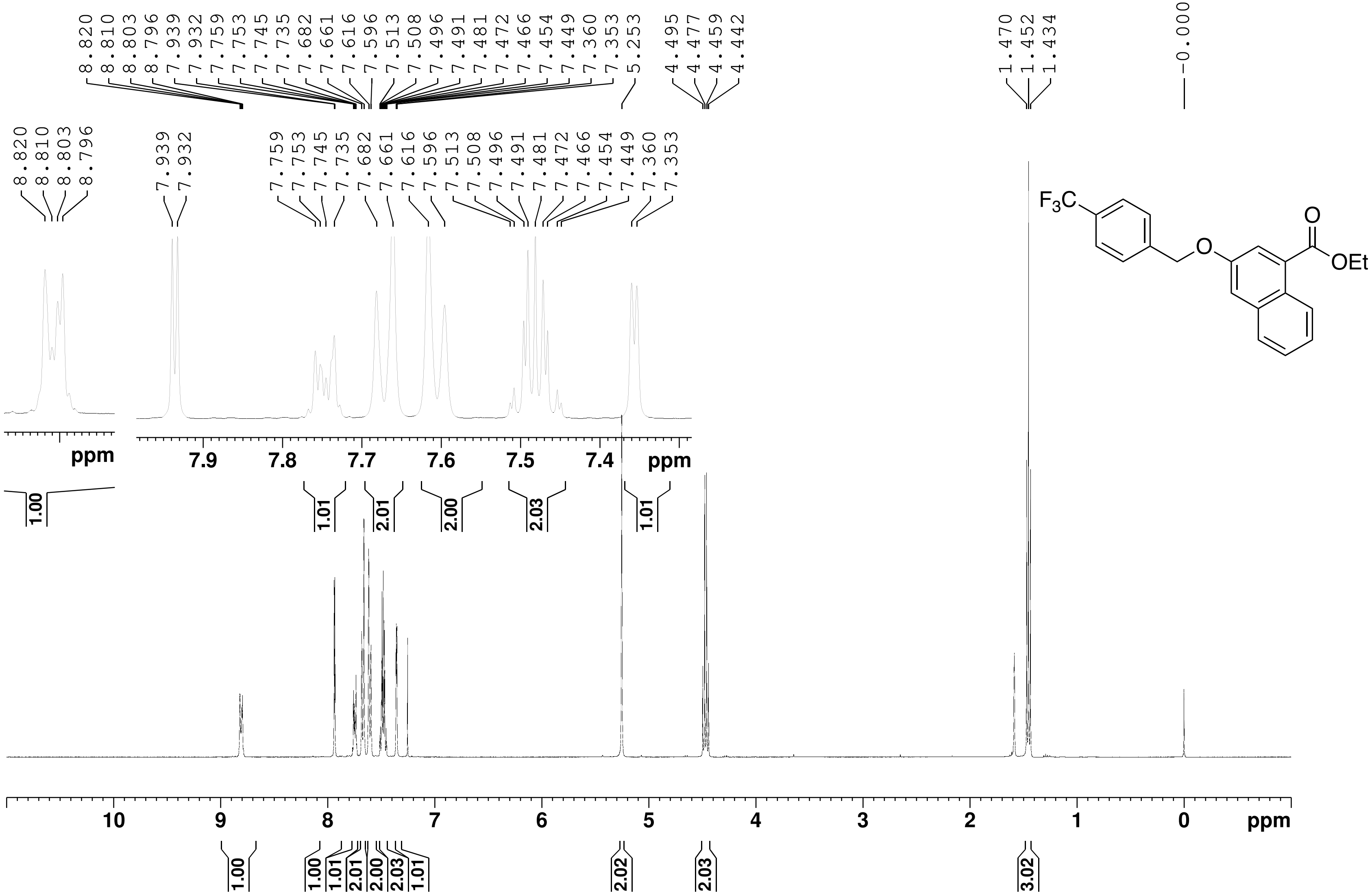

^{13}C NMR of **3ea** (100.6 MHz, $CDCl_3$)

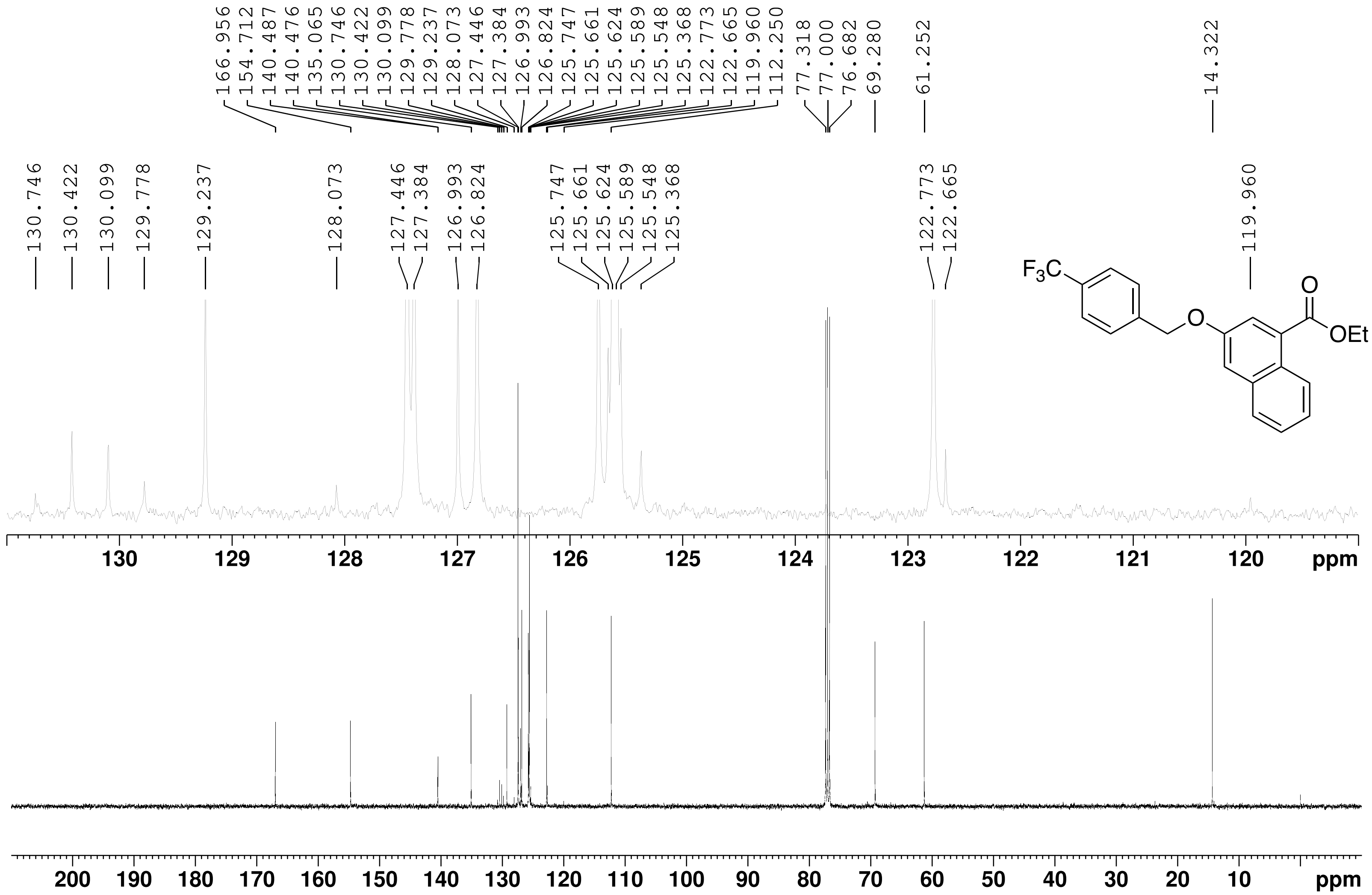

^{19}F NMR of **3ea** (376.5 MHz, $CDCl_3$)

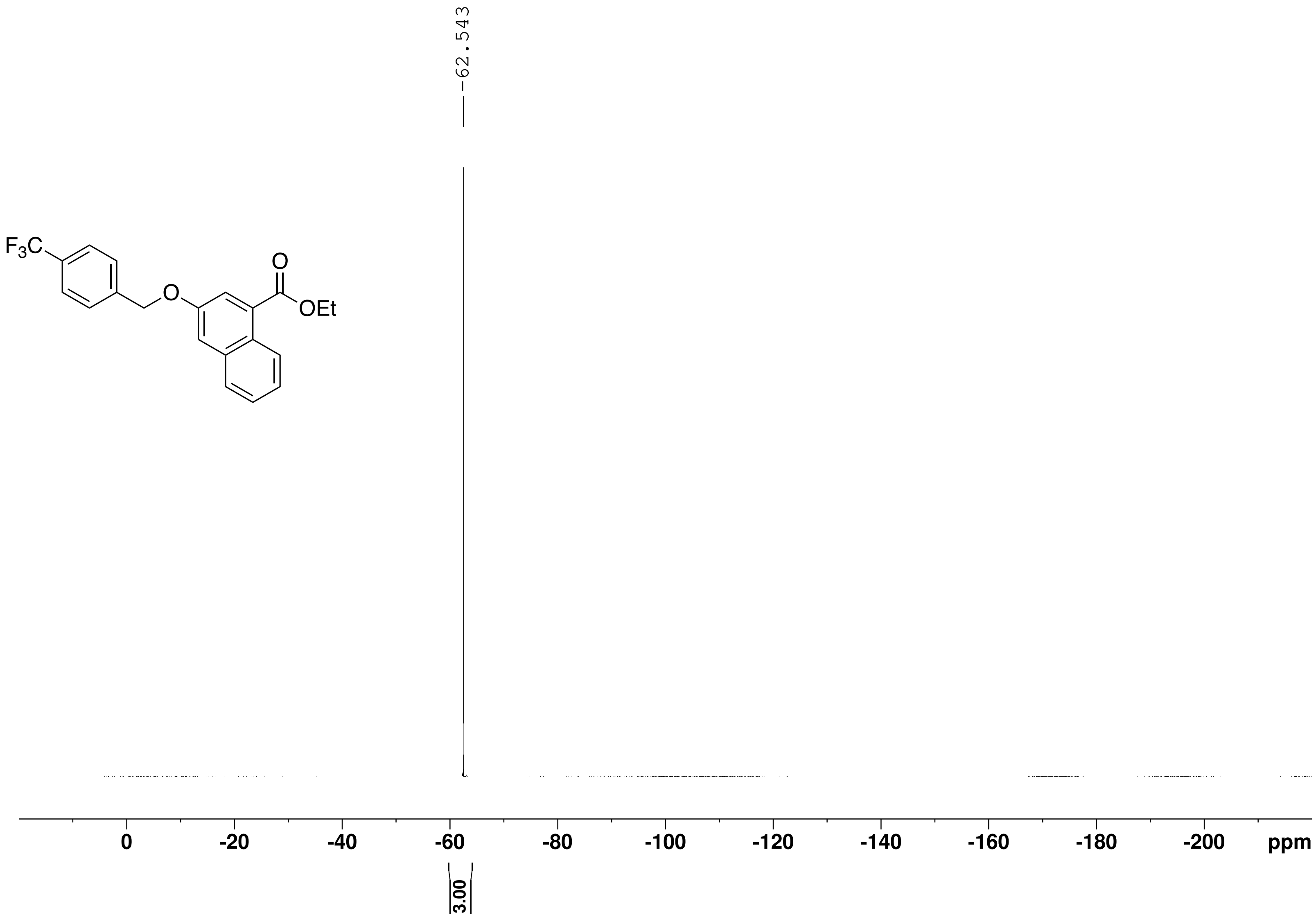

^1H NMR of **3fa** (400 MHz, $CDCl_3$)

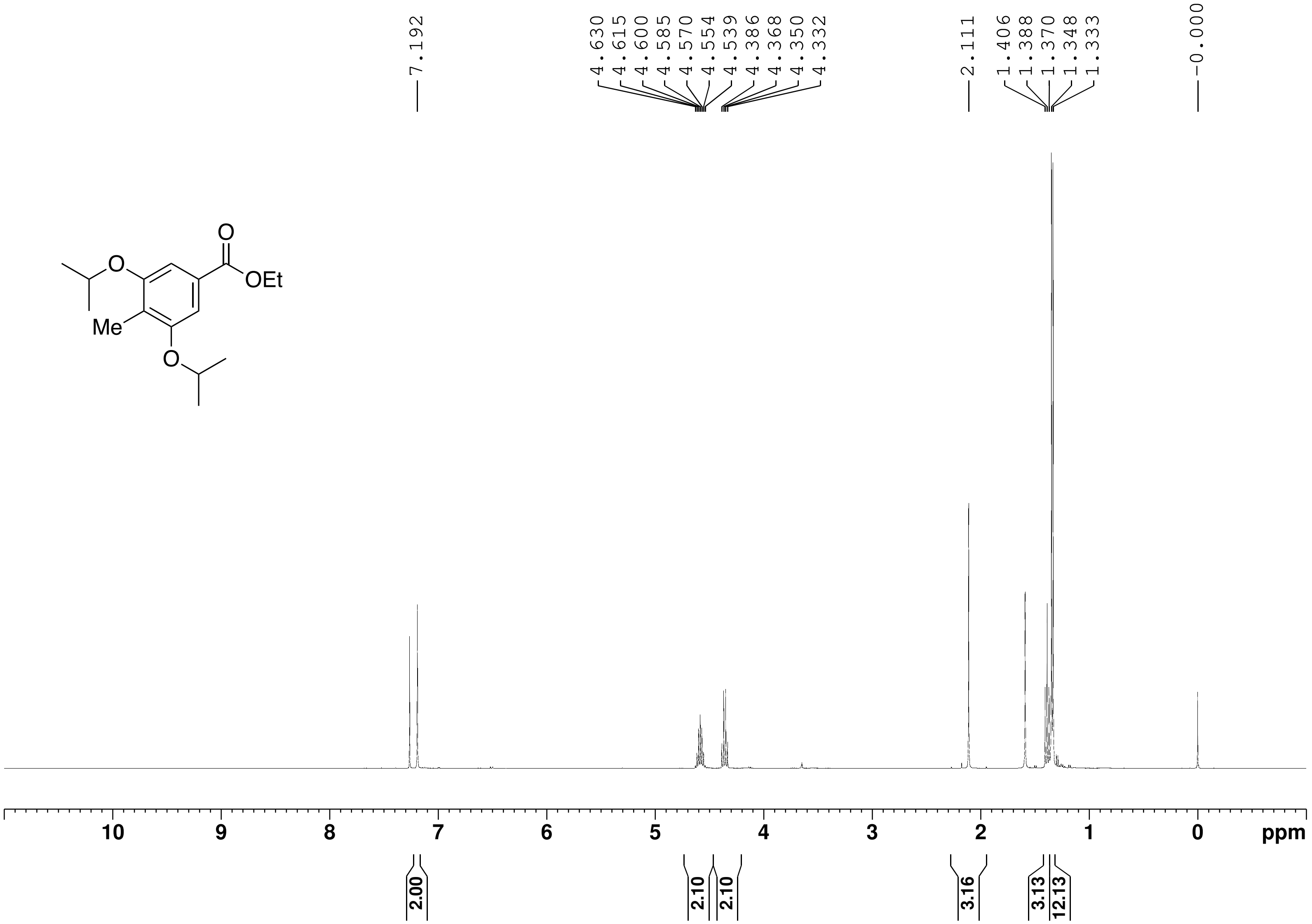

^{13}C NMR of **3fa** (100.6 MHz, $CDCl_3$)

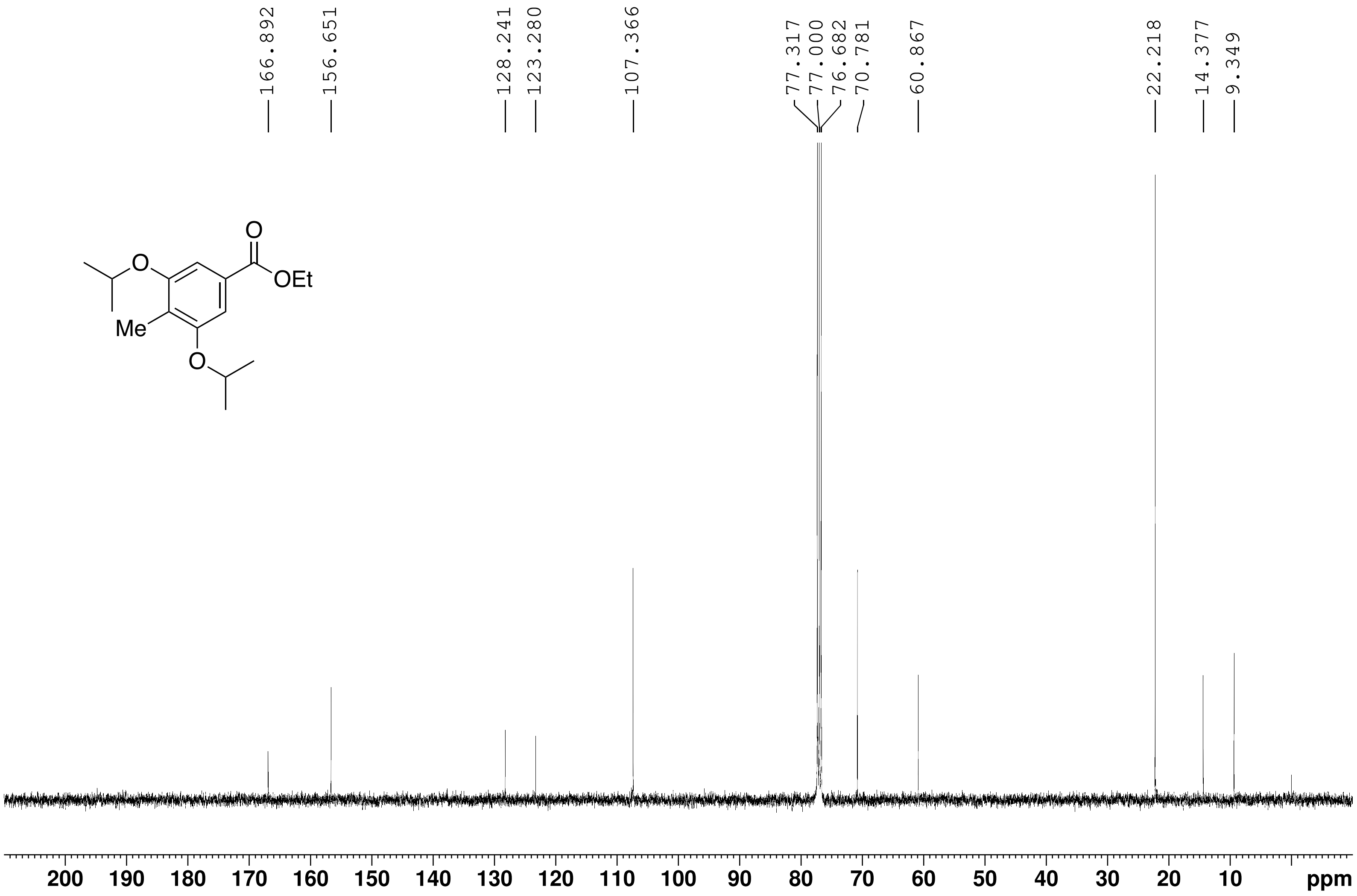

^{1}H NMR of **3ga** (400 MHz, $CDCl_3$)

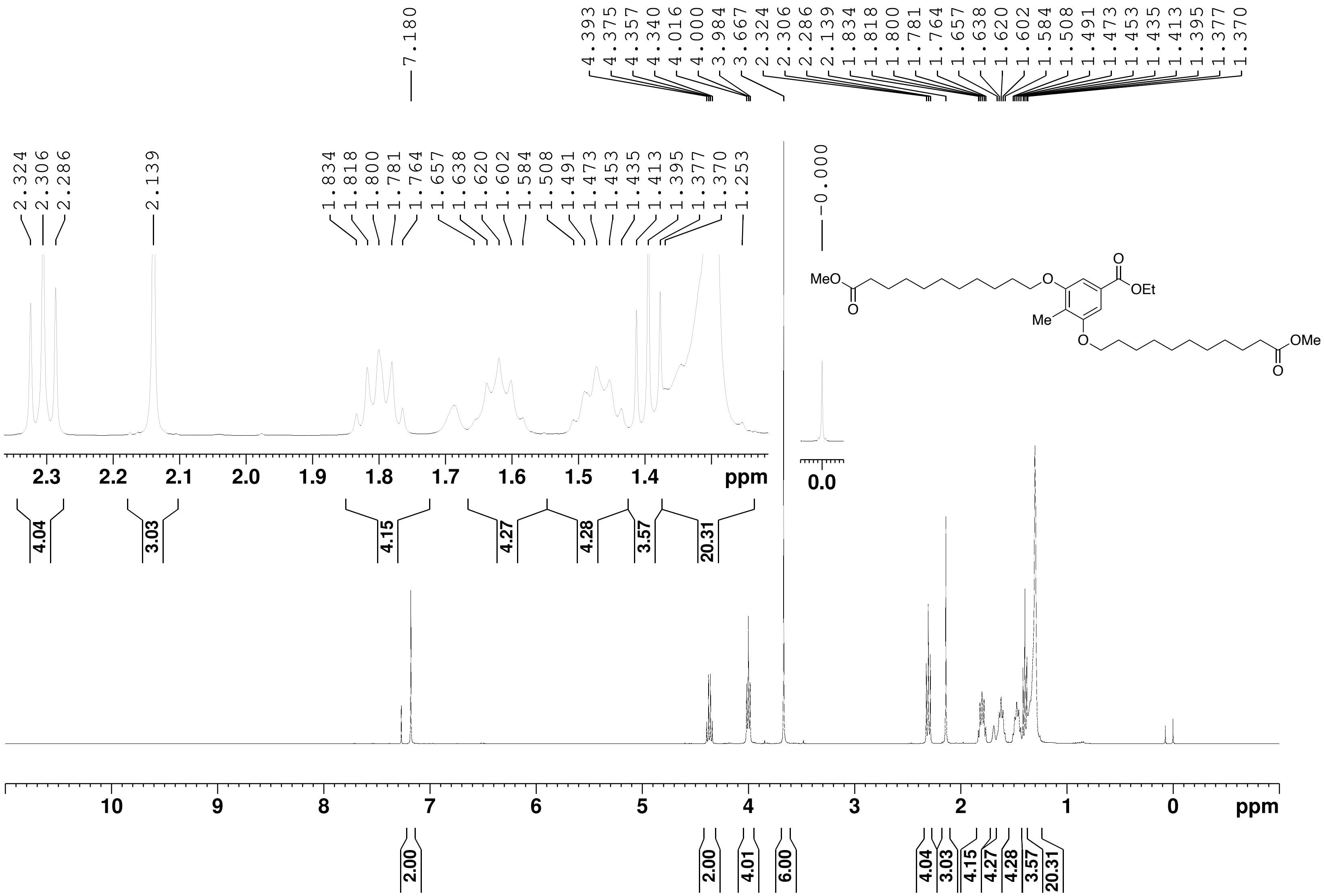

^{13}C NMR of **3ga** (100.6 MHz, $CDCl_3$)

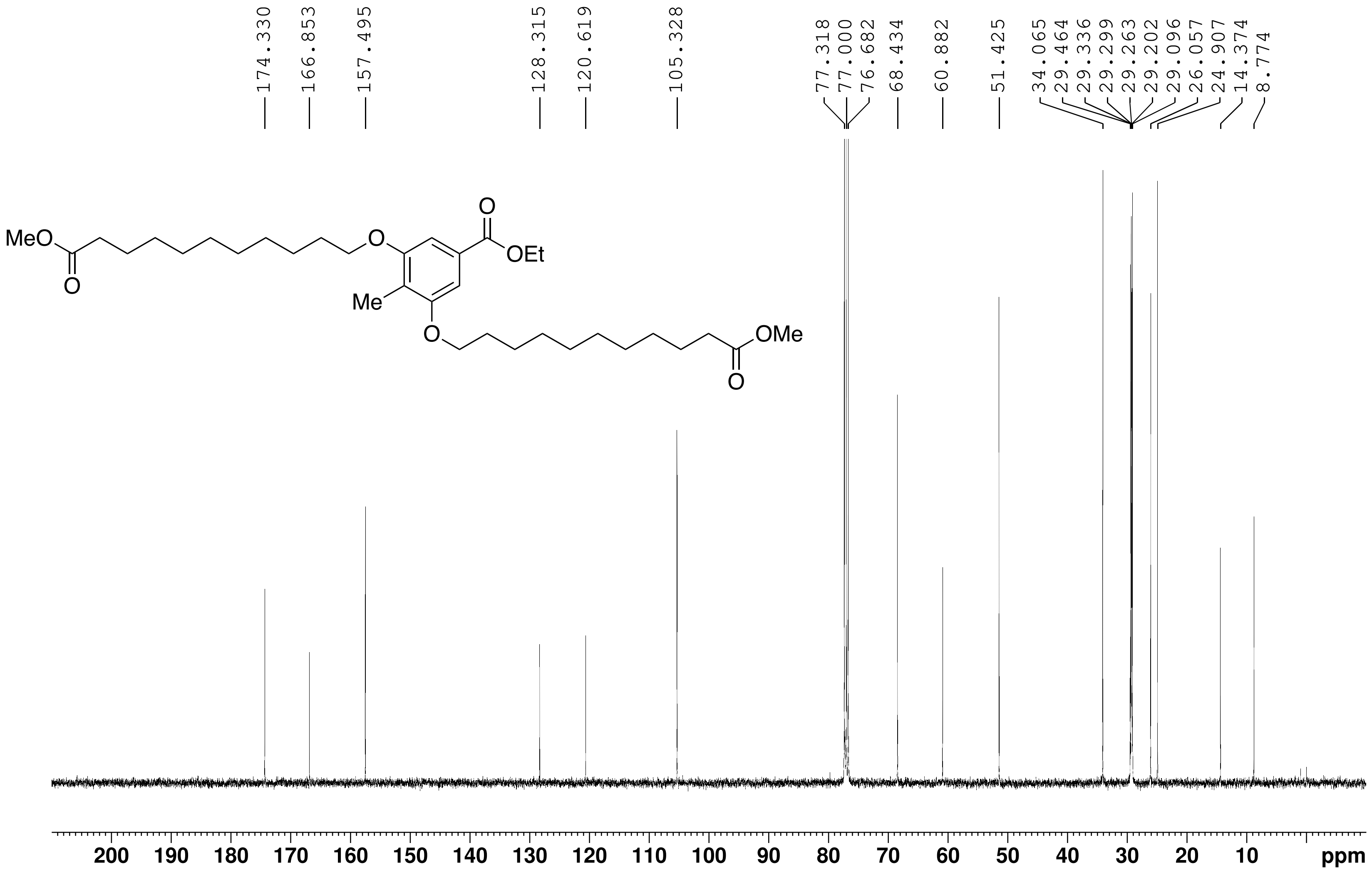

^{1}H NMR of **3ha** (400 MHz, $CDCl_3$)

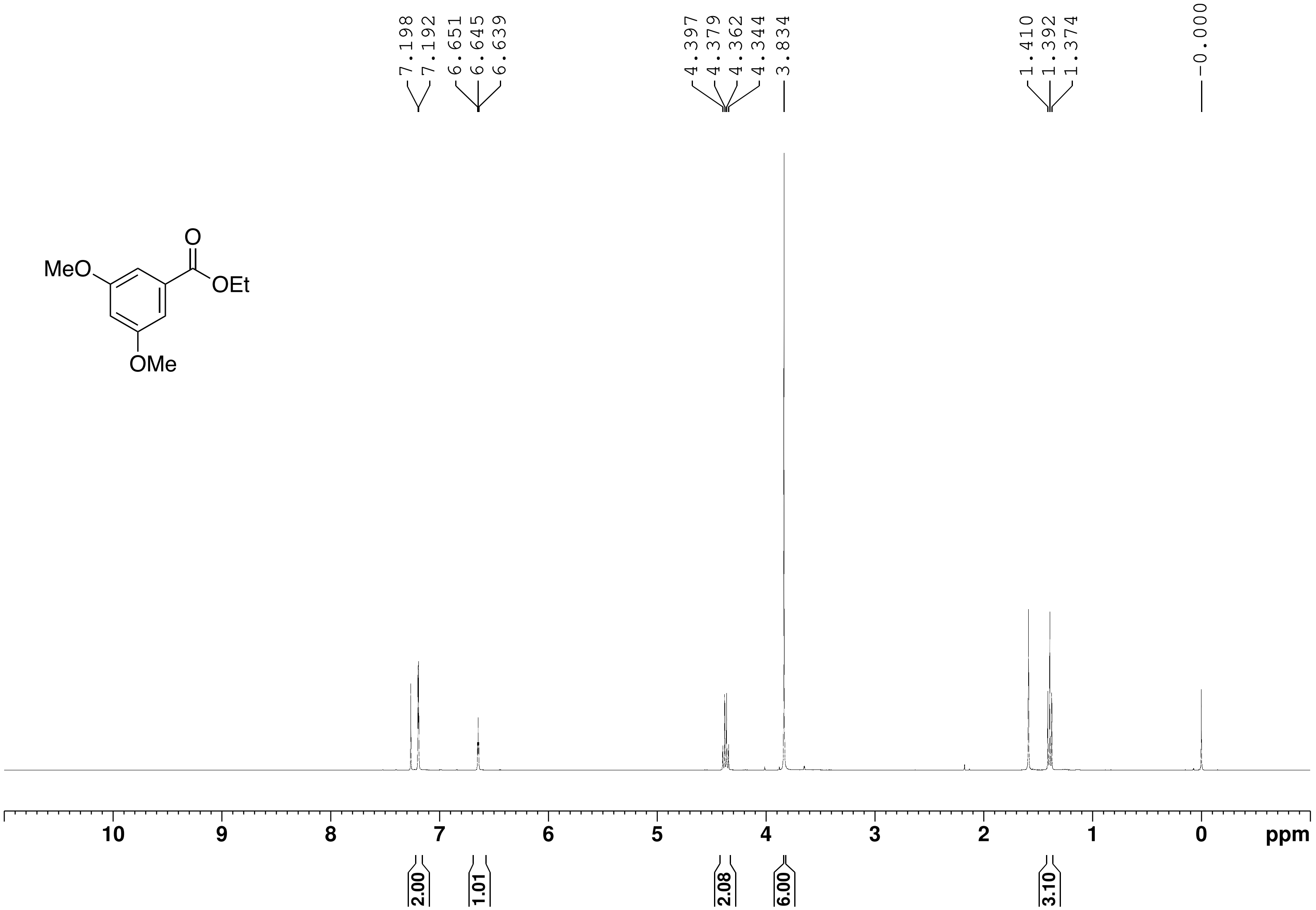

^{13}C NMR of **3ha** (100.6 MHz, $CDCl_3$)

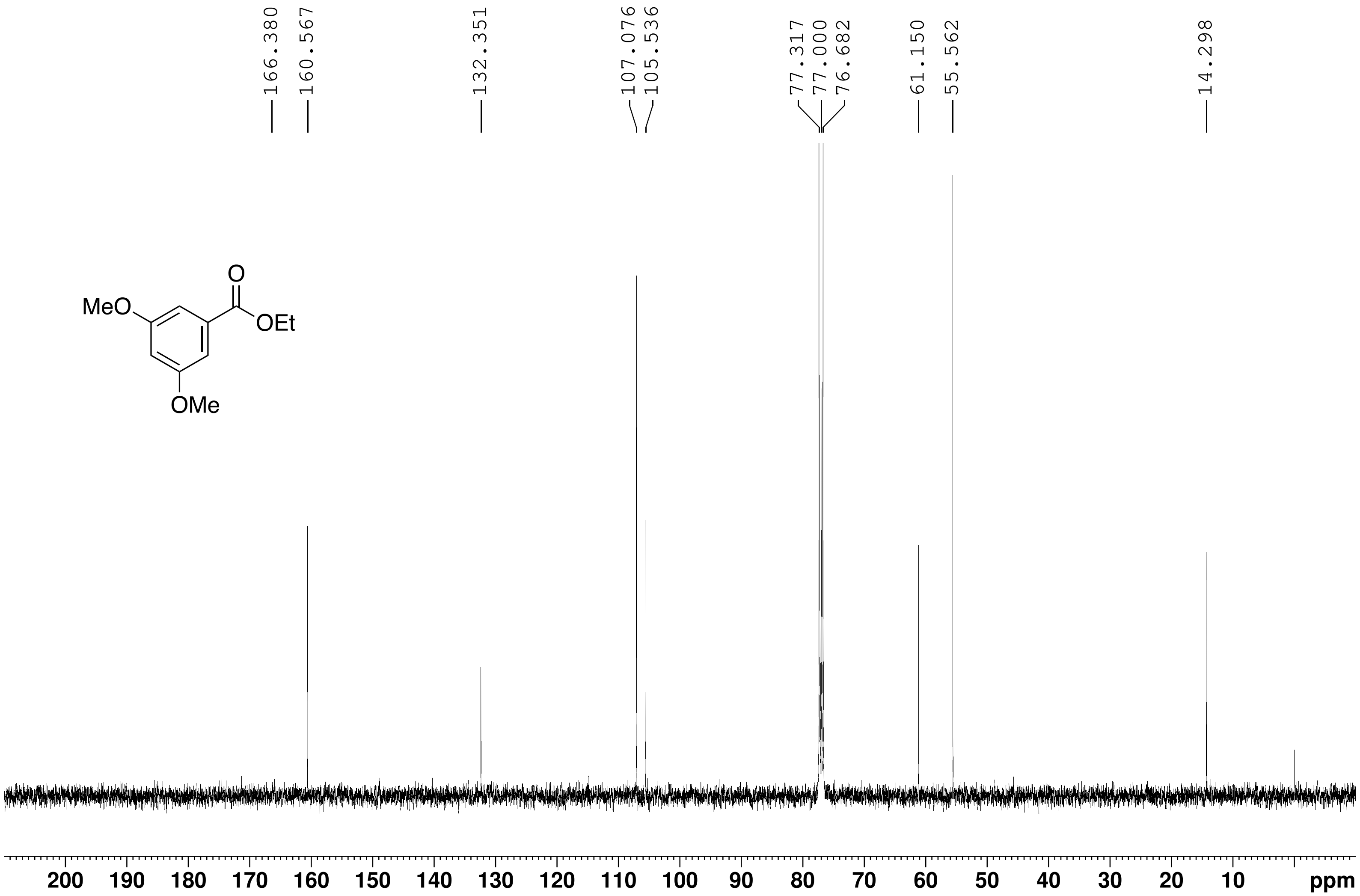

^{1}H NMR of **3ia** (400 MHz, $CDCl_3$)

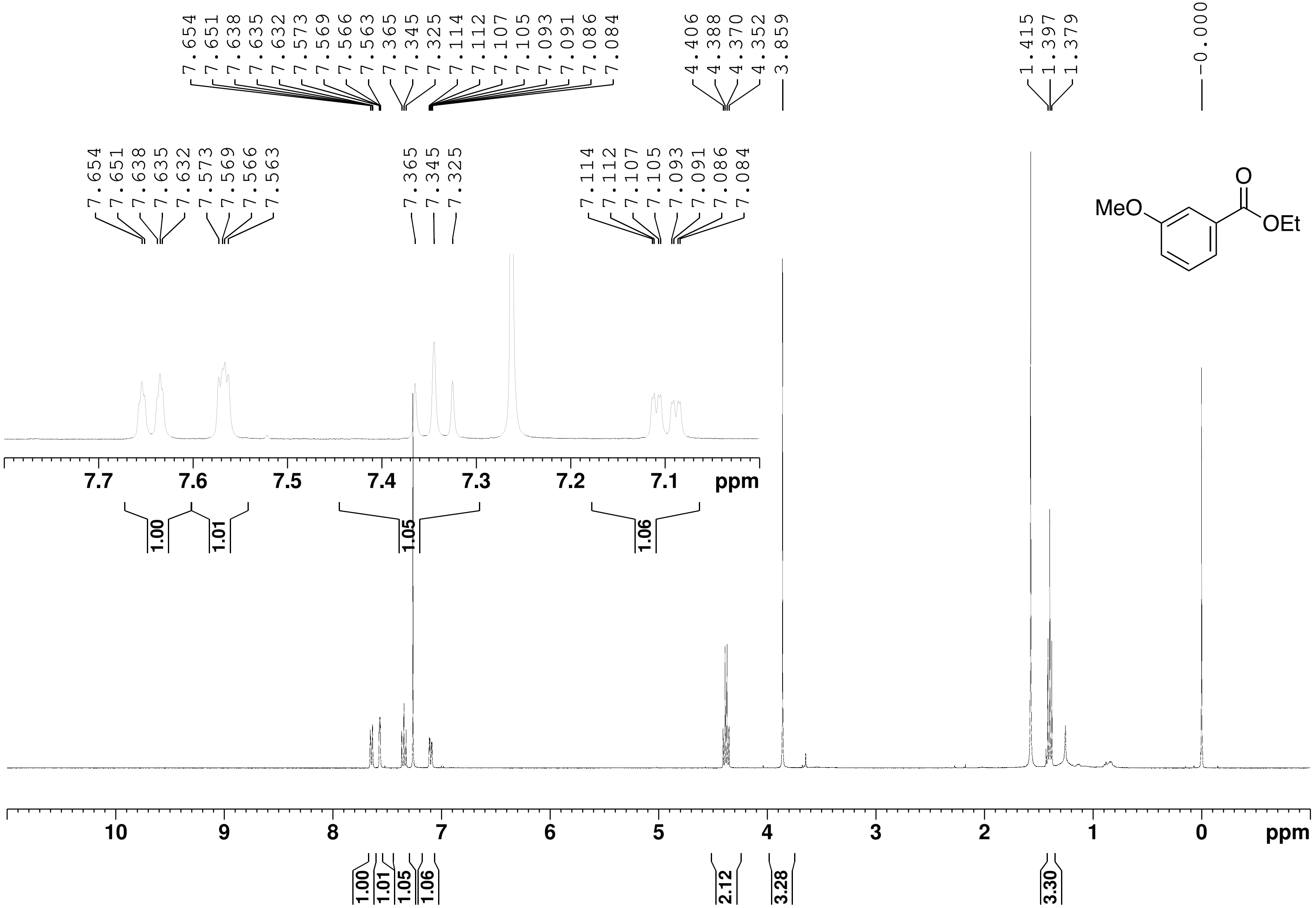

^{13}C NMR of **3ia** (100.6 MHz, $CDCl_3$)

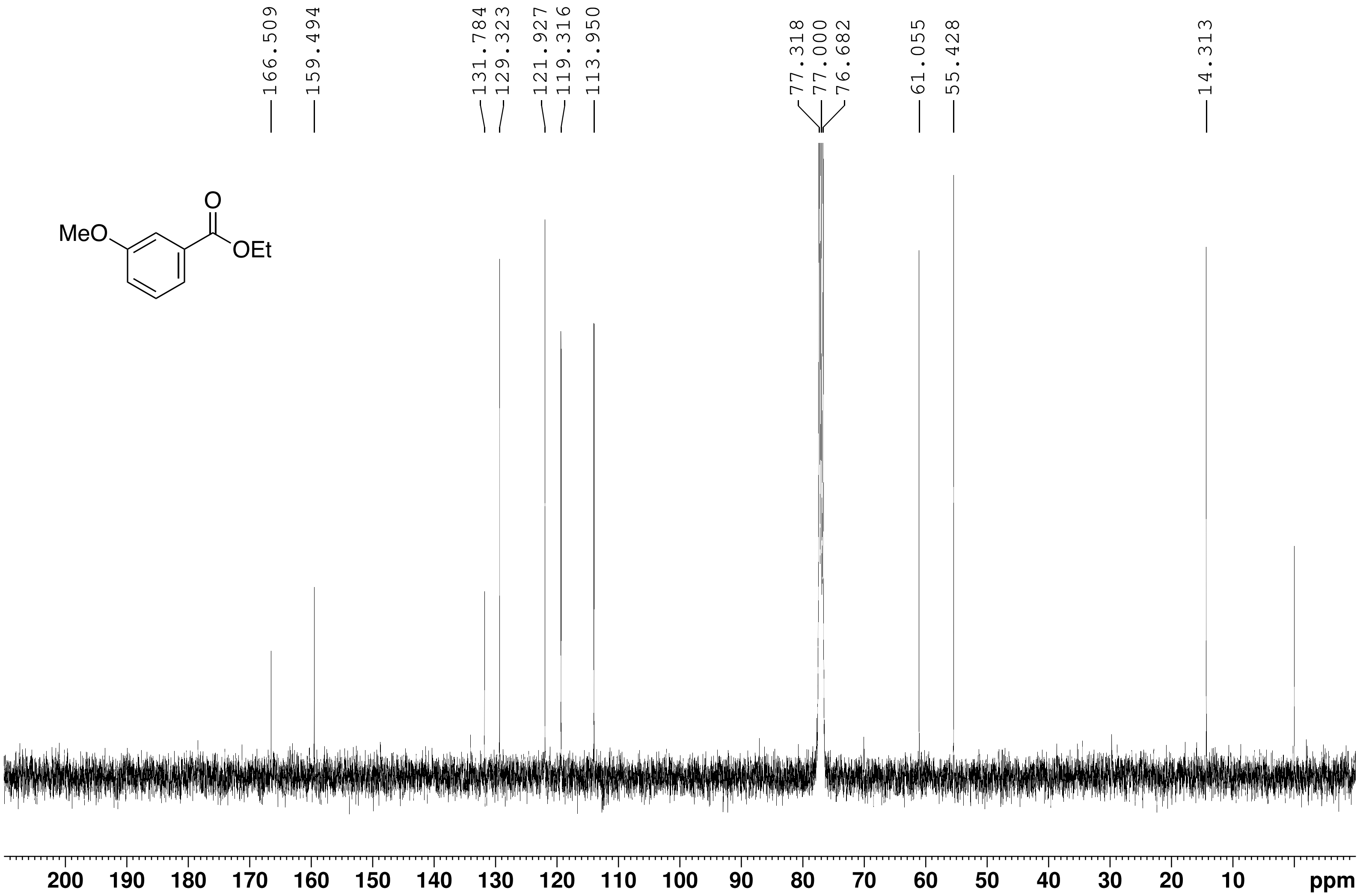

Supplement: SC-016-D4SC08407A-s001 [file SC-016-D4SC08407A-s001.pdf]
